# Bibliometric Analysis of Renal Fibrosis in Diabetic Kidney Disease From 1985 to 2020

**DOI:** 10.3389/fpubh.2022.767591

**Published:** 2022-02-04

**Authors:** Yuqing Zhang, De Jin, Yingying Duan, Yuehong Zhang, Liyun Duan, Fengmei Lian, Xiaolin Tong

**Affiliations:** ^1^Endocrinology Department, Guang'anmen Hospital, China Academy of Chinese Medical Sciences, Beijing, China; ^2^Endocrinology Department, Guang'anmen Hospital, Beijing University of Chinese Medicine, Beijing, China; ^3^Endocrinology Department, Affiliated Hospital to Changchun University of Chinese Medicine, Changchun, China

**Keywords:** bibliometrics, diabetic kidney disease, renal fibrosis, CiteSpace, cluster analysis

## Abstract

**Background:**

Diabetic renal fibrosis (DRF) is an irreversible renal pathological change in the end-stage of diabetic kidney disease (DKD), which plays a significant role in the development and deterioration of the disease. However, data for bibliometric analysis of renal fibrosis in DKD is currently missing. This study aimed to provide a comprehensive and visualized view of DRF research and lay the foundation for further studies.

**Materials and Methods:**

Firstly, the data was collected from the Web of Science Core Collection (WoSCC) database. Secondly, the Web of Science analytic tool was performed to analyze publication years, authors, countries/regions, organizations, and citation frequency. Finally, CiteSpace was employed to construct a visualization bibliometric network to reveal the emerging trends and hotspots of DRF.

**Results:**

A total of 3,821 publications from 1985 to 2020 were included in this study. The number of publications has maintained a growth trend since 2003. Cooper is the most prolific author in this field, and the *American Journal of Physiology-Renal Physiology* ranking as first place compared with other journals. In terms of the number of publications, China contributed the most to DRF. Monash University is the organization that published the most papers. The top 5 clusters of keyword co-appearance are “chronic kidney disease”, “primary biliary cirrhosis”, “receptor”, “TGF-beta”, “renal tubulointerstitium”. The top 5 clusters of reference co-citation are “microRNAs”, “bone morphogenetic protein”, “hypertrophy”, “glomerulosclerosis”, “diabetic kidney disease”. The strongest citation burst of keyword is “diabetic kidney disease” and the strongest burst of cited reference is “Meng, 2016”.

**Conclusions:**

The present study analyzed the research hotspots, Frontiers, and development trend of DRF and have important implications for future research.

## Introduction

The past few decades have witnessed an increasing development in the field of renal fibrosis, and its importance in diabetic kidney disease (DKD) has been explored. Diabetic renal fibrosis (DRF) is an irreversible renal pathological change in the late stage of DKD, characterized by massive collagen deposition, extracellular matrix (ECM) accumulation, renal parenchyma sclerosis, and scar formation (1, 2). In high glucose conditions, the kidney is stimulated by various pathogenic factors that promote the renal intrinsic cells undergo some pathological transformation including epithelial-to-mesenchymal transition (EMT), endothelial-to-mesenchymal transition (EndoMT), and fibroblasts and pericytes activation (3), eventually causing the formation of myofibroblasts (4). Then, a large amount of collagen is secreted by myofibroblasts (5), resulting in abnormal accumulation and deposition of extracellular matrix (ECM) (6), which eventually leads to glomerular sclerosis and fibrosis of renal tubules, renal interstitium, and renal vessels. DRF is a common pathway leading to the progressive decline of renal function and the development of end-stage renal failure in patients with DKD (7, 8), which plays a significant role in the progression and deterioration of the disease. Bohle et al. published the first paper on DRF on the Web of Science in 1991 and suggested that diabetes patients with renal interstitial fibrosis had a decreased survival rate (9). Due to its importance for the prognosis of DKD, an increasing number of studies have focused on the mechanism of DRF, and efforts have been made to explore novel therapies to alleviate renal fibrosis.

Bibliometric analysis was first proposed by Pritchard in 1969 to show the changes and characteristics of written communication in the development of a discipline (10). In 2004, Van Raan further deepened this method and introduced infographics into bibliometric analysis, such as bibliometric mapping, showing the achievements and development of a field from a clearer and more intuitive perspective (11). Through the continuous improvement of researchers, bibliometric analysis methods have become mature. Based on the online database, basic information of literature including publication years, authors, journals, countries, institutions, and citing frequency could be obtained (12). With the use of the existing easy-to-use software, readers can quickly understand the research hotspots and Frontiers in a specific field, and grasp the future trends (13). In recent years, with the surge of medical academic papers, bibliometric analysis plays a more important role in the field of medicine. However, there is still a lack of data on the bibliometric analysis of diabetic renal fibrosis.

CiteSpace is a Java application designed for visual exploration of quantitative information and development trends in the field of scientific research (14). It could identify the nature of research Frontiers and timely discover new trends and abrupt changes (15). The main advantage of CiteSpace is that it can extract information from titles, keywords, abstracts, and generate clustering labels based on various algorithms (16). In terms of data processing, CiteSpace can slice data by time and use different filtering criteria for time slices (17). Therefore, CiteSpace was employed for the bibliometric analysis of DRF for its strong operability.

In this study, we retrieved DRF-related articles from the Web of Science database and analyzed the literature characteristics and research hotspots employing bibliometric analysis tools. The purpose of this study is to provide a comprehensive and visualized view of DRF research and to lay a solid foundation for further studies.

## Materials and Methods

The online literature data was collected from the Web of Science Core Collection (WoSCC) database through the Science Citation Index Expanded (SCI-EXPANDED) on April 30, 2021. Our search strategy was: TS= (renal fibrosis OR renal interstitial fibrosis) AND TS= (diabetic kidney disease^*^ OR diabetic nephropath^*^ OR diabetes) AND PY= (1985-2020) AND language=(English). Only articles and reviews meet the requirement of this study and were included in the analysis. The literature retrieval and download were conducted by two authors independently (YQZ and DJ). After the data verification and standardization, the online literature including full records and cited references were exported in the plain text format. These records were then imported into CiteSpace 5.7.R2 for visualized analysis.

The Web of Science analytic tool was performed to analyze some general publication characteristics, including publication years, authors, countries/regions, organizations, and citation frequency. Microsoft Excel 2019 was conducted to draw the column diagram as well as predict the growth trend of publications using the existing data.

The CiteSpace 5.7.R2 was employed to construct a visualization bibliometric network based on literature information collected from the dataset, such as titles, abstracts, keywords, references, descriptors, and identifiers (18). It analyzed the emerging trends and hotspots of DRF in terms of keyword co-appearance network, reference co-citation network, and citation burst detection (16, 19).

## Results

### Annual Literature Quantity and Growth Prediction

**Figure 1:**
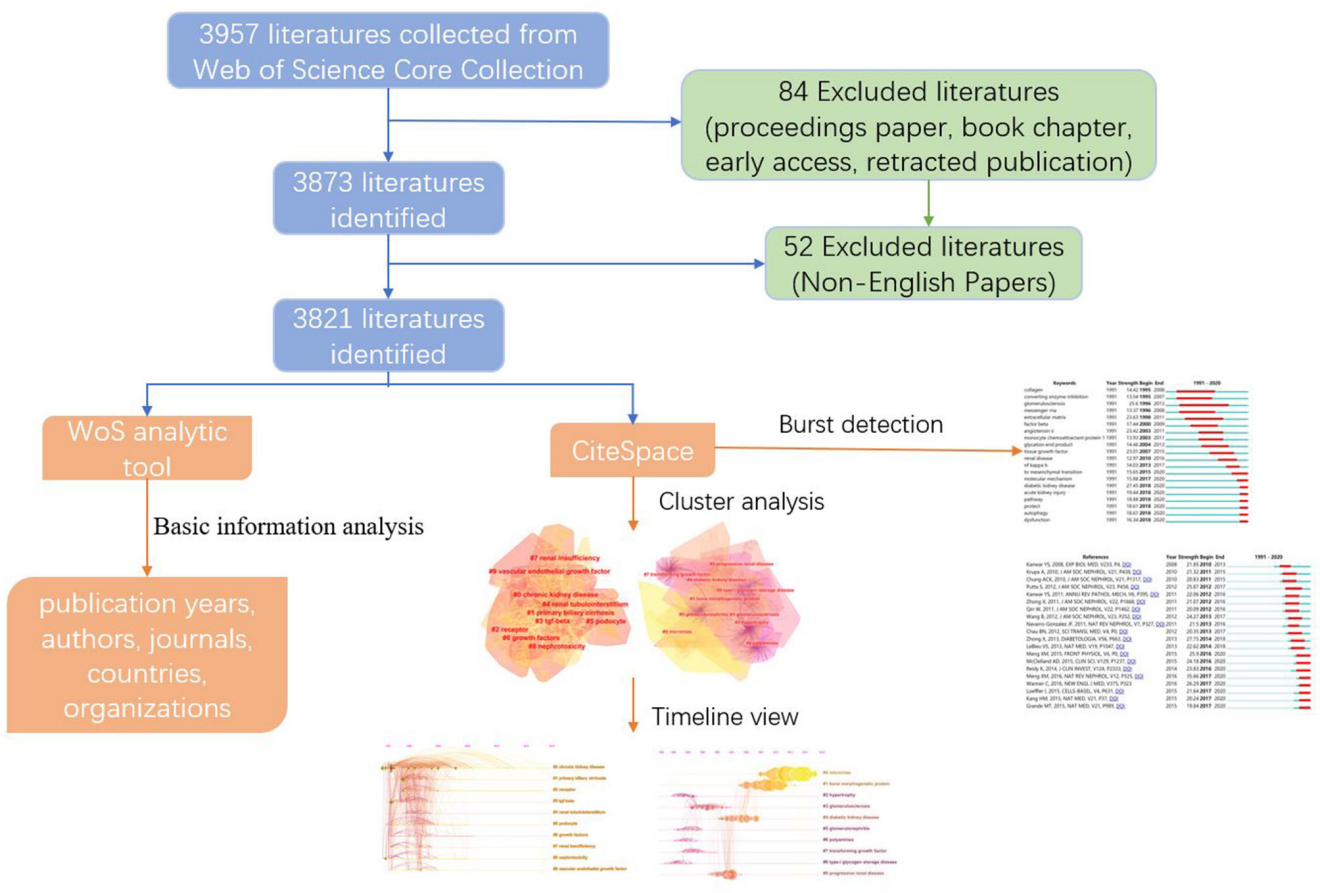
Bibliometric analysis of renal fibrosis in DKD presented in the workflow. DKD, diabetic kidney disease.

**Figure 2:**
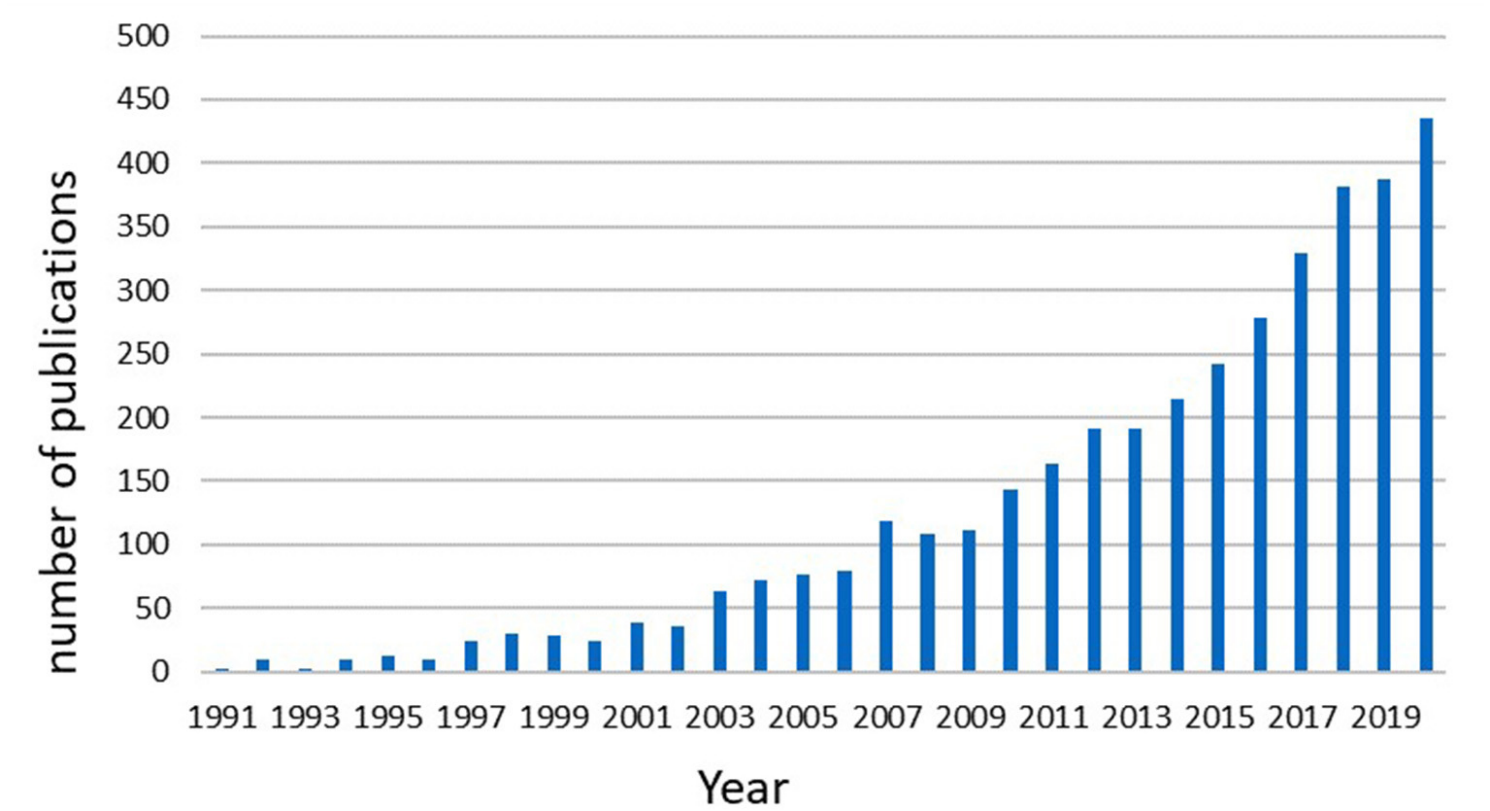
The annual quantities of diabetic renal fibrosis publications.

**Figure 3:**
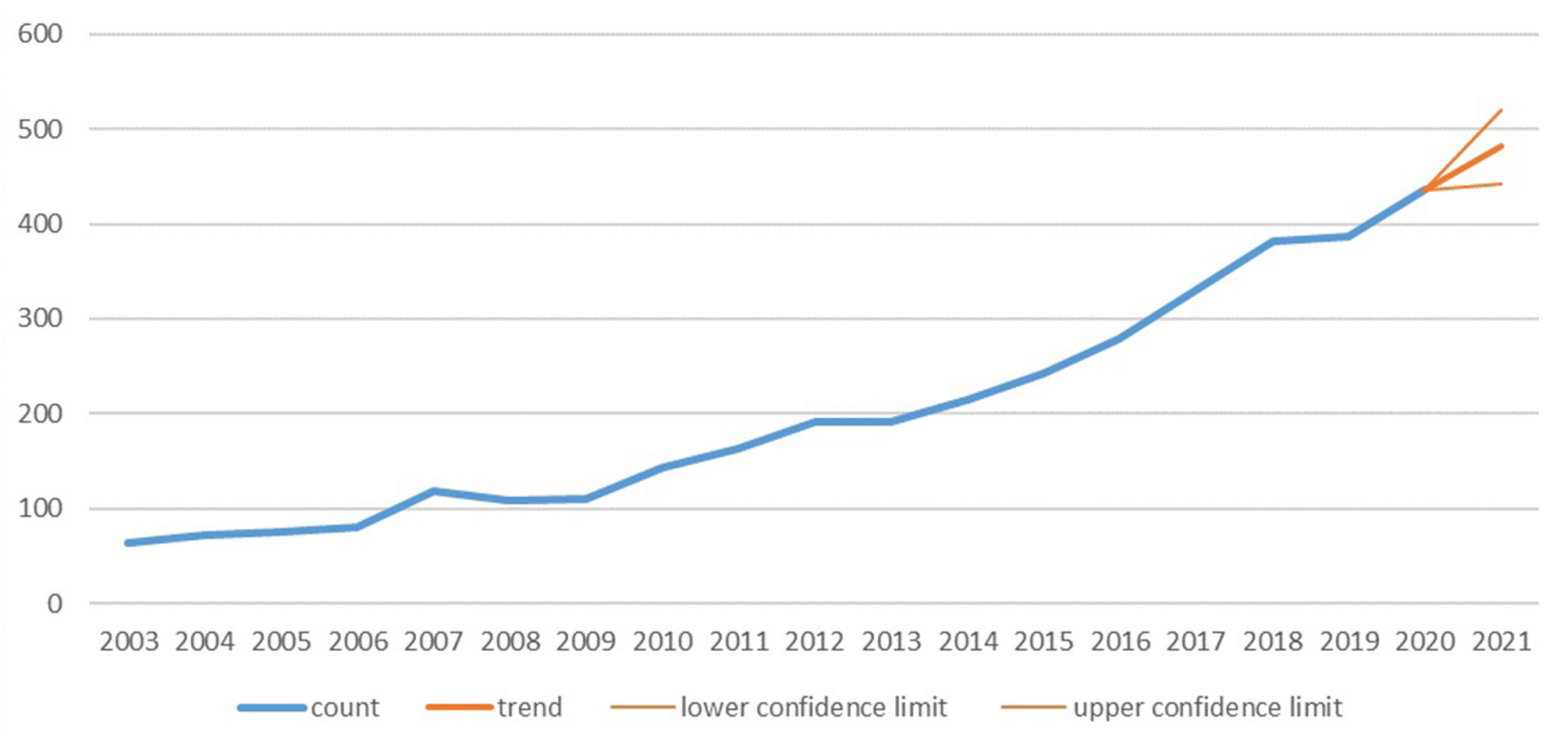
The growth trend and prediction of diabetic renal fibrosis.

According to the workflow presented in Figure 1, a total of 3,821 publications from 1985 to 2020 were included in this analysis. The first article that related to diabetic renal fibrosis was published in WoSCC in 1991, and the number of publications kept a sustained growth trend since 2003 (Figure 2). The record count reached 436 in 2020, indicating that renal fibrosis is an emerging focus of diabetes-related disease. Microsoft Excel 2019 self-contained forecast worksheet was performed to predict the growth trend of DRF and depict the polynomial fitting curve based on the data from 2003 to 2020. As shown in Figure 3, the DRF-related publications will reach approximately 482 in 2021. In general, although studies on DRF have increased significantly in the past few decades, it is still a relatively new research field that needs to be further explored.

### Distribution of Literature

Based on the WOSCC database, the distribution of authors, journals, countries, and organizations of literature were analyzed. More than 15,088 authors contributed to the 3,821 DRF-related literatures published in WoSCC. Among the 20 most prolific authors, Cooper takes first place with 47 articles, followed by Lan and Li, both with 44 articles. Zhang and Wang are in fourth and fifth places with 43 and 42 literatures, respectively. More than half of the top 20 authors come from China, indicating that diabetic renal fibrosis is a hot topic among Chinese authors (Supplementary Materials 1, 2).

The publications of diabetic renal fibrosis are distributed among more than 783 journals. The *American Journal of Physiology-Renal Physiology* ranking as the first has published 169 articles on the subject of DRF, which accounts for 4.423% of all the articles. It is followed by *Kidney International* (166 publications), *Journal of the American Society of Nephrology* (132 publications), *PLoS ONE* (104 publications), and *Neurology Dialysis Transplantation* (91 publications) (Supplementary Materials 3, 4). Impact factor (IF) is a relative statistic that acts as an important indicator to measure the academic level of the journal and the quality of the publications. Among the top 5 articles, the *Journal of the American Society of Nephrology* has the highest IF which reaches 9.274. The IF of the other four journals named *American Journal of Physiology-Renal Physiology, Kidney International, PLoS ONE*, and *Neurology Dialysis Transplantation* are 3.191, 8.945, 2.74, and 4.531 respectively. In general, most of the DRF-related articles published in these journals are of high quality and worth further analysis.

According to the retrieval results of the WoSCC database, 54 countries have published literature focusing on diabetic renal fibrosis. China contributed the most with 1,165 publications, followed by the United States (1,052 publications), Japan (363 publications), Australia (243 publications), and Germany (236 publications) (Supplementary Materials 5, 6). In terms of the organizations, there are 3,304 institutes involved in the field of DRF. According to the number of DRF-related publications, Monash University ranked the first with 82 articles on diabetic renal fibrosis, followed by the University of Melbourne (57 articles), University of Sydney (57 articles), China Medical University (56 articles), and Sun Yat-Sen University (55 articles). Organizations from Australia and China occupied the top 9 places (Supplementary Materials 7, 8). Given the above, China keeps its leading role in the field of DRF, and Monash University is at the top of the rankings in terms of the number of articles issued by the organizations.

### Cluster Analysis of Keyword Co-appearance

The keyword is a concise word used in indexing or cataloging to describe the subject of the article succinctly and accurately. Therefore, we could have a general understanding of the theme and characteristics of publications through the analysis of keywords. A merged network of keyword co-appearance with 1,046 nodes and 5,444 links was constructed using CiteSpace 5.7.R2 software. The parameters of the software were set as follows. Time slicing: from 1991 to 2020 and 1 year per slice; node types: keyword; selection criteria: select top 50 levels of most occurred items from each slice; pruning: pathfinder, pruning sliced networks, and the merged network.

**Figure 4:**
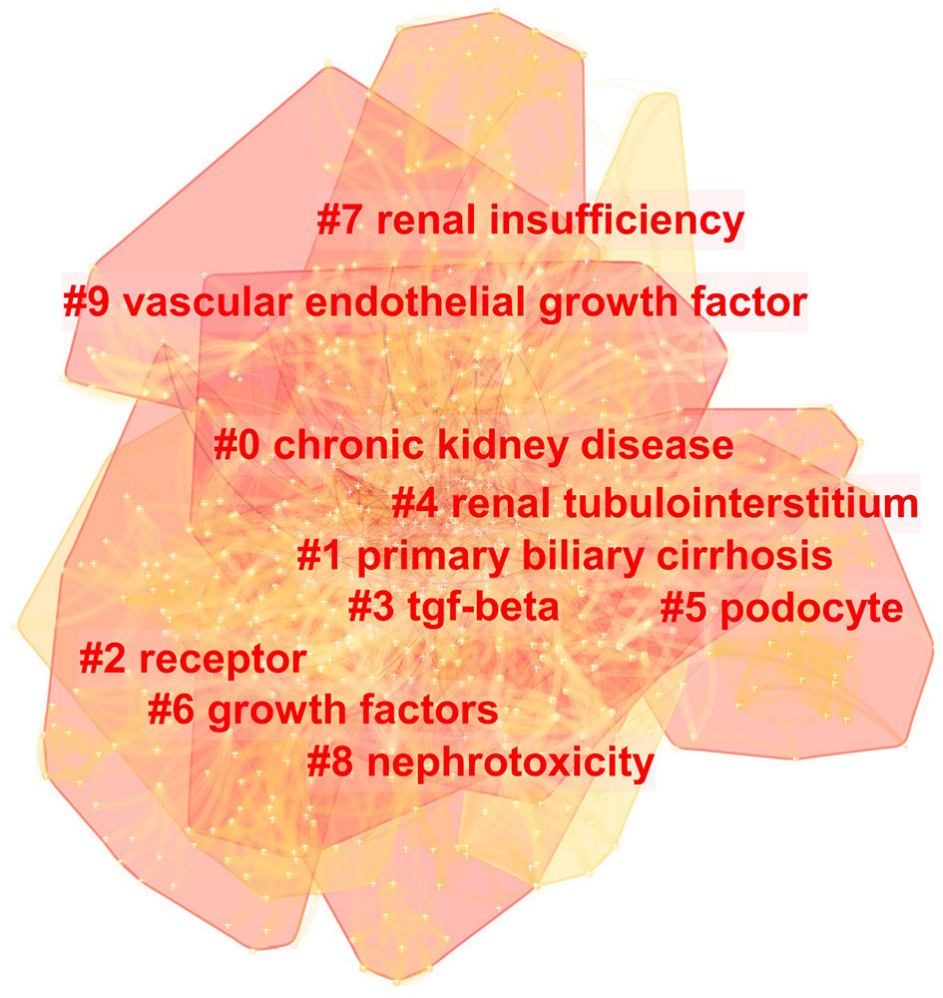
Cluster analysis of keyword co-appearance in diabetic renal fibrosis.

**Figure 5:**
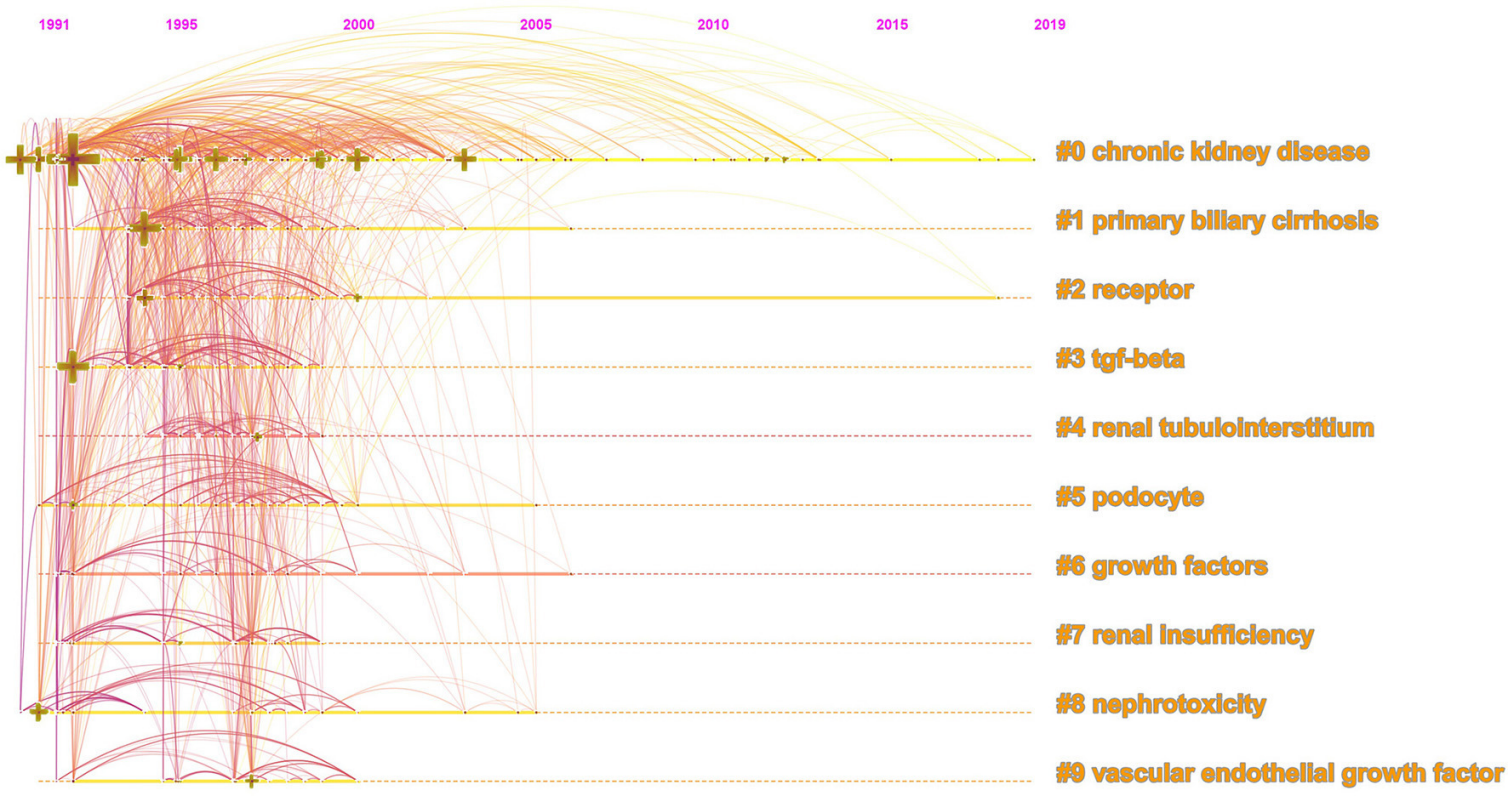
The timeline view of keywords co-appearance in diabetic renal fibrosis.

The top 20 most occurred keywords were listed in Table 1. “Diabetic nephropathy” was most frequently used as the keyword in the literature, followed by “fibrosis”, “expression”, “renal fibrosis”, “nephropathy”, “oxidative stress”, “kidney”, “inflammation”, “TGF-β” and “disease”. Cluster analysis was conducted according to these keywords, and the result was presented in Figure 4. The top 5 clusters ranked by cluster size are “chronic kidney disease”, “primary biliary cirrhosis”, “receptor”, “TGF-beta”, “renal tubulointerstitium”. The timeline view of keywords co-appearance showed in Figure 5 demonstrates the development and variation of the keywords in each cluster.

**Table 1:** Top 20 keywords of diabetic renal fibrosis.

**Ranking**	**Counts**	**Centrality**	**Keywords**
1	1,696	0.04	Diabetic nephropathy
2	930	0.02	Fibrosis
3	811	0.02	Expression
4	565	0.01	Renal fibrosis
5	553	0.03	Nephropathy
6	510	0.01	Oxidative stress
7	487	0.04	Kidney
8	485	0.01	Inflammation
9	449	0.01	TGF-beta
10	422	0.02	Disease
11	405	0.02	Chronic kidney disease
12	357	0.04	Growth factor beta
13	352	0.01	Kidney disease
14	350	0.02	Activation
15	334	0.01	Mechanism
16	333	0.02	Injury
17	288	0.01	Cell
18	284	0.02	Progression
19	267	0.03	Mesangial cell
20	265	0.03	Angiotensin II

### Cluster Analysis of Reference Co-citation

Reference co-citation clustering is a superior function of CiteSpace, which gives us an intuitive understanding of research topics and hotspots. Each cluster is considered to represent a research Frontier to some extent. Therefore, CiteSpace was conducted to depict the cluster view and timeline view of reference co-citation, to analyze the trend of diabetic renal fibrosis. The parameters of the software were set as follows. Time slicing: from 1991 to 2020 and 1 year per slice; node types: reference; selection criteria: select top 30 levels of most occurred items from each slice.

**Figure 6:**
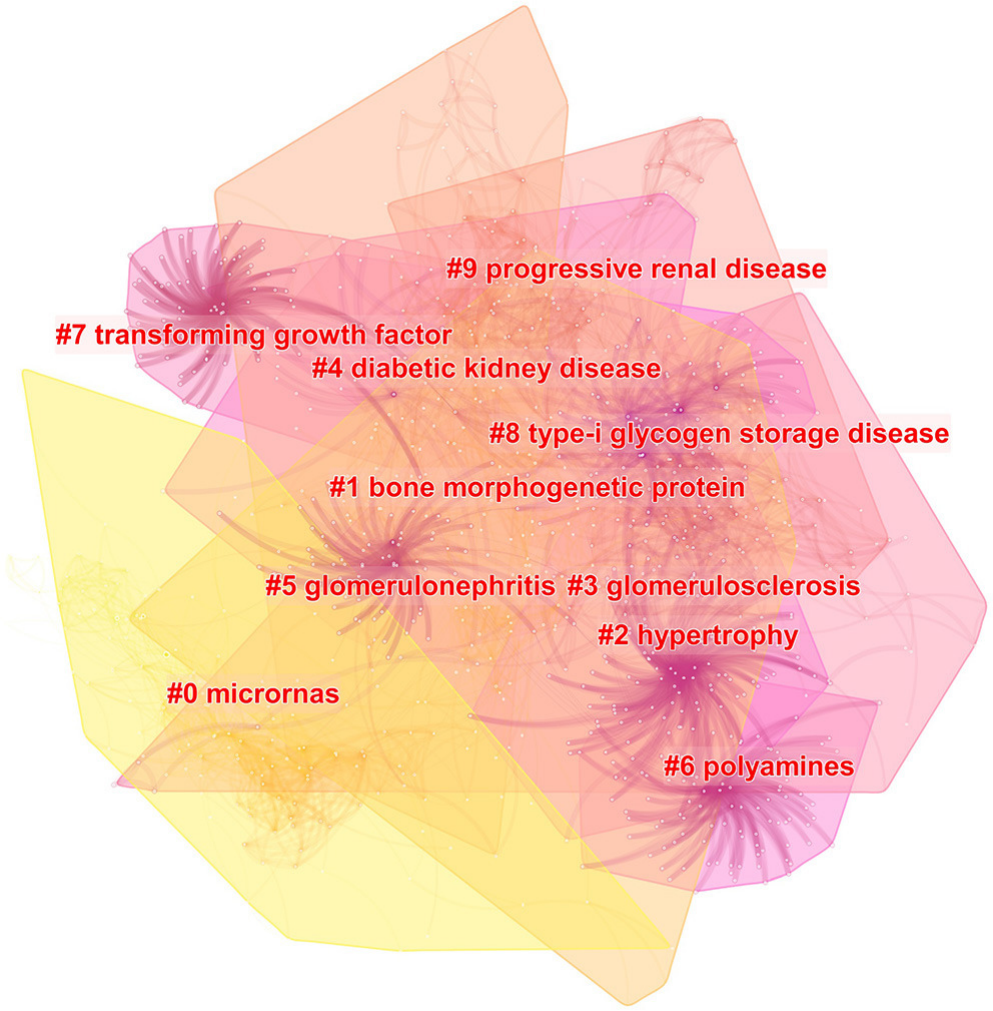
Cluster analysis of reference co-citation in diabetic renal fibrosis.

**Figure 7:**
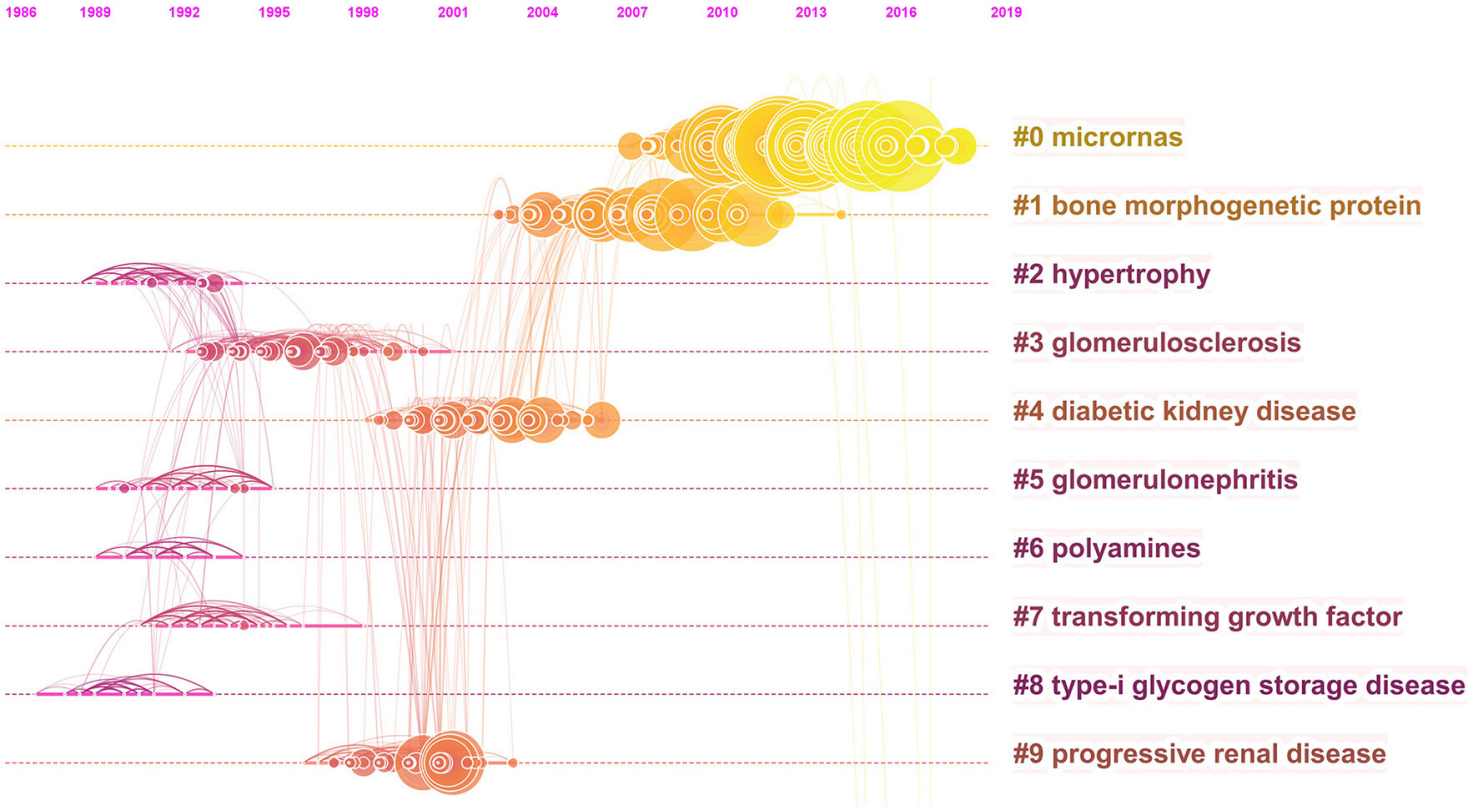
The timeline view of reference co-citation in diabetic renal fibrosis.

A merged network of reference co-citation with 1,646 nodes and 6,270 links was constructed for clustering. The top 20 most cited references were presented in Table 2. The top two papers were from the same author Meng, published in 2015 and 2016, respectively. In addition, the result of the cluster analysis was presented in Figure 6. The top 5 clusters ranked by cluster size are “microRNAs”, “bone morphogenetic protein”, “hypertrophy”, “glomerulosclerosis”, “diabetic kidney disease”. The timeline view of references-cited co-appearance shown in Figure 7 reflects the change of the hotspots of cited references over time.

**Table 2:** Top 20 cited references of diabetic renal fibrosis.

**Ranking**	**Counts**	**Centrality**	**Publication year**	**Author**	**Journal**	**Vol**	**Page**
1	77	0.01	2016	Meng, X. M.	Nat. Rev. Nephrol.	12	325
2	61	0.01	2015	Meng, X. M	Front. Physiol.	6	0
3	60	0.06	2013	Zhong, X.	Diabetologia	56	663
4	59	0.02	2012	Putta, S	J. Am. Soc. Nephrol.	23	458
5	57	0	2016	Wanner, C.	N. Engl. J. Med.	375	323
6	57	0.02	2015	McClelland, A. D.	Clin. Sci.	129	1237
7	50	0.01	2012	Wang, B.	J. Am. Soc. Nephrol.	23	252
8	49	0.01	2013	LeBleu, V. S.	Nat. Med.	19	1047
9	49	0.01	2014	Reidy, K.	J. Clin. Invest.	124	2333
10	47	0.01	2015	Loeffler, I.	Cells	4	631
11	45	0	2011	Kanwar, Y. S.	Annu. Rev. Pathol. Mech.	6	395
12	44	0.02	2015	Kang, H. M.	Nat Med	21	37
13	44	0.11	2010	Krupa, A.	J. Am. Soc. Nephrol.	21	438
14	43	0.01	2010	Chung, A. C. K.	J. Am. Soc. Nephrol.	21	1317
15	43	0.01	2011	Zhong, X.	J. Am. Soc. Nephrol.	22	1668
16	42	0.03	2012	Chau, B. N.	Sci. Transl. Med.	4	0
17	41	0.01	2011	Qin, W.	J. Am. Soc. Nephrol.	22	1462
18	40	0.01	2011	Navarro-Gonzalez, J. F.	Nat. Rev. Nephrol.	7	327
19	40	0.01	2008	Kanwar, Y. S.	Exp. Biol. Med.	233	4
20	38	0	2015	Grande, M. T.	Nat. Med.	21	989

### Burst Detection of Keywords and References

**Figure 8:**
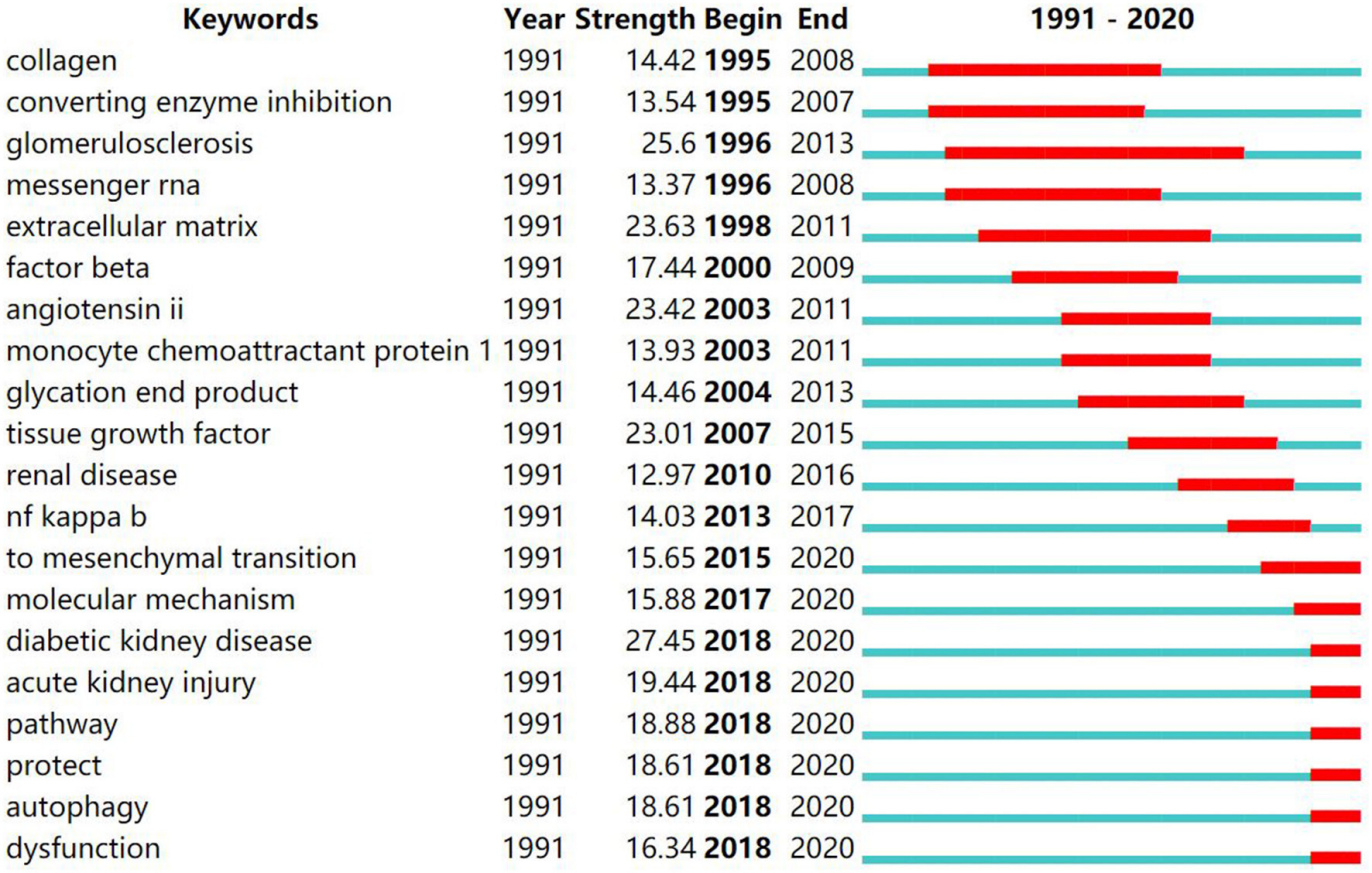
The burst detection of keywords in diabetic renal fibrosis.

**Figure 9:**
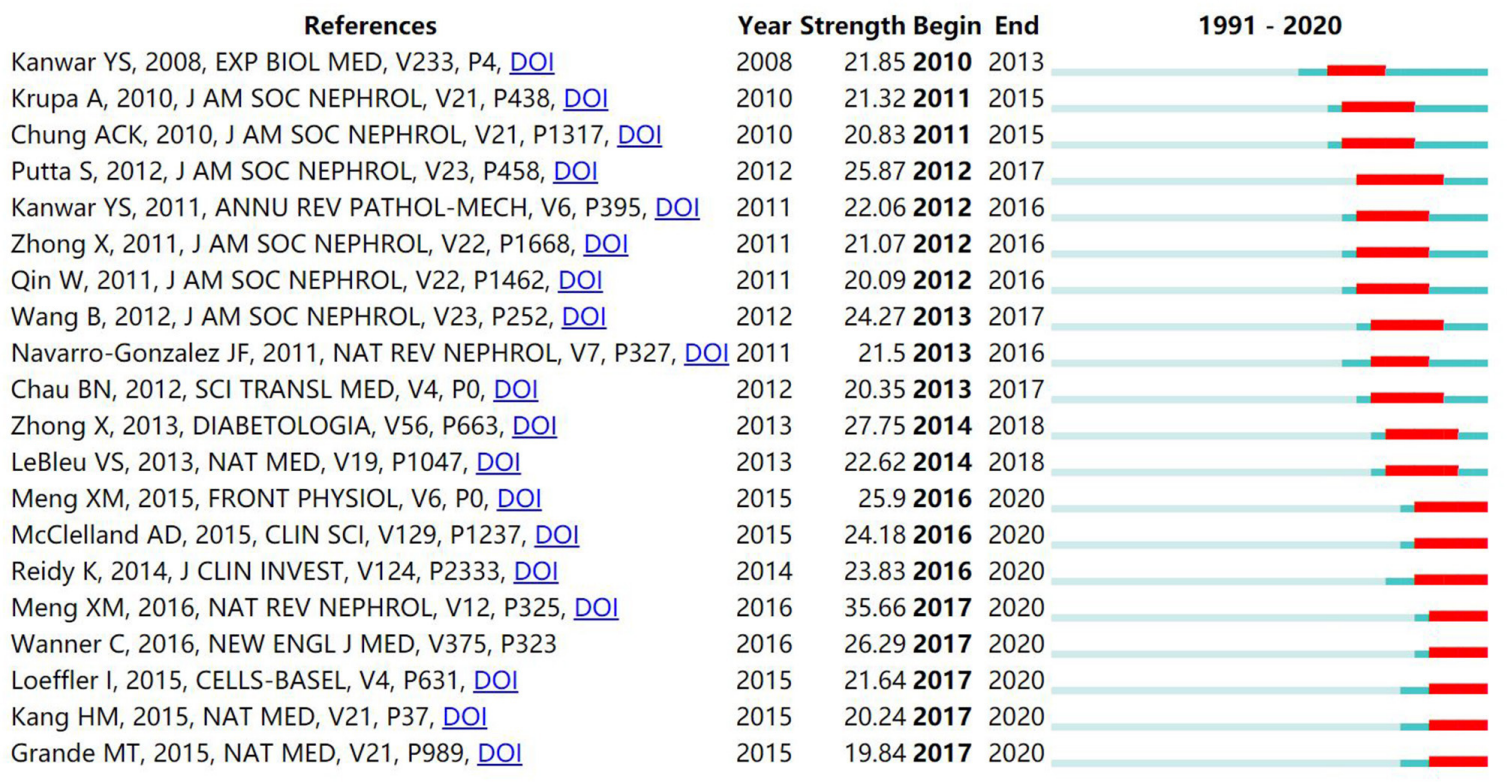
The burst detection of cited references in diabetic renal fibrosis.

CiteSpace provides the burst detection function to detect great changes in the amount of reference in a certain period. It is used to discover the decline or rise of a certain subject term or keyword. The top 20 keywords (Figure 8) and top 20 cited references (Figure 9) were screened according to the strength of the burst and were listed by the burst time. The strongest citation burst of keyword is “diabetic kidney disease (27.45)”, and the ones that burst most recently are “dysfunction (16.34)”, “autophagy (18.61)”, “protect (18.61)”, “pathway (18.88)”, “acute kidney injury (19.44)” and “diabetic kidney disease (27.45)”. In addition, the strongest citation burst of cited reference is “Meng, 2016 (35.66)”, and the top 5 references ranked by burst time are “Grande, 2015 (19.84)”, “Kang, 2015 (20.24)”, “Loeffler, 2015 (21.64)”, “Wanner, 2016 (26.29)”, and “Meng, 2016 (35.66)”.

## Discussion

DKD is one of the most common complications of diabetes and it also becomes the major cause of the end-stage renal disease (ESRD). The progress of DKD not only affects the patients' quality of life but also brings a heavy economic burden to society (20). Due to the complexity and irreversibility of renal fibrosis, it plays a crucial role in the development of DKD. Hence, prevention and treatment of renal fibrosis are of decisive significance for the prognosis of DKD, which needs our attention and concern. Bibliometric analysis is an effective and feasible literature analysis method, which can capture the characteristics and hot spots of literature and help people quickly understand an unfamiliar field. Therefore, a comprehensive understanding of the field could be obtained by using the bibliometric analysis method, which contributes a lot to subsequent research and clinical treatment. In this paper, we performed a bibliometric analysis of diabetic renal fibrosis to explore its status of development.

Our study has included 3,821 articles related to DRF from the WoSCC database for bibliometric analysis and visualization. Over the past few decades, the quantity of DRF-related publications shows an overall upward trend, and the number is expected to further increase in 2,021. It demonstrates that renal fibrosis has attracted more and more attention as an important and irreversible pathological change in the progression of DKD. In terms of the authors, the top 20 prolific authors have all published 25 or more articles on DRF. The articles published by the top 20 authors account for 17% of all the published papers, making great contributions to the development and progress of DRF. In particular, Cooper emerged as the most prolific author with 47 articles. Among the top 20 medical journals, the *American Journal of Physiology-Renal Physiology* has published the most literature related to DRF, while the *Journal of the American Society of Nephrology* has the highest IF that reaches 9.274, showing the superiority in quantity and quality. Furthermore, 12 of the top 20 journals with the most publications on DRF are American journals, indicating the strong interest and leadership of the United States in this field. Among organizations, Monash University located in Australia has published the most articles. Concerning countries, China leads the way in the number of publications, closely followed by the United States. According to the report by the International Diabetes Federation (IDF), China ranked first in the number of adults (20–79 years old) with diabetes in 2019, with 116.4 million cases, while the United States ranked third with 31.0 million patients (https://www.diabetesatlas.org/en/resources/), and this situation is anticipated to remain so until 2030. Therefore, the leading position that these countries took may partly attribute to the high prevalence rate of diabetes in these countries, as well as the emphasis on medical research.

According to the cluster analysis of keyword co-appearance and reference co-citation, the top ten clusters marked by clusters 0–9 were obtained and displayed in Figures 4, 6, respectively. These clusters represent the keywords or terms that appear most frequently in the literature, therefore reflect the hotspots in a period. Cluster 3 “TGF-β”, cluster 6 “growth factors”, and cluster 9 “vascular endothelial growth factor” in Figure 4, as well as cluster 1 “bone morphogenetic protein” and cluster 7 “transforming growth factor” in Figure 6, are all related to growth factors, indicating the crucial role they play in the process of DRF. The most studied growth factors are transforming growth factor-β (TGF-β), vascular endothelial growth factor (VEGF), and bone morphogenetic protein (BMP). Studies show that the above growth factors are the key link in the formation and progression of fibrosis (21). They cause the proliferation and differentiation of fibroblasts in the renal interstitium and accelerate their transformation into myofibroblasts (22). Among them, the TGF-β discovered by De Larco (23) in 1980 has the strongest correlation with DRF. Therefore, growth factors are of concern in the field of DRF, and they need to be further studied. Cluster 5 “podocyte” is another hotspot of DRF. A lot of studies have indicated that podocyte dysfunction, including inflammation, foot process fusion, hypertrophy, and apoptosis, is one of the major causes of albuminuria and renal fibrosis in DKD (24, 25). Studies have shown that ameliorating podocyte injury can improve renal function and manage renal fibrosis (26–28), indicating the important research direction of podocyte protection. Terms of related disease such as “glomerulosclerosis”, “glomerulonephritis”, “renal insufficiency”, and “progressive renal disease” were presented in the cluster analysis results, indicating a close relationship between DRF and renal disease. The results of the DRF study are of certain reference significance to those of renal disease fibrosis. Combined with the cluster analysis and the timeline view of references, we could find that microRNAs (miRNAs) are not only the largest clustering but also the emerging research Frontier in recent years. More attention has been paid to miRNAs for their potential fibrotic or antifibrotic effects (29). Researchers found that the dysregulated expression of miRNAs accelerated the ECM accumulation and stimulated pro-fibrotic signaling, finally leading to renal fibrosis (30). The focuses of current studies are to inhibit the expression of miRNAs that induce renal fibrosis and increase the expression of antifibrotic miRNAs (31). To sum up, the use of RNA interference technology may become a hot topic in the future prevention and treatment of diabetic renal fibrosis.

According to the burst detection displayed in Figure 8, keywords related to DRF mechanisms have been frequently mentioned. Collagen has been the focus of research from 1995 to 2008 with a burst strength of 14.42, and extracellular matrix from 1998 to 2011 with a burst strength of 23.63. Researchers found that myofibroblasts transformed from renal cells promote the production of collagen and ECM, which accelerates the fibrotic process (32). Therefore, efforts have been made to block the transformation of intrinsic renal cells and slow the secretion of collagen and the accumulation of ECM (33, 34). In addition, burst analysis shows that EMT has been the hot topic of research from 2015 to 2020. It is a key link in fibrogenesis under the stimulation of various signaling pathways, which promotes the formation of myofibroblasts and the secretion of collagen (35). Researchers found that targeted inhibition of EMT effectively reduces collagen production and ECM accumulation, ultimately alleviates renal fibrosis (36). To sum up, understanding the pathological mechanism of DRF is essential for the treatment of disease and new drug discovery, which promotes researchers to attach importance to the mechanism of DRF. In recent years, terms such as “pathway”, “protect”, “autophagy”, and “dysfunction” have been frequently discussed in DRF literature. The study of signaling pathways is the basis of the new drug discovery. Multiple pathways involved in DRF including TGF-β, PI3K/Akt, MAPK, JAK/STAT, mTOR, Wnt/β-catenin, Hippo, and Notch pathways and their related molecular mechanisms have been well-studied (37–42). The blocking of these pathways provides novel therapeutic strategies for DRF. As shown in Figure 9, the article published by Meng in 2016 has the highest burst strength 35.66. It shows that this author not only published the most articles but also took the lead in the quality of the publications, making great contributions to the research in the field of DRF.

In conclusion, the current research hotspots mainly focus on the exploration of the pathological mechanism of DRF. Researchers have made efforts to delay the progression of renal fibrosis by blocking some of the key pathological processes, such as podocyte injury, ECM accumulation, and EMT (5, 43). They found a variety of signaling pathways involved in renal fibrosis, such as TGF-β, MAPK, PI3K/Akt, JAK/STAT, Wnt/β-catenin, and Notch pathways. The inhibition of related signaling pathways has been demonstrated to block the pathological progression of fibrosis. In addition, the crucial role of miRNAs in renal fibrosis has been observed by researchers (29), indicating that RNA interference technology may become an important tool in DRF. However, studies on DRF have mostly remained in animal experiments. There is still a lack of clinical trials in this area. At present, there are no agents approved to treat renal fibrosis in clinical practice (44). Also, the diagnosis of renal fibrosis is difficult, and invasive biopsy remains the gold diagnostic standard (45), which caused great hindrance to the new drug design and clinical efficacy evaluation. Hence, the development of non-invasive examinations and new anti-fibrotic drugs would be desired in future research endeavors.

This paper analyzes the research trends and hotspots of DRF, which will benefit many researchers. On the one hand, researchers can refer to the research trend to avoid some outdated research on selected topics, reduce the repetitive work of research projects and reduce the waste of project funds. On the other hand, researchers can optimize and improve their research design based on research hotspots, making DRF research more innovative and feasible. At the same time, this study provides a timeline of changes in DRF research. It provides the research changes of DRF in the past, present, and future for the implementation of health care and the formulation of health policy, lays a foundation for the precise prevention and treatment of DRF, and provides an essential reference value for the formulation of DKD guidelines and the adjustment of medical insurance policies. Ultimately, more patients will benefit from these, thus reducing the medical burden as well as the corresponding economic pressure of DKD prevention and treatment around the world.

However, the limitations of this study must be taken into account when analyzing the results. Firstly, only literature written in English was included in this study, which may lead to bias in the study results. Secondly, we only retrieved the data from the WoSCC database and did not search information from other databases, resulting in incomplete literature collection. Thirdly, there is no systematic standard for parameter setting and analysis method of the CiteSpace software, which may cause differences in results. Therefore, the design of this study still needs to be further improved.

## Conclusion

Focusing on the global research results of diabetic renal fibrosis, the present study analyzed the research hotspots, Frontiers, and development trends in this field. In the past decades, the number of literatures related to DRF has been on the rise, which indicates that this emerging field has attracted more and more attention. Our findings are a summary of the current state of DRF research and have important implications for future research direction.

## Data Availability Statement

The original contributions presented in the study are included in the article/Supplementary Material, further inquiries can be directed to the corresponding author.

## Author Contributions

YuqZ and DJ contributed to conception and design of the study. YuqZ collected data from the database and wrote the first draft of the manuscript. DJ, YD, YueZ, and LD wrote sections of the manuscript. All authors contributed to manuscript revision, read, and approved the submitted version.

## Funding

This work was funded by the 2015 Traditional Chinese Medicine Scientific Research (No. 201507001-11).

## Conflict of Interest

The authors declare that the research was conducted in the absence of any commercial or financial relationships that could be construed as a potential conflict of interest.

## Publisher's Note

All claims expressed in this article are solely those of the authors and do not necessarily represent those of their affiliated organizations, or those of the publisher, the editors and the reviewers. Any product that may be evaluated in this article, or claim that may be made by its manufacturer, is not guaranteed or endorsed by the publisher.
