# Supplementary material for: Bibliometric Analysis of Renal Fibrosis in Diabetic Kidney Disease From 1985 to 2020
Source: Front Public Health. 2022 Feb 4;10:767591. doi: 10.3389/fpubh.2022.767591 (PMC8855938; doi:10.3389/fpubh.2022.767591)
Supplement: Supplementary file 1 [file Table_1.DOCX]

Countries/Regions records % of 3821

PEOPLES R CHINA 1165 30.489

USA 1052 27.532

JAPAN 363 9.500

AUSTRALIA 243 6.360

GERMANY 236 6.176

SOUTH KOREA 155 4.057

ITALY 148 3.873

ENGLAND 146 3.821

CANADA 139 3.638

NETHERLANDS 120 3.141

FRANCE 115 3.010

TAIWAN 104 2.722

SPAIN 95 2.486

INDIA 77 2.015

SWITZERLAND 56 1.466

BRAZIL 52 1.361

DENMARK 47 1.230

IRELAND 39 1.021

TURKEY 39 1.021

SWEDEN 37 0.968

CHILE 33 0.864

BELGIUM 32 0.837

AUSTRIA 29 0.759

EGYPT 28 0.733

ISRAEL 27 0.707

MEXICO 26 0.680

WALES 26 0.680

POLAND 25 0.654

SCOTLAND 23 0.602

SAUDI ARABIA 21 0.550

ARGENTINA 20 0.523

GREECE 18 0.471

HUNGARY 18 0.471

ROMANIA 18 0.471

IRAN 17 0.445

THAILAND 16 0.419

NEW ZEALAND 14 0.366

NORTH IRELAND 14 0.366

PORTUGAL 12 0.314

FINLAND 11 0.288

LEBANON 11 0.288

SLOVAKIA 11 0.288

NORWAY 8 0.209

BANGLADESH 7 0.183

CZECH REPUBLIC 7 0.183

MALAYSIA 7 0.183

CROATIA 6 0.157

QATAR 6 0.157

RUSSIA 5 0.131

U ARAB EMIRATES 5 0.131

SINGAPORE 4 0.105

JORDAN 3 0.079

PAKISTAN 3 0.079

SERBIA 3 0.079

SOUTH AFRICA 3 0.079

COLOMBIA 2 0.052

GUINEA 2 0.052

KUWAIT 2 0.052

LUXEMBOURG 2 0.052

OMAN 2 0.052

SLOVENIA 2 0.052

TANZANIA 2 0.052

ALGERIA 1 0.026

AZERBAIJAN 1 0.026

BAHRAIN 1 0.026

BARBADOS 1 0.026

CUBA 1 0.026

CYPRUS 1 0.026

EL SALVADOR 1 0.026

ETHIOPIA 1 0.026

GUYANA 1 0.026

ICELAND 1 0.026

LATVIA 1 0.026

MOLDOVA 1 0.026

MOROCCO 1 0.026

NIGERIA 1 0.026

SUDAN 1 0.026

UGANDA 1 0.026

VENEZUELA 1 0.026

(2 records (0.052%) do not contain data in the field being analyzed.)
