# Supplementary material for: Bibliometric Analysis of Renal Fibrosis in Diabetic Kidney Disease From 1985 to 2020
Source: Front Public Health. 2022 Feb 4;10:767591. doi: 10.3389/fpubh.2022.767591 (PMC8855938; doi:10.3389/fpubh.2022.767591)
Supplement: Supplementary file 2 [file Table_2.DOCX]

Organizations records % of 3821

MONASH UNIV 82 2.146

UNIV MELBOURNE 57 1.492

UNIV SYDNEY 57 1.492

CHINA MED UNIV 56 1.466

SUN YAT SEN UNIV 55 1.439

CHINESE UNIV HONG KONG 52 1.361

JILIN UNIV 51 1.335

SHANDONG UNIV 47 1.230

CENT S UNIV 46 1.204

HARVARD UNIV 46 1.204

SOUTHERN MED UNIV 46 1.204

UNIV GRONINGEN 42 1.099

UNIV LOUISVILLE 41 1.073

VANDERBILT UNIV 41 1.073

WENZHOU MED UNIV 40 1.047

FUDAN UNIV 39 1.021

NANJING MED UNIV 38 0.995

SHANGHAI JIAO TONG UNIV 38 0.995

KANAZAWA MED UNIV 36 0.942

UNIV TORONTO 36 0.942

INSERM 34 0.890

MAYO CLIN 34 0.890

UNIV PITTSBURGH 34 0.890

UNIV COLL DUBLIN 33 0.864

UNIV UTAH 33 0.864

UNIV MICHIGAN 31 0.811

UNIV WASHINGTON 31 0.811

UNIV CALIF LOS ANGELES 30 0.785

HUAZHONG UNIV SCI TECHNOL 29 0.759

SICHUAN UNIV 29 0.759

UNIV PENN 29 0.759

BAKER IDI HEART DIABET INST 28 0.733

UNIV AUTONOMA MADRID 28 0.733

UNIV QUEENSLAND 28 0.733

KAOHSIUNG MED UNIV 27 0.707

MONASH MED CTR 27 0.707

UNIV CALIF SAN DIEGO 27 0.707

XUZHOU MED UNIV 27 0.707

ANHUI MED UNIV 26 0.680

UNIV HONG KONG 26 0.680

UNIV MED CTR UTRECHT 26 0.680

LEIDEN UNIV 25 0.654

UNIV TEXAS HLTH SCI CTR SAN ANTONIO 25 0.654

CAPITAL MED UNIV 24 0.628

UNIV AUSTRAL CHILE 24 0.628

NORTHWESTERN UNIV 23 0.602

SOUTHEAST UNIV 23 0.602

TOHOKU UNIV 23 0.602

EWHA WOMANS UNIV 22 0.576

UNIV HOSP 22 0.576

WUHAN UNIV 22 0.576

NANJING UNIV 21 0.550

UNIV MONTREAL 21 0.550

ZHENGZHOU UNIV 21 0.550

CATHOLIC UNIV KOREA 20 0.523

GUANGZHOU UNIV CHINESE MED 20 0.523

NIDDK 20 0.523

TONGJI UNIV 20 0.523

WASHINGTON UNIV 20 0.523

CENT SOUTH UNIV 19 0.497

GEORGETOWN UNIV 19 0.497

HANNOVER MED SCH 19 0.497

HARVARD MED SCH 19 0.497

OSAKA UNIV 19 0.497

ROYAL N SHORE HOSP 19 0.497

UNIV MISSISSIPPI 19 0.497

UNIV SAO PAULO 19 0.497

UNIV TOKYO 19 0.497

UNIV TURIN 19 0.497

CASE WESTERN RESERVE UNIV 18 0.471

CHANG GUNG UNIV 18 0.471

KYOTO UNIV 18 0.471

UNIV PADUA 18 0.471

ZHEJIANG UNIV 18 0.471

CHONGQING MED UNIV 17 0.445

DALIAN MED UNIV 17 0.445

HEBEI MED UNIV 17 0.445

JOHNS HOPKINS UNIV 17 0.445

NAGOYA UNIV 17 0.445

SEOUL NATL UNIV 17 0.445

SHANGHAI UNIV TRADIT CHINESE MED 17 0.445

TIANJIN MED UNIV 17 0.445

UNIV COPENHAGEN 17 0.445

UNIV TUBINGEN 17 0.445

YALE UNIV 17 0.445

CARDIFF UNIV 16 0.419

CHANG GUNG MEM HOSP 16 0.419

CHINESE ACAD MED SCI 16 0.419

INDIANA UNIV 16 0.419

MED COLL WISCONSIN 16 0.419

OKAYAMA UNIV 16 0.419

PEKING UNIV 16 0.419

UNIV ERLANGEN NURNBERG 16 0.419

UNIV MUNICH 16 0.419

UNIV OTTAWA 16 0.419

BRIGHAM WOMENS HOSP 15 0.393

CHARITE 15 0.393

GUIZHOU MED UNIV 15 0.393

HEIDELBERG UNIV 15 0.393

NANJING UNIV CHINESE MED 15 0.393

UNIV MINNESOTA 15 0.393

CITY HOPE NATL MED CTR 14 0.366

HARBIN MED UNIV 14 0.366

JINAN UNIV 14 0.366

KYUNGPOOK NATL UNIV 14 0.366

MASSACHUSETTS GEN HOSP 14 0.366

ST MICHAELS HOSP 14 0.366

UNIV AMSTERDAM 14 0.366

UNIV COLORADO 14 0.366

UNIV MIAMI 14 0.366

UNIV ZURICH 14 0.366

UNIV ZURICH HOSP 14 0.366

BETH ISRAEL DEACONESS MED CTR 13 0.340

NATL TAIWAN UNIV 13 0.340

SOUTH TEXAS VET HLTH CARE SYST 13 0.340

TAIPEI MED UNIV 13 0.340

THIRD MIL MED UNIV 13 0.340

UNIV CALIF SAN FRANCISCO 13 0.340

UNIV MISSOURI 13 0.340

BEIJING UNIV CHINESE MED 12 0.314

CHUNG SHAN MED UNIV 12 0.314

GEORGE WASHINGTON UNIV 12 0.314

HOP TENON 12 0.314

KAGAWA UNIV 12 0.314

KANAZAWA UNIV 12 0.314

KOREA UNIV 12 0.314

NATL TAIWAN UNIV HOSP 12 0.314

NIIGATA UNIV 12 0.314

QUEENS UNIV BELFAST 12 0.314

SHANXI MED UNIV 12 0.314

SHIGA UNIV MED SCI 12 0.314

TOKAI UNIV 12 0.314

TOKYO WOMENS MED UNIV 12 0.314

TULANE UNIV 12 0.314

XI AN JIAO TONG UNIV 12 0.314

YONSEI UNIV 12 0.314

ALBERT EINSTEIN COLL MED 11 0.288

BAYLOR COLL MED 11 0.288

CHINA JAPAN FRIENDSHIP HOSP 11 0.288

CHINESE ACAD SCI 11 0.288

COLUMBIA UNIV 11 0.288

EMORY UNIV 11 0.288

HALLYM UNIV 11 0.288

KEIO UNIV 11 0.288

KURUME UNIV 11 0.288

KYUNG HEE UNIV 11 0.288

MANSOURA UNIV 11 0.288

SECOND MIL MED UNIV 11 0.288

SOOCHOW UNIV 11 0.288

TEL AVIV UNIV 11 0.288

UCL 11 0.288

UNIV CALIF IRVINE 11 0.288

UNIV COLORADO DENVER 11 0.288

UNIV EDINBURGH 11 0.288

UNIV HLTH NETWORK 11 0.288

UNIV PARIS 05 11 0.288

UNIV PARIS 06 11 0.288

WONKWANG UNIV 11 0.288

XIAMEN UNIV 11 0.288

XINJIANG MED UNIV 11 0.288

AMER UNIV BEIRUT 10 0.262

BROWN UNIV 10 0.262

CHUNG HWA UNIV MED TECHNOL 10 0.262

KUMAMOTO UNIV 10 0.262

MED UNIV S CAROLINA 10 0.262

NANCHANG UNIV 10 0.262

OREGON HLTH SCI UNIV 10 0.262

ROYAL MELBOURNE HOSP 10 0.262

SEMMELWEIS UNIV 10 0.262

UNIV ALABAMA BIRMINGHAM 10 0.262

UNIV BIRMINGHAM 10 0.262

UNIV CHICAGO 10 0.262

UNIV GOTTINGEN 10 0.262

UNIV N CAROLINA 10 0.262

UNIV VIRGINIA 10 0.262

AARHUS UNIV 9 0.236

AUGUSTA UNIV 9 0.236

BOSTON UNIV 9 0.236

CHINA PHARMACEUT UNIV 9 0.236

CNR 9 0.236

FIBROGEN INC 9 0.236

FIRST HOSP JILIN UNIV 9 0.236

HOP NECKER ENFANTS MALAD 9 0.236

ICAHN SCH MED MT SINAI 9 0.236

INHA UNIV 9 0.236

JOSLIN DIABET CTR 9 0.236

KAOHSIUNG CHANG GUNG MEM HOSP 9 0.236

MT SINAI SCH MED 9 0.236

NANTONG UNIV 9 0.236

NATL CHENG KUNG UNIV 9 0.236

NYU 9 0.236

PEKING UNION MED COLL 9 0.236

RHEIN WESTFAL TH AACHEN 9 0.236

SOON CHUN HYANG UNIV 9 0.236

UNIV CALGARY 9 0.236

UNIV ILLINOIS 9 0.236

UNIV MANITOBA 9 0.236

UNIV MILAN 9 0.236

UNIV NACL AUTONOMA MEXICO 9 0.236

WAYNE STATE UNIV 9 0.236

WENZHOU MED COLL 9 0.236

ASTRAZENECA 8 0.209

CHARLIE NORWOOD VA MED CTR 8 0.209

CHI MEI MED CTR 8 0.209

CHINESE PEOPLES LIBERAT ARMY GEN HOSP 8 0.209

CHUNG SHAN MED UNIV HOSP 8 0.209

COMENIUS UNIV 8 0.209

DUKE UNIV 8 0.209

FUJIAN MED UNIV 8 0.209

HENAN UNIV 8 0.209

JIKEI UNIV 8 0.209

JUNTENDO UNIV 8 0.209

KINGS COLL LONDON 8 0.209

MAASTRICHT UNIV 8 0.209

MCGILL UNIV 8 0.209

MCMASTER UNIV 8 0.209

NATL YANG MING UNIV 8 0.209

OHIO STATE UNIV 8 0.209

RADBOUD UNIV NIJMEGEN 8 0.209

SOUTHWEST MED UNIV 8 0.209

UNIV ALABAMA 8 0.209

UNIV FED SAO PAULO 8 0.209

UNIV GLASGOW 8 0.209

UNIV MED CTR GRONINGEN 8 0.209

UNIV OKLAHOMA 8 0.209

UNIV OXFORD 8 0.209

UNIV WISCONSIN 8 0.209

VET AFFAIRS MED CTR 8 0.209

WAKE FOREST UNIV 8 0.209

WEILL CORNELL MED COLL 8 0.209

XUZHOU MED COLL 8 0.209

YOKOHAMA CITY UNIV 8 0.209

AARHUS UNIV HOSP 7 0.183

AKITA UNIV 7 0.183

ASTELLAS PHARMA INC 7 0.183

BAKER HEART DIABET INST 7 0.183

BAKER HEART RES INST 7 0.183

BAYLOR UNIV 7 0.183

BECKMAN RES INST CITY HOPE 7 0.183

CHONNAM NATL UNIV 7 0.183

COLL FRANCE 7 0.183

GEORGIA REGENTS UNIV 7 0.183

GUANGXI MED UNIV 7 0.183

GUANGZHOU MED UNIV 7 0.183

HARBOR UCLA MED CTR 7 0.183

HOSP ALEMAN 7 0.183

HOSP CIVILS LYON 7 0.183

INJE UNIV 7 0.183

INST NACL CARDIOL IGNACIO CHAVEZ 7 0.183

KAOHSIUNG MED UNIV HOSP 7 0.183

KEIMYUNG UNIV 7 0.183

KYUSHU UNIV 7 0.183

MAHIDOL UNIV 7 0.183

MED COLL GEORGIA 7 0.183

MED UNIV INNSBRUCK 7 0.183

MED UNIV VIENNA 7 0.183

MUDANJIANG MED UNIV 7 0.183

NIEHS 7 0.183

STENO DIABET CTR 7 0.183

THOMAS JEFFERSON UNIV 7 0.183

TORANOMON GEN HOSP 7 0.183

UNIV ALBERTA 7 0.183

UNIV BERN 7 0.183

UNIV CALIF DAVIS 7 0.183

UNIV HAMBURG 7 0.183

UNIV HOSP GENEVA 7 0.183

UNIV LORRAINE 7 0.183

UNIV PARIS DIDEROT 7 0.183

UNIV ROMA LA SAPIENZA 7 0.183

UNIV SHEFFIELD 7 0.183

UNIV TOULOUSE 3 7 0.183

UNIV WURZBURG 7 0.183

UPPSALA UNIV 7 0.183

ZAGAZIG UNIV 7 0.183

ASAHIKAWA MED UNIV 6 0.157

BAKER MED RES INST 6 0.157

BIRLA INST TECHNOL SCI PILANI 6 0.157

CATHOLIC UNIV LOUVAIN 6 0.157

CEDARS SINAI MED CTR 6 0.157

CHENGDU UNIV TRADIT CHINESE MED 6 0.157

CLEVELAND CLIN FDN 6 0.157

DOKUZ EYLUL UNIV 6 0.157

FOURTH MIL MED UNIV 6 0.157

GENZYME CORP 6 0.157

GUANGDONG PHARMACEUT UNIV 6 0.157

HAINAN UNIV 6 0.157

HARRY S TRUMAN MEM VET HOSP 6 0.157

HOSP CLIN SAN CARLOS 6 0.157

JADAVPUR UNIV 6 0.157

JILIN PROV PEOPLES HOSP 6 0.157

KAROLINSKA INST 6 0.157

KAWASAKI MED SCH 6 0.157

KING SAUD UNIV 6 0.157

KUNMING MED UNIV 6 0.157

LOUISIANA STATE UNIV 6 0.157

MARIO NEGRI INST PHARMACOL RES 6 0.157

MAX DELBRUCK CTR MOL MED 6 0.157

MINIST EDUC 6 0.157

NATL DEF MED COLL 6 0.157

NCI 6 0.157

NEWCASTLE UNIV 6 0.157

NINGBO UNIV 6 0.157

NOTTINGHAM TRENT UNIV 6 0.157

QINGDAO UNIV 6 0.157

RABIN MED CTR 6 0.157

ROYAL PRINCE ALFRED HOSP 6 0.157

SAITAMA MED UNIV 6 0.157

SHENZHEN UNIV 6 0.157

SHIMANE UNIV 6 0.157

SICHUAN ACAD MED SCI 6 0.157

SICHUAN PROV PEOPLES HOSP 6 0.157

SOUTH TEXAS VET HEALTHCARE SYST 6 0.157

SPANISH BIOMED RES CTR DIABET ASSOCIATED METAB 6 0.157

ST MARIANNA UNIV 6 0.157

ST VINCENTS HOSP 6 0.157

SUNGKYUNKWAN UNIV 6 0.157

UNIV ALCALA DE HENARES 6 0.157

UNIV ARKANSAS MED SCI 6 0.157

UNIV CINCINNATI 6 0.157

UNIV FLORIDA 6 0.157

UNIV HELSINKI 6 0.157

UNIV KENTUCKY 6 0.157

UNIV MARYLAND 6 0.157

UNIV MESSINA 6 0.157

UNIV PISA 6 0.157

UNIV POTSDAM 6 0.157

UNIV SASKATCHEWAN 6 0.157

UNIV SOUTHERN DENMARK 6 0.157

UNIV TENNESSEE 6 0.157

UNIV TOLEDO 6 0.157

UNIV TRIESTE 6 0.157

UNIV VERONA 6 0.157

UNIV WALES COLL MED 6 0.157

UNIV WARWICK 6 0.157

WANNAN MED COLL 6 0.157

ACAD MED CTR 5 0.131

ALBANY MED COLL 5 0.131

ARMY MED UNIV 5 0.131

BENGBU MED COLL 5 0.131

BIRLA INST TECHNOL SCI 5 0.131

BOEHRINGER INGELHEIM PHARMA GMBH CO KG 5 0.131

CHANGCHUN UNIV CHINESE MED 5 0.131

CHARITE UNIV MED BERLIN 5 0.131

CHILDRENS HOSP 5 0.131

CHILDRENS HOSP WESTMEAD 5 0.131

CHU POITIERS 5 0.131

CHUNG HWA COLL MED TECHNOL 5 0.131

DAINIPPON SUMITOMO PHARMA CO LTD 5 0.131

DUBLIN MOL MED CTR 5 0.131

EMERGENTEC BIODEV GMBH 5 0.131

FDN JIMENEZ DIAZ 5 0.131

FUKUOKA UNIV 5 0.131

GILEAD SCI INC 5 0.131

GOETHE UNIV FRANKFURT 5 0.131

GUANGDONG ACAD MED SCI 5 0.131

GUANGDONG MED UNIV 5 0.131

HONG KONG BAPTIST UNIV 5 0.131

IMPERIAL COLL LONDON 5 0.131

INDIANA UNIV SCH MED 5 0.131

INST SALUD CARLOS III 5 0.131

JACKSON LAB 5 0.131

JINING 1 PEOPLES HOSP 5 0.131

KAGOSHIMA UNIV 5 0.131

KLINIKUM UNIV MUNCHEN 5 0.131

LIVERPOOL JOHN MOORES UNIV 5 0.131

LUND UNIV 5 0.131

LUZHOU MED COLL 5 0.131

MED UNIV GRAZ 5 0.131

MINIST HLTH 5 0.131

MOREHOUSE SCH MED 5 0.131

NAGASAKI UNIV 5 0.131

NATL DEF MED CTR 5 0.131

NHLBI 5 0.131

NINGXIA MED UNIV 5 0.131

OSPED RIUNITI BERGAMO 5 0.131

SOONCHUNHYANG UNIV 5 0.131

STANFORD UNIV 5 0.131

TECH UNIV MUNICH 5 0.131

TIANJIN UNIV TRADIT CHINESE MED 5 0.131

TOKYO MED DENT UNIV 5 0.131

TROOPS 95935 UNIT 5 0.131

UNIV BARCELONA 5 0.131

UNIV BUENOS AIRES 5 0.131

UNIV CAMBRIDGE 5 0.131

UNIV COLOGNE 5 0.131

UNIV FED MINAS GERAIS 5 0.131

UNIV GENEVA 5 0.131

UNIV GEORGIA 5 0.131

UNIV HEIDELBERG 5 0.131

UNIV HOSP JENA 5 0.131

UNIV JENA 5 0.131

UNIV LYON 1 5 0.131

UNIV MANCHESTER 5 0.131

UNIV MILANO BICOCCA 5 0.131

UNIV NEW S WALES 5 0.131

UNIV OTAGO 5 0.131

UNIV REGENSBURG 5 0.131

UNIV SALAMANCA 5 0.131

UNIV SO CALIF 5 0.131

UNIV SZEGED 5 0.131

UNIV TEXAS SOUTHWESTERN MED CTR DALLAS 5 0.131

UNIV TSUKUBA 5 0.131

UNIV UTAH HLTH 5 0.131

UNIV WESTERN ONTARIO 5 0.131

UNIV YAMANASHI 5 0.131

VA MED CTR 5 0.131

VAMC 5 0.131

VIRGINIA COMMONWEALTH UNIV 5 0.131

YANBIAN UNIV 5 0.131

ALFRED HOSP 4 0.105

ALL INDIA INST MED SCI 4 0.105

ASAHIKAWA MED COLL 4 0.105

ASTRAZENECA R D 4 0.105

BELFAST CITY HOSP 4 0.105

BENXI CTR HOSP 4 0.105

BINZHOU MED UNIV 4 0.105

BOEHRINGER INGELHEIM GMBH CO KG 4 0.105

BOEHRINGER INGELHEIM PHARMACEUT INC 4 0.105

CHA UNIV 4 0.105

CHARLES UNIV PRAGUE 4 0.105

CHONBUK NATL UNIV 4 0.105

CHU RANGUEIL 4 0.105

COLUMBIA UNIV COLL PHYS SURG 4 0.105

DALIAN UNIV 4 0.105

DANIELLE ALBERTI MEM CTR DIABET COMPLICAT 4 0.105

DEPT VET AFFAIRS 4 0.105

ERASMUS MC 4 0.105

F HOFFMANN LA ROCHE LTD 4 0.105

FLORIDA STATE UNIV 4 0.105

FREE UNIV BERLIN 4 0.105

FUJITA HLTH UNIV 4 0.105

GACHON UNIV 4 0.105

GERMAN CANC RES CTR 4 0.105

GHENT UNIV HOSP 4 0.105

GUIZHOU PROV PEOPLES HOSP 4 0.105

HACETTEPE UNIV 4 0.105

HADASSAH UNIV HOSP 4 0.105

HAINAN GEN HOSP 4 0.105

HEBREW UNIV JERUSALEM 4 0.105

HIROSHIMA UNIV 4 0.105

HOKKAIDO UNIV 4 0.105

HOSP CLIN PORTO ALEGRE 4 0.105

HOSP UNIV BELLVITGE 4 0.105

HUNAN NORMAL UNIV 4 0.105

HUNAN UNIV CHINESE MED 4 0.105

HUNGKUANG UNIV 4 0.105

I SHOU UNIV 4 0.105

INST PASTEUR 4 0.105

IRCCS 4 0.105

IST SCI SAN RAFFAELE 4 0.105

JIAXING UNIV 4 0.105

JICHI MED UNIV 4 0.105

KANAZAWA UNIV HOSP 4 0.105

KOBE UNIV 4 0.105

KOREA INST ORIENTAL MED 4 0.105

LEICESTER GEN HOSP 4 0.105

LMU 4 0.105

LUDWIG MAXIMILIANS UNIV MUNCHEN 4 0.105

MACAU UNIV SCI TECHNOL 4 0.105

MACKAY MED COLL 4 0.105

MAGNA GRAECIA UNIV CATANZARO 4 0.105

MONTEFIORE MED CTR 4 0.105

MT SINAI HOSP 4 0.105

NATL CEREBRAL CARDIOVASC CTR 4 0.105

NATL CHUNG HSING UNIV 4 0.105

NATL LOCAL UNITED ENGN LAB DRUGGABIL NEW DRUG 4 0.105

NIDDKD 4 0.105

NIHON UNIV 4 0.105

NIIGATA UNIV PHARM APPL LIFE SCI 4 0.105

NINGBO UROL NEPHROL HOSP 4 0.105

NORTH CHINA UNIV SCI TECHNOL 4 0.105

NORTHWEST UNIV 4 0.105

OTTAWA HOSP 4 0.105

QUEEN ELIZABETH HOSP 4 0.105

RUSH UNIV 4 0.105

SHANDONG UNIV TRADIT CHINESE MED 4 0.105

SHINSHU UNIV 4 0.105

SHOWA UNIV 4 0.105

STENO DIABET CTR COPENHAGEN 4 0.105

TABRIZ UNIV MED SCI 4 0.105

TORANOMON HOSP KAJIGAYA 4 0.105

UNIV AARHUS 4 0.105

UNIV ANTWERP 4 0.105

UNIV AUTONOMA BARCELONA 4 0.105

UNIV BARI 4 0.105

UNIV BASEL 4 0.105

UNIV BRITISH COLUMBIA 4 0.105

UNIV CATTOLICA SACRO CUORE 4 0.105

UNIV DESARROLLO 4 0.105

UNIV ESTADO RIO DE JANEIRO 4 0.105

UNIV EXETER 4 0.105

UNIV FED RIO GRANDE DO SUL 4 0.105

UNIV GIESSEN 4 0.105

UNIV LEICESTER 4 0.105

UNIV LIVERPOOL 4 0.105

UNIV LONDON IMPERIAL COLL SCI TECHNOL MED 4 0.105

UNIV LYON 4 0.105

UNIV MALAYA 4 0.105

UNIV MARBURG 4 0.105

UNIV MED CTR GOTTINGEN 4 0.105

UNIV MUNSTER 4 0.105

UNIV OSLO 4 0.105

UNIV PAVIA 4 0.105

UNIV SOUTH CHINA 4 0.105

UNIV TEXAS MD ANDERSON CANC CTR 4 0.105

UNIV TEXAS SW MED CTR DALLAS 4 0.105

UNIV UTRECHT 4 0.105

UNIV VIRGINIA HLTH SYST 4 0.105

VA SAN DIEGO HEALTHCARE SYST 4 0.105

VET AFFAIRS SALT LAKE CITY HLTH CARE SYST 4 0.105

VICTOR BABES UNIV MED PHARM 4 0.105

VRIJE UNIV AMSTERDAM 4 0.105

WASHINGTON STATE UNIV 4 0.105

WESTERN UNIV 4 0.105

YANGZHOU UNIV 4 0.105

ZHEJIANG CHINESE MED UNIV 4 0.105

ZUNYI MED UNIV 4 0.105

AARHUS KOMMUNE HOSP 3 0.079

ABBVIE 3 0.079

ACAD ATHENS 3 0.079

AICHI MED UNIV 3 0.079

ANHUI UNIV 3 0.079

AOMORI UNIV HLTH WELF 3 0.079

AP HP 3 0.079

ATOM ENERGY COUNCIL 3 0.079

AUSTIN HLTH 3 0.079

AUSTIN HOSP 3 0.079

BANGLADESH AGR UNIV 3 0.079

BAR ILAN UNIV 3 0.079

BAXTER HEALTHCARE CORP 3 0.079

BEIHUA UNIV 3 0.079

BEIJING UNION MED COLL HOSP 3 0.079

CAIRO UNIV 3 0.079

CANGZHOU CENT HOSP 3 0.079

CATHOLIC UNIV 3 0.079

CHANG JUNG CHRISTIAN UNIV 3 0.079

CHANGHUA CHRISTIAN HOSP 3 0.079

CHANGSHA CENT HOSP 3 0.079

CHARLIE NORWOOD VET AFFAIRS MED CTR 3 0.079

CHIANG MAI UNIV 3 0.079

CHILDRENS HOSP LOS ANGELES 3 0.079

CHILDRENS NATL MED CTR 3 0.079

CHINA ACAD CHINESE MED SCI 3 0.079

CHINA MED UNIV HOSP 3 0.079

CHRU NANCY 3 0.079

CHU PURPAN 3 0.079

CHU TOURS 3 0.079

CHUNGNAM NATL UNIV 3 0.079

CLEVELAND CLIN 3 0.079

CTR ESTUDIOS CIENT 3 0.079

CTR HOSP LYON SUD 3 0.079

CTR RECH CORDELIERS 3 0.079

DAEGU GYEONGBUK MED INNOVAT FDN 3 0.079

DEAKIN UNIV 3 0.079

DICLE UNIV 3 0.079

DONGGUK UNIV 3 0.079

EGE UNIV 3 0.079

ELI LILLY CO 3 0.079

ERASMUS UNIV 3 0.079

ERNST MORITZ ARNDT UNIV GREIFSWALD 3 0.079

EULJI UNIV 3 0.079

FAC MED 3 0.079

FIRST PEOPLES HOSP FOSHAN 3 0.079

FLINDERS UNIV S AUSTRALIA 3 0.079

FUNDACIO PUIGVERT 3 0.079

GANNAN MED UNIV 3 0.079

GEORGIA GWINNETT COLL 3 0.079

GEORGIA HLTH SCI UNIV 3 0.079

GERMAN CTR CARDIOVASC RES DZHK 3 0.079

GIFU UNIV 3 0.079

GOETHE UNIV 3 0.079

GUANGDONG GEN HOSP 3 0.079

HAMAD MED CORP 3 0.079

HANYANG UNIV 3 0.079

HEART INST SPOKANE 3 0.079

HEBEI KEY LAB KIDNEY DIS 3 0.079

HELSINKI UNIV HOSP 3 0.079

HENAN UNIV TRADIT CHINESE MED 3 0.079

HENRY FORD HLTH SYST 3 0.079

HENRY FORD HOSP 3 0.079

HIROSHIMA UNIV HOSP 3 0.079

HKBU INST RES CONTINUING EDUC 3 0.079

HOFFMANN LA ROCHE INC 3 0.079

HOP BICETRE 3 0.079

HOP EUROPEEN GEORGES POMPIDOU 3 0.079

HOP ST ANTOINE 3 0.079

HOSP 12 OCTUBRE 3 0.079

HOSP CHENGDU UNIV TRADIT CHINESE MED 3 0.079

HOSP GEN UNIV GREGORIO MARANON 3 0.079

HOSP MAR 3 0.079

HOSP SICK CHILDREN 3 0.079

HOSP UNIV LA PAZ 3 0.079

HOSP UNIV SALAMANCA 3 0.079

HUBEI UNIV MED 3 0.079

IDIPAZ 3 0.079

IIS FDN JIMENEZ DIAZ 3 0.079

IMPERIAL COLL 3 0.079

IMSS 3 0.079

INNER MONGOLIA MED UNIV 3 0.079

INST CLIN EXPT MED 3 0.079

INST NACL CIENCIAS MED NUTR SALVADOR ZUBIRAN 3 0.079

INST POLITECN NACL 3 0.079

INST RECERCA BIOMED LLEIDA IRBLLEIDA 3 0.079

INTERCEPT PHARMACEUT 3 0.079

IRAN UNIV MED SCI 3 0.079

IRCCS IST RIC FARMACOL MARIO NEGRI 3 0.079

IRONWOOD PHARMACEUT 3 0.079

ISTANBUL UNIV 3 0.079

JAGIELLONIAN UNIV 3 0.079

JAMES COOK UNIV 3 0.079

JCHO TOKYO TAKANAWA HOSP 3 0.079

JDRF DANIELLE ALBERTI MEM CTR DIABET COMPLICAT 3 0.079

JENA UNIV HOSP 3 0.079

JIANGNAN UNIV 3 0.079

JIANGSU NORMAL UNIV 3 0.079

JIANGSU UNIV 3 0.079

KATHOLIEKE UNIV LEUVEN 3 0.079

KING ABDULAZIZ UNIV 3 0.079

KING MED DIAGNOST CTR 3 0.079

KYOTO PHARMACEUT UNIV 3 0.079

KYOTO PREFECTURAL UNIV MED 3 0.079

MATER MED RES INST 3 0.079

MED UNIV GDANSK 3 0.079

MED UNIV WARSAW 3 0.079

MERCK CO INC 3 0.079

MONASH HLTH 3 0.079

MT SINAI MED CTR 3 0.079

NAGOYA UNIV HOSP 3 0.079

NANFANG HOSP 3 0.079

NASHVILLE VET AFFAIRS HOSP 3 0.079

NATL INST PHARMACEUT EDUC RES 3 0.079

NATL KAPODISTRIAN UNIV ATHENS 3 0.079

NATL UNIV IRELAND UNIV COLL DUBLIN 3 0.079

NIH 3 0.079

NO GEN HOSP 3 0.079

NORTHEASTERN UNIV 3 0.079

OSAKA CITY GEN HOSP 3 0.079

OSAKA CITY UNIV 3 0.079

PANJAB UNIV 3 0.079

PANYU CENT HOSP 3 0.079

PARIS DESCARTES UNIV 3 0.079

PENNINGTON BIOMED RES CTR 3 0.079

PEOPLES HOSP JIANGYIN 3 0.079

PEOPLES HOSP LIAONING PROV 3 0.079

RALPH H JOHNSON VET ADM MED CTR 3 0.079

RED INVEST RENAL REDINREN 3 0.079

REDINREN 3 0.079

RHODE ISL HOSP 3 0.079

RICHARD L ROUDEBUSH VET AFFAIRS MED CTR 3 0.079

RIGSHOSP 3 0.079

ROSALIND FRANKLIN UNIV MED SCI 3 0.079

ROYAL NORTH SHORE HOSP 3 0.079

ROYAL VET COLL 3 0.079

RUSSIAN ACAD SCI 3 0.079

SANJAY GANDHI POSTGRAD INST MED SCI 3 0.079

SCH MED 3 0.079

SE UNIV 3 0.079

SHAANXI UNIV TECHNOL 3 0.079

SHANDONG FIRST MED UNIV 3 0.079

SHENYANG PHARMACEUT UNIV 3 0.079

SHONAN KAMAKURA GEN HOSP 3 0.079

SO MED UNIV 3 0.079

ST VINCENTS UNIV HOSP 3 0.079

SUNY STONY BROOK 3 0.079

SUZHONG PHARMACEUT GRP CO LTD 3 0.079

SVKMS NMIMS 3 0.079

TAIPEI VET GEN HOSP 3 0.079

TECHNION ISRAEL INST TECHNOL 3 0.079

TEXAS A M UNIV 3 0.079

TIANJIN INST ENDOCRINOL 3 0.079

TOKYO METROPOLITAN INST GERONTOL 3 0.079

TORONTO GEN HOSP 3 0.079

TRISERV GEN HOSP 3 0.079

UAM 3 0.079

UNITED ARAB EMIRATES UNIV 3 0.079

UNIV AACHEN 3 0.079

UNIV CALCUTTA 3 0.079

UNIV CHINESE ACAD SCI 3 0.079

UNIV CLAUDE BERNARD LYON 1 3 0.079

UNIV COIMBRA 3 0.079

UNIV COMPLUTENSE MADRID 3 0.079

UNIV COPENHAGEN HOSP 3 0.079

UNIV DUISBURG ESSEN 3 0.079

UNIV DUSSELDORF 3 0.079

UNIV FLORENCE 3 0.079

UNIV FREIBURG 3 0.079

UNIV IOWA 3 0.079

UNIV KANSAS 3 0.079

UNIV LA REUNION 3 0.079

UNIV LAUSANNE 3 0.079

UNIV LUND HOSP 3 0.079

UNIV MAASTRICHT 3 0.079

UNIV MASSACHUSETTS 3 0.079

UNIV NACL CUYO 3 0.079

UNIV NEW MEXICO 3 0.079

UNIV NEWCASTLE 3 0.079

UNIV OULU 3 0.079

UNIV POITIERS 3 0.079

UNIV PORTO 3 0.079

UNIV RYUKYUS 3 0.079

UNIV S CAROLINA 3 0.079

UNIV SHERBROOKE 3 0.079

UNIV SO QUEENSLAND 3 0.079

UNIV TABUK 3 0.079

UNIV TEXAS 3 0.079

UNIV TEXAS HLTH 3 0.079

UNIV TEXAS HLTH SCI CTR HOUSTON 3 0.079

UNIV TOKUSHIMA 3 0.079

UNIV TOULOUSE III PAUL SABATIER 3 0.079

US DEPT VET AFFAIRS 3 0.079

UT HLTH SAN ANTONIO 3 0.079

UTRECHT INST PHARMACEUT SCI 3 0.079

WENZHOU UNIV 3 0.079

WESTMEAD HOSP 3 0.079

WROCLAW MED UNIV 3 0.079

XUHUI DIST CENT HOSP SHANGHAI 3 0.079

XUZHOU CENT HOSP 3 0.079

YEUNGNAM UNIV 3 0.079

ZHEJIANG INTEGRATED WESTERN MED HOSP 3 0.079

ACAD SINICA 2 0.052

ADDENBROOKES HOSP 2 0.052

AHVAZ JUNDISHAPUR UNIV MED SCI 2 0.052

AL AZHAR UNIV 2 0.052

ALBANY MED CTR 2 0.052

ALBERT SZENT GYORGYI MED UNIV 2 0.052

ALEXANDRIA UNIV 2 0.052

ANHUI INST INNOVAT DRUGS 2 0.052

ANHUI UNIV TRADIT CHINESE MED 2 0.052

ANNAMALAI UNIV 2 0.052

ANQING MED PHARMACEUT COLL 2 0.052

APTABIO THERAPEUT INC 2 0.052

ARISTOTLE UNIV THESSALONIKI 2 0.052

ASIA UNIV 2 0.052

ASSIUT UNIV 2 0.052

ASTON UNIV 2 0.052

ATLANTA VET AFFAIRS MED CTR 2 0.052

BADR UNIV CAIRO 2 0.052

BAKER IDI HEART DIABET RES INST 2 0.052

BAKIRKOY DR SADI KONUK TRAINING RES HOSP 2 0.052

BAOJI CENT HOSP 2 0.052

BASKENT UNIV 2 0.052

BAYER AG 2 0.052

BEIJING KEY LAB TCM COLLATERAL DIS THEORY RES 2 0.052

BEIJING MED UNIV 2 0.052

BEN GURION UNIV NEGEV 2 0.052

BETHUNE INT PEACE HOSP 2 0.052

BIOGAZELLE 2 0.052

BIOGEN 2 0.052

BIRJAND UNIV MED SCI 2 0.052

BIRMINGHAM HEARTLANDS HOSP 2 0.052

BOEHRINGER INGELHEIM ANIM HLTH GMBH 2 0.052

BOMBAY COLL PHARM 2 0.052

BRITISH UNIV EGYPT 2 0.052

CADILA HEALTHCARE LTD 2 0.052

CALTECH 2 0.052

CARDIFF MEDICTR 2 0.052

CATHAY GEN HOSP 2 0.052

CENT ARKANSAS VET HEALTHCARE SYST 2 0.052

CENT QUEENSLAND UNIV 2 0.052

CHANG GUNG INST TECHNOL 2 0.052

CHANGDE VOCAT TECH COLL 2 0.052

CHARLES DREW UNIV 2 0.052

CHARLES R DREW UNIV MED SCI 2 0.052

CHENGDU FIFTH PEOPLES HOSP 2 0.052

CHENGDU MIL GEN HOSP 2 0.052

CHIBA UNIV 2 0.052

CHU BORDEAUX 2 0.052

CHU BREST 2 0.052

CHU CAEN 2 0.052

CHU LILLE 2 0.052

CHU NANTES 2 0.052

CHU REIMS 2 0.052

CHU RENNES 2 0.052

CHU VAUDOIS 2 0.052

CHUGAI PHARMACEUT CO LTD 2 0.052

CHULALONGKORN UNIV 2 0.052

CHUNG ANG UNIV 2 0.052

CHUNGNAM NATL UNIV HOSP 2 0.052

CIMA UNIV NAVARRA 2 0.052

CINVESTAV IPN 2 0.052

CLEA JAPAN INC 2 0.052

CLIN UNIV NAVARRA 2 0.052

CMIC CO LTD 2 0.052

CTR DIS CONTROL PREVENT CDC 2 0.052

CYCLERION THERAPEUT 2 0.052

DAEGU HAANY UNIV 2 0.052

DAEJEON ST MARY HOSP 2 0.052

DANA FARBER CANC INST 2 0.052

DAQING OILFIELD GEN HOSP 2 0.052

DEPT INTERNAL MED 2 0.052

DREXEL UNIV 2 0.052

DZHK GERMAN CTR CARDIOVASC RES 2 0.052

EXPT CLIN RES CTR 2 0.052

FAC MED TOULOUSE 2 0.052

FDN PUIGVERT 2 0.052

FDN RENAL INIGO ALVAREZ DE TOLEDO IRSIN 2 0.052

FIRST PEOPLES HOSP YUNNAN PROV 2 0.052

FOOYIN UNIV 2 0.052

FRAMINGHAM HEART DIS EPIDEMIOL STUDY 2 0.052

FRIEDRICH ALEXANDER UNIV ERLANGEN NURNBERG 2 0.052

FU JEN CATHOLIC UNIV 2 0.052

GANSU PROV PEOPLES HOSP 2 0.052

GEISINGER MED CLIN 2 0.052

GENET MODELS INC 2 0.052

GILEAD SCI 2 0.052

GRAZ UNIV 2 0.052

GUANGDONG MED COLL 2 0.052

GUANGDONG PROV HOSP CHINESE MED 2 0.052

GUANGDONG PROV KEY LAB NEW DRUG DESIGN EVALUAT 2 0.052

GUANGZHOU RED CROSS HOSP 2 0.052

GUILIN MED UNIV 2 0.052

GULHANE MIL MED ACAD 2 0.052

GUNMA UNIV 2 0.052

GUYS HOSP 2 0.052

GYEONGSANG NATL UNIV 2 0.052

HADASSAH HEBREW UNIV 2 0.052

HAMAMATSU UNIV SCH MED 2 0.052

HANGZHOU NORMAL UNIV 2 0.052

HARBIN MED COLL 2 0.052

HARBOR UCLA 2 0.052

HARRY S TRUMAN VA MED CTR 2 0.052

HARVARD INST MED 2 0.052

HARVARD MIT DIV HLTH SCI TECHNOL 2 0.052

HARVARD STEM CELL INST 2 0.052

HARVARD TH CHAN SCH PUBL HLTH 2 0.052

HEART RES INST 2 0.052

HEILONGJIANG PROV HOSP 2 0.052

HENAN MED COLL 2 0.052

HENAN UNIV CHINESE MED 2 0.052

HENAN UNIV SCI TECHNOL 2 0.052

HENNEPIN CTY MED CTR 2 0.052

HERLEV HOSP 2 0.052

HITIT UNIV 2 0.052

HONG KONG SANAT HOSP 2 0.052

HOP BICHAT CLAUDE BERNARD 2 0.052

HOP COCHIN 2 0.052

HOP LARIBOISIERE 2 0.052

HOP MAISON NEUVE ROSEMONT 2 0.052

HOP UNIV STRASBOURG 2 0.052

HOPE HEART INST 2 0.052

HOSP ARNAU VILANOVA 2 0.052

HOSP CLIN BARCELONA 2 0.052

HOSP DEL MAR 2 0.052

HOSP PRINCIPE ASTURIAS 2 0.052

HOSP ST GEORG 2 0.052

HOSP UNIV CENT ASTURIAS 2 0.052

HOSP UNIV FDN ALCORCON 2 0.052

HOSP UNIV LA PRINCESA 2 0.052

HOSP UNIV PENN 2 0.052

HOSP UNIV VALL DHEBRON 2 0.052

HUAIAN SECOND PEOPLES HOSP 2 0.052

HUBEI UNIV SCI TECHNOL 2 0.052

HUDSON INST MED RES 2 0.052

HUMBOLDT UNIV 2 0.052

HUNGARIAN ACAD SCI 2 0.052

HYOGO COLL MED 2 0.052

INDIAN INST CHEM BIOL 2 0.052

INNER MONGOLIA UNIV 2 0.052

INNER MONGOLIA UNIV NATIONALITIES 2 0.052

INNSBRUCK MED UNIV 2 0.052

INST CARDIOVASC METAB DIS 2 0.052

INST INVEST BIOMED SALAMANCA IBSAL 2 0.052

INST ISRAELITA ENSINO PESQUISA ALBERT EINSTEIN 2 0.052

INST MEXICANO SEGURO SOCIAL 2 0.052

INST PREVENT CLIN MED 2 0.052

INST RECH CLIN MONTREAL 2 0.052

INST SYST BIOL 2 0.052

INST TRANSPLANTAT 2 0.052

IOWA STATE UNIV 2 0.052

IRSIN 2 0.052

ISCIII 2 0.052

IST GIANNINA GASLINI 2 0.052

IWATE UNIV 2 0.052

JANSSEN RES DEV LLC 2 0.052

JAPAN SOC PROMOT SCI 2 0.052

JIAOTONG UNIV 2 0.052

JINING MED UNIV 2 0.052

JOHANNES GUTENBERG UNIV MAINZ 2 0.052

JOHNS HOPKINS BLOOMBERG SCH PUBL HLTH 2 0.052

KAGAWA MED UNIV 2 0.052

KAISER PERMANENTE HAWAII 2 0.052

KAISER PERMANENTE NORTHWEST 2 0.052

KALLYOPE 2 0.052

KAOHISUNG CHANG GUNG MEM HOSP 2 0.052

KAOHSIUNG MED COLL 2 0.052

KAROLINSKA UNIV HOSP 2 0.052

KASHAN UNIV MED SCI 2 0.052

KERMANSHAH UNIV MED SCI 2 0.052

KING FAISAL SPECIALIST HOSP RES CTR 2 0.052

KING KHALID UNIV 2 0.052

KITASATO UNIV 2 0.052

KLINIKUM JW GOETHE UNIV FRANKFURT MAIN 2 0.052

KLINIKUM NURNBERG 2 0.052

KNOLL AG 2 0.052

KOC UNIV 2 0.052

KOCHI UNIV 2 0.052

KYOTO WOMENS UNIV 2 0.052

LANZHOU UNIV 2 0.052

LIAOCHENG UNIV 2 0.052

LIAONING MED UNIV 2 0.052

LINCOLN UNIV 2 0.052

LINKOPING UNIV 2 0.052

LINYI PEOPLES HOSP 2 0.052

LIVERPOOL HOSP 2 0.052

LORRAINE UNIV 2 0.052

MADRAS DIABET RES FDN 2 0.052

MADURAI KAMARAJ UNIV 2 0.052

MANCHESTER ROYAL INFIRM 2 0.052

MANCHESTER UNIV NHS FDN TRUST 2 0.052

MARMARA UNIV 2 0.052

MATER MISERICORDIAE UNIV HOSP 2 0.052

MED CTR 2 0.052

MED UNIV LODZ 2 0.052

MEDIPOL UNIV 2 0.052

MEIJI GAKUIN UNIV 2 0.052

MENZIES SCH HLTH RES 2 0.052

MERCK RES LABS 2 0.052

METHODIST HOSP 2 0.052

METROHLTH MED CTR 2 0.052

MICHIGAN STATE UNIV 2 0.052

MIE UNIV 2 0.052

MING DAO UNIV 2 0.052

MISR SCI TECHNOL UNIV 2 0.052

MITSUBISHI TANABE PHARMA CORP 2 0.052

MONTANA STATE UNIV 2 0.052

MOSA DIAGNOST GMBH 2 0.052

NAGOYA CITY UNIV 2 0.052

NANJING AGR UNIV 2 0.052

NANJING UNIV TCM 2 0.052

NATL CHENG KUNG UNIV HOSP 2 0.052

NATL CTR GLOBAL HLTH MED 2 0.052

NATL HOSP ORG 2 0.052

NATL INST CARDIOL IGNACIO CHAVEZ 2 0.052

NATL INST CHEM PHARMACEUT RES DEV 2 0.052

NATL TAIWAN OCEAN UNIV 2 0.052

NATL UNIV SINGAPORE 2 0.052

NEI 2 0.052

NETHERLANDS HEART INST 2 0.052

NEW YORK MED COLL 2 0.052

NEWCASTLE UPON TYNE HOSP NHS FDN TRUST 2 0.052

NIA 2 0.052

NIAAA 2 0.052

NICHHD 2 0.052

NIPPON BOEHRINGER INGELHEIM CO LTD 2 0.052

NO GEN HOSP TRUST 2 0.052

NORD BIOSCI AS 2 0.052

NORTH SOUTH UNIV 2 0.052

NORTHSHORE UNIV HEALTHSYST 2 0.052

NOVO NORDISK 2 0.052

OCEAN UNIV CHINA 2 0.052

ODENSE UNIV HOSP 2 0.052

OITA UNIV 2 0.052

OKAYAMA UNIV HOSP 2 0.052

OKINAKA MEM INST MED RES 2 0.052

ONO PHARMACEUT CO LTD 2 0.052

OSAKA PREFECTURE UNIV 2 0.052

OSPED SAN RAFFAELE 2 0.052

OTSUKA PHARMACEUT CO LTD 2 0.052

OTTAWA HOSP RES INST 2 0.052

OZGENE PTY LTD 2 0.052

PENINSULA MED SCH 2 0.052

PENN STATE COLL MED 2 0.052

PENN STATE UNIV 2 0.052

PFIZER 2 0.052

PFIZER GLOBAL RES DEV 2 0.052

PFIZER WORLDWIDE RES DEV 2 0.052

PHARMAFOOD INST 2 0.052

PHRAMONGKUTKLAO HOSP COLL MED 2 0.052

PONTIFICIA UNIV CATOLICA CHILE 2 0.052

PONTIFICIA UNIV CATOLICA PARANA 2 0.052

PONTIFICIA UNIV CATOLICA VALPARAISO 2 0.052

PORTLAND VA MED CTR 2 0.052

POSTGRAD INST MED EDUC RES 2 0.052

PRINCE HENRYS INST MED RES 2 0.052

PRINCESS ALEXANDRA HOSP 2 0.052

PROVIDENCE HLTH CARE 2 0.052

PUSAN NATL UNIV 2 0.052

PUSAN NATL UNIV HOSP 2 0.052

QATAR UNIV 2 0.052

RMIT UNIV 2 0.052

ROCKEFELLER UNIV 2 0.052

ROUDEBUSH VA MED CTR 2 0.052

ROYAL CHILDRENS HOSP 2 0.052

ROYAL DARWIN HOSP 2 0.052

RUPRECHT KARLS UNIV HEIDELBERG 2 0.052

RWTH AACHEN UNIV HOSP 2 0.052

S TEXAS VET HLTH CARE SYST 2 0.052

SAARLAND UNIV 2 0.052

SACRED HEART MED CTR 2 0.052

SAGA UNIV 2 0.052

SAISEIKAI SUITA HOSP 2 0.052

SALT LAKE VET AFFAIRS MED CTR 2 0.052

SAN BORTOLO HOSP 2 0.052

SAN FRANCISCO VA MED CTR 2 0.052

SAN GERARDO HOSP 2 0.052

SANDOZ PHARMA LTD 2 0.052

SAPIENZA UNIV ROMA 2 0.052

SEACROFT HOSP 2 0.052

SEATTLE CHILDRENS HOSP 2 0.052

SECHENOV FIRST MOSCOW STATE MED UNIV 2 0.052

SECOND HOSP JILIN UNIV 2 0.052

SEOUL NATL UNIV HOSP 2 0.052

SHAANXI PROV PEOPLES HOSP 2 0.052

SHAANXI TRADIT CHINESE MED HOSP 2 0.052

SHANDONG PROV QIANFOSHAN HOSP 2 0.052

SHEFFIELD CHILDRENS NHS FDN TRUST 2 0.052

SHENYANG 8 HOSP 2 0.052

SHENZHEN CHILDRENS HOSP 2 0.052

SHENZHEN PEOPLES HOSP 2 0.052

SHIRAZ UNIV MED SCI 2 0.052

SLOVAK ACAD SCI 2 0.052

ST HELIER HOSP 2 0.052

ST JAMES UNIV HOSP 2 0.052

ST JOHN HOSP MED CTR 2 0.052

ST JOSEPHS HOSP 2 0.052

STATE KEY LAB LONG ACTING TARGETING DRUG DELIVE 2 0.052

STATE KEY LAB MED GENET CHINA 2 0.052

SUEZ CANAL UNIV 2 0.052

SULTAN QABOOS UNIV 2 0.052

TAIAN CITY CENT HOSP 2 0.052

TAICHUNG VET GEN HOSP 2 0.052

TAIPEI CITY HOSP 2 0.052

TAIPEI MED UNIV HOSP 2 0.052

TAJEN UNIV 2 0.052

TANTA UNIV 2 0.052

TECH UNIV DENMARK 2 0.052

TECH UNIV DRESDEN 2 0.052

TEMPLE UNIV 2 0.052

TENON HOSP 2 0.052

TEXAS CHILDRENS HOSP 2 0.052

TEXAS TECH UNIV 2 0.052

THIRD HOSP NANCHANG 2 0.052

TOKYO MED UNIV 2 0.052

TORAY INDUSTRIES LTD 2 0.052

TORBAY HOSP 2 0.052

TOTTORI UNIV 2 0.052

TOYAMA UNIV 2 0.052

TRADIT CHINESE MED HOSP TONGZHOU DIST 2 0.052

TRINITY COLL DUBLIN 2 0.052

TURGUT OZAL UNIV 2 0.052

UCL ROYAL FREE UNIV COLL MED SCH 2 0.052

UFR ST ANTOINE 2 0.052

ULB 2 0.052

UNIV ARIZONA 2 0.052

UNIV ATHENS 2 0.052

UNIV AUCKLAND 2 0.052

UNIV AUTONOMA CHILE 2 0.052

UNIV BARI ALDO MORO 2 0.052

UNIV BERGEN 2 0.052

UNIV BRESCIA 2 0.052

UNIV BRISTOL 2 0.052

UNIV CAMPANIA LUIGI VANVITELLI 2 0.052

UNIV CHICAGO MED 2 0.052

UNIV CONNECTICUT 2 0.052

UNIV CORDOBA 2 0.052

UNIV DUNDEE 2 0.052

UNIV ELECT SCI TECHNOL CHINA 2 0.052

UNIV FED ALAGOAS 2 0.052

UNIV FED SAO PAULO UNIFESP 2 0.052

UNIV FRANKFURT KLINIKUM 2 0.052

UNIV FUKUI 2 0.052

UNIV GOTHENBURG 2 0.052

UNIV GRENOBLE 1 2 0.052

UNIV GRONINGEN UNIV MED CTR GRONINGEN 2 0.052

UNIV HALLE WITTENBERG 2 0.052

UNIV HOSP CLEVELAND 2 0.052

UNIV HOSP COLOGNE 2 0.052

UNIV HOSP FREIBURG 2 0.052

UNIV HOSP GOTTINGEN 2 0.052

UNIV HOSP LEICESTER 2 0.052

UNIV HOSP LEUVEN 2 0.052

UNIV HOSP MUNSTER 2 0.052

UNIV HOUSTON 2 0.052

UNIV HULL 2 0.052

UNIV HYOGO 2 0.052

UNIV INNSBRUCK 2 0.052

UNIV IOANNINA 2 0.052

UNIV KEBANGSAAN MALAYSIA 2 0.052

UNIV KIEL 2 0.052

UNIV KLINIKUM SCHLESWIG HOLSTEIN 2 0.052

UNIV LAVAL 2 0.052

UNIV LEIDEN HOSP 2 0.052

UNIV LEUVEN 2 0.052

UNIV LILLE 2 0.052

UNIV LINCOLN 2 0.052

UNIV LJUBLJANA 2 0.052

UNIV LONDON 2 0.052

UNIV LYON 1 LAENNEC 2 0.052

UNIV MED CTR 2 0.052

UNIV MED CTR HAMBURG EPPENDORF 2 0.052

UNIV MED GREIFSWALD 2 0.052

UNIV MED PHARM CAROL DAVILA 2 0.052

UNIV MED PHARM CRAIOVA 2 0.052

UNIV NAPLES 2 2 0.052

UNIV NEBRASKA MED CTR 2 0.052

UNIV NORTH TEXAS HLTH SCI CTR 2 0.052

UNIV NOTTINGHAM 2 0.052

UNIV NSW 2 0.052

UNIV PARIS SUD 2 0.052

UNIV PATRAS 2 0.052

UNIV S ALABAMA 2 0.052

UNIV SAARLAND 2 0.052

UNIV SAINS MALAYSIA 2 0.052

UNIV SALERNO 2 0.052

UNIV SEVILLE 2 0.052

UNIV SHIZUOKA 2 0.052

UNIV SOUTHERN CALIF 2 0.052

UNIV SOUTHERN QUEENSLAND 2 0.052

UNIV SPLIT 2 0.052

UNIV TECHNOL SYDNEY 2 0.052

UNIV TEXAS HLTH SCI CTR 2 0.052

UNIV TEXAS HOUSTON 2 0.052

UNIV TEXAS MED BRANCH 2 0.052

UNIV TOULOUSE 2 0.052

UNIV TOURS 2 0.052

UNIV TURKU 2 0.052

UNIV VERMONT 2 0.052

UNIV VIGO 2 0.052

UNIV WALES COLL CARDIFF 2 0.052

UNIV WALES HOSP 2 0.052

UNIV WESTERN AUSTRALIA 2 0.052

VA PITTSBURGH HEALTHCARE SYST 2 0.052

VALL DHEBRON RES INST VHIR 2 0.052

VANDERBILT UNIV SCH MED 2 0.052

VET ADM SAN DIEGO HEALTHCARE SYST 2 0.052

VET AFFAIRS CONNECTICUT HEALTHCARE SYST 2 0.052

VET AFFAIRS SAN DIEGO HEALTHCARE SYST 2 0.052

VICTORIA UNIV 2 0.052

WAKAYAMA MED UNIV 2 0.052

WELLINGTON SCH MED 2 0.052

WUHAN THIRD HOSP 2 0.052

WUXI HIGHER HLTH VOCAT TECHNOL SCH 2 0.052

XIANGYA HOSP 2 0.052

YANBIAN UNIV HOSP 2 0.052

YANGTZE UNIV 2 0.052

YANTAI YUHUANGDING HOSP 2 0.052

YEDITEPE UNIV 2 0.052

ZHEJIANG HOSP 2 0.052

ZHEJIANG OCEAN UNIV 2 0.052

105TH HOSP PLA 1 0.026

150TH HOSP PLA 1 0.026

2ND HOSP CHANGCHUN 1 0.026

303TH HOSP PLA 1 0.026

323 HOSP PLA 1 0.026

3PM WUHAN 1 0.026

463RD HOSP PLA 1 0.026

A MANZONI HOSP 1 0.026

AACHEN UNIV HOSP 1 0.026

AALBORG UNIV 1 0.026

AALTO UNIV 1 0.026

ABANT IZZET BAYSAL UNIV 1 0.026

ABBOTT LABS 1 0.026

ABBOTT PROD GMBH 1 0.026

ABBVIE BIOPHARMACEUT 1 0.026

ABU DHABI HLTH SERV CORP SEHA 1 0.026

ACAD HOSP ST JAN 1 0.026

ACAD MED CTR AMSTERDAM 1 0.026

ACAD SCI 1 0.026

ACIREALE HOSP 1 0.026

ADA UNIV 1 0.026

ADIS INT LTD 1 0.026

AFFILIATED HOSP BEIHUA UNIV 1 0.026

AGENCIA SANITARIA COSTA DEL SOL MARBELLA 1 0.026

AGRI STATE HOSP 1 0.026

AICHI GAKUIN UNIV 1 0.026

AIMST UNIV 1 0.026

AINTREE UNIV HOSP NHS FDN TRUST 1 0.026

AIR FORCE MED UNIV 1 0.026

AJA UNIV MED SCI 1 0.026

AKEBONO CLIN 1 0.026

AKER ULLEVAL DIABET RES CTR 1 0.026

AKITA MED CTR 1 0.026

AKITA UNIV HOSP 1 0.026

AL WAILI FDN SCI 1 0.026

ALAGAPPA UNIV 1 0.026

ALAMEDA HLTH SYST 1 0.026

ALAMOGORDO PRIMATE FACIL 1 0.026

ALBERT LUDWIGS UNIV 1 0.026

ALBERT LUDWIGS UNIV FREIBURG 1 0.026

ALBERTA CHILDRENS PROV GEN HOSP 1 0.026

ALBERTA KIDNEY DIS NETWORK 1 0.026

ALDER HEY CHILDRENS NHS FDN TRUST 1 0.026

ALDO MORO UNIV BARI 1 0.026

ALFAISAL UNIV 1 0.026

ALFRED HLTH 1 0.026

ALFRED MED RES EDUC PRECINCT 1 0.026

ALMA MATER STUDIORUM UNIV BOLOGNA 1 0.026

ALPHA MED AS 1 0.026

ALSTROM SYNDROME INT 1 0.026

ALSTROM SYNDROME UK 1 0.026

ALTEON INC 1 0.026

ALTON OCHSNER MED FDN OCHSNER CLIN 1 0.026

ALTSHULER CTR EDUC RES METROCARE SERV 1 0.026

AMBULATORY INTERNAL MED TEACHING CLIN 1 0.026

AMD 1 0.026

AMITY UNIV 1 0.026

AMSTERDAM UNIV 1 0.026

ANAT PATOL ASOU MOLINETTE 1 0.026

ANDHRA UNIV 1 0.026

ANDONG NATL UNIV 1 0.026

ANHUI ACAD CHINESE MED 1 0.026

ANHUI KEY LAB ECOENGN BIOTECHNOL 1 0.026

ANHUI KEY LAB ECOL ENGN BIOTECHNOL 1 0.026

ANHUI MED COLL 1 0.026

ANHUI PROV CANC HOSP 1 0.026

ANHUI PROV KEY LAB CHINESE MED FORMULA 1 0.026

ANHUI PROV KEY LAB CHINESE MED RES DEV 1 0.026

ANHUI SCI TECHNOL UNIV 1 0.026

ANHUI UNIV CHINESE MED 1 0.026

ANHUI UNIV SCI TECHNOL 1 0.026

ANIM MONITORING INST 1 0.026

ANKANG HOSP TRADIT CHINESE MED 1 0.026

ANKARA PEDIAT HEMATOL ONCOL EDUC TRAINING HOSP 1 0.026

ANKARA PEDIAT HLTH HEMATOL ONCOL HOSP 1 0.026

ANKARA UNIV 1 0.026

ANN ROBERT H LURIE CHILDRENS HOSP CHICAGO 1 0.026

AOKI INTERNAL MED DIGEST CLIN 1 0.026

APIS FLORA IND COMERCIAL LTDA 1 0.026

APTAMER SCI INC 1 0.026

ARABIAN GULF UNIV 1 0.026

ARBOR RES COLLABORAT HLTH 1 0.026

ARMED FORCES TAICHUNG GEN HOSP 1 0.026

ARMY MIL MED UNIV 1 0.026

ASAHI LIFE FDN 1 0.026

ASL TURIN 5 1 0.026

ASOU OIRM ST ANNA 1 0.026

ASOU SAN LUIGI GONZAGA 1 0.026

ASSOC LORRAINE TRAITEMENT INSUFFISANCE RENALE 1 0.026

ASTRA ZENECA 1 0.026

ASTRAZENECA GOTHENBURG 1 0.026

AT STILL UNIV 1 0.026

ATATURK STATE HOSP 1 0.026

ATATURK UNIV 1 0.026

ATHENS NAVAL HOSP 1 0.026

ATHENS UNIV 1 0.026

ATLANTA VA MED CTR 1 0.026

ATLANTIC CANC RES INST 1 0.026

AUCKLAND CITY HOSP 1 0.026

AUCKLAND DIST HLTH BOARD 1 0.026

AUDIE L MURPHY MEM VET ADM MED CTR 1 0.026

AUGSBURG CITY HOSP 1 0.026

AUGUSTA BIOMED RES CORP 1 0.026

AUSL DIABET UNIT ROMAGNA 1 0.026

AUSTIN REPATRIAT MED CTR 1 0.026

AUSTRALIA NEW ZEALAND DIALYSIS TRANSPLANT REG 1 0.026

AUSTRALIAN CATHOLIC UNIV 1 0.026

AUTONOMA UNIV MADRID UAM 1 0.026

AUTONOMA UNIV UAM 1 0.026

AVENTIS PHARMA DEUTSCHLAND GMBH 1 0.026

AZ DELTA 1 0.026

AZIENDA OSPED PAPA GIOVANNI XXIII 1 0.026

AZIENDA OSPED RIUNITI BERGAMO 1 0.026

AZIENDA OSPED UNIV 1 0.026

AZIENDA SOCIO SANIT TERR ASST PAPA GIOVANNI XXIII 1 0.026

BAD GLEICHENBERG CLIN 1 0.026

BAICHENG MED COLL 1 0.026

BAKER IDL HEART DIABET INST 1 0.026

BAKER RES INST 1 0.026

BANGALDESH AGR UNIV 1 0.026

BANGHABANDHU SHEIKH MUJIB MED UNIV 1 0.026

BANGLADESH UNIV HLTH SCI 1 0.026

BANTING BEST DIABET CTR 1 0.026

BAODING FIRST HOSP 1 0.026

BAOJI PEOPLES HOSP 1 0.026

BARNES JEWISH HOSP 1 0.026

BARRETOS CANC HOSP 1 0.026

BARSHOP INST LONGEV AGING STUDIES 1 0.026

BARUCH PADEH PORIYA MED CTR 1 0.026

BARWON MED IMAGING 1 0.026

BASSINI ICP HOSP 1 0.026

BAXTER HEALTHCARE 1 0.026

BAYER HEALTHCARE 1 0.026

BAYLOR HEART VASC INST 1 0.026

BEHESHTI UNIV MED SCI 1 0.026

BEIHANG UNIV 1 0.026

BEIJING ANZHEN HOSP 1 0.026

BEIJING INST HEPATOL 1 0.026

BEIJING INST RADIAT MED 1 0.026

BEIJING KEY LAB DIABET RES CARE 1 0.026

BEIJING KEY LAB IMMUNE MEDIATED INFLAMMATORY DIS 1 0.026

BEIJING KEY LAB INNOVAT DRUG DISCOVERY TRADIT CHI 1 0.026

BEIJING KEY LAB METAB DISORDER RELATED CARDIOVASC 1 0.026

BEIJING KEY LAB TRADIT CHINESE MED COLLATERAL DIS 1 0.026

BEIJING SPORT UNIV 1 0.026

BEIJING UNIV 1 0.026

BEIJING UNIV TRADIT CHINESE MED 1 0.026

BEIRUT ARAB UNIV 1 0.026

BELLVITGE HOSP 1 0.026

BENHA UNIV 1 0.026

BENI SUEF UNIV 1 0.026

BERLIN INST HLTH CTR REGENERAT THERAPIES BCRT 1 0.026

BERTHA UNIV 1 0.026

BG MED 1 0.026

BGI TECHNOL 1 0.026

BHARATI VIDYAPEETH DEEMED UNIV 1 0.026

BHARATI VIDYAPEETH UNIV 1 0.026

BHU 1 0.026

BIH 1 0.026

BIKUR HOLIM HOSP 1 0.026

BINZHOU MED UNIV HOSP 1 0.026

BIO NANO SOLUT 1 0.026

BIOCRATES LIFE SCI AG 1 0.026

BIODONOSTIA INST 1 0.026

BIOGEM SCARL 1 0.026

BIOGEN INC 1 0.026

BIOMARKERS RES 1 0.026

BIOMED HELSINKI 1 0.026

BIOMED HELSINKI C318B 1 0.026

BIOMED RES FDN ACAD ATHENS 1 0.026

BIOMED RES INST SALAMANCA IBSAL 1 0.026

BIOMED RES INST ST PAU IIB ST PAU 1 0.026

BIOMEDICUM HELSINKI 1 0.026

BIONT AS 1 0.026

BIOVIEW INC 1 0.026

BIRMINGHAM CHILDRENS HOSP 1 0.026

BIRMINGHAM CHILDRENS HOSP NHS TRUST 1 0.026

BIRMINGHAM VET ADM MED CTR 1 0.026

BITS PILANI 1 0.026

BJELOVAR GEN HOSP 1 0.026

BLACKPOOL VICTORIA HOSP 1 0.026

BLR BIO LLC 1 0.026

BML INC 1 0.026

BOARD TRUSTEES DIALYSIS KIDNEY TRANSPLANTAT 1 0.026

BOEHRINGER INGELHEIM CANADA LTD 1 0.026

BOEHRINGER INGELHEIM FRANCE SAS 1 0.026

BOEHRINGER INGELHEIM INT GMBH 1 0.026

BOKU UNIV NAT RESOURCES APPL LIFE SCI 1 0.026

BON SECOURS HLTH SYST 1 0.026

BON SECOURS LIVER INST VIRGINIA 1 0.026

BOYS TOWN NATL RES HOSP 1 0.026

BOZHOU VOCAT TECH COLL 1 0.026

BRETONNEAU HOSP 1 0.026

BRISTOL MYERS SQUIBB 1 0.026

BRISTOL MYERS SQUIBB CO 1 0.026

BRISTOL MYERS SQUIBB R D 1 0.026

BRISTOL ROYAL HOSP CHILDREN 1 0.026

BRITISH COLUMBIA CANC RES CTR 1 0.026

BROAD INST 1 0.026

BROCK UNIV 1 0.026

BROOKDALE MED CTR 1 0.026

BROOKDALE UNIV HOSP 1 0.026

BROUSSAIS HOSP 1 0.026

BUDDHIST DALIN TZU CHI GEN HOSP 1 0.026

BURKE MED RES INST 1 0.026

CABINET ANAT CYTOL PATHOL RICHIER 1 0.026

CALIF STATE UNIV LONG BEACH 1 0.026

CALIF STATE UNIV SACRAMENTO 1 0.026

CAMBRIDGE UNIV HOSP NHS FDN TRUST 1 0.026

CAMILO CASTELO BRANCO UNIV UNICASTELO 1 0.026

CAMPBELLTOWN HOSP 1 0.026

CANADIAN CTR AGRI FOOD RES HLTH MED 1 0.026

CANBERRA HOSP HLTH SERV 1 0.026

CANTABRIA UNIV 1 0.026

CANTACUZINO NATL INST RES DEV MICROBIOL IMMUN 1 0.026

CANTERBURY DIST HLTH BOARD 1 0.026

CANTONAL HOSP FRAUENFELD 1 0.026

CAOXIAN PEOPLES HOSP 1 0.026

CAPES COORDENACAO APERFEICOAMENTO PESSOAL NIVEL S 1 0.026

CARDIOVASC RES CTR 1 0.026

CAREGGI UNIV HOSP 1 0.026

CARESS ALLIANCE NIKKO MEM HOSP 1 0.026

CARLOS CHAGAS INST 1 0.026

CARLOS III INST HLTH 1 0.026

CAROL DAVILA CLIN HOSP NEPHROL 1 0.026

CAROL DAVILA NEPHROL HOSP 1 0.026

CAROL DAVILA UNIV MED PHARM 1 0.026

CAROLINAS MED CTR 1 0.026

CARSO CONSORTIUM 1 0.026

CASE WESTERN RESERVE SCH MED 1 0.026

CATHOLIC UNIV ARGENTINA 1 0.026

CATHOLIC UNIV CHILE 1 0.026

CATHOLIC UNIV CROATIA 1 0.026

CATHOLIC UNIV DAEGU 1 0.026

CELGENE CORP 1 0.026

CENT ARKANSAS VET HEALTHCARE SYST LITTLE ROCK 1 0.026

CENT CLIN SCH 1 0.026

CENT HOSP BAZHONG CITY 1 0.026

CENT HOSP WUHAN 1 0.026

CENT MICHIGAN UNIV 1 0.026

CENT MUNICIPAL HOSP HUIZHOU 1 0.026

CENT TAIWAN UNIV SCI TECHNOL 1 0.026

CENT UTAH CLIN 1 0.026

CENTAURE 1 0.026

CERES GMBH EVALUAT RES 1 0.026

CH CHARTRES 1 0.026

CH LA ROCHELLE 1 0.026

CHAIRA MEDICA ASSOC 1 0.026

CHANG BING SHOW CHWAN MEM HOSP 1 0.026

CHANG GANG UNIV 1 0.026

CHANG GUNG MEM HOSP LINKOU 1 0.026

CHANG GUNG UNIV COLL MED 1 0.026

CHANGCHUN INST FOOD DRUG CONTROL 1 0.026

CHANGZHI MED COLL 1 0.026

CHANGZHOU FOURTH PEOPLES HOSP 1 0.026

CHAOYANG UNIV TECHNOL 1 0.026

CHARITE CAMPUS BENJAMIN FRANKLIN 1 0.026

CHARITE CAMPUS MITTE 1 0.026

CHARITE CAMPUS VIRCHOW KLINIKUM CVK 1 0.026

CHARITE MED UNIV BERLIN 1 0.026

CHARLES DARWIN UNIV 1 0.026

CHEJU HALLA GEN HOSP 1 0.026

CHENGDU MED COLL 1 0.026

CHENZHOU 1 PEOPLES HOSP 1 0.026

CHENZHOU 3RD PEOPLES HOSP 1 0.026

CHEST HOSP 1 0.026

CHI MEI FDN HOSP 1 0.026

CHIA NAN UNIV PHARM SCI 1 0.026

CHIAYI CHANG GUNG MEM HOSP 1 0.026

CHILDRENS HLTH QUEENSLAND 1 0.026

CHILDRENS HOSP BOSTON 1 0.026

CHILDRENS HOSP MED CTR 1 0.026

CHILDRENS HOSP MONTEFIORE 1 0.026

CHILDRENS HOSP PHILADELPHIA 1 0.026

CHILDRENS HOSP PITTSBURGH 1 0.026

CHILDRENS HOSP RES INST MANITOBA 1 0.026

CHILDRENS HOSP UNIV MED CTR HAMBURG EPPENDORF 1 0.026

CHILDRENS MED RES INST 1 0.026

CHILDRENS MEM HOSP 1 0.026

CHINA AGR UNIV 1 0.026

CHINA UNIV MIN TECHNOL 1 0.026

CHINESE ACAD MED SCI PEKING UNION MED COLL 1 0.026

CHINESE ACAD TRADIT CHINESE MED 1 0.026

CHINESE AMER RES INST DIABET COMPLICAT 1 0.026

CHINESE CULTURE UNIV 1 0.026

CHINESE MED HOSP JIANGXI PROV 1 0.026

CHINESE MED HOSP SHAANXI 1 0.026

CHINESE MINIST EDUC 1 0.026

CHINESE PEOPLE ARMED POLICE FORCES 1 0.026

CHINESE PEOPLES LIBERAT ARMY 202 HOSP 1 0.026

CHONGQING CHEM IND VOCAT COLL 1 0.026

CHONGQING CLIN RES CTR GERIATR 1 0.026

CHONGQING HOSP TRADIT CHINESE MED 1 0.026

CHONGQING KEY LAB TRADIT CHINESE MED PREVENT CU 1 0.026

CHONGQING KEY LAB TRANSLAT MED MAJOR METAB DIS 1 0.026

CHONGQING NANKAI MIDDLE SCH 1 0.026

CHOSUN UNIV 1 0.026

CHRISTCHURCH HOSP 1 0.026

CHRISTIAN MED COLL HOSP 1 0.026

CHRON DIS RES GRP 1 0.026

CHRU TOURS 1 0.026

CHU AMIENS 1 0.026

CHU ANGERS 1 0.026

CHU BESANCON 1 0.026

CHU CLERMONT FERRAND 1 0.026

CHU COTE 1 0.026

CHU LA MILETRIE 1 0.026

CHU LIEGE 1 0.026

CHU NANCY 1 0.026

CHU ROUEN 1 0.026

CHU SART TILMAN 1 0.026

CHU SART TILMAN B35 1 0.026

CHU ST LOUIS 1 0.026

CHU STRASBOURG 1 0.026

CHU TOULOUSE 1 0.026

CHUBU ROSAI HOSP 1 0.026

CHUM HOSP ST LUC 1 0.026

CHUNG HWU UNIV MED TECHNOL 1 0.026

CHUQ 1 0.026

CI PARHON UNIV HOSP 1 0.026

CIBERDEM BIOMED RES CTR DIABET ASSOCIATED METAB 1 0.026

CIBEREHD 1 0.026

CINCINNATI CHILDRENS HOSP 1 0.026

CINCINNATI CHILDRENS HOSP RES FDN 1 0.026

CLIN ALEMANA UNIV DESARROLLO 1 0.026

CLIN CTR VOJVODINA 1 0.026

CLIN GENET CTR 1 0.026

CLIN HOSP CTR REBRO 1 0.026

CLIN HOSP CTR RIJEKA 1 0.026

CLIN HOSP SPLIT 1 0.026

CLIN RENAL ASSOCIATES LTD 1 0.026

CLIN RES INST MONTREAL 1 0.026

CLIN UNIV UCL ST LUC UCL ST LUC 1 0.026

CMIC 1 0.026

CMIC HOLDINGS CO LTD 1 0.026

CMIC PHARMA SCI CO LTD 1 0.026

CNPQ CONSELHO NACL DESENVOLVIMENTO CIENT TECNOL 1 0.026

CNRS 1 0.026

COHEN CHILDRENS MED CTR NEW YORK 1 0.026

COINNOVAT CTR IIANGSU MARINE BIOIND TECHNOL 1 0.026

COLLABORAT INNOVAT CTR BIOTHERAPY 1 0.026

COLOMBIAN DIABET ASSOC 1 0.026

COLUMBIA PRESBYTERIAN MED CTR 1 0.026

COLUMBUS GEMELLI HOSP CATHOLIC UNIV 1 0.026

COLUMBUS NEIGHBORHOOD HLTH CTR 1 0.026

COMMUNITY HLTH SERV CTR XIANGFU 1 0.026

COMMUNITY HOSP DORTMUND 1 0.026

COMPETENCE CTR PERSONALIZED MED 1 0.026

CONACYT IPICYT CIIDZA 1 0.026

CONCORD HOSP 1 0.026

CONCORDIA UNIV 1 0.026

CONSTANTIN BRANCUSI UNIV 1 0.026

CONWAY INST BIOMOL BIOMED SCI 1 0.026

COORDINATING COMMISS NATL HLTH INST 1 0.026

CORDELIERS RES CTR 1 0.026

CORK UNIV HOSP 1 0.026

CORNELL UNIV 1 0.026

CORPORACIO SANITARIA PARC TAULI 1 0.026

CORRELOG SYST INC 1 0.026

CORTONA HOSP 1 0.026

COSMET CLIN GRP 1 0.026

CRG 1 0.026

CSIC 1 0.026

CSIR CENT FOOD TECHNOL RES INST 1 0.026

CSIR NEIST 1 0.026

CSIR NORTH EAST INST SCI TECHNOL 1 0.026

CTR ANNA MARIA ASTORI 1 0.026

CTR BIOL MOL SEVERO OCHOA 1 0.026

CTR BIOMED RES 1 0.026

CTR BIOMED RES LA RIOJA CIBIR 1 0.026

CTR CELL SIGNALING DRUG DISCOVERY RES 1 0.026

CTR CHRON KIDNEY DIS PERITONEAL DIALYSIS 1 0.026

CTR CLIN BASIC RES IRCCS 1 0.026

CTR DIS CONTROL PREVENT 1 0.026

CTR EDUC MED INVEST CLIN NORBERTO QUIRNO CEMIC 1 0.026

CTR EXCELLENCE NOREG 1 0.026

CTR GENET CLIN 1 0.026

CTR HIV HEPATOGASTROENTEROL 1 0.026

CTR HOSP GEN 1 0.026

CTR HOSP MULHOUSE 1 0.026

CTR HOSP REG UNIV NANCY 1 0.026

CTR HOSP UNIV 1 0.026

CTR HOSP UNIV MONTREAL 1 0.026

CTR HOSP UNIV STE JUSTINE 1 0.026

CTR IMAGING DIAG NEUROMED 1 0.026

CTR INTERNAL MED GASTROENTEROL HEPATOL ENDOCRIN 1 0.026

CTR INVEST BIOMED RED ENFERMEDADES HEPAT DIGEST 1 0.026

CTR INVEST BIOMED RED ENFERMEDADES RARAS CIBERER 1 0.026

CTR INVEST PRINCIPE FELIPE 1 0.026

CTR MED UNIV GENEVA 1 0.026

CTR MICROSCOPIE ELECT APPL BIOL 1 0.026

CTR OUTCOMES RES CLIN EPIDEMIOL CORE 1 0.026

CTR PAUL STRAUSS 1 0.026

CTR POPULAT STUDIES 1 0.026

CTR REGULACIO GENOM 1 0.026

CTR SOUTH UNIV 1 0.026

CTR TRANSLAT CANC RES TRANSLATUM 1 0.026

CTR TRANSLAT RES SYST MED 1 0.026

CTR UNIV SAUDE ABC 1 0.026

CTR UNIV ST PERES 1 0.026

CTY EMERGENCY HOSP TIMISOARA 1 0.026

CUHK 1 0.026

CUHK RES INST 1 0.026

CUHK SHENZHEN RES INST 1 0.026

CVK RES 1 0.026

CYCLER THERAPEUT 1 0.026

CZECH ACAD SCI 1 0.026

CZECH UNIV LIFE SCI 1 0.026

D109 DIABET CTR UHC 1 0.026

D109 HSC DIABET CTR 1 0.026

DA YEH UNIV 1 0.026

DAEWON FOREIGN LANGUAGE HIGH SCH 1 0.026

DAIICHI SANKYO CO LTD 1 0.026

DAINIPPON SUMITOMO PHARMA 1 0.026

DALARNA UNIV 1 0.026

DALHOUSIE UNIV 1 0.026

DANKOOK UNIV 1 0.026

DANONE RES 1 0.026

DANUBE UNIV KREMS 1 0.026

DAQINGSHI 4 HOSP 1 0.026

DAR ES SALAAM UNIV 1 0.026

DASMAN DIABET INST 1 0.026

DAZHOU CENT HOSP 1 0.026

DAZHOU MIL HOSP CHINESE PLA 1 0.026

DELTA UNIV SCI TECHNOL 1 0.026

DENT CLIN 1 0.026

DENVAR VA MED CTR 1 0.026

DEPT ANAT EMBRYOL 1 0.026

DEPT BIOL CHEM MOL PHARMACOL 1 0.026

DEPT CELLULAR MOL BIOL 1 0.026

DEPT CLIN LABS 1 0.026

DEPT ENDOCRINOL 1 0.026

DEPT GASTROENTEROL 1 0.026

DEPT INTERNAL MED A 1 0.026

DEPT INTERNAL MED E 1 0.026

DEPT LAB MED 1 0.026

DEPT MED 1 0.026

DEPT MED CLIN SCI 1 0.026

DEPT NEPHROL 1 0.026

DEPT PATHOL LAB MED 1 0.026

DEPT PATHOPHYSIOL 1 0.026

DEPT PEDIAT 1 0.026

DEPT PHARMACOL 1 0.026

DEPT PUBL HLTH MED 1 0.026

DEPT RADIOL 1 0.026

DEPT RENAL MED 1 0.026

DEPT SURG 1 0.026

DEPT VASC ENDOVASC SURG 1 0.026

DEPT VET AFFAIRS MED CTR 1 0.026

DERRIFORD HOSP 1 0.026

DIABET CARDIOVASC CTR 1 0.026

DIABET GLANDULAR DIS CLIN 1 0.026

DIABET NEPHROPATHY ACADEMICIAN WORKSTN HENAN PROV 1 0.026

DIMICCOLI HOSP 1 0.026

DIPARTIMENTO SCI CLIN ENDOCRINOL 1 0.026

DIPARTIMENTO SCI MED CHIRURG 1 0.026

DIV ENDOCRINOL METAB DIABET 1 0.026

DIV NEPHROL 1 0.026

DIV NEPHROL HYPERTENS 1 0.026

DOKKYO MED UNIV 1 0.026

DONG A UNIV 1 0.026

DONG WHA PHARMACEUT CO 1 0.026

DONGGUAN TUNGWAH HOSP 1 0.026

DONGKUK UNIV 1 0.026

DR ALMPGIBMS UNIV MADRAS 1 0.026

DR BHANUBEN NANAVATI COLL PHARM 1 0.026

DR CAROL DAVILA TEACHING HOSP NEPHROL 1 0.026

DR JOSIP BENCEVIC GEN HOSP 1 0.026

DR MOHANS DIABET SPECIALTIES CTR 1 0.026

DR SAMI ULUS MATERN CHILDRENS HOSP 1 0.026

DR SAMI ULUS MATERN CHILDRENS RES TRAINING HO 1 0.026

DR SIYAMI ERSEK RES TRAINING HOSP 1 0.026

DR VIRINDER SINGH KIDNEY CLIN DIALYSIS CTR 1 0.026

DRAKE UNIV 1 0.026

DUKE CLIN RES INST 1 0.026

DURHAM VA MED CTR 1 0.026

DWI LEIBNIZ INST INTERACT MAT 1 0.026

DZD EV 1 0.026

E CHINA NORMAL UNIV 1 0.026

EAST CAROLINA UNIV 1 0.026

EAST CHINA UNIV POLIT SCI LAW 1 0.026

EASTERN MEDITERRANEAN UNIV 1 0.026

EASTERN MICHIGAN UNIV 1 0.026

EASTERN VIRGINIA MED SCH 1 0.026

EASTON HOSP 1 0.026

EBOLI HOSP MARIA SS ADDOLORATA 1 0.026

EDAH UNIV 1 0.026

EDUC BUR HEI LONG JIANG PROV 1 0.026

EDUC RES HOSP 1 0.026

ELISABETH KRANKENHAUS ESSEN 1 0.026

ELM BANK 1 0.026

EN CHU KONG HOSP 1 0.026

ENCY2 CONSULTING 1 0.026

ENGN RES CTR CHINESE TRADIT VET MED 1 0.026

ENGN TECHNOL RES CTR ANTIAGING CHINESE HERBAL MED 1 0.026

ENGN TECHNOL RES CTR XENOTRANSPLANTAT H 1 0.026

EP JOSLIN RES LAB 1 0.026

ERNST MORITZ ARNDT UNIV MED GREIFSWALD 1 0.026

ERZURUM REG TRAINING RES HOSP 1 0.026

ETTORE SANSAVINI HLTH SCI FDN 1 0.026

EUNICE KENNEDY SHRIVER NATL INST CHILD HLTH HUM 1 0.026

EUROPEAN INST ONCOL 1 0.026

EVERCYTE 1 0.026

EVOTEC FRANCE SAS 1 0.026

EXELIXIS INC 1 0.026

F CRIN NETWORK 1 0.026

F HOFFMANN LA ROCHE CIE AG 1 0.026

FAC HLTH SCI 1 0.026

FAC MED ABC 1 0.026

FAC MED ALEXIS CARREL 1 0.026

FAC MED RANGUEIL 1 0.026

FAC PHARMACEUT BIOL SCI 1 0.026

FAC PHARMACEUT SCI 1 0.026

FACP 1 0.026

FAGHIHI HOSP 1 0.026

FAR E MEMORY HOSP 1 0.026

FATIH UNIV 1 0.026

FDN AMICO RIC MALATTIE RENALI 1 0.026

FDN AMICO RICERCA MALATTIE RENALI 1 0.026

FDN CA GRANDA OSPED MAGGIORE IST SCI 1 0.026

FDN CTR ESTUDIOS INFECTOL 1 0.026

FDN DAMICO RIC MALATTIE RENALI 1 0.026

FDN DON C GNOCCHI 1 0.026

FDN INIGO ALVAREZ DE TOLEDO 1 0.026

FDN IRCCS 1 0.026

FDN IRCCS CA GRANDA OSPED MAGGIORE POLICLIN 1 0.026

FDN IRCCS POLICLIN SAN MATTEO 1 0.026

FDN JIMENEZ DIAZ IIS FJD UAM 1 0.026

FDN RENAL INIGO ALVAREZ TOLEDO IRSIN REDINREN 1 0.026

FDN RENAL INIGO DE ALVAREZ TOLEDO IRSIN C JOSE AB 1 0.026

FDN SEIMC GESIDA 1 0.026

FED UNIV 1 0.026

FED UNIV GOIAS UFG 1 0.026

FED UNIV MINAS GERAIS UFMG 1 0.026

FED UNIV SAO PAULO UNIFESP 1 0.026

FEDER 1 0.026

FEINSTEIN INST MED RES 1 0.026

FENGHUA DIST PEOPLES HOSP 1 0.026

FENGXIAN PEOPLES HOSP 1 0.026

FIBROGEN 1 0.026

FIBROSIS RES LAB 1 0.026

FIBROTECH THERAPEUT 1 0.026

FIBROTECH THERAPEUT PTY LTD 1 0.026

FIRST CENT HOSP BAODING 1 0.026

FIRST HOSP HANGZHOU 1 0.026

FIRST HOSP JIAXING 1 0.026

FIRST HOSP YIBIN 1 0.026

FIRST PEOPLES FOSHAN HOSP 1 0.026

FIRST PEOPLES HOSP 1 0.026

FIRST PEOPLES HOSP HANGZHOU 1 0.026

FIRST PEOPLES HOSP JINAN CITY 1 0.026

FIRST PEOPLES HOSP JINGZHOU 1 0.026

FIRST PEOPLES HOSP LIANGSHAN 1 0.026

FIRST PEOPLES HOSP NEIJIANG 1 0.026

FIRST PEOPLES HOSP SHANGQIU 1 0.026

FIRST PEOPLES HOSP TAICHANG 1 0.026

FIRST PEOPLES HOSP WUJIANG DIST SUZHOU 1 0.026

FIRST PEOPLES HOSP YICHANG 1 0.026

FIRST PEOPLES HOSP YUN NAN PROV 1 0.026

FIVE HILLS HLTH REG 1 0.026

FJD 1 0.026

FLINDERS MED CTR 1 0.026

FOLKHALSAN INST GENET 1 0.026

FORENS SCI SA 1 0.026

FORTY FOURTH MIL HOSP 1 0.026

FOURTH PEOPLES HOSP SHENGYANG 1 0.026

FRANCIS CRICK INST 1 0.026

FRANZISKUS HOSP BERLIN 1 0.026

FREDERIKSBERG UNIV HOSP 1 0.026

FREE UNIV BRUSSELS 1 0.026

FREEMAN RD HOSP 1 0.026

FRENCH CLIN RES INFRASTRUCT NETWORK 1 0.026

FRESENIUS MED CARE GERMANY 1 0.026

FRIAT 1 0.026

FUJI OIL HOLDINGS INC 1 0.026

FUJIAN NORMAL UNIV 1 0.026

FUJIAN PROV HOSP 1 0.026

FUJIAN UNIV TRADIT CHINESE MED 1 0.026

FUKUI KOSEI HOSP 1 0.026

FUKUI MED SCH 1 0.026

FUKUOKA DENT COLL 1 0.026

FUKUSHIMA MED UNIV 1 0.026

FUKUSHIMA MED UNIV HOSP 1 0.026

FUSHUN MIN BUR 1 0.026

FUTURE MED CO 1 0.026

FUTURE UNIV EGYPT 1 0.026

FUWAI HOSP 1 0.026

FUYANG NORMAL UNIV 1 0.026

G GASLINI CHILDREN HOSP 1 0.026

GALILEE MED CTR 1 0.026

GALLY INT BIOMED RES CONSULTING LLC 1 0.026

GALWAY UNIV HOSP 1 0.026

GANSU KEY LAB ENDOCRINE METAB 1 0.026

GAZI UNIV 1 0.026

GE HEALTHCARE 1 0.026

GEELONG HOSP 1 0.026

GEN HOSP 1 0.026

GEN HOSP ARTA 1 0.026

GEN HOSP DAQING OIL FIELD 1 0.026

GEN HOSP FELTRE BELLUNO 1 0.026

GEN HOSP MED UNIT AMBULATORY CARE 1 0.026

GEN HOSP WESTERN THEATER COMMAND 1 0.026

GEN UNIV HOSP PRAGUE 1 0.026

GENKYOTEX 1 0.026

GEORG SPEYER HAUS 1 0.026

GEORGE WASHINGTON SCH MED 1 0.026

GEORGES POMPIDOU EUROPEAN HOSP 1 0.026

GERMAN CANC CONSORTIUM DKTK 1 0.026

GERMAN CTR CARDIOVASC RES DZHK PARTNER SITE 1 0.026

GERMAN CTR DIABET RES DZD 1 0.026

GERMAN CTR LUNG RES 1 0.026

GERMAN RES CTR ENVIRONM HLTH 1 0.026

GERMAN RES CTR FOOD CHEM 1 0.026

GIANNINA GASLINI CHILDRENS HOSP 1 0.026

GIFU PHARMACEUT UNIV 1 0.026

GIL HOSP 1 0.026

GIRESUN UNIV 1 0.026

GLASGOW ROYAL INFIRM 1 0.026

GLAXOSMITHKLINE 1 0.026

GLAXOSMITHKLINE INC 1 0.026

GLENFIELD HOSP 1 0.026

GLYNDWR UNIV 1 0.026

GNS SCI 1 0.026

GOLESTAN UNIV MED SCI 1 0.026

GONGYI PEOPLES HOSP 1 0.026

GOVT SIVAGANGAI MED COLL HOSP 1 0.026

GRADENIGO HOSP 1 0.026

GRIGORE T POPA UNIV MED 1 0.026

GROCHOWSKI HOSP 1 0.026

GUANGDONG ACAD AGR SCI 1 0.026

GUANGDONG ACAD GERIATR 1 0.026

GUANGDONG HOSP TRADIT CHINESE MED 1 0.026

GUANGDONG PROV ENGN LAB DRUGGABIL NEW DRUGS EVA 1 0.026

GUANGDONG PROV ENGN TECHNOL RES CTR DIS MODEL A 1 0.026

GUANGDONG PROV INST NEPHROL 1 0.026

GUANGDONG PROV PEOPLES HOSP 1 0.026

GUANGDONG SECOND PROVIN GEN HOSP 1 0.026

GUANGZHOU EIGHTH PEOPLES HOSP 1 0.026

GUANGZHOU KEY LAB DRUGGABIL ASSESSMENT BIOL ACT C 1 0.026

GUANGZHOU UNIV 1 0.026

GUANGZHOU UNIV TRADIT CHINESE MED 1 0.026

GUANGZHOU YOUDI BIOTECHNOL CO LTD 1 0.026

GUBRA APS 1 0.026

GUIYANG MED COLL 1 0.026

GUIYANG MED UNIV 1 0.026

GUIZHOU MED HOSP 1 0.026

GUIZHOU UNIV 1 0.026

GUIZHOU UNIV TRADIT CHINESE MED 1 0.026

GUOZHOU UNIV 1 0.026

GURU NANAK DEV UNIV 1 0.026

GUYS ST THOMAS NHS FDN TRUST 1 0.026

GV SONNY MONTGOMERY VA MED CTR 1 0.026

GV SONNY MONTGOMERY VET AFFAIRS MED CTR 1 0.026

GYEONGGI BIO CTR 1 0.026

HACHINOHE GAKUIN UNIV 1 0.026

HADASSAH MED CTR 1 0.026

HAERBIN MED UNIV 1 0.026

HAGA TEACHING HOSP 1 0.026

HAINAN MED COLL 1 0.026

HAINAN MED UNIV 1 0.026

HAINAN PROV NONG KEN HOSP 1 0.026

HAKODATE JR COLL 1 0.026

HAMAD HEALTHCARE QUAL INST 1 0.026

HAMAD MED CORP HOSP 1 0.026

HANDAN SECOND HOSP 1 0.026

HANGZHOU FIRST PEOPLES HOSP 1 0.026

HANGZHOU HAI QIN SANAT 1 0.026

HANNOVER MED SCH MHH 1 0.026

HANS KNOELL INST 1 0.026

HARBIN INST TECHNOL 1 0.026

HARBOR UCLA RES EDUC INST INC 1 0.026

HAROLD HAMM OKLAHOMA DIABET CTR 1 0.026

HARPER UNIV HOSP 1 0.026

HARRAN UNIV 1 0.026

HARVARD MED SCH BOSTON 1 0.026

HEART CTR LEIPZIG 1 0.026

HEART HOSP 1 0.026

HEBEI CIVIL AFFAIRS GEN HOSP 1 0.026

HEBEI COLLABORAT INNOVAT CTR CARDIOCEREBROVASC DI 1 0.026

HEBEI KEY LAB ANIM SCI 1 0.026

HEBEI PROV HOSP TRADIT CHINESE MED 1 0.026

HEBEI UNIV 1 0.026

HEBEI UNIV ENGN 1 0.026

HEFEI ION MED CTR 1 0.026

HEFEI LIFEON PHARMACEUT CO LTD 1 0.026

HEILONGJIANG ACAD MED SCI 1 0.026

HEILONGJIANG PROV ACAD CHINESE MED SCI 1 0.026

HEINRICH HEINE UNIV 1 0.026

HELEN L DORRIS INST NEUROL PSYCHIAT DISORDERS 1 0.026

HELIOS CLIN BERLIN BUCH 1 0.026

HELIOS CLIN DAMP 1 0.026

HELIOS KLIN 1 0.026

HELMHOLTZ INST PHARMACEUT RES SAARLAND HIPS 1 0.026

HENAN ACAD CHINESE MED 1 0.026

HENAN PROV HOSP TRADIT CHINESE MED 1 0.026

HENRY FORD HLTH CTR 1 0.026

HEPING HOSP 1 0.026

HERBERT IRVING COMPREHENS CANC CTR 1 0.026

HIGH TECH DIST PEOPLES HOSP 1 0.026

HIGHLAND HOSP 1 0.026

HIGHLY SPECIALIZED HOSP 1 0.026

HILLMAN CTR PEDIAT TRANSPLANTAT 1 0.026

HISTOBRIDGE LLC 1 0.026

HISTOCHEM SOC 1 0.026

HISTOSTEM CO 1 0.026

HKBU 1 0.026

HLTH MANAGEMENT INST 1 0.026

HLTH SCI STUDIES INST CASTILLA LEON IESCYL 1 0.026

HOKURIKU UNIV 1 0.026

HOMMA INTERNAL MED CARDIOVASC CLIN 1 0.026

HOP ANTOINE BECLERE 1 0.026

HOP BEAUJON 1 0.026

HOP BICHAT 1 0.026

HOP BROUSSAIS 1 0.026

HOP CROIX ROUSSE 1 0.026

HOP EDOUARD HERRIOT 1 0.026

HOP ENFANTS 1 0.026

HOP EUROPEEN GEORGES POMIDOU 1 0.026

HOP FOCH 1 0.026

HOP JEAN BERNARD 1 0.026

HOP KREMLIN BICETRE 1 0.026

HOP LA PITIE SALPETRIERE 1 0.026

HOP MAISON BLANCHE 1 0.026

HOP PAUL BROUSSE 1 0.026

HOP ROBERT DEBRE 1 0.026

HOP SACRE COEUR MONTREAL 1 0.026

HOP ST LOUIS 1 0.026

HOP UNIV GENEVE 1 0.026

HOP UNIV PITIE SALPETRIERE CHARLES FOIX 1 0.026

HOP XAVIER BICHAT 1 0.026

HORUS UNIV EGYPT 1 0.026

HOSP BADALONA GERMANS TRIAS PUJOL 1 0.026

HOSP BEIHUA UNIV 1 0.026

HOSP CENT ASTURIAS 1 0.026

HOSP CLIN 1 0.026

HOSP CLIN UNIV 1 0.026

HOSP CLIN UNIV LOZANO BLESA 1 0.026

HOSP CLINICAS PORTO ALEGRE 1 0.026

HOSP DEL MAR IMIM 1 0.026

HOSP DONOSTIA 1 0.026

HOSP FELICIO ROCHO 1 0.026

HOSP FERNANDO FONSECA 1 0.026

HOSP GALDAKANO 1 0.026

HOSP GEN ALICANTE 1 0.026

HOSP GEN MEXICO DR EDUARDO LICEAGA 1 0.026

HOSP GEN REG IMSS 1 0.026

HOSP GEN UNIV 1 0.026

HOSP GEN UNIV CONSORTIUM 1 0.026

HOSP GEN VALLE HEBRON 1 0.026

HOSP GREGORIO MARANON 1 0.026

HOSP JM RAMOS MEJIA 1 0.026

HOSP JOSEP TRUETA 1 0.026

HOSP JUAN RAMON JIMENEZ 1 0.026

HOSP KRNOV 1 0.026

HOSP LA PAZ 1 0.026

HOSP LLOBREGAT 1 0.026

HOSP MAR FUNDACIO IMIM 1 0.026

HOSP MARQUES VALDECILLA 1 0.026

HOSP PALAMOS 1 0.026

HOSP PRINCESA 1 0.026

HOSP SANTA CREU SANT PAU 1 0.026

HOSP SAO JOAO 1 0.026

HOSP SOOCHOW UNIV 1 0.026

HOSP SULTANAH BAHIYAH 1 0.026

HOSP UNIV 1 0.026

HOSP UNIV 12 OCTUBRE IMAS12 1 0.026

HOSP UNIV ARNAU VILANOVA 1 0.026

HOSP UNIV AUSTRAL 1 0.026

HOSP UNIV CANARIAS 1 0.026

HOSP UNIV CARLOS HAYA 1 0.026

HOSP UNIV COIMBRA 1 0.026

HOSP UNIV DOCE OCTUBRE 1 0.026

HOSP UNIV DR PESET 1 0.026

HOSP UNIV GETAFE 1 0.026

HOSP UNIV GUADALAJARA 1 0.026

HOSP UNIV INFANTA LEONOR 1 0.026

HOSP UNIV JOAN XXIII 1 0.026

HOSP UNIV LA FE 1 0.026

HOSP UNIV MIGUEL SERVET 1 0.026

HOSP UNIV MOSTOLES 1 0.026

HOSP UNIV NTRA SRA CANDELARIA 1 0.026

HOSP UNIV POLITECN LA FE 1 0.026

HOSP UNIV PRINCIPE ASTURIAS 1 0.026

HOSP UNIV RAMON CAJAL 1 0.026

HOSP UNIV SEVERO OCHOA 1 0.026

HOSP UNIV TENERIFE 1 0.026

HOSP UNIV VALL HEBRON 1 0.026

HOSP UNIV VIRGEN DEL ROCIO 1 0.026

HOSP VALL DHEBRON RES INST VHIR 1 0.026

HOSP VIRGEN DEL ROCIO 1 0.026

HOSP VIRGEN MACARENA 1 0.026

HOSP XUZHOU MED COLL 1 0.026

HOSP XUZHOU UNIV TECHNOL 1 0.026

HOUSTON METHODIST RES INST 1 0.026

HOWARD UNIV 1 0.026

HUAIHAI INST TECHNOL 1 0.026

HUAINAN FIRST PEOPLES HOSP 1 0.026

HUAIYIN NORMAL UNIV 1 0.026

HUBEI KEY LAB CARDIOL 1 0.026

HUBEI PROV KEY LAB DEVELOPMENTALLY ORIGINATED DIS 1 0.026

HUBEI UNIV ARTS SCI 1 0.026

HUBEI UNIV CHINESE MED 1 0.026

HULL UNIV TEACHING HOSP NHS TRUST 1 0.026

HULL YORK MED SCH 1 0.026

HUMANITAS HOSP ROZZANO MILANO 1 0.026

HUMANITAS SCI INST 1 0.026

HUNTER HOLMES MCGUIRE VA MED CTR 1 0.026

HYOGO PREFECTURAL KOBE CHILDRENS HOSP 1 0.026

IATRIKO PALEOU FALIROU MED CTR 1 0.026

IBENS 1 0.026

IBIS 1 0.026

IBSAL HOSP UNIV SALAMANCA 1 0.026

ICMR CTR ADV RES DIABET 1 0.026

IDISNA 1 0.026

IFI INST INTERDISCIPLINARY MED 1 0.026

IFLB 1 0.026

IHOPE INT 1 0.026

IIBB 1 0.026

IIS FDN JIMENEZDIAZ 1 0.026

IISGM 1 0.026

IIT 1 0.026

IMBECU CONYCET 1 0.026

IMIM HOSP MAR MED RES INST 1 0.026

IMMTECH INT INC 1 0.026

IMMUNDIAGNOST AG 1 0.026

IMPERIAL COLL BUSINESS STUDIES 1 0.026

IMPERIAL COLL MED 1 0.026

IMPRESS FOOT ANKLE 1 0.026

IMTTS 1 0.026

INADA MED CLIN 1 0.026

INCHEON ST MARYS HOSP 1 0.026

INDIAN INST SCI 1 0.026

INDIAN INST SCI EDUC RES 1 0.026

INDIANA UNIV PURDUE UNIV 1 0.026

INDIANAPOLIS VET ADM MED CTR 1 0.026

INDRAPRASTHA APOLLO HOSP 1 0.026

INDUS BIOTECH PRIVATE LTD 1 0.026

INGHAM INST APPL MED RES 1 0.026

INHA UNIV MED 1 0.026

INI CRCT 1 0.026

INNER MONGOLIA FORESTRY GEN HOSP 1 0.026

INNER MONGOLIA MED COLL 1 0.026

INNER MONGOLIA UNIV SCI TECHNOL 1 0.026

INOKUCHI CLIN 1 0.026

INOVA FAIRFAX HOSP 1 0.026

INOVA HEART VASC INST 1 0.026

INOVA HLTH SYST 1 0.026

INRA 1 0.026

INSELSPITAL BERN 1 0.026

INSELSPITAL UNIV HOSP 1 0.026

INSERM U1138 TEAM 1 1 0.026

INSERM U845 1 0.026

INST ADV STUDIES SCI TECHNOL 1 0.026

INST BIORESPONSE INFORMAT 1 0.026

INST BIOTECHNOL CAS 1 0.026

INST CARLOS III FEDER 1 0.026

INST CELLULAR BIOL PATHOL NICOLAE SIMIONESCU 1 0.026

INST CHEM TECHNOL 1 0.026

INST CORDELIERS 1 0.026

INST CURIE 1 0.026

INST FRANCILIEN RECH NEPHROL TRANSPLANTAT 1 0.026

INST HLTH OUTCOMES PROC EVALUAT RES 1 0.026

INST HOSPITALIZAT CARE SCI CTR NEUROLESI BONINO 1 0.026

INST INVEST BIOMED AUGUST PI SUNYER IIBB CSIC I 1 0.026

INST INVEST METABOL 1 0.026

INST INVEST SANITARIA GREGORIO MARANON 1 0.026

INST INVEST SANITARIA LA PAZ 1 0.026

INST INVEST SANITARIA NAVARRA IDISNA 1 0.026

INST LAB MED BERLIN GMBH 1 0.026

INST LORRAIN COEUR VAISSEAUX 1 0.026

INST LOUIS BUGNARD 1 0.026

INST MOL CELL BIOL 1 0.026

INST MOL MED FINLAND 1 0.026

INST NACL NUTR SALVADOR ZUBIRAN 1 0.026

INST NEPHROL 1 0.026

INST NUTRIT SCI 1 0.026

INST PATHOL 1 0.026

INST PATHOL LUTHERSTADT EISLEBEN 1 0.026

INST QUEEN SOPHIE RENAL RES 1 0.026

INST RECERCA BIOMED LLEIDA 1 0.026

INST RECERCA ONCOL 1 0.026

INST RECH INT SERVIER CO DEV 1 0.026

INST RECH INTERDISCIPLINAIRE BIOL HUMAINE MOL 1 0.026

INST RECH SERVIER 1 0.026

INST REINA SOFIA INVEST RENAL 1 0.026

INST TRANSLAT HLTH SCI 1 0.026

INST UNIV CARDIOL PNEUMOL QUEBEC 1 0.026

INST UNIV FRANCE 1 0.026

INT TRAVEL HLTH CARE CTR 1 0.026

INT UNIV AFRICA 1 0.026

INT UNIV HLTH WELF 1 0.026

INTERCEPT 1 0.026

INTERUNIV CARDIOL INST NETHERLANDS 1 0.026

INVENTIV HLTH CTR 1 0.026

INVEST MED SONORA SC 1 0.026

IOWA STATE UNIV SCI TECHNOL 1 0.026

IPN 1 0.026

IQVIA 1 0.026

IRCCS CA GRANDA 1 0.026

IRCCS CA GRANDA FDN 1 0.026

IRCCS CASA SOLLIEVO SOFFERENZA 1 0.026

IRCCS GRP MULTIMED 1 0.026

IRCCS IST SCI SAN RAFFAELE 1 0.026

IRCCS MULTIMED 1 0.026

IRCCS OSPED SAN RAFFAELE 1 0.026

IRONWOOD PHARMACEUT INC 1 0.026

IRRIV 1 0.026

ISALA CLIN 1 0.026

ISF COLL PHARM 1 0.026

ISFAHAN UNIV MED SCI 1 0.026

ISHIKAWA KINENKAI KAWAGOE EKIMAE CLIN 1 0.026

ISKRA IND CO LTD 1 0.026

ISLAMIC AZAD UNIV 1 0.026

IST RIC FARMACOL MARIO NEGRI 1 0.026

IST RIC FARMACOL MARIO NEGRI IRCCS 1 0.026

IST SUPER SANITA 1 0.026

IST VET NOVARA 1 0.026

ITB 1 0.026

IWATE MED UNIV 1 0.026

IWK HLTH CTR 1 0.026

IZAAK WALTON KILLAM HOSP 1 0.026

IZMIR KATIP CELEBI UNIV 1 0.026

IZMIR TEPECIK TRAINING RES HOSP 1 0.026

JA HIROSHIMA GEN HOSP 1 0.026

JAGIELLONIAN UNIV MED COLL 1 0.026

JAIPUR DIABET RES CTR 1 0.026

JAMES J PETER VET ADM 1 0.026

JAMES J PETERS VET AFFAIRS MED CTR 1 0.026

JAMIA MILLIA ISLAMIA 1 0.026

JANSSEN RES DEV 1 0.026

JAPAN AGCY MED RES DEV 1 0.026

JAPAN TOBACCO INC 1 0.026

JAPAN WOMENS UNIV 1 0.026

JEFFERSON COLL HLTH SCI 1 0.026

JEONJU AGROBIOMAT INST 1 0.026

JESSE BROWN VET ADM HOSP 1 0.026

JESSE BROWN VET AFFAIRS MED CTR 1 0.026

JIAMUSI UNIV 1 0.026

JIANG YOU PEOPLES HOSP 1 0.026

JIANGSHAN PEOPLES HOSP 1 0.026

JIANGSU ENGN RES CTR MICRORNA BIOL BIOTECHNOL 1 0.026

JIANGSU JIANKANG VOCAT COLL 1 0.026

JIANGSU JIANKANG VOCAT UNIV 1 0.026

JIANGSU KEY LAB MARINE BIOTECHNOL 1 0.026

JIANGSU PROV ACAD CHINESE MED 1 0.026

JIANGSU PROV HOSP TRADIT CHINESE MED 1 0.026

JIANGSU PROV INST TRADIT CHINESE MED 1 0.026

JIANGSU QIANHONG ENGN RES CTR INNOVAT BIOL DRUGS 1 0.026

JIANGSU SUZHONG PHARMACEUT GRP CO LTD 1 0.026

JIANGSU TAIZHOU PEOPLES HOSP 1 0.026

JIANGXI CANC HOSP 1 0.026

JIANGXI PROV PEOPLES HOSP 1 0.026

JIANGXI UNIV TRADIT CHINESE MED 1 0.026

JIANGYIN PEOPLES HOSP 1 0.026

JIAXIANG CTY MED HOSP 1 0.026

JICHI MED SCH 1 0.026

JILIN AGR UNIV 1 0.026

JILIN CENT GEN HOSP 1 0.026

JINLING HOSP 1 0.026

JINNAH POSTGRAD MED CTR 1 0.026

JIUJIANG UNIV 1 0.026

JKK NATTRAJA COLL PHARM 1 0.026

JKK NATTRAJA COLL PHARMA 1 0.026

JOHN RADCLIFFE HOSP 1 0.026

JOHNS HOPKINS SCH MED 1 0.026

JOHNS HOPKINS UNIV HOSP 1 0.026

JOHNS HOPKINS VASCULIT CTR 1 0.026

JOHNSON JOHNSON INC 1 0.026

JORDAN UNIV SCI TECHNOL 1 0.026

JOSE MARIA CULLEN HOSP 1 0.026

JOSLIN CLIN 1 0.026

JST 1 0.026

JUNTENDO TOKYO KOTO GERIATR MED CTR 1 0.026

JUSTUS LIEBIG UNIV GIESSEN 1 0.026

JUVENILE DIABET RES FDN 1 0.026

JW GOETHE UNIV FRANKFURT 1 0.026

KACST 1 0.026

KAFKAS UNIV 1 0.026

KAHRAMANMARAS SUTCU IMAM UNIV 1 0.026

KAIRUKI HOSP 1 0.026

KAISER PERMANENTE 1 0.026

KAISER PERMANENTE NO CALIF 1 0.026

KALISIZO HOSP UGANDA 1 0.026

KAMILLIANER HOSP MONCHENGLADBACH 1 0.026

KANAGAWA UNIV HUMAN SERV 1 0.026

KANAZAWA MED CTR 1 0.026

KANGNAM ST MARYS HOSP 1 0.026

KANSAI ELECT POWER MED RES INST 1 0.026

KANSAI MED UNIV 1 0.026

KANSAI UNIV 1 0.026

KANSAS STATE UNIV 1 0.026

KANTONSSPITAL 1 0.026

KANUNI SULTAN SULEYMAN TRAINING RES HOSP 1 0.026

KAOHSIUNG VET GEN HOSP 1 0.026

KARL FRANZENS UNIV GRAZ 1 0.026

KARSIYAKA STATE HOSP 1 0.026

KARTAL RES TRAINING HOSP 1 0.026

KARTAL TRAINING HOSP 1 0.026

KATHOLIEKE UNIV LEUVEN HOSP 1 0.026

KATIP CELEBI UNIV 1 0.026

KBNP TECHNOL INST 1 0.026

KBSI 1 0.026

KEY LAB DRUG TARGET RES PHARMACODYNAM EVALUAT H 1 0.026

KEY LAB KIDNEY DIS HEBEI PROV 1 0.026

KEY LAB MODERN BIORNANUFACTURING ANHUI PROV 1 0.026

KEY LAB NEPHROL BLOOD PURIFICAT HUNAN 1 0.026

KEY LAB PEOPLES LIBERAT ARMY 1 0.026

KEY LAB PRECIS DIAG TREATMENT CHRON KIDNEY DIS 1 0.026

KFRI 1 0.026

KH ELISABETHINEN 1 0.026

KHON KAEN UNIV 1 0.026

KHULNA UNIV 1 0.026

KIDNEY CARE 1 0.026

KIDNEY CTR 1 0.026

KIDNEY RES CTR 1 0.026

KIDNEY RES INST 1 0.026

KIMS CLIN DIALYSIS UNIT 1 0.026

KING ABDULAZIZ UNIV HOSP 1 0.026

KING CHULALONGKORN MEM HOSP 1 0.026

KING SAOUD UNIV 1 0.026

KINGS HLTH PARTNERS ACAD HLTH SCI CTR AHSC 1 0.026

KITANO HOSP 1 0.026

KLINIKUM BERLIN 1 0.026

KLINIKUM BRANDENBURG 1 0.026

KLINIKUM COBURG 1 0.026

KLINIKUM GOETHE UNIV 1 0.026

KLINIKUM GOETHE UNIV FRANKFURT MAIN 1 0.026

KLINIKUM GROSSHADERN 1 0.026

KLINIKUM JW GOETHE UNIV 1 0.026

KLINIKUM KLAGENFURT WORTHERSEE 1 0.026

KLINIKUM UNIV 1 0.026

KOBE CITY GEN HOSP 1 0.026

KOCAELI UNIV 1 0.026

KOGA RED CROSS HOSP 1 0.026

KOLLING INST MED RES 1 0.026

KONKUK UNIV 1 0.026

KONYA RES TRAINING HOSP 1 0.026

KONYA UNIV 1 0.026

KOREA CTR DIS CONTROL PREVENT 1 0.026

KOREA FOOD RES INST 1 0.026

KOREA UNIV SCI TECHNOL UST 1 0.026

KOSAIR CHILDRENS HOSP 1 0.026

KRANKENHAUS HARLACHING 1 0.026

KRINGLE PHARMA INC 1 0.026

KU LEUVEN UNIV LEUVEN 1 0.026

KUANG TIEN GEN HOSP 1 0.026

KUBAN STATE MED UNIV 1 0.026

KURDISTAN UNIV MED SCI 1 0.026

KUREHA CORP 1 0.026

KUROBE MUNICIPAL HOSP 1 0.026

KURUME CHUO HOSP 1 0.026

KURUME INTERNAL MED ASSOC 1 0.026

KUSATSU GEN HOSP 1 0.026

KUWAIT UNIV 1 0.026

KWONG WAH HOSP 1 0.026

KYORIN UNIV 1 0.026

KYOTO UNIV HOSP 1 0.026

KYOWA HAKKO KIRIN CO LTD 1 0.026

LA BIOMED RES INST 1 0.026

LAB ATHEROSCLEROSIS METAB RES 1 0.026

LAB EXPT NEPHROL 1 0.026

LAB FISIOPATOL RENAL 1 0.026

LAB INTERDISCIPLINAR INVEST MED 1 0.026

LAB NATL SANTE 1 0.026

LAB RENAL PHYSIOPATHOL 1 0.026

LAB ROCHE 1 0.026

LABBAFINEJAD HOSP 1 0.026

LABEX INFLAMEX 1 0.026

LABIOMED 1 0.026

LADY CILENTO CHILDRENS HOSP 1 0.026

LADY HARDINGE MED COLL HOSP 1 0.026

LAIKO HOSP ATHENS 1 0.026

LANDSPITALI UNIV HOSP 1 0.026

LATIN AMER LIVER RES EDUC AWARENESS NETWORK LAL 1 0.026

LE BONHEUR CHILDRENS HOSP 1 0.026

LE SCOTTE HOSP 1 0.026

LEBANESE UNIV 1 0.026

LEBERSTIFTUNGS GMBH DEUTSCHLAND 1 0.026

LEEDS GEN INFIRM 1 0.026

LEEDS TEACHING HOSP NHS TRUST 1 0.026

LEEDS TEACHING HOSP TRUST 1 0.026

LEES ENDOCRINOL CLIN 1 0.026

LEXICON PHARMACEUT 1 0.026

LI KA SHING INST HLTH SCI 1 0.026

LIANSHUI CTY PEOPLES HOSP 1 0.026

LIAONING UNIV TCM 1 0.026

LIBIN CARDIOVASC INST ALBERTA 1 0.026

LIHUILI EASTERN HOSP 1 0.026

LILLE INFLAMMAT RES INT CTR 1 0.026

LILLE MED SCH 1 0.026

LINYI CITY PEOPLE HOSP 1 0.026

LIQUN HOSP 1 0.026

LIYANG HOSP TRADIT CHINESE MED 1 0.026

LKH HARTBERG 1 0.026

LOMA LINDA UNIV 1 0.026

LONDON HLTH SCI CTR 1 0.026

LONDON SCH HYG TROP MED 1 0.026

LORESTAN UNIV MED SCI 1 0.026

LOS ANGELES BIOMED RES INST 1 0.026

LOUGHBOROUGH UNIV 1 0.026

LOUIS B STOKES VET AFFAIRS MED CTR 1 0.026

LOUIS PRADEL HOSP 1 0.026

LOWELL GEN HOSP 1 0.026

LUDWIG BOLTZMANN INST TRANSLAT HEART FAILURE RES 1 0.026

LUDWIG MAXIMILIANS UNIV HOSP MUNICH 1 0.026

LUDWIG MAXIMILIANS UNIV MED MUNICH 1 0.026

LUNGSCREEN AUSTRALIA 1 0.026

LUTFI KIRDAR KARTAL RES EDUC HOSP 1 0.026

LUZHOU KEY LAB CARDIOVASC METAB DIS 1 0.026

M2RLAB XCELL 1 0.026

MAASSTAD HOSP 1 0.026

MAASTRICHT UNIV MED CTR 1 0.026

MACKAY MED NURSING MANAGEMENT COLL 1 0.026

MACKAY MEM HOSP 1 0.026

MACQUARIE UNIV 1 0.026

MADIGAN ARMY MED CTR 1 0.026

MADONNA GRAZIE HOSP 1 0.026

MAE FAH LUANG UNIV 1 0.026

MAGGIORE DELLA CARITA HOSP 1 0.026

MAGNA GRAECIA UNIV HOSP 1 0.026

MANCHESTER TEACHING HOSP NHS TRUST 1 0.026

MANSOURA FAC MED 1 0.026

MARBURG LUNG CTR 1 0.026

MARIO NEGRI INST PHARMACEUT RES 1 0.026

MARIO NEGRI IRCCS CENTRO ANNA MARIA ASTORI 1 0.026

MARNAC INC 1 0.026

MARTIN LUTHER UNIV HALLE WITTENBERG 1 0.026

MASARYK UNIV 1 0.026

MASHHAD UNIV MED SCI 1 0.026

MASSACHUSETTS COLL PHARM HLTH SCI 1 0.026

MASSEY UNIV 1 0.026

MASSONE INST 1 0.026

MATER HLTH 1 0.026

MATER RES 1 0.026

MATERN CHILDREN HOSP 1 0.026

MATSUYAMA RED CROSS HOSP 1 0.026

MAWLANA BHASHANI SCI TECHNOL UNIV 1 0.026

MAX DELBRUCK CTR MOL MED HELMHOLTZ ASSOC 1 0.026

MAX PLANCK INST BIOL AGEING 1 0.026

MAX PLANCK INST HEART LUNG RES 1 0.026

MAYO CLIN JACKSONVILLE 1 0.026

MAYO CLIN MAYO FDN 1 0.026

MAZANDARAN UNIV MED SCI 1 0.026

MAZANKOWSKI ALBERTA HEART INST 1 0.026

MAZE THERAPEUT 1 0.026

MBU TECHNOL LTD 1 0.026

MD ANDERSON CANC CTR 1 0.026

MEANDER MC AMERSFOORT 1 0.026

MEANDER MED CTR 1 0.026

MED ACAD 1 0.026

MED COLL MILWAUKEE 1 0.026

MED COLL PENN HAHNEMANN UNIV 1 0.026

MED CTR LEEUWARDEN 1 0.026

MED SCH NANCY 1 0.026

MED SCH RIBEIRAO PRETO 1 0.026

MED SCH XAVIER BICHAT 1 0.026

MED SUR CLIN FDN 1 0.026

MED UNIV 1 0.026

MED UNIV BIALYSTOK 1 0.026

MED UNIV COLOGNE 1 0.026

MED UNIV LUBLIN 1 0.026

MED UNIV SILESIA 1 0.026

MEDANTA 1 0.026

MEDIMMUNE 1 0.026

MEIJI PHARMACEUT UNIV 1 0.026

MEIJI UNIV 1 0.026

MEIJI UNIV INTEGRAT MED 1 0.026

MEIR MED CTR 1 0.026

MEKELLE UNIV 1 0.026

MEM MED CTR 1 0.026

MEM UNIV NEWFOUNDLAND 1 0.026

MENIA UNIV 1 0.026

MERAM RES TRAINING HOSP 1 0.026

MERCER UNIV 1 0.026

MERCK KGAA 1 0.026

METAXA GEN HOSP 1 0.026

METROPOLITAN HOSP 1 0.026

MEYER CHILDRENS UNIV HOSP 1 0.026

MEYER UNIV HOSP 1 0.026

MHC HOSP 1 0.026

MIAN YANG CENT HOSP 1 0.026

MICROS VET 1 0.026

MICROSOFT RES UNIV TRENTO 1 0.026

MILANO BICOCCA UNIV 1 0.026

MIN DONG HOSP NINGDE CITY 1 0.026

MINIST HLTH 23618104 1 0.026

MINIST HLTH CHINA 1 0.026

MIRIAM HOSP 1 0.026

MISSISSIPPI STATE UNIV 1 0.026

MITO RED CROSS HOSP 1 0.026

MITOCHONDRIAL THERAPEUT CONSULTING 1 0.026

MITSUBISHI NAGOYA HOSP 1 0.026

MITSUBISHI TANABE PHARMA CORP IKUYAKU 1 0.026

MIYAZAKI UNIV 1 0.026

MOHAMMED FIRST UNIV 1 0.026

MOLINETTE MAURIZIANO HOSP 1 0.026

MOMENTUM RES 1 0.026

MONASH BIOMED DISCOVERY INST 1 0.026

MONASH UNIV CENT 1 0.026

MONILEK HOSP RES CTR 1 0.026

MONTEFIORE HOSP 1 0.026

MONTREAL CHILDRENS HOSP 1 0.026

MONTREAL DIABET RES CTR 1 0.026

MONTREAL GEN HOSP 1 0.026

MOOREHOUSE SCH MED 1 0.026

MOSA DIAGNOST 1 0.026

MOSAIQUES DIAGNOST GMBH 1 0.026

MOSAIQUES DIAGNOST THERAPEUT AG 1 0.026

MOTHERS BABIES RES CTR 1 0.026

MRC 1 0.026

MRC HARWELL INST 1 0.026

MRC MILOCHONDRIAL BIOL UNIT 1 0.026

MS RAMIAH MED COLL 1 0.026

MT SINAI ST LUKES HOSP 1 0.026

MTA SE 1 0.026

MUCOS PHARMA 1 0.026

MUKAE INTERNAL MED CLIN 1 0.026

MUKOGAWA WOMENS UNIV 1 0.026

MUSASHINO UNIV 1 0.026

MUSTAFA KEMAL UNIV HOSP 1 0.026

MV HOSP DIABETES 1 0.026

MVZ MED LAB CTR DR STEIN PARTNER 1 0.026

MYMENSINGH MED COLL 1 0.026

MYMENSINGH MED COLL HOSP 1 0.026

N CAROLINA BAPTIST HOSP 1 0.026

NAFLD RES CTR 1 0.026

NAGASAKI INT UNIV 1 0.026

NAGOYA KYORITSU HOSP 1 0.026

NAKAMURA GAKUEN UNIV 1 0.026

NAMBOUR SELANGOR PRIVATE HOSP 1 0.026

NAN FANG SOUTHERN MED UNIV 1 0.026

NANFANG MED UNIV 1 0.026

NANJING RED CROSS BLOOD CTR 1 0.026

NANJING TECH UNIV 1 0.026

NANJING UNIV CHINESE MEDICISNE 1 0.026

NANJING UNIV SCI TECHNOL 1 0.026

NANYANG CENT HOSP 1 0.026

NANYANG INST TECHNOL 1 0.026

NARA MED UNIV 1 0.026

NARA MED UNIV HOSP 1 0.026

NASHVILLE VET ADM HOSP 1 0.026

NATL AIDS RES INST 1 0.026

NATL APPL RES LABS 1 0.026

NATL AUTONOMOUS UNIV MEXICO UNAM 1 0.026

NATL CANC CTR 1 0.026

NATL CENT UNIV 1 0.026

NATL CEREBRAL CARDIOVASC RES INST 1 0.026

NATL CLIN RES CTR KIDNEY DIS 1 0.026

NATL CLIN RES CTR KIDNEY DIS BEIJING CHINA 1 0.026

NATL FORMOSA UNIV 1 0.026

NATL GENOTYPING CTR 1 0.026

NATL GRAD INST POLICY STUDIES 1 0.026

NATL HLTH RES INST 1 0.026

NATL HOSP ORG KANAZAWA MED CTR 1 0.026

NATL INNOVAT CTR TCM MODERNIZAT 1 0.026

NATL INST DIABET DIGEST KIDNEY DIS 1 0.026

NATL INST DIABET DIGEST KIDNEY DIS NIDDK 1 0.026

NATL INST HLTH WELF 1 0.026

NATL INST LUNG DIS ISMAEL COSIO VILLEGAS 1 0.026

NATL INST MED SCI NUTR SALVADOR ZUBIRAN 1 0.026

NATL INST NUTR 1 0.026

NATL INST PHARMACEUT EDUC RES AHMEDABAD 1 0.026

NATL INST PHARMACEUT EDUC RES NIPER AHMEDABAD 1 0.026

NATL INST STAT ISTAT 1 0.026

NATL KAOHSIUNG UNIV SCI TECHNOL 1 0.026

NATL LIVER INST 1 0.026

NATL LOCAL UNITED ENGN LAB DRUG SCREENING EVALU 1 0.026

NATL POLYTECH INST 1 0.026

NATL POLYTECH INST CINVESTAV IPN 1 0.026

NATL POLYTECH INST IPN 1 0.026

NATL RES CTR GRAPES 1 0.026

NATL RES CTR POMEGRANATE 1 0.026

NATL SCH PUBL HLTH 1 0.026

NATL SUN YAT SEN UNIV 1 0.026

NATL TAIWAN NORMAL UNIV 1 0.026

NATL TSING HUA UNIV 1 0.026

NATL UNIV IRELAND GALWAY 1 0.026

NATL UNIV SINGAPORE HOSP 1 0.026

NATL YANG MING UNIV HOSP 1 0.026

NE NORMAL UNIV 1 0.026

NE OHIO UNIV 1 0.026

NE WALES INST 1 0.026

NEAR EAST UNIV 1 0.026

NECMETTIN ERBAKAN UNIV 1 0.026

NEPHI MED CLIN 1 0.026

NEPHROL LAB 1 0.026

NETHERLANDS ORG APPL SCI RES TNO 1 0.026

NETWORKING CTR BIOENGN BIOMAT NANOMED CIBER BBN 1 0.026

NEVILL HALL HOSP 1 0.026

NEW AREA HOSP 1 0.026

NEW ENGLAND MED CTR 1 0.026

NEW YORK PRESBYTERIAN HOSP WEILL CORNELL 1 0.026

NEW YORK STATE INST BASIC RES DEV DISABIL 1 0.026

NEWCASTLE TYNE HOSP NHS FDN TRUST 1 0.026

NEYSHABUR UNIV MED SCI 1 0.026

NHS BLOOD TRANSPLANT 1 0.026

NIAID 1 0.026

NICKAN RES INST 1 0.026

NICOLAE PAULESCU DIABET NUTR METAB DIS INST 1 0.026

NICOLAUS COPERNICUS UNIV 1 0.026

NIHR EXETER CLIN RES FACIL 1 0.026

NIIGATA UNIV HOSP 1 0.026

NINEWELLS HOSP 1 0.026

NINGBO 2 HOSP 1 0.026

NIOSH 1 0.026

NIPER 1 0.026

NIPPON MED SCH 1 0.026

NIPPON VET LIFE SCI UNIV 1 0.026

NISHIUMEDA CLIN ASIAN MED COLLABORAT 1 0.026

NMIMS UNIV 1 0.026

NO TERR DEPT HLTH COMMUNITY SERV 1 0.026

NOOSA HOSP 1 0.026

NORD BIOSCI 1 0.026

NORD BIOSCI BIOMARKERS RES 1 0.026

NORTH EAST MED COLL 1 0.026

NORTH KHORASAN UNIV MED SCI 1 0.026

NORTH SHORE UNIV 1 0.026

NORTHERN ONTARIO SCH MED 1 0.026

NORTHERN SYDNEY LOCAL HLTH DIST 1 0.026

NORTHERN UNIV SYDNEY 1 0.026

NORTHPORT VET ADM 1 0.026

NORTHSHORE UNIV HLTH SYST 1 0.026

NORTHWEST HOSP WOMEN CHILDREN 1 0.026

NORTHWEST UNIV FEINBERG SCH MED 1 0.026

NORTON CHILDREN 1 0.026

NORTON CHILDREN HOSP 1 0.026

NORWEGIAN UNIV SCI TECHNOL 1 0.026

NORWICH EATON PHARMACEUT 1 0.026

NOTRE DAME UNIV 1 0.026

NOUVEL HOP CIVIL 1 0.026

NOVARTIS INST BIOMED RES 1 0.026

NOVO NORDISK AS 1 0.026

NOVO NORDISK PK 1 0.026

NOVOTAIWAN BIOTECH 1 0.026

NOXXON PHARMA AG 1 0.026

NUMAZU CITY HOSP 1 0.026

NW MEM HOSP 1 0.026

NYU LANGONE MED CTR 1 0.026

OAKWOOD HEALTHCARE SYST 1 0.026

OATES BIOMED CONSULTING LLC 1 0.026

OCCUPAT DIS PREVENT CONTROL HOSP CHONGQING 1 0.026

ODENSE UNIV 1 0.026

OHIO UNIV OSTEOPATH MED 1 0.026

OITA MED UNIV 1 0.026

OKAN UNIV 1 0.026

OKAN UNIV MED HOSP ICMELER 1 0.026

OKAYAMA PREFECTURAL UNIV 1 0.026

OKAYAMA UNIV GRAD SCH MED DENT PHARMACEUT SCI 1 0.026

OKAYAMA UNIV SCI 1 0.026

OKINAKA MEM INST 1 0.026

OKINAWA DAIICHI HOSP 1 0.026

OKLAHOMA MED RES FDN 1 0.026

OLVG 1 0.026

ONASSIS CARDIAC SURG CTR 1 0.026

ONDOKUZ MAYIS UNIV 1 0.026

ONO PHARMACEUT 1 0.026

ONTARIO INST CANC RES 1 0.026

OPTUM 1 0.026

ORDU UNIV 1 0.026

OREBRO UNIV 1 0.026

OREBRO UNIV HOSP 1 0.026

ORG NACL TRASPLANTES 1 0.026

ORTHOPED HOSP SHENYANG 1 0.026

OSAKA BIOSCI INST 1 0.026

OSAKA GEN HOSP 1 0.026

OSAKA MED COLL 1 0.026

OSAKA MED CTR 1 0.026

OSAKA RED CROSS HOSP 1 0.026

OSAKA UNIV PHARMACEUT SCI 1 0.026

OSLO UNIV HOSP 1 0.026

OSPED A MANZONI 1 0.026

OSPED ACIREALE 1 0.026

OSPED MAGGIORE MILANO 1 0.026

OSPED MAGGIORE POLICLIN 1 0.026

OSPED PROV CIRCOLO 1 0.026

OSPED PROV G FORNAROLI 1 0.026

OSPED PROV MAGGIORE 1 0.026

OSPED PROV PREDABISSI 1 0.026

OSPED REG S MICHELE G BROTZU 1 0.026

OSPED SAN CARLO BORROMEO MILANO 1 0.026

OSPED SAN GERARDO 1 0.026

OSPED SAN GIOVANNI BOSCO 1 0.026

OSPED SAN MARTINO GENOVA 1 0.026

OSUN STATE UNIV 1 0.026

OULU UNIV HOSP 1 0.026

PACIFIC NORTHWEST RES INST 1 0.026

PACIFIC NW DIABET RES INST 1 0.026

PALAFITO 1 0.026

PAMELA YOUDE NETHERSOLE EASTERN HOSP 1 0.026

PAPWORTH HOSP 1 0.026

PAPWORTH HOSP NHS TRUST 1 0.026

PARACELSUS MED UNIV 1 0.026

PARIS DESCARTES SORBONNE PARIS CITE UNIV 1 0.026

PARIS ST JOSEPH HOSP 1 0.026

PARMA UNIV HOSP 1 0.026

PATHOL QUEENSLAND 1 0.026

PEAK DISCIPLINE INTEGRATED CHINESE WESTERN MED 1 0.026

PECS UNIV 1 0.026

PEKING TSINGHUA CTR LIFE SCI 1 0.026

PEKING UNION MED COLL CHINESE ACAD MED SCI 1 0.026

PEKING UNIV FIRST HOSP 1 0.026

PENGHU HOSP 1 0.026

PEOPLE HOSP LIAONING PROV 1 0.026

PEOPLE HOSP SHANXI PROV 1 0.026

PEOPLES HOSP GUANGXI ZHUANG AUTONOMOUS REG 1 0.026

PEOPLES HOSP GUIZHOU PROV 1 0.026

PEOPLES HOSP JIANGXI PROV 1 0.026

PEOPLES HOSP JILIN PROV 1 0.026

PEOPLES HOSP RIZHAO 1 0.026

PEOPLES HOSP RONGCHANG DISTRCT 1 0.026

PEOPLES HOSP XUECHENG 1 0.026

PEOPLES HOSP YONGCHUAN 1 0.026

PEOPLES LIBERAT ARMY GEN HOSP 1 0.026

PETROLEOS MEXICANOS 1 0.026

PFIZER GROTON RES DEV 1 0.026

PFIZER INC 1 0.026

PHARIS BIOTEC 1 0.026

PHARMACEUT UNIV 1 0.026

PHARMAHUNGARY GRP 1 0.026

PHARMAXIS LTD 1 0.026

PHARMAXIS PHARMACEUT LTD 1 0.026

PHENOS GMBH 1 0.026

PHILIPPS UNIV MARBURG 1 0.026

PHYSIOGENIX 1 0.026

PHYSIOL FUNCT EXPLORAT DEPT 1 0.026

PINNACLE CLIN RES 1 0.026

PIUS BRINZEU CTY EMERGENCY HOSP 1 0.026

PLA 1 0.026

PLA 81ST HOSP 1 0.026

PLA 85TH HOSP 1 0.026

PLA ROCKET FORCE CHARACTERIST MED CTR 1 0.026

PO ROYAL MELBOURNE HOSP 1 0.026

POLICE OUTPATIENT CLIN 1 0.026

POLICLIN IRCCS MULTIMED SESTO SAN GIOVANNI 1 0.026

POLICLIN MONZA 1 0.026

POLICLIN S ELENA 1 0.026

POLICLIN UMBERTO 1 1 0.026

POLICLIN UNIV PADUA 1 0.026

POLYTECH INST VISEU 1 0.026

POLYTECHN INST BRAGANCA 1 0.026

PONTIFICIA UNIV JAVERIAMA 1 0.026

PORTLAND STATE UNIV 1 0.026

PORTUGUESE DIABET ASSOC EDUC RES CTR APDP ERC 1 0.026

POZNAN UNIV MED SCI 1 0.026

PRESBYTERIAN MED CTR 1 0.026

PREVENTIONGENETICS 1 0.026

PRIMARY CARE UNIT VARDCENTRALEN 1 0.026

PRINCE CHARLES HOSP 1 0.026

PRINCE MOHAMED BIN ABDULAZIZ HOSP 1 0.026

PRINCE SATTAM BIN ABDULAZIZ UNIV 1 0.026

PRINCE SONGKLA UNIV 1 0.026

PROF M VISWANATHAN DIABET RES CTR 1 0.026

PROGRAM HUMAN MOL BIOL GENET 1 0.026

PROMET BIOSCI INC 1 0.026

PROVIDENCE HOSP MED CTR 1 0.026

PROVIDENCE PK HEART INST 1 0.026

PROVIDENCE SACRED HEART MED CTR 1 0.026

PROVIDENCE UNIV 1 0.026

PUBL CENT HOSP MATTO ISHIKAWA 1 0.026

PUNJABI UNIV 1 0.026

PUNZI MED CTR 1 0.026

PURDUE UNIV 1 0.026

PURDUE UNIV CALUMET 1 0.026

PURE NORTH SENERGY FDN 1 0.026

PUSAN NATL UNIV YANGSAN HOSP 1 0.026

PXBIOVISION GMBH 1 0.026

QATAR FDN 1 0.026

QIANFOSHAN HOSP SHANDONG PROV 1 0.026

QILU UNIV TECHNOL 1 0.026

QINGDAO MUNICIPAL HOSP 1 0.026

QINGDAO NATL LAB MARINE SCI TECHNOL 1 0.026

QINGDAO UNIV SCI TECHNOL 1 0.026

QUEEN ELIZABETH HOSP BIRMINGHAM 1 0.026

QUEEN ELIZABETH II MED CTR 1 0.026

QUEEN MARY HOSP 1 0.026

QUEEN MARY UNIV LONDON 1 0.026

QUEENS MED RES INST 1 0.026

QUEENS UNIV 1 0.026

QUEENSLAND UNIV TECHNOL 1 0.026

RADIANT RES 1 0.026

RADIOL RES 1 0.026

RAFIK HARIRI UNIV HOSP 1 0.026

RAKUNO GAKUEN UNIV 1 0.026

RAKUWAKAI OTOWA HOSP 1 0.026

RALPH H JOHNSON VET AFFAIRS MED CTR 1 0.026

RAMBAM MED CTR 1 0.026

RAMMELKAMP CTR RES EDUC 1 0.026

RAZI UNIV 1 0.026

RED CROSS HOSP 1 0.026

RED INVEST RENAL 1 0.026

REG INST PARAMED NURSING SCI 1 0.026

REG UNIV HOSP LILLE 1 0.026

REGENERON PHARMACEUT INC 1 0.026

RENAL ASSOCIATES PA 1 0.026

RENAL CARE CONSULTANTS 1 0.026

RENAL RES INST 1 0.026

RENAL UNIT 1 0.026

REPATRIAT MED CTR 1 0.026

REPUBL CLIN HOSP 1 0.026

RES CTR KIDNEY DIS 1 0.026

RES DEV BIOGEN 1 0.026

RES DEV CTR CHIATAI QINGCHUNBAO 1 0.026

RES INST 1 0.026

RES INST CHILD NUTR 1 0.026

RES INST MATERNAL CHILD HLTH 1 0.026

RES LAB BIOCHEM PATHOL 1 0.026

RES UNIT HOSP UNIV CANARIAS 1 0.026

RETROPHIN 1 0.026

RG KAR MED COLL HOSP 1 0.026

RICE MEM HOSP 1 0.026

RICHARD L ROUDEBUSH VA MED CTR 1 0.026

RIJNSTATE HOSP 1 0.026

RITS 1 0.026

ROBERT DEBRE HOSP 1 0.026

ROCHE SINGAPORE HUB TRANSLAT MED 1 0.026

ROGOSIN INST 1 0.026

ROMANIAN RENAL REGISTRY 1 0.026

ROSALES NATL HOSP 1 0.026

ROSALIND FRANKLIN MED SCI 1 0.026

ROUEN UNIV HOSP 1 0.026

ROYAL BRISBANE WOMENS HOSP 1 0.026

ROYAL FREE SCH MED 1 0.026

ROYAL GWENT HOSP 1 0.026

ROYAL HOLLOWAY UNIV LONDON 1 0.026

ROYAL HOSP SICK CHILDREN 1 0.026

ROYAL MARSDEN HOSP 1 0.026

RUDOLFSTIFTUNG HOSP 1 0.026

RUHR UNIV 1 0.026

RUHR UNIV BOCHUM 1 0.026

RUIJIN HOSP 1 0.026

RUSH MED CTR 1 0.026

RUTGERS NEW JERSEY MED SCH 1 0.026

RWTH UNIV 1 0.026

RWTH UNIV HOSP 1 0.026

RWTH UNIV HOSP AACHEN 1 0.026

S D CO LTD 1 0.026

S GIUSEPPE HOSP 1 0.026

S OSTROBOTHNIA CENT HOSP 1 0.026

S PAOLO HOSP 1 0.026

SABANCI UNIV 1 0.026

SABZEVAR UNIV MED SCI 1 0.026

SACYL 1 0.026

SAGA UNIV HOSP 1 0.026

SAGLIK BILIMLERI UNIV 1 0.026

SAHLGRENS UNIV HOSP 1 0.026

SAHLGRENSHA UNIV HOSP OSTRA 1 0.026

SAHLGRENSKA UNIV 1 0.026

SAHMYOOK MED CTR 1 0.026

SAITAMA MED SCH 1 0.026

SAITAMA PREFECTURAL UNIV 1 0.026

SAKARYA EGITIM ARASTIRMA HASTANESI 1 0.026

SAKARYA UNIV TRAINING RES HOSP 1 0.026

SAKURA NATL HOSP 1 0.026

SALEM VET AFFAIRS MED CTR 1 0.026

SAMSUNG BIOMED RES INST 1 0.026

SAMUEL LUNENFELD RES INST 1 0.026

SAN DIEGO HEALTHCARE SYST 1 0.026

SAN GIOVANNI DIO HOSP 1 0.026

SAN JUAN DIOS NATL HOSP 1 0.026

SANFORD BURNHAM PREBYS MED DISCOVERY INST 1 0.026

SANWA KAGAKU KENKYUSHO CO LTD 1 0.026

SAO JOSE DO RIO PRETO MED SCH 1 0.026

SAPIENZA UNIV ROME 1 0.026

SAPPORO MED UNIV 1 0.026

SAVITRIBAI PHULE PUNE UNIV 1 0.026

SCHENA FDN 1 0.026

SCHNEIDER CHILDRENS HOSP 1 0.026

SCHNEIDER CHILDRENS HOSP N SHORE LONG ISL JEWISH 1 0.026

SCHWABINGER KRANKENHAUS 1 0.026

SCI CLIN INST MAUGERI 1 0.026

SCRIPPS CLIN 1 0.026

SCRIPPS NEUROAIDS PRECLIN STUDIES CTR 1 0.026

SEATTLE CHILDRENS RES INST 1 0.026

SECHENOV MOSCOW MED ACAD 1 0.026

SECOND PEOPLES HOSP 1 0.026

SECOND PEOPLES HOSP HUNAN PROV 1 0.026

SECOND PEOPLES HOSP KUNSHAN 1 0.026

SECOND PEOPLES HOSP LIAOCHENG 1 0.026

SECOND UNIV NAPOLI 1 0.026

SECOND XIANGYA HOSP 1 0.026

SECRETARIA SALUD ESTADO VERACRUZ 1 0.026

SELCUK UNIV 1 0.026

SENDAI RED CROSS HOSP 1 0.026

SENDAI SHAKAIHOKEN HOSP 1 0.026

SENDELTA INT ACAD 1 0.026

SEOUL CORD BLOOD BANK 1 0.026

SEOUL METROPOLITAN GOVT BORAMAE MED CTR 1 0.026

SEOUL ST MARYS HOSP 1 0.026

SERODUS ASA 1 0.026

SERUM INST INDIA LTD 1 0.026

SETTIMO MILANESE 1 0.026

SGIT INIA 1 0.026

SHAANXI BLACK ORGAN FOOD ENGN CTR 1 0.026

SHAHEED BEHESHTI UNIV MED SCI 1 0.026

SHAHREKORD UNIV MED SCI 1 0.026

SHANDONG ACAD CLIN MED 1 0.026

SHANDONG ELECT POWER CENT HOSP 1 0.026

SHANDONG ENGN RES CTR NAT DRUG 1 0.026

SHANDONG ENGN TECHNOL RES CTR JUJUBE FOOD DRU 1 0.026

SHANDONG INST FOOD DRUG CONTROL 1 0.026

SHANDONG SPORTS UNIV 1 0.026

SHANDONG WEIFANG PEOPLES HOSP 1 0.026

SHANDONG WULIAN PEOPLES HOSP 1 0.026

SHANGHAI ACAD SCI TECHNOL 1 0.026

SHANGHAI CHEMPARTNER CO LTD 1 0.026

SHANGHAI EAST HOSP 1 0.026

SHANGHAI INNOVAT RES CTR TRADIT CHINESE MED 1 0.026

SHANGHAI INST BIOL SCI 1 0.026

SHANGHAI JIAO TONG UNIV AFFILIATED PEOPLES HOSP 6 1 0.026

SHANGHAI PUDONG NEW AREA PEOPLES HOSP 1 0.026

SHANGHAI PUNAN HOSP PUDONG NEW DIST 1 0.026

SHANGHAI RES INST HYPERTENS 1 0.026

SHANGHAI UNIV SCI TECHNOL 1 0.026

SHANXI ACAD MED SCI 1 0.026

SHANXI DATONG UNIV 1 0.026

SHANXI KIDNEY DIS INST 1 0.026

SHANXI UNIV CHINESE MED 1 0.026

SHANXIAN CENT HOSP 1 0.026

SHAOGUAN UNIV 1 0.026

SHEFFIELD TEACHING HOSP 1 0.026

SHEFFIELD TEACHING HOSP TRUST 1 0.026

SHENYANG 4TH HOSP PEOPLE 1 0.026

SHENYANG FOURTH PEOPLES HOSP 1 0.026

SHENYANG MED COLL 1 0.026

SHENYANG WOMENS CHILDRENS HOSP 1 0.026

SHENZHEN E GENE TECH CO LTD 1 0.026

SHENZHEN HENGSHENG HOSP 1 0.026

SHENZHEN KEY LAB HOSP CHINESE MED PREPARAT 1 0.026

SHENZHEN POLYTECH 1 0.026

SHENZHEN SECOND PEOPLES HOSP 1 0.026

SHENZHEN THIRD PEOPLES HOSP 1 0.026

SHIBAURA INST TECHNOL 1 0.026

SHIN KOGA HOSP 1 0.026

SHIRE 1 0.026

SHIZUOKA PREFECTURAL GEN HOSP 1 0.026

SHIZUOKA UNIV 1 0.026

SHONAN KOMAKURA GEN HOSP 1 0.026

SHOWA PHARMACEUT UNIV 1 0.026

SHUANGCHENG DIST PEOPLES HOSP 1 0.026

SICHUAN CLIN RES CTR NEPHROPATHY 1 0.026

SICHUAN UNIV SCI ENGN 1 0.026

SIEMENS HEALTHCARE 1 0.026

SILBERMAN INST LIFE SCI 1 0.026

SILESIAN CTR HEART DIS 1 0.026

SIMON FRASER UNIV 1 0.026

SINGAPORE BIOIMAGING CONSORTIUM 1 0.026

SINGAPORE GEN HOSP 1 0.026

SIR CHARLES GAIRDNER HOSP 1 0.026

SIR MORTIMER B DAVIS JEWISH HOSP 1 0.026

SIR RUN RUN SHAW HOSP 1 0.026

SISLI ETFAL EDUC RES HOSP 1 0.026

SIXTH PEOPLES HOSP 1 0.026

SK BIOLAND HAIMEN CO LTD 1 0.026

SKEJBY SYGEHUS 1 0.026

SLOVAK MED UNIV 1 0.026

SLOVAK TECH UNIV 1 0.026

SMITHKLINE BEECHAM PHARMACEUT 1 0.026

SO CROSS UNIV 1 0.026

SO ILLINOIS UNIV 1 0.026

SOC EXPT LAB MED 1 0.026

SOONCHUNHYANG UNIV SEOUL HOSP 1 0.026

SOROKA UNIV MED CTR 1 0.026

SOUTH AUSTRALIAN HLTH MED RES INST 1 0.026

SOUTH CHINA UNIV 1 0.026

SOUTHERN CROSS UNIV 1 0.026

SOUTHERN TAIWAN UNIV SCI TECHNOL 1 0.026

SOUTHERN UNIV SCI TECHNOL 1 0.026

SOUTHMEAD HOSP 1 0.026

SOUTHWEST NATL PRIMATE RES CTR 1 0.026

SOUTHWEST UNIV 1 0.026

SOUTHWESTERN HOSP 1 0.026

SPANISH KIDNEY RES NETWORK REDINREN 1 0.026

SPANISH RES NETWORK RENAL DIS REDINREN ISCIII 1 0.026

SPECIALIST CLIN REHABIL PV BAD AUSSEE 1 0.026

SPIRITO SANTO HOSP 1 0.026

SPIRITO SANTO HOSP PESCARA 1 0.026

SPP SCH PHARM TECHNOL MANAGEMENT SVKMS NMIMS 1 0.026

SRI INT 1 0.026

SRI RAMACHANDRA UNIV 1 0.026

SRR CVR DEGREE COLL 1 0.026

SS NEPHROL ASOU SAN LUIGI 1 0.026

ST ANNA UNIV HOSP 1 0.026

ST BONIFACE GEN HOSP 1 0.026

ST BORTOLO HOSP 1 0.026

ST FRANCIS REG MED CTR 1 0.026

ST GEORGE HOSP 1 0.026

ST GEORGES UNIV LONDON 1 0.026

ST JOHN PROVIDENCE HLTH SYST 1 0.026

ST LOUIS CHILDRENS HOSP 1 0.026

ST LOUIS HOSP PARIS 1 0.026

ST LOUIS UNIV 1 0.026

ST LUKES EPISCOPAL HOSP 1 0.026

ST LUKES HOSP 1 0.026

ST MARYS HOSP 1 0.026

ST PATRICKS COLL 1 0.026

ST PAULS HOSP 1 0.026

ST THOMAS HOSP 1 0.026

ST VINCENT DE PAUL HOSP 1 0.026

ST VINCENTS INST MED RES 1 0.026

STADT KLINIKUM BRAUNSCHWEIG GMBH 1 0.026

STAMFORD HOSP 1 0.026

STANFORD SCH MED 1 0.026

STARSHIP CHILDRENS HOSP 1 0.026

STATE ADM TRADIT CHINESE MED 1 0.026

STATE FOOD DRUG ADM 1 0.026

STATE KEY LAB CONSERVAT UTILIZAT BIORESOURCES Y 1 0.026

STATE KEY LAB FRESHWATER ECOL BIOTECHNOL 1 0.026

STATE KEY LAB ORGAN FAILURE RES 1 0.026

STATE UNIV HLTH SCI 1 0.026

STATE UNIV HOSP 1 0.026

STENO DIABET CTR COPENHAGEN SDCC 1 0.026

SUGITA GENPAKU MEM OBAMA MUNICIPAL HOSP 1 0.026

SUITA MUNICIPAL HOSP 1 0.026

SUNCHON NATL UNIV 1 0.026

SUNNYBROOK HLTH SCI CTR 1 0.026

SUNSHINE CLIN RES 1 0.026

SUNSHINE COAST UNIV PRIVATE HOSP 1 0.026

SUZHONG PHARMACEUT RES INST 1 0.026

SVKMS DR BHANUBEN NANAVATI COLL PHARM 1 0.026

SWAMI VIVEKANAND SUBHARTI UNIV 1 0.026

SWANSEA UNIV 1 0.026

SWISS TROP PUBL HLTH INST 1 0.026

SYDNEY MED SCH 1 0.026

SYMBIOSIS INT UNIV 1 0.026

SYNLAB ACAD 1 0.026

SYNLAB SERV GMBH 1 0.026

SYRACUSE UNIV 1 0.026

SYST BIOL IRELAND 1 0.026

SZEGED FAC MED 1 0.026

SZYY GRP PHARMACEUT LTD 1 0.026

TAIAN CENT HOSP 1 0.026

TAIBAH UNIV 1 0.026

TAICHUNG ARMED FORCES GEN HOSP 1 0.026

TAISHO PHARMACEUT CO LTD 1 0.026

TAIWAN YU SHING BIOTECH CO LTD 1 0.026

TAIZHOU FIRST PEOPLES HOSP 1 0.026

TAIZHOU PEOPLES HOSP 1 0.026

TAIZHOU UNIV 1 0.026

TAKEDA CHEM IND LTD 1 0.026

TAKEDA GEN HOSP 1 0.026

TAKEDA PHARMACEUT CO LTD 1 0.026

TAMPERE UNIV HOSP 1 0.026

TARBIAT MODARES UNIV 1 0.026

TARLETON STATE UNIV 1 0.026

TASC 1 0.026

TECH UNIV 1 0.026

TECHNION 1 0.026

TECHNOL DEV CO 1 0.026

TEIKYO UNIV 1 0.026

TEL AVIV MED CTR SCH MED 1 0.026

TEMPLE ST CHILDRENS UNIV HOSP 1 0.026

TENGION INC 1 0.026

TENGZHOU CENT PEOPLES HOSP 1 0.026

TEXAS A M HLTH SCI CTR 1 0.026

TEXAS HEART INST 1 0.026

TGHRI 1 0.026

THAMMASAT UNIV 1 0.026

THIRD HOSP MIANYANG 1 0.026

THIRD HOSP SHIJIAZHUANG 1 0.026

THIRD PEOPLES HOSP QINGDAO 1 0.026

THIRD XIANGYA HOSP 1 0.026

TIANJIN ACAD TRADIT CHINESE MED AFFILIATED HOSP 1 0.026

TIANJIN BEICHEN DIST CHINESE MED HOSP 1 0.026

TIANJIN FIRST CENT HOSP 1 0.026

TIANJIN KEY LAB TRANSLAT RES TCM PRESCRIPT SYND 1 0.026

TIANJIN MED DEVICES QUAL SUPERVIS TESTING CTR 1 0.026

TIANJIN MED UNIV GEN HOSP 1 0.026

TIANJIN PUBL SECUR HOSP 1 0.026

TIBET UNIV 1 0.026

TNO 1 0.026

TOCHIGI INST CLIN PATHOL 1 0.026

TOHO UNIV 1 0.026

TOHOKU FUKUSHI UNIV 1 0.026

TOHOKU UNIV HOSP 1 0.026

TOKUSHIMA UNIV 1 0.026

TOKUSHIMA UNIV HOSP 1 0.026

TOKYO METROPOLITAN GERIATR HOSP 1 0.026

TOKYO METROPOLITAN HIROO GEN HOSP 1 0.026

TOKYO METROPOLITAN INST GERONTOL TMIG 1 0.026

TOKYO SAISEIKAI CENT HOSP 1 0.026

TOKYO UNIV SCI 1 0.026

TOWNSVILLE HOSP 1 0.026

TOYAMA MED PHARMACEUT UNIV 1 0.026

TRADIT MED HOSP HEBEI PROV 1 0.026

TRANSLAT GENOM RES INST 1 0.026

TRANSLAT GENOM RES INST TGEN 1 0.026

TRI SERV GEN HOSP 1 0.026

TRIANGULO MINEIRO FED UNIV 1 0.026

TROOPS 93253 UNIT 1 0.026

TROOPS 95988 UNIT 1 0.026

TSINGHUA UNIV 1 0.026

TSUMURA CLIN 1 0.026

TUFTS MED CTR 1 0.026

TUFTS UNIV 1 0.026

TUNG WAH HOSP 1 0.026

TURGUT OZAL UNIV HASTANESI 1 0.026

TURKU UNIV HOSP 1 0.026

TZU CHI UNIV SCI TECHNOL 1 0.026

UBA CONICET 1 0.026

UCB CELLTECH PHARMACEUT 1 0.026

UCBL 1 1 0.026

UCD CONWAY INST BIOMOL BIOMED RES 1 0.026

UCD SCH BIOMOL BIOMED SCI 1 0.026

UCI 1 0.026

UCL INST CHILD HLTH 1 0.026

UCSD SCH MED 1 0.026

UDA CITY HOSP 1 0.026

UHN 1 0.026

UK HEALTHCARE 1 0.026

ULLEVAAL UNIV HOSP 1 0.026

ULUDAG UNIV 1 0.026

UMC UTRECHT 1 0.026

UMEA UNIV 1 0.026

UMR S1155 1 0.026

UNICYTE SRL 1 0.026

UNIDAD INVEST ENFERMEDADES NEFROL IMSS 1 0.026

UNIV ADELAIDE 1 0.026

UNIV ALCALA UAH 1 0.026

UNIV ANDRES BELLO 1 0.026

UNIV ATLANTA 1 0.026

UNIV AUTONOMA 1 0.026

UNIV AUTONOMA QUERETARO 1 0.026

UNIV AVEIRO 1 0.026

UNIV BARCELONA HOSP 1 0.026

UNIV BASILICATA 1 0.026

UNIV BASQUE COUNTRY 1 0.026

UNIV BASQUE COUNTRY UPV EHU 1 0.026

UNIV BEDFORDSHIRE 1 0.026

UNIV BELGRADE 1 0.026

UNIV BERGAMO 1 0.026

UNIV BIELEFELD 1 0.026

UNIV BOLOGNA 1 0.026

UNIV BOURGOGNE FRANCHE COMTE 1 0.026

UNIV BUCHAREST 1 0.026

UNIV BUENOS AIRES CONICET 1 0.026

UNIV BURGUNDY 1 0.026

UNIV CALIF BERKELEY 1 0.026

UNIV CALIF SAN DIEGO VA MED SYST 1 0.026

UNIV CALIF SANTA BARBARA 1 0.026

UNIV CAMILO CASTELO BRANCO 1 0.026

UNIV CAMPINAS 1 0.026

UNIV CATANZARO 1 0.026

UNIV CENT LANCASHIRE 1 0.026

UNIV CHILDRENS HOSP 1 0.026

UNIV CHILDRENS HOSP HEIDELBERG 1 0.026

UNIV CHILE 1 0.026

UNIV CLAUDE BERNARD 1 0.026

UNIV CLIN ESSEN 1 0.026

UNIV CLIN HOSP VIRGEN ARRIXACA 1 0.026

UNIV COLL HOSP 1 0.026

UNIV COLL LONDON HOSP 1 0.026

UNIV COLL MIDDLESEX SCH MED 1 0.026

UNIV COLORADO AMC 1 0.026

UNIV COLORADO ANSCHUTZ MED CAMPUS 1 0.026

UNIV COMPLUTENSE 1 0.026

UNIV CONCEPCION 1 0.026

UNIV CRETE 1 0.026

UNIV CRUZEIRO SUL 1 0.026

UNIV CYPRUS 1 0.026

UNIV DEBRECEN 1 0.026

UNIV DELHI 1 0.026

UNIV DIEGO PORTALES 1 0.026

UNIV DRESDEN 1 0.026

UNIV DURHAM 1 0.026

UNIV ESTADUAL LONDRINA 1 0.026

UNIV ESTADUAL MARINGA 1 0.026

UNIV ESTADUAL PAULISTA 1 0.026

UNIV FED BAHIA 1 0.026

UNIV FED CEARA 1 0.026

UNIV FED FLUMINENSE 1 0.026

UNIV FED GOIAS 1 0.026

UNIV FED JUIZ DE FORA 1 0.026

UNIV FED MATO GROSSO 1 0.026

UNIV FED PELOTAS 1 0.026

UNIV FED RIO DE JANEIRO 1 0.026

UNIV FED RIO GRANDE DO NORTE 1 0.026

UNIV FED RIO GRANDE NORTE 1 0.026

UNIV FED SANTA MARIA 1 0.026

UNIV FED TRIANGULO MINEIRO 1 0.026

UNIV FED UBERLANDIA 1 0.026

UNIV FEDERICO II NAPLES 1 0.026

UNIV FERRARA 1 0.026

UNIV FINDLAY 1 0.026

UNIV FIRENZE 1 0.026

UNIV FOGGIA 1 0.026

UNIV FORTALEZA 1 0.026

UNIV FRIBOURG 1 0.026

UNIV FUKUI HOSP 1 0.026

UNIV G DANNUNZIO 1 0.026

UNIV GENOA 1 0.026

UNIV GHENT 1 0.026

UNIV GIESSEN MARBURG LUNG CTR 1 0.026

UNIV GRENOBLE ALPES 1 0.026

UNIV GRONINGEN HOSP 1 0.026

UNIV GUYANA 1 0.026

UNIV HAIFA 1 0.026

UNIV HAIL 1 0.026

UNIV HAWAI HILO 1 0.026

UNIV HAWAII 1 0.026

UNIV HAWAII MANOA 1 0.026

UNIV HEIDELBERG ANAT ENTWICKLUNGSBIOL 1 0.026

UNIV HEIDELBERG HOSP 1 0.026

UNIV HELSINKI HOSP 1 0.026

UNIV HENRI POINCARE 1 0.026

UNIV HIROSHIMA 1 0.026

UNIV HLTH NETWORK 8N 849 1 0.026

UNIV HONG KONG SHENZHEN HOSP 1 0.026

UNIV HOSP AACHEN 1 0.026

UNIV HOSP ALBERT 1 0.026

UNIV HOSP AOU G MARTINO 1 0.026

UNIV HOSP BIRMINGHAM NHS FDN TRUST 1 0.026

UNIV HOSP BRANDENBURG 1 0.026

UNIV HOSP BRISTOL NHS FDN TRUST 1 0.026

UNIV HOSP BRNO 1 0.026

UNIV HOSP BRNO BOHUNICE 1 0.026

UNIV HOSP CASE MED CTR 1 0.026

UNIV HOSP CTR RIJEKA 1 0.026

UNIV HOSP GEELONG 1 0.026

UNIV HOSP HAMBURG EPPENDORF 1 0.026

UNIV HOSP HARTLEPOOL 1 0.026

UNIV HOSP INNSBRUCK 1 0.026

UNIV HOSP IOANNINA 1 0.026

UNIV HOSP LEICESTER NHS TRUST 1 0.026

UNIV HOSP LEIPZIG 1 0.026

UNIV HOSP MUENSTER 1 0.026

UNIV HOSP NUESTRA SENORA CANDELARIA 1 0.026

UNIV HOSP OSTRAVA 1 0.026

UNIV HOSP PATRAS 1 0.026

UNIV HOSP RIJEKA 1 0.026

UNIV HOSP RWTH 1 0.026

UNIV HOSP SANTA MARIA 1 0.026

UNIV HOSP SANTA MARIA MISERICORDIA 1 0.026

UNIV HOSP TUEBINGEN 1 0.026

UNIV HOSP VERONA 1 0.026

UNIV HOSP WURZBURG 1 0.026

UNIV HUDDERSFIELD 1 0.026

UNIV HYDERABAD 1 0.026

UNIV INSUBRIA 1 0.026

UNIV JINAN 1 0.026

UNIV JOHANNESBURG 1 0.026

UNIV JORDAN 1 0.026

UNIV KENTUCKY HEALTHCARE 1 0.026

UNIV KLIN JENA 1 0.026

UNIV KLINIKUM 1 0.026

UNIV KLINIKUM GOTTINGEN 1 0.026

UNIV KLINIKUM REGENSBURG 1 0.026

UNIV KLINIKUM RWTH AACHEN 1 0.026

UNIV KOREA HOSP 1 0.026

UNIV KWAZULU NATAL 1 0.026

UNIV LA LAGUNA 1 0.026

UNIV LAHORE 1 0.026

UNIV LAUSANNE HOSP 1 0.026

UNIV LEEDS 1 0.026

UNIV LIBRE BRUXELLES 1 0.026

UNIV LIEGE 1 0.026

UNIV LISBON 1 0.026

UNIV LONDON IMPERIAL COLL SCI TECHNOL 1 0.026

UNIV LOUGHBOROUGH 1 0.026

UNIV LUXEMBOURG 1 0.026

UNIV MAGDEBURG 1 0.026

UNIV MALAGA 1 0.026

UNIV MANSOURA 1 0.026

UNIV MARACAIBO 1 0.026

UNIV MARYLAND HOSP 1 0.026

UNIV MED BERLIN 1 0.026

UNIV MED CTR GOETTINGEN 1 0.026

UNIV MED DENT NEW JERSEY 1 0.026

UNIV MED FARM TIMISOARA 1 0.026

UNIV MED GOTTINGEN 1 0.026

UNIV MED PHARM IULIU HATIEGANU CLUJ NAPOCA 1 0.026

UNIV MED PHARM V BABE TIMISOARA 1 0.026

UNIV MED PHARM V BABES TIMISOARA 1 0.026

UNIV MED PHARM VICTOR BABE TIMISOARA 1 0.026

UNIV MEDITERRANEE 1 0.026

UNIV MISSOURI COLUMBIA 1 0.026

UNIV MIYAZAKI 1 0.026

UNIV MODENA 1 0.026

UNIV MOLISE 1 0.026

UNIV MONS UMONS 1 0.026

UNIV MONTREAL CRCHUM 1 0.026

UNIV N DAKOTA 1 0.026

UNIV NACL CORDOBA 1 0.026

UNIV NACL TUCUMAN 1 0.026

UNIV NAMUR 1 0.026

UNIV NANTES 1 0.026

UNIV NANTONG 1 0.026

UNIV NAPLES FEDERICO 2 1 0.026

UNIV NAPLES FEDERICO II 1 0.026

UNIV NAPOLI FEDERICO II 1 0.026

UNIV NEWCASTLE UPON TYNE 1 0.026

UNIV NIS 1 0.026

UNIV NO BRITISH COLUMBIA 1 0.026

UNIV NO COLORADO 1 0.026

UNIV NORTH BENGAL 1 0.026

UNIV NORTH CAROLINA CHAPEL HILL 1 0.026

UNIV NORTH CAROLINA HOSP 1 0.026

UNIV NOVA LISBOA 1 0.026

UNIV OCCUPAT ENVIRONM HLTH 1 0.026

UNIV OKLAHOMA HLTH SCI CTR 1 0.026

UNIV OKLAHORRIA HLTH SCI CTR 1 0.026

UNIV OSTRAVA 1 0.026

UNIV PADOVA HOSP 1 0.026

UNIV PALERMO 1 0.026

UNIV PANAMER 1 0.026

UNIV PANAMERICANA 1 0.026

UNIV PARIS 07 1 0.026

UNIV PARIS 11 1 0.026

UNIV PARIS SACLAY 1 0.026

UNIV PAUL SABATIER 1 0.026

UNIV PECS 1 0.026

UNIV PENN PERELMAN 1 0.026

UNIV PESHAWAR 1 0.026

UNIV PICARDIE JULES VERNE 1 0.026

UNIV PIEMONTE ORIENTALE 1 0.026

UNIV PIERRE MARIE CURIE PARIS 6 1 0.026

UNIV PUERTO RICO 1 0.026

UNIV QUEBEC 1 0.026

UNIV QUEBEC TROIS RIVIERES 1 0.026

UNIV QUEENSLAND BIOL RESOURCES 1 0.026

UNIV ROCHESTER 1 0.026

UNIV ROMA TOR VERGATA 1 0.026

UNIV ROVIRA VIRGILI 1 0.026

UNIV RWTH AACHEN 1 0.026

UNIV SALVADOR 1 0.026

UNIV SASKATOON 1 0.026

UNIV SASSARI 1 0.026

UNIV SCI PHILADELPHIA 1 0.026

UNIV SCI TECHNOL CHINA 1 0.026

UNIV SHANGHAI SCI TECHNOL 1 0.026

UNIV SHARJAH 1 0.026

UNIV SIENA 1 0.026

UNIV SOUTHAMPTON 1 0.026

UNIV STRASBOURG 1 0.026

UNIV SUSSEX 1 0.026

UNIV SWABI 1 0.026

UNIV TABRIZ 1 0.026

UNIV TABUK KINGDOM SAUDI ARABIA 1 0.026

UNIV TAMPERE 1 0.026

UNIV TEHRAN 1 0.026

UNIV TEHRAN MED SCI 1 0.026

UNIV TEXAS HLTH SAN ANTONIO 1 0.026

UNIV TEXAS MED CTR 1 0.026

UNIV TEXAS SAN ANTONIO 1 0.026

UNIV THESSALY 1 0.026

UNIV TOLIMA 1 0.026

UNIV TORINO 1 0.026

UNIV TOULOUSE III PAUL SABATIER TOULOUSE 1 0.026

UNIV TRASOS MONTES 1 0.026

UNIV TRISOS MONTES ALTO DOURO 1 0.026

UNIV TROMSO 1 0.026

UNIV UDINE 1 0.026

UNIV ULM 1 0.026

UNIV ULSAN 1 0.026

UNIV ULSTER 1 0.026

UNIV UPPSALA 1 0.026

UNIV UTAH HLTH SCI 1 0.026

UNIV VALENCIA 1 0.026

UNIV VERACRUZANA 1 0.026

UNIV VERSAILLES 1 0.026

UNIV VERSAILLES ST QUENTIN 1 0.026

UNIV VIENNA 1 0.026

UNIV VITA SALUTE SAN RAFFAELE 1 0.026

UNIV WARSAW 1 0.026

UNIV WEST INDIES 1 0.026

UNIV WESTERN SYDNEY 1 0.026

UNIV WITTEN HERDECKE 1 0.026

UNIV WITWATERSRAND 1 0.026

UNIV ZAGREB 1 0.026

URGO GRP 1 0.026

URMIA UNIV 1 0.026

UROL INST NORTHEASTERN NEW YORK 1 0.026

US FDA 1 0.026

USDA ARS 1 0.026

USP 1 0.026

USTHB 1 0.026

UT HLTH SCI CTR SAN ANTONIO 1 0.026

UTAH CTR ADV IMAGING RES 1 0.026

UTH SAN ANTONIO 1 0.026

UTRECHT INST PHATRMACEUT SCI 1 0.026

UTRECHT MED CTR 1 0.026

VA HLTH CARE SYST 1 0.026

VA PITTSURGH HLTH SYST 1 0.026

VA PUGET SOUND HLTH CARE SYST 1 0.026

VA SALT LAKE CITY HLTH CARE SYST 1 0.026

VA SAN DIEGO HLTH CARE 1 0.026

VA ST LOUIS HLTH CARE SYST 1 0.026

VAINCRE MUCOVISCIDOSE 1 0.026

VALL DHEBRON HOSP 1 0.026

VALL DHEBRON INST RECERCA 1 0.026

VANDERBILT CTR KIDNEY DIS 1 0.026

VENICE CITY HOSP 1 0.026

VERONA UNIV HOSP 1 0.026

VET ADM 1 0.026

VET ADM RES GERIATR RES EDUC CLIN CTR 1 0.026

VET AFFAIRS HOSP 1 0.026

VET GEN HOSP 1 0.026

VET HEALTHCARE SYST 1 0.026

VET MED RES FDN 1 0.026

VETAGRO SUP 1 0.026

VIB 1 0.026

VIBORG KJELLERUP CTY HOSP 1 0.026

VICHEM CHEM RES LTD 1 0.026

VICTOR BABES INST PATHOL 1 0.026

VICTOR BABES UNIV MED PHARM TIMISOARA 1 0.026

VILLA BIANCA HOSP 1 0.026

VIROMED CO LTD 1 0.026

VRIJE UNIV MED CTR 1 0.026

VRIJE UNIV MED CTR AMSTERDAM 1 0.026

VU UNIV MED CTR AMSTERDAM 1 0.026

W LOS ANGELES VET AFFAIRS MED CTR 1 0.026

W SIDE VET ADM MED CTR 1 0.026

W VIRGINIA UNIV 1 0.026

WAKE FOREST SCH MED 1 0.026

WAKISAKA NAIKA WAKISAKA INTERNAL MED CLIN 1 0.026

WAM TEACHING HOSP 1 0.026

WANNA MED COLL 1 0.026

WARNER LAMBERT INC 1 0.026

WARTBURG COLL 1 0.026

WARWICK MED SCH 1 0.026

WCMC 1 0.026

WEI GONG MEM HOSP 1 0.026

WEIFANG CTR DIS CONTROL PREVENT 1 0.026

WEIFANG MED UNIV 1 0.026

WEIZMANN INST SCI 1 0.026

WENZHOU MED UNIVERSITG 1 0.026

WEST CHINA HLTH CARE HOSP 1 0.026

WEST VIRGINIA UNIV 1 0.026

WESTERN MELBOURNE HOSP 1 0.026

WHO COLLABORATING CTR NONCOMMUNICABLE DIS PREVENT 1 0.026

WILLIAM BEAUMONT HOSP 1 0.026

WILLIAM JENNINGS BRYAN DORN VET AFFAIRS MED CTR 1 0.026

WOMEN CHILDRENS HOSP 1 0.026

WOMENS COLL HOSP 1 0.026

WORCESTERSHIRE ROYAL HOSP 1 0.026

WUHAN 1 HOSP 1 0.026

WUHAN CHILDRENS HOSP 1 0.026

WUHAN HAMILTON BIOTECHNOL CO LTD 1 0.026

WUHAN INST BIOTECHNOL 1 0.026

WUHAN UNIV SCI TECHNOL 1 0.026

WUHAN WUCHANG HOSP 1 0.026

WUZHOU PHARMACEUT GRP CO LTD 1 0.026

WYNN DOMAIN BAKER HEART RES INST 1 0.026

XAVIER BICHAT UNIV MED 1 0.026

XCENDA GMBH 1 0.026

XIAN 4 HOSP 1 0.026

XIAN THIRD HOSP 1 0.026

XINXIANG MED UNIV 1 0.026

XISHUANGBANNA TROP BOT GARDEN 1 0.026

XOMA CORP 1 0.026

XUCHANG INST FOOD DRUG CONTROL 1 0.026

XUZHOU CTR DIS CONTROL PREVENT 1 0.026

YALE NEW HAVEN MED CTR 1 0.026

YAMANASHI KOSEI HOSP 1 0.026

YANBIAN UNIV MED COLL 1 0.026

YANCHENG CITY 1 PEOPLES HOSP 1 0.026

YANGZI RIVER PHARMACEUT GRP CO LTD 1 0.026

YANTAI UNIV 1 0.026

YANTAISHAN HOSP 1 0.026

YARMOUK UNIV 1 0.026

YENIMAHALLE STATE HOSP 1 0.026

YEOUIDO ST MARYS HOSP 1 0.026

YIJISHAN HOSP 1 0.026

YILDIRIM BEYAZIT UNIV 1 0.026

YOKOHAMA INST 1 0.026

YOKOHAMA ROSAI HOSP 1 0.026

YONSEI UNIV HLTH SYST 1 0.026

YOUJIANG MED UNIV NATIONALITIES 1 0.026

YUE BEI PEOPLES HOSP 1 0.026

YUHUANGDING HOSP YANTAI CITY 1 0.026

YUNNAN AGR UNIV 1 0.026

YUZUNCU YIL UNIV 1 0.026

ZAOZHUANG MUNICIPAL HOSP 1 0.026

ZHANGQIU PEOPLES HOSP 1 0.026

ZHEJIANG ACAD TRADIT CHINESE MED 1 0.026

ZHEJIANG CONBA PHARMACEUT CO LTD 1 0.026

ZHEJIANG MARINE DEV RES INST 1 0.026

ZHEJIANG MED COLL 1 0.026

ZHEJIANG PHARMACEUT COLL 1 0.026

ZHEJIANG PROV PEOPLES HOSP 1 0.026

ZHEJIANG STYX MED MANAGEMENT CO LTD 1 0.026

ZHEJIANG UNIV TECHNOL 1 0.026

ZHENHAI PEOPLES HOSP NINGBO CITY 1 0.026

ZHUJI PEOPLE HOSP 1 0.026

ZHUMADIAN CENT HOSP 1 0.026

ZHUMADIAN CITY CTR HOSP 1 0.026

ZIEKENHUIS GRP TWENTE 1 0.026

ZIGONG FOURTH PEOPLES HOSP 1 0.026

ZUCERO THERAPEUT 1 0.026

ZUNYI MED COLL 1 0.026

ZUNYI MED PHARMACEUT COLL 1 0.026

(3 records (0.079%) do not contain data in the field being analyzed.)
