# Supplementary material for: Bibliometric Analysis of Renal Fibrosis in Diabetic Kidney Disease From 1985 to 2020
Source: Front Public Health. 2022 Feb 4;10:767591. doi: 10.3389/fpubh.2022.767591 (PMC8855938; doi:10.3389/fpubh.2022.767591)
Supplement: Supplementary file 3 [file Table_3.DOCX]

Source Titles records % of 3821

AMERICAN JOURNAL OF PHYSIOLOGY RENAL PHYSIOLOGY 169 4.423

KIDNEY INTERNATIONAL 166 4.344

JOURNAL OF THE AMERICAN SOCIETY OF NEPHROLOGY 132 3.455

PLOS ONE 104 2.722

NEPHROLOGY DIALYSIS TRANSPLANTATION 91 2.382

SCIENTIFIC REPORTS 74 1.937

DIABETES 53 1.387

INTERNATIONAL JOURNAL OF MOLECULAR SCIENCES 42 1.099

DIABETOLOGIA 40 1.047

CURRENT OPINION IN NEPHROLOGY AND HYPERTENSION 38 0.995

AMERICAN JOURNAL OF NEPHROLOGY 37 0.968

AMERICAN JOURNAL OF PATHOLOGY 37 0.968

NEPHROLOGY 37 0.968

BIOCHEMICAL AND BIOPHYSICAL RESEARCH COMMUNICATIONS 36 0.942

JOURNAL OF DIABETES RESEARCH 36 0.942

BIOMEDICINE PHARMACOTHERAPY 34 0.890

MOLECULAR MEDICINE REPORTS 34 0.890

RENAL FAILURE 34 0.890

FRONTIERS IN PHARMACOLOGY 30 0.785

KIDNEY BLOOD PRESSURE RESEARCH 29 0.759

CLINICAL SCIENCE 28 0.733

NATURE REVIEWS NEPHROLOGY 26 0.680

CLINICAL JOURNAL OF THE AMERICAN SOCIETY OF NEPHROLOGY 25 0.654

FASEB JOURNAL 25 0.654

HYPERTENSION 24 0.628

MOLECULAR AND CELLULAR ENDOCRINOLOGY 24 0.628

TRANSPLANTATION PROCEEDINGS 24 0.628

AMERICAN JOURNAL OF KIDNEY DISEASES 23 0.602

LABORATORY INVESTIGATION 22 0.576

EUROPEAN JOURNAL OF PHARMACOLOGY 21 0.550

JOURNAL OF CELLULAR AND MOLECULAR MEDICINE 20 0.523

FRONTIERS IN PHYSIOLOGY 19 0.497

JOURNAL OF BIOLOGICAL CHEMISTRY 19 0.497

NEPHRON 19 0.497

PHARMACOLOGICAL RESEARCH 19 0.497

CLINICAL AND EXPERIMENTAL NEPHROLOGY 18 0.471

INTERNATIONAL JOURNAL OF CLINICAL AND EXPERIMENTAL MEDICINE 18 0.471

JOURNAL OF CELLULAR PHYSIOLOGY 18 0.471

JOURNAL OF HYPERTENSION 18 0.471

BIOCHIMICA ET BIOPHYSICA ACTA MOLECULAR BASIS OF DISEASE 17 0.445

DIABETES RESEARCH AND CLINICAL PRACTICE 17 0.445

EVIDENCE BASED COMPLEMENTARY AND ALTERNATIVE MEDICINE 17 0.445

EXPERIMENTAL AND THERAPEUTIC MEDICINE 17 0.445

JOURNAL OF NEPHROLOGY 17 0.445

AMERICAN JOURNAL OF TRANSLATIONAL RESEARCH 16 0.419

BIOMED RESEARCH INTERNATIONAL 16 0.419

BMC NEPHROLOGY 16 0.419

CHINESE MEDICAL JOURNAL 16 0.419

CONTRIBUTIONS TO NEPHROLOGY 16 0.419

JOURNAL OF DIABETES AND ITS COMPLICATIONS 16 0.419

JOURNAL OF ETHNOPHARMACOLOGY 16 0.419

LIFE SCIENCES 16 0.419

SEMINARS IN NEPHROLOGY 16 0.419

CELLULAR PHYSIOLOGY AND BIOCHEMISTRY 15 0.393

CLINICAL AND EXPERIMENTAL PHARMACOLOGY AND PHYSIOLOGY 15 0.393

FREE RADICAL BIOLOGY AND MEDICINE 15 0.393

NEPHRON EXPERIMENTAL NEPHROLOGY 15 0.393

TRANSPLANTATION 15 0.393

ANTIOXIDANTS REDOX SIGNALING 14 0.366

BRITISH JOURNAL OF PHARMACOLOGY 14 0.366

CLINICAL NEPHROLOGY 14 0.366

EUROPEAN REVIEW FOR MEDICAL AND PHARMACOLOGICAL SCIENCES 14 0.366

JOURNAL OF CELLULAR BIOCHEMISTRY 14 0.366

PEDIATRIC NEPHROLOGY 14 0.366

ACTA PHARMACOLOGICA SINICA 13 0.340

CELL DEATH DISEASE 13 0.340

DIABETES CARE 13 0.340

DRUG DESIGN DEVELOPMENT AND THERAPY 13 0.340

JOURNAL OF PHARMACOLOGICAL SCIENCES 13 0.340

METABOLISM CLINICAL AND EXPERIMENTAL 13 0.340

EXPERIMENTAL BIOLOGY AND MEDICINE 12 0.314

INTERNATIONAL JOURNAL OF BIOCHEMISTRY CELL BIOLOGY 12 0.314

INTERNATIONAL JOURNAL OF CLINICAL AND EXPERIMENTAL PATHOLOGY 12 0.314

INTERNATIONAL JOURNAL OF MOLECULAR MEDICINE 12 0.314

INTERNATIONAL UROLOGY AND NEPHROLOGY 12 0.314

JOURNAL OF PATHOLOGY 12 0.314

ONCOTARGET 12 0.314

PHYTOMEDICINE 12 0.314

AMERICAN JOURNAL OF TRANSPLANTATION 11 0.288

CLINICAL TRANSPLANTATION 11 0.288

CURRENT HYPERTENSION REPORTS 11 0.288

ADVANCES IN EXPERIMENTAL MEDICINE AND BIOLOGY 10 0.262

AMERICAN JOURNAL OF PHYSIOLOGY ENDOCRINOLOGY AND METABOLISM 10 0.262

BMC COMPLEMENTARY AND ALTERNATIVE MEDICINE 10 0.262

CURRENT DIABETES REPORTS 10 0.262

JOURNAL OF ENDOCRINOLOGY 10 0.262

OXIDATIVE MEDICINE AND CELLULAR LONGEVITY 10 0.262

PHARMACOLOGY 10 0.262

ENDOCRINOLOGY 9 0.236

EXPERIMENTAL CELL RESEARCH 9 0.236

JOURNAL OF FUNCTIONAL FOODS 9 0.236

JOURNAL OF PHARMACOLOGY AND EXPERIMENTAL THERAPEUTICS 9 0.236

JOURNAL OF THE RENIN ANGIOTENSIN ALDOSTERONE SYSTEM 9 0.236

MEDICAL SCIENCE MONITOR 9 0.236

MOLECULAR AND CELLULAR BIOCHEMISTRY 9 0.236

NUTRIENTS 9 0.236

TOXICOLOGY AND APPLIED PHARMACOLOGY 9 0.236

CELLS 8 0.209

DIABETES METABOLIC SYNDROME AND OBESITY TARGETS AND THERAPY 8 0.209

DIABETES METABOLISM 8 0.209

HISTOLOGY AND HISTOPATHOLOGY 8 0.209

JOURNAL OF CLINICAL INVESTIGATION 8 0.209

JOURNAL OF CLINICAL MEDICINE 8 0.209

JOURNAL OF MOLECULAR MEDICINE JMM 8 0.209

MATRIX BIOLOGY 8 0.209

NAUNYN SCHMIEDEBERGS ARCHIVES OF PHARMACOLOGY 8 0.209

RENAL FIBROSIS MECHANISMS AND THERAPIES 8 0.209

ADVANCES IN CHRONIC KIDNEY DISEASE 7 0.183

AGING US 7 0.183

AMERICAN JOURNAL OF HYPERTENSION 7 0.183

AMERICAN JOURNAL OF PHYSIOLOGY REGULATORY INTEGRATIVE AND COMPARATIVE PHYSIOLOGY 7 0.183

DIABETES METABOLISM RESEARCH AND REVIEWS 7 0.183

EUROPEAN JOURNAL OF HEART FAILURE 7 0.183

HYPERTENSION RESEARCH 7 0.183

JOURNAL OF CARDIOVASCULAR PHARMACOLOGY 7 0.183

JOURNAL OF THE AMERICAN HEART ASSOCIATION 7 0.183

KIDNEY INTERNATIONAL SUPPLEMENTS 7 0.183

MEDIATORS OF INFLAMMATION 7 0.183

MINERAL AND ELECTROLYTE METABOLISM 7 0.183

MOLECULES 7 0.183

NEPHRON PHYSIOLOGY 7 0.183

TRANSLATIONAL RESEARCH 7 0.183

ACTA MEDICA MEDITERRANEA 6 0.157

AMERICAN JOURNAL OF THE MEDICAL SCIENCES 6 0.157

CARDIOVASCULAR DIABETOLOGY 6 0.157

CELLULAR SIGNALLING 6 0.157

CHEMICO BIOLOGICAL INTERACTIONS 6 0.157

CURRENT PHARMACEUTICAL DESIGN 6 0.157

DIABETES AND THE KIDNEY 6 0.157

DIABETES OBESITY METABOLISM 6 0.157

DIABETOLOGY METABOLIC SYNDROME 6 0.157

ENDOCRINE 6 0.157

EXPERIMENTAL AND CLINICAL ENDOCRINOLOGY DIABETES 6 0.157

EXPERIMENTAL AND MOLECULAR PATHOLOGY 6 0.157

FRONTIERS IN IMMUNOLOGY 6 0.157

INTERNATIONAL IMMUNOPHARMACOLOGY 6 0.157

JOURNAL OF HISTOCHEMISTRY CYTOCHEMISTRY 6 0.157

JOURNAL OF RECEPTORS AND SIGNAL TRANSDUCTION 6 0.157

JOURNAL OF TRANSLATIONAL MEDICINE 6 0.157

JOURNAL OF UROLOGY 6 0.157

KIDNEY INTERNATIONAL REPORTS 6 0.157

NEPHRON CLINICAL PRACTICE 6 0.157

ACTA DIABETOLOGICA 5 0.131

ALIMENTARY PHARMACOLOGY THERAPEUTICS 5 0.131

AMERICAN JOURNAL OF CHINESE MEDICINE 5 0.131

AMERICAN JOURNAL OF PHYSIOLOGY CELL PHYSIOLOGY 5 0.131

ANNALS OF TRANSLATIONAL MEDICINE 5 0.131

BIOCHEMICAL JOURNAL 5 0.131

BIOLOGICAL PHARMACEUTICAL BULLETIN 5 0.131

BIOSCIENCE REPORTS 5 0.131

BMJ OPEN DIABETES RESEARCH CARE 5 0.131

CARDIOVASCULAR RESEARCH 5 0.131

CELL AND TISSUE RESEARCH 5 0.131

CELL CYCLE 5 0.131

CURRENT MEDICINAL CHEMISTRY 5 0.131

DIABETES METABOLISM JOURNAL 5 0.131

EXPERIMENTAL AND MOLECULAR MEDICINE 5 0.131

EXPERT OPINION ON INVESTIGATIONAL DRUGS 5 0.131

FOOD FUNCTION 5 0.131

FRONTIERS IN BIOSCIENCE LANDMARK 5 0.131

FRONTIERS IN CELL AND DEVELOPMENTAL BIOLOGY 5 0.131

INTERNAL MEDICINE 5 0.131

INTERNATIONAL JOURNAL OF ENDOCRINOLOGY 5 0.131

IRANIAN JOURNAL OF KIDNEY DISEASES 5 0.131

JOURNAL OF AGRICULTURAL AND FOOD CHEMISTRY 5 0.131

JOURNAL OF BIOLOGICAL REGULATORS AND HOMEOSTATIC AGENTS 5 0.131

JOURNAL OF CELL COMMUNICATION AND SIGNALING 5 0.131

JOURNAL OF PHYSIOLOGY AND BIOCHEMISTRY 5 0.131

LATIN AMERICAN JOURNAL OF PHARMACY 5 0.131

MEDICINE 5 0.131

PEDIATRIC DIABETES 5 0.131

PEPTIDES 5 0.131

PHARMACOLOGICAL REPORTS 5 0.131

PHYSIOLOGICAL GENOMICS 5 0.131

RSC ADVANCES 5 0.131

ACTA HISTOCHEMICA 4 0.105

AMERICAN JOURNAL OF CARDIOLOGY 4 0.105

ANNALS OF CLINICAL AND LABORATORY SCIENCE 4 0.105

BIOCHEMICAL PHARMACOLOGY 4 0.105

BIOMOLECULES 4 0.105

BLOOD PURIFICATION 4 0.105

CLINICA CHIMICA ACTA 4 0.105

CLINICAL RHEUMATOLOGY 4 0.105

CURRENT DRUG TARGETS 4 0.105

CYTOKINE 4 0.105

DIABETIC MEDICINE 4 0.105

EBIOMEDICINE 4 0.105

EXPERIMENTAL ANIMALS 4 0.105

EXPERIMENTAL PHYSIOLOGY 4 0.105

EXPERT OPINION ON DRUG DISCOVERY 4 0.105

EXPERT OPINION ON THERAPEUTIC TARGETS 4 0.105

FOOD AND CHEMICAL TOXICOLOGY 4 0.105

FREE RADICAL RESEARCH 4 0.105

FRONTIERS IN ENDOCRINOLOGY 4 0.105

HEART FAILURE REVIEWS 4 0.105

HEMODIALYSIS INTERNATIONAL 4 0.105

IMMUNOPHARMACOLOGY AND IMMUNOTOXICOLOGY 4 0.105

INTERNATIONAL JOURNAL OF BIOLOGICAL MACROMOLECULES 4 0.105

JCI INSIGHT 4 0.105

JOURNAL OF BIOMATERIALS AND TISSUE ENGINEERING 4 0.105

JOURNAL OF CYSTIC FIBROSIS 4 0.105

JOURNAL OF DIABETES 4 0.105

JOURNAL OF DIABETES INVESTIGATION 4 0.105

JOURNAL OF HUAZHONG UNIVERSITY OF SCIENCE AND TECHNOLOGY MEDICAL SCIENCES 4 0.105

JOURNAL OF NUTRITIONAL BIOCHEMISTRY 4 0.105

JOURNAL OF PHYSIOLOGY LONDON 4 0.105

LIVER INTERNATIONAL 4 0.105

MODERN PATHOLOGY 4 0.105

MOLECULAR THERAPY NUCLEIC ACIDS 4 0.105

NATURE CLINICAL PRACTICE NEPHROLOGY 4 0.105

NATURE COMMUNICATIONS 4 0.105

PEERJ 4 0.105

PHYTOTHERAPY RESEARCH 4 0.105

PROCEEDINGS OF THE NATIONAL ACADEMY OF SCIENCES OF THE UNITED STATES OF AMERICA 4 0.105

SEMINARS IN DIALYSIS 4 0.105

ACTA PHYSIOLOGICA 3 0.079

AGING CELL 3 0.079

AMERICAN JOURNAL OF GASTROENTEROLOGY 3 0.079

AMERICAN JOURNAL OF PHYSIOLOGY 3 0.079

AMERICAN JOURNAL OF ROENTGENOLOGY 3 0.079

ANTIOXIDANTS 3 0.079

ARCHIVES OF BIOCHEMISTRY AND BIOPHYSICS 3 0.079

ARCHIVES OF PATHOLOGY LABORATORY MEDICINE 3 0.079

ARCHIVES OF PHARMACAL RESEARCH 3 0.079

BIOCHIMICA ET BIOPHYSICA ACTA MOLECULAR AND CELL BIOLOGY OF LIPIDS 3 0.079

BIOCHIMICA ET BIOPHYSICA ACTA MOLECULAR CELL RESEARCH 3 0.079

BIOCHIMIE 3 0.079

BIOMEDICAL RESEARCH INDIA 3 0.079

BIOORGANIC MEDICINAL CHEMISTRY LETTERS 3 0.079

CARDIORENAL MEDICINE 3 0.079

CARDIOVASCULAR DRUGS AND THERAPY 3 0.079

CELL BIOLOGY INTERNATIONAL 3 0.079

CIRCULATION 3 0.079

CLINICAL BIOCHEMISTRY 3 0.079

CLINICAL KIDNEY JOURNAL 3 0.079

CYTOKINE GROWTH FACTOR REVIEWS 3 0.079

DIGESTIVE DISEASES AND SCIENCES 3 0.079

DISEASE MODELS MECHANISMS 3 0.079

DNA AND CELL BIOLOGY 3 0.079

ENDOTHELIN IN RENAL PHYSIOLOGY AND DISEASE 3 0.079

EUROPEAN JOURNAL OF NUTRITION 3 0.079

EUROPEAN RESPIRATORY JOURNAL 3 0.079

EXPERIMENTAL DIABETES RESEARCH 3 0.079

EXPERIMENTAL NEPHROLOGY 3 0.079

GENE 3 0.079

HORMONE AND METABOLIC RESEARCH 3 0.079

HUMAN PATHOLOGY 3 0.079

INTERNATIONAL JOURNAL OF BIOLOGICAL SCIENCES 3 0.079

INTERNATIONAL JOURNAL OF NANOMEDICINE 3 0.079

JOURNAL OF DRUG TARGETING 3 0.079

JOURNAL OF FOOD AND DRUG ANALYSIS 3 0.079

JOURNAL OF HEART AND LUNG TRANSPLANTATION 3 0.079

JOURNAL OF MOLECULAR AND CELLULAR CARDIOLOGY 3 0.079

JOURNAL OF MOLECULAR ENDOCRINOLOGY 3 0.079

JOURNAL OF PHARMACOLOGICAL AND TOXICOLOGICAL METHODS 3 0.079

JOURNAL OF THE AMERICAN COLLEGE OF CARDIOLOGY 3 0.079

JOURNAL OF VIRAL HEPATITIS 3 0.079

KIDNEY DISEASES 3 0.079

MARINE DRUGS 3 0.079

MEDICAL HYPOTHESES 3 0.079

MOLECULAR BIOLOGY REPORTS 3 0.079

MOLECULAR METABOLISM 3 0.079

MOLECULAR NUTRITION FOOD RESEARCH 3 0.079

PATHOLOGY RESEARCH AND PRACTICE 3 0.079

PHARMACOLOGY THERAPEUTICS 3 0.079

PHARMAZIE 3 0.079

PROTEOMICS 3 0.079

REDOX BIOLOGY 3 0.079

SCANDINAVIAN JOURNAL OF UROLOGY AND NEPHROLOGY 3 0.079

TRANSPLANT INFECTIOUS DISEASE 3 0.079

TROPICAL JOURNAL OF PHARMACEUTICAL RESEARCH 3 0.079

WIENER KLINISCHE WOCHENSCHRIFT 3 0.079

WORLD JOURNAL OF DIABETES 3 0.079

WORLD JOURNAL OF GASTROENTEROLOGY 3 0.079

ACTA PHYSIOLOGICA HUNGARICA 2 0.052

AMERICAN JOURNAL OF HEALTH SYSTEM PHARMACY 2 0.052

AMERICAN JOURNAL OF MEDICAL GENETICS PART A 2 0.052

ANNALS OF TRANSPLANTATION 2 0.052

ARCHIVES OF MEDICAL RESEARCH 2 0.052

BASIC CLINICAL PHARMACOLOGY TOXICOLOGY 2 0.052

BASIC RESEARCH IN CARDIOLOGY 2 0.052

BIOCELL 2 0.052

BIOCHEMICAL SOCIETY TRANSACTIONS 2 0.052

BIOCHIMICA ET BIOPHYSICA ACTA GENE REGULATORY MECHANISMS 2 0.052

BIOCHIMICA ET BIOPHYSICA ACTA GENERAL SUBJECTS 2 0.052

BIOLOGICAL RESEARCH 2 0.052

BIOMEDICINES 2 0.052

BIOSCIENCE BIOTECHNOLOGY AND BIOCHEMISTRY 2 0.052

BIOSCIENCE TRENDS 2 0.052

BMC MEDICAL GENETICS 2 0.052

BRITISH JOURNAL OF CLINICAL PHARMACOLOGY 2 0.052

CANADIAN JOURNAL OF DIABETES 2 0.052

CANADIAN JOURNAL OF PHYSIOLOGY AND PHARMACOLOGY 2 0.052

CELL REPORTS 2 0.052

CELLS TISSUES ORGANS 2 0.052

CHEST 2 0.052

CHINESE JOURNAL OF NATURAL MEDICINES 2 0.052

CIRCULATION HEART FAILURE 2 0.052

COCHRANE DATABASE OF SYSTEMATIC REVIEWS 2 0.052

COMPARATIVE MEDICINE 2 0.052

CORONARY ARTERY DISEASE 2 0.052

CRITICAL CARE 2 0.052

CRITICAL REVIEWS IN CLINICAL LABORATORY SCIENCES 2 0.052

CURRENT OPINION IN INVESTIGATIONAL DRUGS 2 0.052

CURRENT OPINION IN ORGAN TRANSPLANTATION 2 0.052

CURRENT OPINION IN PHARMACOLOGY 2 0.052

CURRENT VASCULAR PHARMACOLOGY 2 0.052

DIABETES VASCULAR DISEASE RESEARCH 2 0.052

DIGESTIVE DISEASES 2 0.052

DISCOVERY MEDICINE 2 0.052

DISEASE MARKERS 2 0.052

DRUG DEVELOPMENT RESEARCH 2 0.052

ENDOCRINE JOURNAL 2 0.052

ENDOCRINE PRACTICE 2 0.052

ENDOCRINE RESEARCH 2 0.052

EUROPEAN JOURNAL OF CLINICAL INVESTIGATION 2 0.052

EUROPEAN JOURNAL OF HUMAN GENETICS 2 0.052

EXPERIMENTAL AND CLINICAL TRANSPLANTATION 2 0.052

EXPERIMENTAL MODELS FOR RENAL DISEASES PATHOGENESIS AND DIAGNOSIS 2 0.052

EXPERT OPINION ON ORPHAN DRUGS 2 0.052

EXPERT OPINION ON PHARMACOTHERAPY 2 0.052

EXPERT REVIEW OF RESPIRATORY MEDICINE 2 0.052

FUNDAMENTAL CLINICAL PHARMACOLOGY 2 0.052

GENETICS AND MOLECULAR RESEARCH 2 0.052

HEPATOLOGY 2 0.052

HUMAN CELL 2 0.052

HUMAN EXPERIMENTAL TOXICOLOGY 2 0.052

HUMAN GENE THERAPY 2 0.052

INFECTIOUS DISEASE CLINICS OF NORTH AMERICA 2 0.052

INFLAMMATION 2 0.052

INTERNAL MEDICINE JOURNAL 2 0.052

INTERNATIONAL JOURNAL OF CARDIOLOGY 2 0.052

INTERNATIONAL JOURNAL OF EXPERIMENTAL PATHOLOGY 2 0.052

INTERNATIONAL JOURNAL OF MEDICAL SCIENCES 2 0.052

INTERNATIONAL JOURNAL OF PHARMACOLOGY 2 0.052

JAMA CARDIOLOGY 2 0.052

JOURNAL OF CARDIAC FAILURE 2 0.052

JOURNAL OF CARDIOVASCULAR MAGNETIC RESONANCE 2 0.052

JOURNAL OF CELL SCIENCE 2 0.052

JOURNAL OF CLINICAL ENDOCRINOLOGY METABOLISM 2 0.052

JOURNAL OF CLINICAL PATHOLOGY 2 0.052

JOURNAL OF GASTROENTEROLOGY AND HEPATOLOGY 2 0.052

JOURNAL OF HEPATOLOGY 2 0.052

JOURNAL OF IMMUNOLOGY RESEARCH 2 0.052

JOURNAL OF INTERNATIONAL MEDICAL RESEARCH 2 0.052

JOURNAL OF INVESTIGATIVE MEDICINE 2 0.052

JOURNAL OF MOLECULAR CELL BIOLOGY 2 0.052

JOURNAL OF PHARMACY AND PHARMACOLOGY 2 0.052

JOURNAL OF TRADITIONAL CHINESE MEDICINE 2 0.052

JOURNAL OF VASCULAR SURGERY 2 0.052

JOURNAL OF ZHEJIANG UNIVERSITY SCIENCE B 2 0.052

KAOHSIUNG JOURNAL OF MEDICAL SCIENCES 2 0.052

KIDNEY RESEARCH AND CLINICAL PRACTICE 2 0.052

LIFE BASEL 2 0.052

MAGNETIC RESONANCE IN MEDICINE 2 0.052

MOLECULAR ASPECTS OF MEDICINE 2 0.052

MOLECULAR ENDOCRINOLOGY 2 0.052

MOLECULAR THERAPY 2 0.052

NEW ENGLAND JOURNAL OF MEDICINE 2 0.052

NITRIC OXIDE BIOLOGY AND CHEMISTRY 2 0.052

PATHOLOGY 2 0.052

PATHOLOGY INTERNATIONAL 2 0.052

PERITONEAL DIALYSIS INTERNATIONAL 2 0.052

PROGRESS IN MOLECULAR BIOLOGY AND TRANSLATIONAL SCIENCE 2 0.052

PROSTAGLANDINS OTHER LIPID MEDIATORS 2 0.052

PROTEIN AND PEPTIDE LETTERS 2 0.052

RNA BIOLOGY 2 0.052

ROMANIAN JOURNAL OF MORPHOLOGY AND EMBRYOLOGY 2 0.052

SCANDINAVIAN JOURNAL OF CLINICAL LABORATORY INVESTIGATION 2 0.052

SCANDINAVIAN JOURNAL OF GASTROENTEROLOGY 2 0.052

SCIENCE SIGNALING 2 0.052

STEM CELL RESEARCH THERAPY 2 0.052

THERAPEUTIC ADVANCES IN ENDOCRINOLOGY AND METABOLISM 2 0.052

TOHOKU JOURNAL OF EXPERIMENTAL MEDICINE 2 0.052

TOXICOLOGICAL SCIENCES 2 0.052

TOXICOLOGY 2 0.052

TRANSPLANT IMMUNOLOGY 2 0.052

ULTRASOUND IN MEDICINE AND BIOLOGY 2 0.052

VASCULAR PHARMACOLOGY 2 0.052

VIRCHOWS ARCHIV 2 0.052

ABDOMINAL RADIOLOGY 1 0.026

ACS OMEGA 1 0.026

ACTA BIOCHIMICA POLONICA 1 0.026

ACTA CARDIOLOGICA 1 0.026

ACTA CLINICA CROATICA 1 0.026

ACTA PATHOLOGICA JAPONICA 1 0.026

ACTA PHARMACEUTICA SINICA B 1 0.026

ADVANCES IN CARDIOLOGY 1 0.026

ADVANCES IN CLINICAL CHEMISTRY 1 0.026

ADVANCES IN CLINICAL CHEMISTRY VOL 82 1 0.026

ADVANCES IN NUTRITION 1 0.026

AFRICAN JOURNAL OF TRADITIONAL COMPLEMENTARY AND ALTERNATIVE MEDICINES 1 0.026

AGEING RESEARCH REVIEWS 1 0.026

AMERICAN FAMILY PHYSICIAN 1 0.026

AMERICAN HEART JOURNAL 1 0.026

AMERICAN JOURNAL OF EPIDEMIOLOGY 1 0.026

AMERICAN JOURNAL OF HEMATOLOGY 1 0.026

AMERICAN JOURNAL OF HUMAN GENETICS 1 0.026

AMERICAN JOURNAL OF MEDICAL GENETICS 1 0.026

AMERICAN JOURNAL OF MEDICINE 1 0.026

AMERICAN JOURNAL OF OBSTETRICS AND GYNECOLOGY 1 0.026

AMERICAN JOURNAL OF PHYSIOLOGY HEART AND CIRCULATORY PHYSIOLOGY 1 0.026

AMERICAN JOURNAL OF PHYSIOLOGY RENAL FLUID AND ELECTROLYTE PHYSIOLOGY 1 0.026

AMERICAN JOURNAL OF RESPIRATORY AND CRITICAL CARE MEDICINE 1 0.026

ANATOLIAN JOURNAL OF CARDIOLOGY 1 0.026

ANATOMICAL RECORD ADVANCES IN INTEGRATIVE ANATOMY AND EVOLUTIONARY BIOLOGY 1 0.026

ANATOMICAL SCIENCE INTERNATIONAL 1 0.026

ANGIOGENESIS 1 0.026

ANNALS ACADEMY OF MEDICINE SINGAPORE 1 0.026

ANNALS OF HEPATOLOGY 1 0.026

ANNALS OF MEDICINE 1 0.026

ANNALS OF PHARMACOTHERAPY 1 0.026

ANNALS OF THE NEW YORK ACADEMY OF SCIENCES 1 0.026

ANNALS OF THE RHEUMATIC DISEASES 1 0.026

ANNALS OF VASCULAR SURGERY 1 0.026

ANNUAL REPORTS IN MEDICINAL CHEMISTRY 1 0.026

ANNUAL REVIEW OF MEDICINE 1 0.026

ANNUAL REVIEW OF PATHOLOGY MECHANISMS OF DISEASE 1 0.026

ANNUAL REVIEW OF PATHOLOGY MECHANISMS OF DISEASE VOL 6 1 0.026

ANTIMICROBIAL AGENTS AND CHEMOTHERAPY 1 0.026

APPLIED BIOCHEMISTRY AND BIOTECHNOLOGY 1 0.026

APPLIED PHYSIOLOGY NUTRITION AND METABOLISM 1 0.026

AQUAPORIN REGULATION 1 0.026

ARCHIVES OF DISEASE IN CHILDHOOD 1 0.026

ARCHIVES OF GERONTOLOGY AND GERIATRICS 1 0.026

ARCHIVES OF INTERNAL MEDICINE 1 0.026

ARCHIVES OF IRANIAN MEDICINE 1 0.026

ARCHIVUM IMMUNOLOGIAE ET THERAPIAE EXPERIMENTALIS 1 0.026

ARTERIOSCLEROSIS THROMBOSIS AND VASCULAR BIOLOGY 1 0.026

ARTHRITIS AND RHEUMATISM 1 0.026

ARTIFICIAL CELLS NANOMEDICINE AND BIOTECHNOLOGY 1 0.026

ASIAN PACIFIC JOURNAL OF TROPICAL MEDICINE 1 0.026

ATHEROSCLEROSIS 1 0.026

ATHEROSCLEROSIS LARGE ARTERIES AND CARDIOVASCULAR RISK 1 0.026

AUTOIMMUNITY 1 0.026

AUTOIMMUNITY REVIEWS 1 0.026

AUTOPHAGY 1 0.026

BEST PRACTICE RESEARCH CLINICAL ENDOCRINOLOGY METABOLISM 1 0.026

BIOCHEMISTRY AND CELL BIOLOGY BIOCHIMIE ET BIOLOGIE CELLULAIRE 1 0.026

BIOCHEMISTRY MOSCOW 1 0.026

BIOCHIMICA ET BIOPHYSICA ACTA PROTEINS AND PROTEOMICS 1 0.026

BIOLOGICAL TRACE ELEMENT RESEARCH 1 0.026

BIOLOGY OF BLOOD AND MARROW TRANSPLANTATION 1 0.026

BIOLOGY OF SEX DIFFERENCES 1 0.026

BIOLOGY OF THE CELL 1 0.026

BIOLOGY OPEN 1 0.026

BIOMARKERS 1 0.026

BIOMARKERS IN MEDICINE 1 0.026

BIOMEDICAL CHROMATOGRAPHY 1 0.026

BIOMEDICAL JOURNAL 1 0.026

BIOMEDICAL RESEARCH TOKYO 1 0.026

BIOMOLECULES THERAPEUTICS 1 0.026

BIOORGANIC MEDICINAL CHEMISTRY 1 0.026

BMC BIOTECHNOLOGY 1 0.026

BMC CARDIOVASCULAR DISORDERS 1 0.026

BMC ENDOCRINE DISORDERS 1 0.026

BMC GASTROENTEROLOGY 1 0.026

BMC MEDICAL GENOMICS 1 0.026

BMC PHARMACOLOGY TOXICOLOGY 1 0.026

BMJ OPEN 1 0.026

BONE 1 0.026

BOSNIAN JOURNAL OF BASIC MEDICAL SCIENCES 1 0.026

BRATISLAVA MEDICAL JOURNAL BRATISLAVSKE LEKARSKE LISTY 1 0.026

BRAZILIAN JOURNAL OF MEDICAL AND BIOLOGICAL RESEARCH 1 0.026

BRITISH HEART JOURNAL 1 0.026

BRITISH JOURNAL OF DERMATOLOGY 1 0.026

BRITISH JOURNAL OF SURGERY 1 0.026

CANADIAN JOURNAL OF CARDIOLOGY 1 0.026

CANADIAN JOURNAL OF OPHTHALMOLOGY JOURNAL CANADIEN D OPHTALMOLOGIE 1 0.026

CARDIOLOGY 1 0.026

CARDIOLOGY JOURNAL 1 0.026

CARDIOVASCULAR THERAPEUTICS 1 0.026

CATHETERIZATION AND CARDIOVASCULAR INTERVENTIONS 1 0.026

CELL BIOCHEMISTRY AND BIOPHYSICS 1 0.026

CELL BIOCHEMISTRY AND FUNCTION 1 0.026

CELL BIOLOGY AND TOXICOLOGY 1 0.026

CELL CALCIUM 1 0.026

CELL DEATH DISCOVERY 1 0.026

CELL STRESS CHAPERONES 1 0.026

CELL TRANSPLANTATION 1 0.026

CELLULAR AND MOLECULAR BIOLOGY 1 0.026

CHEMICAL PHARMACEUTICAL BULLETIN 1 0.026

CHEMOSPHERE 1 0.026

CHINESE JOURNAL OF PHYSIOLOGY 1 0.026

CHINESE MEDICINE 1 0.026

CIBA FOUNDATION SYMPOSIA 1 0.026

CIRCULATION ARRHYTHMIA AND ELECTROPHYSIOLOGY 1 0.026

CIRCULATION JOURNAL 1 0.026

CIRCULATION RESEARCH 1 0.026

CLINICAL AND EXPERIMENTAL MEDICINE 1 0.026

CLINICAL AND INVESTIGATIVE MEDICINE 1 0.026

CLINICAL CARDIOLOGY 1 0.026

CLINICAL CHEMISTRY 1 0.026

CLINICAL CHEMISTRY AND LABORATORY MEDICINE 1 0.026

CLINICAL DYSMORPHOLOGY 1 0.026

CLINICAL GASTROENTEROLOGY AND HEPATOLOGY 1 0.026

CLINICAL IMMUNOLOGY 1 0.026

CLINICAL LABORATORY 1 0.026

CLINICAL NUTRITION 1 0.026

CLINICAL RADIOLOGY 1 0.026

CLINICS 1 0.026

CLINICS AND RESEARCH IN HEPATOLOGY AND GASTROENTEROLOGY 1 0.026

CLINICS IN PODIATRIC MEDICINE AND SURGERY 1 0.026

COMBINATORIAL CHEMISTRY HIGH THROUGHPUT SCREENING 1 0.026

COMPREHENSIVE PHYSIOLOGY 1 0.026

COMPUTATIONAL BIOLOGY AND CHEMISTRY 1 0.026

CURRENT ATHEROSCLEROSIS REPORTS 1 0.026

CURRENT GENE THERAPY 1 0.026

CURRENT GENOMICS 1 0.026

CURRENT HIV AIDS REPORTS 1 0.026

CURRENT OPINION IN GASTROENTEROLOGY 1 0.026

CURRENT OPINION IN HIV AND AIDS 1 0.026

CURRENT PHARMACEUTICAL BIOTECHNOLOGY 1 0.026

CURRENT PROTEIN PEPTIDE SCIENCE 1 0.026

CURRENT TOPICS IN MEDICINAL CHEMISTRY 1 0.026

CYTOTECHNOLOGY 1 0.026

DIABETES TECHNOLOGY THERAPEUTICS 1 0.026

DIAGNOSTIC PATHOLOGY 1 0.026

DIFFERENTIATION 1 0.026

DIGESTIVE AND LIVER DISEASE 1 0.026

DM DISEASE A MONTH 1 0.026

DOSE RESPONSE 1 0.026

DRUG DELIVERY 1 0.026

DRUG DISCOVERY TODAY 1 0.026

DRUG NEWS PERSPECTIVES 1 0.026

DRUG SAFETY 1 0.026

DRUGS 1 0.026

DRUGS AGING 1 0.026

DRUGS OF THE FUTURE 1 0.026

ENDOCRINOLOGY AND METABOLISM 1 0.026

EUROPEAN CYTOKINE NETWORK 1 0.026

EUROPEAN HEART JOURNAL 1 0.026

EUROPEAN HEART JOURNAL CARDIOVASCULAR PHARMACOTHERAPY 1 0.026

EUROPEAN JOURNAL OF ENDOCRINOLOGY 1 0.026

EUROPEAN JOURNAL OF GASTROENTEROLOGY HEPATOLOGY 1 0.026

EUROPEAN JOURNAL OF HAEMATOLOGY 1 0.026

EUROPEAN JOURNAL OF MEDICAL RESEARCH 1 0.026

EUROPEAN JOURNAL OF MEDICINAL CHEMISTRY 1 0.026

EUROPEAN JOURNAL OF PHARMACEUTICAL SCIENCES 1 0.026

EUROPEAN JOURNAL OF RADIOLOGY 1 0.026

EUROPEAN RADIOLOGY 1 0.026

EXPERIMENTAL GERONTOLOGY 1 0.026

EXPERT OPINION ON BIOLOGICAL THERAPY 1 0.026

EXPERT OPINION ON THERAPEUTIC PATENTS 1 0.026

EXPERT REVIEW OF ANTI INFECTIVE THERAPY 1 0.026

EXPERT REVIEW OF CLINICAL IMMUNOLOGY 1 0.026

EXPERT REVIEW OF GASTROENTEROLOGY HEPATOLOGY 1 0.026

EXPERT REVIEW OF MOLECULAR DIAGNOSTICS 1 0.026

EXPERT REVIEW OF PROTEOMICS 1 0.026

FEBS JOURNAL 1 0.026

FEBS LETTERS 1 0.026

FEBS OPEN BIO 1 0.026

FLAVOUR AND FRAGRANCE JOURNAL 1 0.026

FOLIA HISTOCHEMICA ET CYTOBIOLOGICA 1 0.026

FOOD HYDROCOLLOIDS 1 0.026

FRONTIERS IN BIOSCIENCE 1 0.026

FRONTIERS IN CARDIOVASCULAR MEDICINE 1 0.026

FRONTIERS IN GENETICS 1 0.026

FRONTIERS IN MOLECULAR BIOSCIENCES 1 0.026

FRONTIERS OF MEDICINE 1 0.026

FUTURE MEDICINAL CHEMISTRY 1 0.026

GENE THERAPY 1 0.026

GENES 1 0.026

GERIATRICS GERONTOLOGY INTERNATIONAL 1 0.026

GERONTOLOGY 1 0.026

GEROSCIENCE 1 0.026

GLYCANS AND GLYCOSAMINOGLYCANS AS CLINICAL BIOMARKERS AND THERAPEUTICS PT B 1 0.026

GROWTH FACTORS 1 0.026

GUT AND LIVER 1 0.026

HEART 1 0.026

HEART RHYTHM 1 0.026

HEPATITIS C IN RENAL DISEASE HEMODIALYSIS AND TRANSPLANTATION 1 0.026

HEPATOLOGY COMMUNICATIONS 1 0.026

HEPATOLOGY INTERNATIONAL 1 0.026

HEPATOLOGY RESEARCH 1 0.026

HIPPOKRATIA 1 0.026

HISTOCHEMICAL JOURNAL 1 0.026

HISTOCHEMISTRY AND CELL BIOLOGY 1 0.026

HISTOPATHOLOGY 1 0.026

HONG KONG MEDICAL JOURNAL 1 0.026

HORMONES INTERNATIONAL JOURNAL OF ENDOCRINOLOGY AND METABOLISM 1 0.026

HUMAN GENETICS 1 0.026

HUMAN MOLECULAR GENETICS 1 0.026

HUMAN MUTATION 1 0.026

IMMUNOLOGICAL REVIEWS 1 0.026

IMMUNOTHERAPY 1 0.026

IN VITRO CELLULAR DEVELOPMENTAL BIOLOGY ANIMAL 1 0.026

IN VIVO 1 0.026

INDIAN JOURNAL OF PHARMACEUTICAL SCIENCES 1 0.026

INFLAMMATION RESEARCH 1 0.026

INHALATION TOXICOLOGY 1 0.026

INTERNATIONAL BRAZ J UROL 1 0.026

INTERNATIONAL JOURNAL OF ARTIFICIAL ORGANS 1 0.026

INTERNATIONAL JOURNAL OF CARDIOVASCULAR IMAGING 1 0.026

INTERNATIONAL JOURNAL OF GERONTOLOGY 1 0.026

INTERNATIONAL JOURNAL OF IMMUNOTHERAPY 1 0.026

INTERNATIONAL JOURNAL OF LOWER EXTREMITY WOUNDS 1 0.026

INTERNATIONAL JOURNAL OF OBESITY 1 0.026

INTERNATIONAL JOURNAL OF RHEUMATIC DISEASES 1 0.026

INTERNATIONAL JOURNAL OF UROLOGY 1 0.026

INVESTIGATIVE RADIOLOGY 1 0.026

IRANIAN JOURNAL OF BASIC MEDICAL SCIENCES 1 0.026

IRANIAN JOURNAL OF PHARMACEUTICAL RESEARCH 1 0.026

IRISH JOURNAL OF MEDICAL SCIENCE 1 0.026

IUBMB LIFE 1 0.026

JACC HEART FAILURE 1 0.026

JCPSP JOURNAL OF THE COLLEGE OF PHYSICIANS AND SURGEONS PAKISTAN 1 0.026

JORNAL BRASILEIRO DE PNEUMOLOGIA 1 0.026

JOURNAL OF ATHEROSCLEROSIS AND THROMBOSIS 1 0.026

JOURNAL OF BIOCHEMISTRY AND MOLECULAR BIOLOGY 1 0.026

JOURNAL OF BIOMEDICAL MATERIALS RESEARCH PART A 1 0.026

JOURNAL OF BIOMEDICAL SCIENCE 1 0.026

JOURNAL OF BIOSCIENCE AND BIOENGINEERING 1 0.026

JOURNAL OF CACHEXIA SARCOPENIA AND MUSCLE 1 0.026

JOURNAL OF CARDIOVASCULAR DEVELOPMENT AND DISEASE 1 0.026

JOURNAL OF CARDIOVASCULAR MEDICINE 1 0.026

JOURNAL OF CARDIOVASCULAR PHARMACOLOGY AND THERAPEUTICS 1 0.026

JOURNAL OF CHILD PSYCHOLOGY AND PSYCHIATRY 1 0.026

JOURNAL OF CLINICAL LABORATORY ANALYSIS 1 0.026

JOURNAL OF CLINICAL RESEARCH IN PEDIATRIC ENDOCRINOLOGY 1 0.026

JOURNAL OF CLINICAL ULTRASOUND 1 0.026

JOURNAL OF CONTROLLED RELEASE 1 0.026

JOURNAL OF CRANIO MAXILLOFACIAL SURGERY 1 0.026

JOURNAL OF DERMATOLOGY 1 0.026

JOURNAL OF DEVELOPMENTAL ORIGINS OF HEALTH AND DISEASE 1 0.026

JOURNAL OF ENDOCRINOLOGICAL INVESTIGATION 1 0.026

JOURNAL OF ENDOUROLOGY 1 0.026

JOURNAL OF EPIDEMIOLOGY 1 0.026

JOURNAL OF ESSENTIAL OIL BEARING PLANTS 1 0.026

JOURNAL OF EXTRACELLULAR VESICLES 1 0.026

JOURNAL OF FOOD BIOCHEMISTRY 1 0.026

JOURNAL OF GASTROENTEROLOGY 1 0.026

JOURNAL OF HEART VALVE DISEASE 1 0.026

JOURNAL OF HUMAN GENETICS 1 0.026

JOURNAL OF IMMUNOLOGY 1 0.026

JOURNAL OF INFECTION 1 0.026

JOURNAL OF INFLAMMATION RESEARCH 1 0.026

JOURNAL OF INHERITED METABOLIC DISEASE 1 0.026

JOURNAL OF INTERNAL MEDICINE 1 0.026

JOURNAL OF KING SAUD UNIVERSITY SCIENCE 1 0.026

JOURNAL OF KOREAN MEDICAL SCIENCE 1 0.026

JOURNAL OF LEUKOCYTE BIOLOGY 1 0.026

JOURNAL OF LIPID RESEARCH 1 0.026

JOURNAL OF MEDICAL BIOCHEMISTRY 1 0.026

JOURNAL OF MEDICINAL CHEMISTRY 1 0.026

JOURNAL OF MOLECULAR DIAGNOSTICS 1 0.026

JOURNAL OF MOLECULAR HISTOLOGY 1 0.026

JOURNAL OF NUTRITION 1 0.026

JOURNAL OF NUTRITIONAL SCIENCE AND VITAMINOLOGY 1 0.026

JOURNAL OF PARASITOLOGY 1 0.026

JOURNAL OF PEDIATRICS 1 0.026

JOURNAL OF PHYSIOLOGY AND PHARMACOLOGY 1 0.026

JOURNAL OF PROTEOMICS 1 0.026

JOURNAL OF RENAL NUTRITION 1 0.026

JOURNAL OF SEXUAL MEDICINE 1 0.026

JOURNAL OF STEROID BIOCHEMISTRY AND MOLECULAR BIOLOGY 1 0.026

JOURNAL OF SURGICAL RESEARCH 1 0.026

JOURNAL OF THE AMERICAN ACADEMY OF DERMATOLOGY 1 0.026

JOURNAL OF THE AMERICAN COLLEGE OF NUTRITION 1 0.026

JOURNAL OF THE AMERICAN DIETETIC ASSOCIATION 1 0.026

JOURNAL OF THE AMERICAN PODIATRIC MEDICAL ASSOCIATION 1 0.026

JOURNAL OF THE AMERICAN SOCIETY OF ECHOCARDIOGRAPHY 1 0.026

JOURNAL OF THE AMERICAN SOCIETY OF HYPERTENSION 1 0.026

JOURNAL OF THE EUROPEAN ACADEMY OF DERMATOLOGY AND VENEREOLOGY 1 0.026

JOURNAL OF THE FORMOSAN MEDICAL ASSOCIATION 1 0.026

JOURNAL OF THE SCIENCE OF FOOD AND AGRICULTURE 1 0.026

JOURNAL OF THROMBOSIS AND HAEMOSTASIS 1 0.026

JOURNAL OF TOXICOLOGIC PATHOLOGY 1 0.026

JOURNAL OF TOXICOLOGICAL SCIENCES 1 0.026

JOURNAL OF TRACE ELEMENTS IN MEDICINE AND BIOLOGY 1 0.026

JOURNAL OF ULTRASOUND IN MEDICINE 1 0.026

JOURNAL OF VETERINARY MEDICAL SCIENCE 1 0.026

JOURNAL OF VETERINARY PHARMACOLOGY AND THERAPEUTICS 1 0.026

JOURNALS OF GERONTOLOGY SERIES A BIOLOGICAL SCIENCES AND MEDICAL SCIENCES 1 0.026

KAFKAS UNIVERSITESI VETERINER FAKULTESI DERGISI 1 0.026

KARDIOLOGIA POLSKA 1 0.026

KLINISCHE WOCHENSCHRIFT 1 0.026

KOREAN JOURNAL OF INTERNAL MEDICINE 1 0.026

KOREAN JOURNAL OF PATHOLOGY 1 0.026

KOREAN JOURNAL OF PHYSIOLOGY PHARMACOLOGY 1 0.026

KOREAN JOURNAL OF RADIOLOGY 1 0.026

LANCET RESPIRATORY MEDICINE 1 0.026

LEGAL MEDICINE 1 0.026

LUPUS 1 0.026

MAGNETIC RESONANCE IMAGING 1 0.026

MATRIX METALLOPROTEINASES AND TISSUE REMODELING IN HEALTH AND DISEASE TARGET TISSUES AND THERAPY 1 0.026

MEDICAL PRINCIPLES AND PRACTICE 1 0.026

MEDICC REVIEW 1 0.026

MEDICINAL NATURAL PRODUCTS A DISEASE FOCUSED APPROACH 1 0.026

MEDICINAL RESEARCH REVIEWS 1 0.026

MEDICINE SCIENCE AND THE LAW 1 0.026

METABOLIC BRAIN DISEASE 1 0.026

MICROBES AND INFECTION 1 0.026

MICRORNA MEDICAL EVIDENCE FROM MOLECULAR BIOLOGY TO CLINICAL PRACTICE 1 0.026

MINERVA MEDICA 1 0.026

MINERVA UROLOGICA E NEFROLOGICA 1 0.026

MOLECULAR CELLULAR TOXICOLOGY 1 0.026

MOLECULAR MEMBRANE BIOLOGY 1 0.026

MOLECULES AND CELLS 1 0.026

MOUNT SINAI JOURNAL OF MEDICINE 1 0.026

MUTATION RESEARCH REVIEWS IN MUTATION RESEARCH 1 0.026

NANOMEDICINE 1 0.026

NANOMEDICINE NANOTECHNOLOGY BIOLOGY AND MEDICINE 1 0.026

NANOSCIENCE AND NANOTECHNOLOGY LETTERS 1 0.026

NATURAL PRODUCT COMMUNICATIONS 1 0.026

NATURAL PRODUCT RESEARCH 1 0.026

NATURE CLINICAL PRACTICE RHEUMATOLOGY 1 0.026

NATURE MEDICINE 1 0.026

NATURE REVIEWS DRUG DISCOVERY 1 0.026

NATURE REVIEWS UROLOGY 1 0.026

NUCLEIC ACID THERAPEUTICS 1 0.026

NUTRITION 1 0.026

NUTRITION METABOLISM 1 0.026

NUTRITION RESEARCH 1 0.026

NUTRITION RESEARCH AND PRACTICE 1 0.026

OBESITY AND THE KIDNEY 1 0.026

OBESITY SURGERY 1 0.026

OBSTETRICAL GYNECOLOGICAL SURVEY 1 0.026

OMICS A JOURNAL OF INTEGRATIVE BIOLOGY 1 0.026

OPEN MEDICINE 1 0.026

PACE PACING AND CLINICAL ELECTROPHYSIOLOGY 1 0.026

PAEDIATRIC RESPIRATORY REVIEWS 1 0.026

PAEDIATRICS CHILD HEALTH 1 0.026

PAKISTAN JOURNAL OF PHARMACEUTICAL SCIENCES 1 0.026

PEDIATRIC PULMONOLOGY 1 0.026

PEDIATRIC RESEARCH 1 0.026

PEDIATRIC TRANSPLANTATION 1 0.026

PERSONALIZED MEDICINE 1 0.026

PHARMACEUTICAL BIOLOGY 1 0.026

PHARMACEUTICAL RESEARCH 1 0.026

PHARMACOLOGICAL REVIEWS 1 0.026

PHARMACOLOGY RESEARCH PERSPECTIVES 1 0.026

PHOTOCHEMISTRY AND PHOTOBIOLOGY 1 0.026

PHYSIOLOGICAL RESEARCH 1 0.026

PLANTA MEDICA 1 0.026

PLASMINOGEN RELATED GROWTH FACTORS 1 0.026

PLOS BIOLOGY 1 0.026

PLOS GENETICS 1 0.026

PLOS MEDICINE 1 0.026

PLOS NEGLECTED TROPICAL DISEASES 1 0.026

PODOCYTOPATHY 1 0.026

POSTGRADUATE MEDICAL JOURNAL 1 0.026

PRIMARY CARE 1 0.026

PROCEEDINGS OF THE JAPAN ACADEMY SERIES B PHYSICAL AND BIOLOGICAL SCIENCES 1 0.026

PROSTAGLANDINS LEUKOTRIENES AND ESSENTIAL FATTY ACIDS 1 0.026

PROTEOMICS CLINICAL APPLICATIONS 1 0.026

PURINERGIC SIGNALLING 1 0.026

QJM AN INTERNATIONAL JOURNAL OF MEDICINE 1 0.026

RADIOLOGY 1 0.026

REDOX REPORT 1 0.026

REGULATORY PEPTIDES 1 0.026

RENIN ANGIOTENSIN SYSTEM AND PROGRESSION OF RENAL DISEASES 1 0.026

RESEARCH IN NURSING HEALTH 1 0.026

RESPIRATORY MEDICINE 1 0.026

REVIEWS IN ENDOCRINE METABOLIC DISORDERS 1 0.026

REVIEWS IN MEDICAL VIROLOGY 1 0.026

REVISTA ESPANOLA DE ENFERMEDADES DIGESTIVAS 1 0.026

RHEUMATOLOGY 1 0.026

RHEUMATOLOGY INTERNATIONAL 1 0.026

SAUDI JOURNAL OF BIOLOGICAL SCIENCES 1 0.026

SCANDINAVIAN JOURNAL OF PUBLIC HEALTH 1 0.026

SCIENCE OF THE TOTAL ENVIRONMENT 1 0.026

SCIENTIFIC WORLD JOURNAL 1 0.026

SEMINARS IN IMMUNOPATHOLOGY 1 0.026

SEMINARS IN RESPIRATORY AND CRITICAL CARE MEDICINE 1 0.026

SEMINARS IN VASCULAR SURGERY 1 0.026

SIGNAL TRANSDUCTION AND TARGETED THERAPY 1 0.026

STEM CELLS AND DEVELOPMENT 1 0.026

STEM CELLS TRANSLATIONAL MEDICINE 1 0.026

SURGERY 1 0.026

SWISS MEDICAL WEEKLY 1 0.026

THERANOSTICS 1 0.026

THERAPEUTIC ADVANCES IN MEDICAL ONCOLOGY 1 0.026

THERAPEUTIC APHERESIS AND DIALYSIS 1 0.026

THERAPEUTIC DRUG MONITORING 1 0.026

THERAPEUTIC ULTRASOUND 1 0.026

THERAPEUTICS AND CLINICAL RISK MANAGEMENT 1 0.026

THESCIENTIFICWORLDJOURNAL 1 0.026

THORAX 1 0.026

THROMBOSIS AND HAEMOSTASIS 1 0.026

TISSUE ENGINEERING 1 0.026

TOMOGRAPHY 1 0.026

TOXICOLOGY LETTERS 1 0.026

TOXICOLOGY MECHANISMS AND METHODS 1 0.026

TRANSLATIONAL ANDROLOGY AND UROLOGY 1 0.026

TRANSPLANT INTERNATIONAL 1 0.026

TRANSPLANTATION REVIEWS 1 0.026

TRENDS IN CELL BIOLOGY 1 0.026

TROPICAL MEDICINE INTERNATIONAL HEALTH 1 0.026

TURKISH JOURNAL OF GASTROENTEROLOGY 1 0.026

TURKISH JOURNAL OF GERIATRICS TURK GERIATRI DERGISI 1 0.026

TURKISH JOURNAL OF PEDIATRICS 1 0.026

ULTRASONIC IMAGING 1 0.026

ULTRASOUND QUARTERLY 1 0.026

UNITED EUROPEAN GASTROENTEROLOGY JOURNAL 1 0.026

UPSALA JOURNAL OF MEDICAL SCIENCES 1 0.026

URIC ACID IN CHRONIC KIDNEY DISEASE 1 0.026

UROLOGIA INTERNATIONALIS 1 0.026

UROLOGY 1 0.026

VETERINARY JOURNAL 1 0.026

VETERINARY PATHOLOGY 1 0.026

VIRCHOWS ARCHIV A PATHOLOGICAL ANATOMY AND HISTOPATHOLOGY 1 0.026

VITAMINS AND HORMONES 1 0.026

WORLD JOURNAL OF SURGERY 1 0.026

WORLD JOURNAL OF UROLOGY 1 0.026

WOUND REPAIR AND REGENERATION 1 0.026

YEAR IN DIABETES AND OBESITY 1 0.026

ZEITSCHRIFT FUR GASTROENTEROLOGIE 1 0.026

(0 records (0.000%) do not contain data in the field being analyzed.)
