# Supplementary material for: Bibliometric Analysis of Renal Fibrosis in Diabetic Kidney Disease From 1985 to 2020
Source: Front Public Health. 2022 Feb 4;10:767591. doi: 10.3389/fpubh.2022.767591 (PMC8855938; doi:10.3389/fpubh.2022.767591)
Supplement: Supplementary file 4 [file Table_4.DOCX]

Authors records % of 3821

COOPER ME 47 1.230

LAN HY 44 1.152

LI Y 44 1.152

ZHANG Y 43 1.125

WANG Y 42 1.099

LIU Y 36 0.942

LI J 33 0.864

ZHANG J 32 0.837

HUANG HQ 31 0.811

POLLOCK CA 31 0.811

KOYA D 30 0.785

CAI L 28 0.733

LIU F 28 0.733

CHEN XM 27 0.707

GOLDSCHMEDING R 27 0.707

HUANG XR 26 0.680

LIU YH 26 0.680

YANG Y 26 0.680

GILBERT RE 25 0.654

KANASAKI K 25 0.654

WANG L 25 0.654

TESCH GH 24 0.628

LI L 23 0.602

NIKOLIC-PATERSON DJ 23 0.602

ZHANG L 23 0.602

CHEN X 21 0.550

EGIDO J 21 0.550

KELLY DJ 21 0.550

LI X 21 0.550

XU Y 21 0.550

ZHANG C 21 0.550

GODSON C 20 0.523

ORTIZ A 20 0.523

SUN L 20 0.523

WANG J 20 0.523

ZHANG W 20 0.523

LIU G 19 0.497

LIU PQ 19 0.497

NATARAJAN R 19 0.497

RUIZ-ORTEGA M 19 0.497

TAN Y 19 0.497

ZHANG F 19 0.497

ZHANG H 19 0.497

CHUANG LY 18 0.471

GUH JY 18 0.471

KITADA M 18 0.471

LIU H 18 0.471

CHEN L 17 0.445

HA H 17 0.445

LI C 17 0.445

TANG SCW 17 0.445

WANG JY 17 0.445

WOLF G 17 0.445

XIAO Y 17 0.445

YANG YL 17 0.445

ZHANG YY 17 0.445

CHEN ZQ 16 0.419

CHUNG ACK 16 0.419

HARRIS RC 16 0.419

HUANG YF 16 0.419

KANTHARIDIS P 16 0.419

LI H 16 0.419

LI P 16 0.419

LIANG G 16 0.419

LIU BC 16 0.419

PHILLIPS AO 16 0.419

WANG M 16 0.419

WANG YY 16 0.419

ZHANG XL 16 0.419

CHEN Y 15 0.393

KATO M 15 0.393

LEVI M 15 0.393

LI YJ 15 0.393

LIN CL 15 0.393

LIU J 15 0.393

NGUYEN TQ 15 0.393

THOMAS MC 15 0.393

WANG B 15 0.393

WANG Q 15 0.393

WANG S 15 0.393

WANG XL 15 0.393

XUE YM 15 0.393

ZIYADEH FN 15 0.393

CHEN J 14 0.366

GONG WY 14 0.366

GUO B 14 0.366

HUANG JY 14 0.366

KIM YS 14 0.366

MARTIN F 14 0.366

NISHIYAMA A 14 0.366

SHARMA K 14 0.366

SHI MJ 14 0.366

YIN XX 14 0.366

ANDERS HJ 13 0.340

BENIGNI A 13 0.340

DAI CS 13 0.340

FOGO AB 13 0.340

KIM J 13 0.340

LEE SH 13 0.340

LERMAN LO 13 0.340

LI HY 13 0.340

SUSZTAK K 13 0.340

WANG F 13 0.340

WU H 13 0.340

XU L 13 0.340

YIU WH 13 0.340

CHEN C 12 0.314

CHEN HY 12 0.314

CHEN Q 12 0.314

CHENIER I 12 0.314

CHOUDHURY GG 12 0.314

FORBES JM 12 0.314

GUAN GJ 12 0.314

HUANG CL 12 0.314

KASINATH BS 12 0.314

KIM HS 12 0.314

KRETZLER M 12 0.314

LANG F 12 0.314

LU Q 12 0.314

MA FY 12 0.314

MEZZANO S 12 0.314

MIAO LN 12 0.314

PARK J 12 0.314

VAN GOOR H 12 0.314

WADA J 12 0.314

WANG H 12 0.314

WANG QY 12 0.314

XIE X 12 0.314

YANG J 12 0.314

ZHANG SL 12 0.314

ZHANG Z 12 0.314

ZHENG ZJ 12 0.314

ZHOU Y 12 0.314

BRAZIL DP 11 0.288

CHAN JSD 11 0.288

CUI WP 11 0.288

DE BOER RA 11 0.288

GAIKWAD AB 11 0.288

GAO YB 11 0.288

HUANG JS 11 0.288

INGELFINGER JR 11 0.288

ITO S 11 0.288

LAI KN 11 0.288

LEE HS 11 0.288

LI JH 11 0.288

LI XK 11 0.288

LIU FY 11 0.288

MAKINO H 11 0.288

MARIC C 11 0.288

MURPHY M 11 0.288

NOBLE NA 11 0.288

SRIVASTAVA SP 11 0.288

SUN Y 11 0.288

WU C 11 0.288

YANG JW 11 0.288

YANG M 11 0.288

ZHANG HJ 11 0.288

ZHANG M 11 0.288

ZHANG MZ 11 0.288

ZHANG N 11 0.288

ZHANG R 11 0.288

ZHANG X 11 0.288

CHA DR 10 0.262

CHATZIANTONIOU C 10 0.262

COUGHLAN MT 10 0.262

FILEP JG 10 0.262

HANEDA M 10 0.262

HIRSCHBERG R 10 0.262

HSU YC 10 0.262

HUANG J 10 0.262

JANDELEIT-DAHM K 10 0.262

JIA Y 10 0.262

JIA YJ 10 0.262

JIANG L 10 0.262

JIANG T 10 0.262

JOLES JA 10 0.262

KANWAR YS 10 0.262

KIM HJ 10 0.262

KOPP JB 10 0.262

LEUNG JCK 10 0.262

LI M 10 0.262

LI N 10 0.262

MA KL 10 0.262

MULLER GA 10 0.262

NAKAGAWA T 10 0.262

NITTA K 10 0.262

REMUZZI G 10 0.262

SAAD S 10 0.262

SHEN Y 10 0.262

SUN WX 10 0.262

WANG D 10 0.262

XUE M 10 0.262

ZHANG JL 10 0.262

ZHANG XM 10 0.262

ZHAO YY 10 0.262

ZHOU J 10 0.262

ATEN J 9 0.236

BORDER WA 9 0.236

CHEN G 9 0.236

CHEN QH 9 0.236

CHEN S 9 0.236

COHEN CD 9 0.236

DONG Z 9 0.236

DUSSAULE JC 9 0.236

EPSTEIN PN 9 0.236

FLOEGE J 9 0.236

FU P 9 0.236

GOMEZ-GUERRERO C 9 0.236

HALLER H 9 0.236

HAN SY 9 0.236

HOCHER B 9 0.236

HUANG KP 9 0.236

HUANG Y 9 0.236

ITO Y 9 0.236

KANG SW 9 0.236

KANG YH 9 0.236

LEE YJ 9 0.236

LI B 9 0.236

LI F 9 0.236

LI R 9 0.236

LI S 9 0.236

LI XY 9 0.236

LI YC 9 0.236

LIU L 9 0.236

LIU WJ 9 0.236

LIU X 9 0.236

LIU ZH 9 0.236

LO CS 9 0.236

MENG XM 9 0.236

NAVIS G 9 0.236

OHASHI K 9 0.236

PARK JH 9 0.236

PENG YM 9 0.236

SCHANSTRA JP 9 0.236

SHAO Y 9 0.236

SOLER MJ 9 0.236

TIAN JW 9 0.236

WANG X 9 0.236

WU J 9 0.236

WU XY 9 0.236

XU J 9 0.236

YANG CW 9 0.236

YANG H 9 0.236

YANG L 9 0.236

ZHANG Q 9 0.236

ZHANG XY 9 0.236

ZHANG YL 9 0.236

ZHAO L 9 0.236

ZHAO TT 9 0.236

ALPERS CE 8 0.209

ATKINS RC 8 0.209

BAI XY 8 0.209

BASCANDS JL 8 0.209

BERTRAM JF 8 0.209

CHAN LYY 8 0.209

CHEN H 8 0.209

CHEN HC 8 0.209

DOMINGUEZ JH 8 0.209

DROGUETT A 8 0.209

DU L 8 0.209

FAN QL 8 0.209

GANSEVOORT RT 8 0.209

GAO P 8 0.209

GUAN MP 8 0.209

HILLS CE 8 0.209

JIANG Y 8 0.209

KANG DG 8 0.209

KUME S 8 0.209

LEE HB 8 0.209

LI LL 8 0.209

LI W 8 0.209

LIANG D 8 0.209

LIU WH 8 0.209

LV C 8 0.209

MEIER M 8 0.209

NAGAI Y 8 0.209

NANGAKU M 8 0.209

NGUYEN G 8 0.209

OGURA Y 8 0.209

PARK SH 8 0.209

PENG W 8 0.209

POLLOCK C 8 0.209

QU XL 8 0.209

RABELINK TJ 8 0.209

ROMAN RJ 8 0.209

SANCHEZ-NINO MD 8 0.209

SCHOLEY JW 8 0.209

SEGERER S 8 0.209

SHEN XY 8 0.209

TAKAHASHI T 8 0.209

TANG L 8 0.209

TAO LJ 8 0.209

WANG CJ 8 0.209

WANG HY 8 0.209

WANG SN 8 0.209

WANG SY 8 0.209

WANG W 8 0.209

WANG ZH 8 0.209

WU HL 8 0.209

WU YG 8 0.209

XU H 8 0.209

YAN MH 8 0.209

YANG HC 8 0.209

YANG S 8 0.209

YOON JJ 8 0.209

ZEISBERG M 8 0.209

ZHANG LY 8 0.209

ZHOU LL 8 0.209

ZHU XJ 8 0.209

ZHUANG SG 8 0.209

ALLEN TJ 7 0.183

BARNES JL 7 0.183

BARUTTA F 7 0.183

BRENNAN EP 7 0.183

BRUNO G 7 0.183

CAO G 7 0.183

CHEN LM 7 0.183

CHEUNG AK 7 0.183

DAS S 7 0.183

FUKAMI K 7 0.183

GAMBARO G 7 0.183

GAMBINO R 7 0.183

GORIN Y 7 0.183

GRUDEN G 7 0.183

GUO H 7 0.183

HAGIWARA S 7 0.183

HE JC 7 0.183

HO C 7 0.183

HU F 7 0.183

HUANG S 7 0.183

HUANG YJ 7 0.183

HUANG YT 7 0.183

IMIG JD 7 0.183

JERUMS G 7 0.183

JOHNSON TS 7 0.183

KALLURI R 7 0.183

KANG YS 7 0.183

KAWANAMI D 7 0.183

KIM S 7 0.183

LEE IK 7 0.183

LEE JH 7 0.183

LI HL 7 0.183

LI Q 7 0.183

LI SY 7 0.183

LI XZ 7 0.183

LI YQ 7 0.183

LIN M 7 0.183

LINDENMEYER MT 7 0.183

LIU LR 7 0.183

LIU XC 7 0.183

LIU YQ 7 0.183

LOEFFLER I 7 0.183

LV LL 7 0.183

MA J 7 0.183

MA JX 7 0.183

MA XY 7 0.183

MAYER G 7 0.183

MOLL S 7 0.183

NAGAI T 7 0.183

OYARZUN C 7 0.183

OZOLS E 7 0.183

PANCHAPAKESAN U 7 0.183

PENG ZZ 7 0.183

PFEILSCHIFTER J 7 0.183

QUEZADA C 7 0.183

RAYEGO-MATEOS S 7 0.183

ROSSING P 7 0.183

RULE AD 7 0.183

SCHAEFER L 7 0.183

SHARMA A 7 0.183

SHI S 7 0.183

SHI Y 7 0.183

SQUIRES PE 7 0.183

SUGAYA T 7 0.183

SUN LN 7 0.183

SZETO CC 7 0.183

TAKAHASHI N 7 0.183

TANG J 7 0.183

THALLAS-BONKE V 7 0.183

TIKELLIS C 7 0.183

TOUYZ RM 7 0.183

TUTTLE KR 7 0.183

UBARA Y 7 0.183

VALLON V 7 0.183

VAZIRI ND 7 0.183

WADA T 7 0.183

WANG SS 7 0.183

WANG WW 7 0.183

WANG YW 7 0.183

WANG Z 7 0.183

WONG DWL 7 0.183

WU HJ 7 0.183

XIAO L 7 0.183

ZHANG B 7 0.183

ZHAO Y 7 0.183

ZHENG SR 7 0.183

ZHOU L 7 0.183

ABBOUD HE 6 0.157

ALARCON S 6 0.157

BOB F 6 0.157

BOOR P 6 0.157

BROWN L 6 0.157

BRUIJN JA 6 0.157

BURNS KD 6 0.157

BURNS WC 6 0.157

CARPIO D 6 0.157

CHEN N 6 0.157

CHEN WY 6 0.157

CHEN YY 6 0.157

CHI ZH 6 0.157

D'AGATI VD 6 0.157

DAHA MR 6 0.157

DAS F 6 0.157

DE HAAN JB 6 0.157

DUAN HJ 6 0.157

EGUCHI J 6 0.157

EID AA 6 0.157

FELIERS D 6 0.157

FERNANDEZ-FERNANDEZ B 6 0.157

FLYVBJERG A 6 0.157

FUJII T 6 0.157

GAGLIARDINI E 6 0.157

GAO CL 6 0.157

GENG J 6 0.157

GHOSH-CHOUDHURY N 6 0.157

GORU SK 6 0.157

GRONE HJ 6 0.157

GU CY 6 0.157

GUO F 6 0.157

HAN JY 6 0.157

HAN KH 6 0.157

HARA S 6 0.157

HE LY 6 0.157

HEERSPINK HJL 6 0.157

HERMAN-EDELSTEIN M 6 0.157

HIGGINS PJ 6 0.157

HILLEBRANDS JL 6 0.157

HOSHINO J 6 0.157

HOU FF 6 0.157

HUANG L 6 0.157

HUANG W 6 0.157

HUANG XZ 6 0.157

HUNG WC 6 0.157

HUWILER A 6 0.157

ITO T 6 0.157

JHA JC 6 0.157

JUNG GS 6 0.157

KELLY KJ 6 0.157

KIM Y 6 0.157

KIYOMOTO H 6 0.157

KLAHR S 6 0.157

KOHAN DE 6 0.157

KOMERS R 6 0.157

LAN T 6 0.157

LEE ES 6 0.157

LEE H 6 0.157

LEE S 6 0.157

LEE SY 6 0.157

LEE TC 6 0.157

LI JP 6 0.157

LI JY 6 0.157

LI XH 6 0.157

LI XQ 6 0.157

LI YH 6 0.157

LIAN X 6 0.157

LIAO TN 6 0.157

LIU C 6 0.157

LIU HD 6 0.157

LIU HF 6 0.157

LIU LL 6 0.157

LIU SH 6 0.157

LIU XQ 6 0.157

LIU Z 6 0.157

LU XM 6 0.157

LU Y 6 0.157

LUPO A 6 0.157

LV SS 6 0.157

MALEK V 6 0.157

MASAKI T 6 0.157

MASOLA V 6 0.157

MATOBA K 6 0.157

MIYATA T 6 0.157

MORRISSEY J 6 0.157

MUKOYAMA M 6 0.157

NAKAMURA T 6 0.157

NELSON RG 6 0.157

NIWA T 6 0.157

OGAWA D 6 0.157

OKADA H 6 0.157

OKUDA S 6 0.157

ONISTO M 6 0.157

PACKER M 6 0.157

PANDEY A 6 0.157

PARK CW 6 0.157

PARK JK 6 0.157

PARK JT 6 0.157

PARK KG 6 0.157

PARVING HH 6 0.157

PETRICA L 6 0.157

PHILLIPS A 6 0.157

RAFIQ K 6 0.157

RANE MJ 6 0.157

RITZ E 6 0.157

SAN MARTIN R 6 0.157

SANZ AB 6 0.157

SHIH YH 6 0.157

SHIN SJ 6 0.157

SIMONSON MS 6 0.157

SONG Y 6 0.157

SOWERS JR 6 0.157

STEADMAN R 6 0.157

SU H 6 0.157

SU Y 6 0.157

SUN B 6 0.157

SUN HL 6 0.157

SUN J 6 0.157

SUN Q 6 0.157

SUN SF 6 0.157

SUZUKI H 6 0.157

TANG PMK 6 0.157

TANG X 6 0.157

TOBLLI JE 6 0.157

UEDA S 6 0.157

UTSUNOMIYA K 6 0.157

WANG C 6 0.157

WANG FS 6 0.157

WANG G 6 0.157

WANG HJ 6 0.157

WANG JP 6 0.157

WANG K 6 0.157

WANG LJ 6 0.157

WANG QZ 6 0.157

WANG SG 6 0.157

WANG SJ 6 0.157

WANG T 6 0.157

WANG XXX 6 0.157

WANG XY 6 0.157

WANG YG 6 0.157

WANG YT 6 0.157

WANG YX 6 0.157

WU M 6 0.157

WU X 6 0.157

WU Y 6 0.157

XIA LL 6 0.157

XIAO J 6 0.157

YAMAGISHI S 6 0.157

YAMANOUCHI M 6 0.157

YAN R 6 0.157

YANAGITA M 6 0.157

YU XQ 6 0.157

YU Y 6 0.157

ZANNAD F 6 0.157

ZHANG CX 6 0.157

ZHANG DM 6 0.157

ZHANG DS 6 0.157

ZHANG HB 6 0.157

ZHANG T 6 0.157

ZHANG XH 6 0.157

ZHANG YD 6 0.157

ZHANG YJ 6 0.157

ZHANG YQ 6 0.157

ZHOU ZM 6 0.157

ZHU X 6 0.157

ZHU Y 6 0.157

ZHU ZY 6 0.157

ZOU DW 6 0.157

ADVANI A 5 0.131

AGARWAL A 5 0.131

AGARWAL R 5 0.131

AMANN K 5 0.131

BARTON M 5 0.131

BELLINI S 5 0.131

BLANCO J 5 0.131

BOFFA JJ 5 0.131

CARUANA G 5 0.131

CHAI ZL 5 0.131

CHANG SY 5 0.131

CHEN F 5 0.131

CHEN JC 5 0.131

CHEN JH 5 0.131

CHEN Z 5 0.131

CHEN ZY 5 0.131

CHENG JQ 5 0.131

CHENG R 5 0.131

CHENG Y 5 0.131

CHIANG TA 5 0.131

CHIN-KANASAKI M 5 0.131

CHOI BS 5 0.131

CHOW KM 5 0.131

COIMBRA TM 5 0.131

CREAN JK 5 0.131

CRUZADO JM 5 0.131

DAI Q 5 0.131

DENG YH 5 0.131

DEWANJEE S 5 0.131

DIWAN V 5 0.131

DONG JJ 5 0.131

DUAN SB 5 0.131

EL NAHAS AM 5 0.131

FAN F 5 0.131

FENG B 5 0.131

FOGO A 5 0.131

FRASER D 5 0.131

FUJITA H 5 0.131

GENOVESE F 5 0.131

GEORGE J 5 0.131

GIANI JF 5 0.131

GOBE G 5 0.131

GOBE GC 5 0.131

GOODWIN JE 5 0.131

GRANDE JP 5 0.131

GRIFFIN M 5 0.131

GU Y 5 0.131

GUO RK 5 0.131

HAN DC 5 0.131

HAN QQ 5 0.131

HAN SH 5 0.131

HAO J 5 0.131

HAO LR 5 0.131

HASEGAWA E 5 0.131

HAYAMI N 5 0.131

HAYDEN MR 5 0.131

HE J 5 0.131

HE WC 5 0.131

HEWITSON TD 5 0.131

HIRAMATSU R 5 0.131

HRUSKA KA 5 0.131

HSU YH 5 0.131

HU GY 5 0.131

IMANISHI M 5 0.131

INOUE K 5 0.131

INOUE T 5 0.131

ISONO M 5 0.131

ISSHIKI K 5 0.131

JAISSER F 5 0.131

JIA Q 5 0.131

JIN J 5 0.131

JOHNSON RJ 5 0.131

KADAKOL A 5 0.131

KANASAKI M 5 0.131

KANEKO S 5 0.131

KANG MK 5 0.131

KASHIHARA N 5 0.131

KASHIWAGI A 5 0.131

KENNEDY CRJ 5 0.131

KHAN S 5 0.131

KIM DJ 5 0.131

KIM JK 5 0.131

KIM MY 5 0.131

KIM SY 5 0.131

KIM YG 5 0.131

KIMURA K 5 0.131

KLEIN J 5 0.131

KOBAYASHI S 5 0.131

KOHNO M 5 0.131

KONG LL 5 0.131

KREMERS WK 5 0.131

LANTING L 5 0.131

LAPAGE J 5 0.131

LEE PH 5 0.131

LI HJ 5 0.131

LI NJ 5 0.131

LI PKT 5 0.131

LI RX 5 0.131

LI SJ 5 0.131

LI T 5 0.131

LI Z 5 0.131

LI ZZ 5 0.131

LIAO L 5 0.131

LIESKE JC 5 0.131

LIM SS 5 0.131

LIN SH 5 0.131

LIU CX 5 0.131

LIU CY 5 0.131

LIU JS 5 0.131

LIU N 5 0.131

LIU P 5 0.131

LIU Q 5 0.131

LIU SF 5 0.131

LIU SX 5 0.131

LIU YL 5 0.131

LIU ZS 5 0.131

LONG DA 5 0.131

LOPEZ-NOVOA JM 5 0.131

LU C 5 0.131

LU J 5 0.131

LU YR 5 0.131

LUO MY 5 0.131

LUO Y 5 0.131

MAEGAWA H 5 0.131

MAO XM 5 0.131

MARSHALL JD 5 0.131

MASFERRER JL 5 0.131

MATSUMOTO K 5 0.131

MCCLELLAND A 5 0.131

MENNE J 5 0.131

MISCHAK H 5 0.131

MISE K 5 0.131

MOHAMED R 5 0.131

MORI K 5 0.131

NAGGERT JK 5 0.131

NAKATSUKA A 5 0.131

OGUIZA A 5 0.131

OLIVER N 5 0.131

OVERSTREET JM 5 0.131

PALM F 5 0.131

PENG CH 5 0.131

PENG HM 5 0.131

PENG R 5 0.131

PENG WF 5 0.131

PORONNIK P 5 0.131

QI W 5 0.131

QIN GJ 5 0.131

RAMESH G 5 0.131

RECIO C 5 0.131

RIERA M 5 0.131

RISER BL 5 0.131

SAKAI N 5 0.131

SAMARAKOON R 5 0.131

SATO S 5 0.131

SAWA N 5 0.131

SHARMA N 5 0.131

SHARMA S 5 0.131

SHI YH 5 0.131

SHI YX 5 0.131

SUMIDA K 5 0.131

SUN T 5 0.131

SUN XH 5 0.131

SUTHERLAND DER 5 0.131

SUWABE T 5 0.131

SUZUKI S 5 0.131

TACHIBANA H 5 0.131

TAKAICHI K 5 0.131

TANAKA T 5 0.131

TANG H 5 0.131

TANG HT 5 0.131

TEXTOR SC 5 0.131

THAI K 5 0.131

TIAN L 5 0.131

TIAN NX 5 0.131

UZU T 5 0.131

VAN DEN BORN J 5 0.131

VAN NIEUWENHOVEN FA 5 0.131

VAN VELDHUISEN DJ 5 0.131

VON WEBSKY K 5 0.131

WAN Q 5 0.131

WANG DD 5 0.131

WANG DW 5 0.131

WANG LY 5 0.131

WANG R 5 0.131

WANG SX 5 0.131

WANG YH 5 0.131

WANG YL 5 0.131

WANG YM 5 0.131

WANG YQ 5 0.131

WATANABE K 5 0.131

WEI QL 5 0.131

WILLIAMS JD 5 0.131

WILLIAMS JM 5 0.131

WU CT 5 0.131

XU F 5 0.131

XU K 5 0.131

XU Z 5 0.131

XU ZQ 5 0.131

YANG C 5 0.131

YANG SK 5 0.131

YANG ZY 5 0.131

YAO L 5 0.131

YOKOTA T 5 0.131

YU XY 5 0.131

ZAKY A 5 0.131

ZENG CH 5 0.131

ZERBINI G 5 0.131

ZHANG CY 5 0.131

ZHANG P 5 0.131

ZHANG S 5 0.131

ZHANG ZG 5 0.131

ZHAO D 5 0.131

ZHAO J 5 0.131

ZHAO JH 5 0.131

ZHAO XY 5 0.131

ZHONG X 5 0.131

ZHOU GY 5 0.131

ZHOU H 5 0.131

ZHOU XY 5 0.131

ZHU W 5 0.131

ZOJA C 5 0.131

ZOU CP 5 0.131

ABDO S 4 0.105

ABE M 4 0.105

ABE Y 4 0.105

ADORINI L 4 0.105

ALAM MA 4 0.105

AOYAMA I 4 0.105

ARAKI S 4 0.105

BABELOVA A 4 0.105

BADER M 4 0.105

BAELDE HJ 4 0.105

BAI YH 4 0.105

BAKKER SJL 4 0.105

BLOCK K 4 0.105

BORDA B 4 0.105

BRADY HR 4 0.105

BRENNAN E 4 0.105

BU SZ 4 0.105

BUEMI M 4 0.105

BURGOS ME 4 0.105

CAMUSSI G 4 0.105

CASTOLDI G 4 0.105

CHADBAN SJ 4 0.105

CHAKRABARTI S 4 0.105

CHAN KW 4 0.105

CHANG PJ 4 0.105

CHANG YS 4 0.105

CHAYKOVSKA L 4 0.105

CHEN B 4 0.105

CHEN FQ 4 0.105

CHEN JK 4 0.105

CHEN JX 4 0.105

CHEN KH 4 0.105

CHEN XG 4 0.105

CHEN XY 4 0.105

CHEN ZX 4 0.105

CHENG J 4 0.105

CHENG M 4 0.105

CHIANG CK 4 0.105

CHOI ME 4 0.105

CHOW FY 4 0.105

COLLIN GB 4 0.105

CONNELLY KA 4 0.105

CONWAY BR 4 0.105

COX AJ 4 0.105

CRAIG ME 4 0.105

CURRIE MG 4 0.105

D'AGATI V 4 0.105

D'ANGELO A 4 0.105

DAI AZ 4 0.105

DAI HY 4 0.105

DE HEER E 4 0.105

DE SEIGNEUX S 4 0.105

DEB DK 4 0.105

DEMARCO VG 4 0.105

DENDOOVEN A 4 0.105

DENIC A 4 0.105

DI GIOIA CRT 4 0.105

DI MARZO V 4 0.105

DIAO ZL 4 0.105

DING H 4 0.105

DOCHERTY NG 4 0.105

DOMINICI FP 4 0.105

DONG X 4 0.105

DU J 4 0.105

DUFFIELD JS 4 0.105

DURIEU I 4 0.105

EDDY AA 4 0.105

ELSHERBINY NM 4 0.105

EZQUER F 4 0.105

EZQUER M 4 0.105

FALKE LL 4 0.105

FANG L 4 0.105

FENG JX 4 0.105

FINK LN 4 0.105

FLORES C 4 0.105

FLYNN ER 4 0.105

FUJII H 4 0.105

FURUICHI K 4 0.105

GADALEAN F 4 0.105

GAO B 4 0.105

GAO Y 4 0.105

GE XX 4 0.105

GESUALDO L 4 0.105

GILL A 4 0.105

GLUHOVSCHI C 4 0.105

GOHDA T 4 0.105

GORDON SC 4 0.105

GRINYO JM 4 0.105

GUELER F 4 0.105

GUO Y 4 0.105

HABIBI J 4 0.105

HAN J 4 0.105

HAN L 4 0.105

HAN PX 4 0.105

HANSON RL 4 0.105

HE JH 4 0.105

HE LL 4 0.105

HE XM 4 0.105

HE Y 4 0.105

HEBERT RL 4 0.105

HEINZEL A 4 0.105

HOLTERMAN CE 4 0.105

HOU XY 4 0.105

HUANG C 4 0.105

HUANG CN 4 0.105

HUANG JN 4 0.105

HUANG WJ 4 0.105

HUDKINS KL 4 0.105

HUGHES J 4 0.105

HUNG CY 4 0.105

HUNG MY 4 0.105

HUNG TJ 4 0.105

HWANG SJ 4 0.105

IKEE R 4 0.105

ISHII A 4 0.105

ISHIZAWA S 4 0.105

JANKOWSKI J 4 0.105

JAYAKUMAR C 4 0.105

JI LL 4 0.105

JIA RH 4 0.105

JIANG H 4 0.105

JIANG Q 4 0.105

JIANG W 4 0.105

JOHNSON DW 4 0.105

JUN HS 4 0.105

JUNG YA 4 0.105

KAIDA Y 4 0.105

KAMIJO-IKEMORI A 4 0.105

KANAZAWA Y 4 0.105

KASAHARA M 4 0.105

KIKUCHI Y 4 0.105

KIM DH 4 0.105

KIM HW 4 0.105

KIM JE 4 0.105

KIM JH 4 0.105

KIM JS 4 0.105

KIM KS 4 0.105

KIM WY 4 0.105

KIM YC 4 0.105

KIM YM 4 0.105

KITOH M 4 0.105

KLEIN JB 4 0.105

KOGA K 4 0.105

KOK RJ 4 0.105

KONISHI Y 4 0.105

KRAG S 4 0.105

KROLEWSKI AS 4 0.105

KRUM H 4 0.105

KULKARNI YA 4 0.105

KUO YH 4 0.105

KUWABARA T 4 0.105

KWAN BCH 4 0.105

LAI YH 4 0.105

LANGHAM RG 4 0.105

LAVOZ C 4 0.105

LAZAR G 4 0.105

LAZARO I 4 0.105

LEE AS 4 0.105

LEE HJ 4 0.105

LEE J 4 0.105

LEE JE 4 0.105

LEE JP 4 0.105

LEE K 4 0.105

LEE MH 4 0.105

LEE MY 4 0.105

LEE SM 4 0.105

LEEUWIS JW 4 0.105

LEI CC 4 0.105

LENGYEL C 4 0.105

LI AM 4 0.105

LI CL 4 0.105

LI HH 4 0.105

LI JJ 4 0.105

LI JL 4 0.105

LI LY 4 0.105

LI RS 4 0.105

LI SL 4 0.105

LI SM 4 0.105

LI WZ 4 0.105

LI XJ 4 0.105

LI XL 4 0.105

LI YB 4 0.105

LI YK 4 0.105

LI YM 4 0.105

LI YY 4 0.105

LI ZH 4 0.105

LI ZJ 4 0.105

LI ZY 4 0.105

LIANG LQ 4 0.105

LIANG W 4 0.105

LIAPIS H 4 0.105

LIEBISCH M 4 0.105

LIM AKH 4 0.105

LIM JH 4 0.105

LIM SW 4 0.105

LIN X 4 0.105

LIN ZY 4 0.105

LIU DG 4 0.105

LIU HM 4 0.105

LIU HY 4 0.105

LIU S 4 0.105

LIU SS 4 0.105

LIU T 4 0.105

LIU XM 4 0.105

LIU XX 4 0.105

LIU YT 4 0.105

LIU YW 4 0.105

LONG Y 4 0.105

LU MM 4 0.105

LUFT FC 4 0.105

LUO DD 4 0.105

LUO J 4 0.105

LUO P 4 0.105

MA LJ 4 0.105

MA Q 4 0.105

MA RCW 4 0.105

MA ZJ 4 0.105

MA ZW 4 0.105

MAEDA N 4 0.105

MAITY S 4 0.105

MALLAVIA B 4 0.105

MANIGRASSO MB 4 0.105

MAO YW 4 0.105

MARIAPPAN MM 4 0.105

MARKOWITZ GS 4 0.105

MARTINEZ-SALGADO C 4 0.105

MASTROCOLA R 4 0.105

MATSUO S 4 0.105

MAUER M 4 0.105

MAXWELL AP 4 0.105

MCCLELLAND AD 4 0.105

MCKAY GJ 4 0.105

MEIJERS WC 4 0.105

MENG J 4 0.105

MIAO X 4 0.105

MIURA S 4 0.105

MIZUNO S 4 0.105

MONNO I 4 0.105

MOON JY 4 0.105

MORII T 4 0.105

MORIKAWA T 4 0.105

MURPHY-ULLRICH JE 4 0.105

NAGAI R 4 0.105

NAGAMINE J 4 0.105

NAGATA R 4 0.105

NAJAFIAN B 4 0.105

NAKAMURA S 4 0.105

NAKAMURA Y 4 0.105

NAKAO K 4 0.105

NI ZH 4 0.105

NOH H 4 0.105

OH HJ 4 0.105

ONO-KISHINO M 4 0.105

OSTENDORF T 4 0.105

PALMER M 4 0.105

PASCUAL J 4 0.105

PATSCHAN D 4 0.105

PENG C 4 0.105

PENG J 4 0.105

PENG L 4 0.105

PENG Y 4 0.105

PERCO P 4 0.105

PICKERING R 4 0.105

POLLOCK DM 4 0.105

PRUNOTTO M 4 0.105

QIAN X 4 0.105

QIN J 4 0.105

QIU CX 4 0.105

QUAN SJ 4 0.105

RAMOS AM 4 0.105

RAZZAQUE MS 4 0.105

REDDY MA 4 0.105

REN HW 4 0.105

REN XJ 4 0.105

REN Y 4 0.105

REYNAUD Q 4 0.105

RODRIGUES-DIEZ R 4 0.105

RONCO P 4 0.105

ROSENBERG AZ 4 0.105

ROSENDAHL A 4 0.105

ROSSI GP 4 0.105

RUDNICKI M 4 0.105

SAID E 4 0.105

SAKAI Y 4 0.105

SATO H 4 0.105

SATO T 4 0.105

SATO Y 4 0.105

SAULNIER-BLACHE JS 4 0.105

SCHMIDT AM 4 0.105

SCHRAMEK H 4 0.105

SCHWARZE K 4 0.105

SEBEKOVA K 4 0.105

SEN U 4 0.105

SERON D 4 0.105

SESHAN SV 4 0.105

SHAO MM 4 0.105

SHEN LS 4 0.105

SHEN S 4 0.105

SHEN YL 4 0.105

SHI J 4 0.105

SILLJE HHW 4 0.105

SILVA ACSE 4 0.105

SIRAGY HM 4 0.105

SONG JH 4 0.105

SOURRIS KC 4 0.105

STELLA A 4 0.105

SUGARU E 4 0.105

SUGAWARA A 4 0.105

SUN D 4 0.105

SUN GP 4 0.105

SUN YBY 4 0.105

SUZUKI K 4 0.105

SUZUKI T 4 0.105

SUZUKI Y 4 0.105

TAIJI M 4 0.105

TAKEDA Y 4 0.105

TAMPE B 4 0.105

TAN SM 4 0.105

TANG CY 4 0.105

TANG RM 4 0.105

TARNOW L 4 0.105

TEMM C 4 0.105

TERAMI N 4 0.105

THANDAVARAYAN RA 4 0.105

THUM T 4 0.105

TOKUNAGA T 4 0.105

TORRAS J 4 0.105

TORRES A 4 0.105

TRACHTMAN H 4 0.105

TSUPRYKOV O 4 0.105

UENO T 4 0.105

VAN DER VLAG J 4 0.105

VAN GILST WH 4 0.105

VAN KOOTEN C 4 0.105

VELCIOV S 4 0.105

VERHAAR MC 4 0.105

WAANDERS F 4 0.105

WALDHERR R 4 0.105

WALKER RJ 4 0.105

WANG HR 4 0.105

WANG JH 4 0.105

WANG JL 4 0.105

WANG JW 4 0.105

WANG LH 4 0.105

WANG LM 4 0.105

WANG NS 4 0.105

WANG P 4 0.105

WANG QQ 4 0.105

WANG TL 4 0.105

WANG WS 4 0.105

WANG XM 4 0.105

WANG YF 4 0.105

WANG YJ 4 0.105

WANG YR 4 0.105

WEN S 4 0.105

WOGENSEN L 4 0.105

WONG MG 4 0.105

WOOLF AS 4 0.105

WU CC 4 0.105

WU F 4 0.105

WU L 4 0.105

WU YC 4 0.105

XIANG XD 4 0.105

XIAO F 4 0.105

XIE YY 4 0.105

XIONG FX 4 0.105

XU G 4 0.105

XU X 4 0.105

XU ZG 4 0.105

YAMADA Y 4 0.105

YAMAMOTO H 4 0.105

YAMAMOTO M 4 0.105

YANG F 4 0.105

YANG GY 4 0.105

YANG JK 4 0.105

YANG Q 4 0.105

YANG SH 4 0.105

YANG SL 4 0.105

YANG WJ 4 0.105

YANG X 4 0.105

YANG ZJ 4 0.105

YAO B 4 0.105

YE C 4 0.105

YI H 4 0.105

YIN QH 4 0.105

YOKOI H 4 0.105

YOSHIDA H 4 0.105

YOU YK 4 0.105

YOUNG MJ 4 0.105

YU C 4 0.105

YU LC 4 0.105

YU MR 4 0.105

YUAN QJ 4 0.105

YUZAWA Y 4 0.105

ZAZA G 4 0.105

ZEN K 4 0.105

ZHANG HY 4 0.105

ZHANG JH 4 0.105

ZHANG JJ 4 0.105

ZHANG LJ 4 0.105

ZHANG WJ 4 0.105

ZHANG XX 4 0.105

ZHANG ZY 4 0.105

ZHAO H 4 0.105

ZHAO JY 4 0.105

ZHAO LJ 4 0.105

ZHAO X 4 0.105

ZHENG HX 4 0.105

ZHENG ZY 4 0.105

ZHOU X 4 0.105

ZHU D 4 0.105

ZHU XY 4 0.105

ZIMMER DP 4 0.105

ZOU XR 4 0.105

ZOU YZ 4 0.105

ADLER SG 3 0.079

ADVANI SL 3 0.079

AIZAWA Y 3 0.079

AKINCI B 3 0.079

ALEXANDER MP 3 0.079

ALI-SHAH ST 3 0.079

ALICIC RZ 3 0.079

AN JN 3 0.079

AN TT 3 0.079

ANDO R 3 0.079

ANDREWS D 3 0.079

ANGEROSA M 3 0.079

ANJANEYULU M 3 0.079

ARAKI H 3 0.079

AROOR AR 3 0.079

AUKEMA HM 3 0.079

BAE YS 3 0.079

BAI L 3 0.079

BAI SJ 3 0.079

BAI Y 3 0.079

BALAKUMAR P 3 0.079

BAMBERG K 3 0.079

BAO Y 3 0.079

BARATI MT 3 0.079

BAROZZINO M 3 0.079

BARRERA-CHIMAL J 3 0.079

BASGEN JM 3 0.079

BECKER GJ 3 0.079

BEDDHU S 3 0.079

BEDFORD JJ 3 0.079

BELLONI AS 3 0.079

BERA A 3 0.079

BHATTACHARJEE N 3 0.079

BHATTI F 3 0.079

BITZER M 3 0.079

BJORNSTAD P 3 0.079

BONVENTRE JV 3 0.079

BOOZ GW 3 0.079

BOSCH RJ 3 0.079

BRECKENRIDGE DG 3 0.079

BROSIUS FC 3 0.079

BURGER D 3 0.079

BURRELL LM 3 0.079

BURTON JO 3 0.079

BUTLER J 3 0.079

BUYS ES 3 0.079

CAI Y 3 0.079

CAO HD 3 0.079

CAO W 3 0.079

CAO Z 3 0.079

CAO ZM 3 0.079

CAPPELLI C 3 0.079

CAREW RM 3 0.079

CARLETTI R 3 0.079

CARRETERO OA 3 0.079

CASSADER M 3 0.079

CHAI Q 3 0.079

CHANDRASEKAR B 3 0.079

CHANG A 3 0.079

CHANG WT 3 0.079

CHEN CH 3 0.079

CHEN CM 3 0.079

CHEN DQ 3 0.079

CHEN HH 3 0.079

CHEN HJ 3 0.079

CHEN HM 3 0.079

CHEN HT 3 0.079

CHEN LH 3 0.079

CHEN LJ 3 0.079

CHEN LX 3 0.079

CHEN SL 3 0.079

CHEN SM 3 0.079

CHEN SY 3 0.079

CHEN W 3 0.079

CHEN XC 3 0.079

CHEN XH 3 0.079

CHEN YB 3 0.079

CHEN YC 3 0.079

CHEN YJ 3 0.079

CHEN YM 3 0.079

CHEN YN 3 0.079

CHENG JT 3 0.079

CHENG L 3 0.079

CHENG P 3 0.079

CHENG YL 3 0.079

CHEVALIER RL 3 0.079

CHIANG WC 3 0.079

CHOI H 3 0.079

CHOI YK 3 0.079

CHOPRA K 3 0.079

CHOW F 3 0.079

CHU YH 3 0.079

CHUNG CH 3 0.079

CHUNG S 3 0.079

CHURCH RH 3 0.079

CLAESSEN N 3 0.079

CONSERVA F 3 0.079

CONTREPAS A 3 0.079

CORNA D 3 0.079

CORNELL LD 3 0.079

CORRIS PA 3 0.079

COSIO FG 3 0.079

COSTA RS 3 0.079

COUSER WG 3 0.079

COVENTRY S 3 0.079

CREAN J 3 0.079

CURRAN SP 3 0.079

CURRIE BJ 3 0.079

DAMMRICH J 3 0.079

DANESH FR 3 0.079

DANIEL C 3 0.079

DANSER AHJ 3 0.079

DAS NA 3 0.079

DE GAETANO M 3 0.079

DE STRIHOU CV 3 0.079

DECLEVES AE 3 0.079

DEL VECCHIO L 3 0.079

DESHPANDE S 3 0.079

DEY P 3 0.079

DIMITRI P 3 0.079

DING LX 3 0.079

DISSARD R 3 0.079

DJAMALI A 3 0.079

DOI T 3 0.079

DONG J 3 0.079

DONG R 3 0.079

DOROTEA D 3 0.079

DUA TK 3 0.079

DUAN JA 3 0.079

DUMITRASCU V 3 0.079

DUNN SR 3 0.079

EIKMANS M 3 0.079

ELLIOTT J 3 0.079

ELMARAKBY AA 3 0.079

FAN Y 3 0.079

FAN YL 3 0.079

FANG F 3 0.079

FANG QL 3 0.079

FANG XD 3 0.079

FEKETE A 3 0.079

FENG Y 3 0.079

FERENBACH D 3 0.079

FIORETTO P 3 0.079

FISHER AJ 3 0.079

FISHER M 3 0.079

FLORQUIN S 3 0.079

FORMENTINI I 3 0.079

FRANCOIS H 3 0.079

FREEDMAN BI 3 0.079

FU X 3 0.079

FU YW 3 0.079

FUJIHARA CK 3 0.079

FUJIKURA Y 3 0.079

FUJISHIMA H 3 0.079

FUJIWARA H 3 0.079

FUJIWARA T 3 0.079

FUKAGAWA M 3 0.079

FURLONG F 3 0.079

GAFTER U 3 0.079

GAI ZB 3 0.079

GAO H 3 0.079

GAO HQ 3 0.079

GAO L 3 0.079

GARRETT MR 3 0.079

GARRIDO W 3 0.079

GE N 3 0.079

GERRITSEN KG 3 0.079

GEURTS AM 3 0.079

GHEE JY 3 0.079

GIRAUD-BILLOUD M 3 0.079

GIRIDHARAN VV 3 0.079

GLASSOCK RJ 3 0.079

GLOTZ D 3 0.079

GNUDI L 3 0.079

GODIN N 3 0.079

GONG YW 3 0.079

GONZALEZ FJ 3 0.079

GONZALEZ J 3 0.079

GOSS CH 3 0.079

GOUJON JM 3 0.079

GRETZ N 3 0.079

GRIFFIN B 3 0.079

GROOP PH 3 0.079

GROSS O 3 0.079

GROSS S 3 0.079

GU HF 3 0.079

GU LY 3 0.079

GU YT 3 0.079

GUAN SS 3 0.079

GUAN YF 3 0.079

GUIDOLIN D 3 0.079

GUO J 3 0.079

GUO WY 3 0.079

GUPTA S 3 0.079

GUTSOL A 3 0.079

HABIB SL 3 0.079

HADJADJ S 3 0.079

HAMAR P 3 0.079

HAN BH 3 0.079

HAN F 3 0.079

HAN H 3 0.079

HAN WB 3 0.079

HAN WX 3 0.079

HAN YJ 3 0.079

HAN ZJ 3 0.079

HAO M 3 0.079

HARCOURT BE 3 0.079

HASAN A 3 0.079

HATANAKA T 3 0.079

HAYASHI N 3 0.079

HE B 3 0.079

HE LJ 3 0.079

HE Q 3 0.079

HE T 3 0.079

HE YN 3 0.079

HEIDLAND A 3 0.079

HEINZE G 3 0.079

HENSTRIDGE DC 3 0.079

HERRERO-FRESNEDA I 3 0.079

HERZENBERG AM 3 0.079

HIGGINS SP 3 0.079

HILGERS KF 3 0.079

HILL C 3 0.079

HILLEGE HL 3 0.079

HINDEN L 3 0.079

HOLIAN J 3 0.079

HONG YA 3 0.079

HORIE Y 3 0.079

HORIGUCHI CS 3 0.079

HORIKOSHI S 3 0.079

HOSHINO S 3 0.079

HOY WE 3 0.079

HRYCIW DH 3 0.079

HSU CC 3 0.079

HU J 3 0.079

HUANG FJ 3 0.079

HUANG H 3 0.079

HUANG HP 3 0.079

HUANG LH 3 0.079

HUANG WL 3 0.079

HUME WE 3 0.079

HUNG CC 3 0.079

HUNG YJ 3 0.079

HWANG SH 3 0.079

IACOBINI C 3 0.079

IMAI H 3 0.079

IMAKIIRE T 3 0.079

INA K 3 0.079

INAGI R 3 0.079

ISAKA Y 3 0.079

ISHIGAKI Y 3 0.079

ITOH H 3 0.079

IX JH 3 0.079

IZOPET J 3 0.079

JACOBS-CACHA C 3 0.079

JANDELEIT-DAHM KA 3 0.079

JANDELEIT-DAHM KAM 3 0.079

JARA C 3 0.079

JEE YH 3 0.079

JHA S 3 0.079

JHENG HF 3 0.079

JI TT 3 0.079

JI XY 3 0.079

JIANG HY 3 0.079

JIANG S 3 0.079

JIANG WL 3 0.079

JIANG ZP 3 0.079

JING H 3 0.079

JOHANSSON U 3 0.079

JOHN R 3 0.079

JOSE PA 3 0.079

KAGAWA T 3 0.079

KAMAR N 3 0.079

KANG JM 3 0.079

KARSDAL MA 3 0.079

KAUCSAR T 3 0.079

KE B 3 0.079

KERESZTES C 3 0.079

KERR B 3 0.079

KHAN ZA 3 0.079

KIM CS 3 0.079

KIM DK 3 0.079

KIM DY 3 0.079

KIM EN 3 0.079

KIM H 3 0.079

KIM HK 3 0.079

KIM HM 3 0.079

KIM HR 3 0.079

KIM HY 3 0.079

KIM JG 3 0.079

KIM JL 3 0.079

KIM MJ 3 0.079

KIM MK 3 0.079

KIM SM 3 0.079

KIM W 3 0.079

KIM YH 3 0.079

KIMURA S 3 0.079

KITAJIMA S 3 0.079

KITAMURA H 3 0.079

KITCHING AR 3 0.079

KLEIN T 3 0.079

KO YA 3 0.079

KOBAYASHI H 3 0.079

KOBORI H 3 0.079

KODAMA M 3 0.079

KOMALA MG 3 0.079

KONG J 3 0.079

KONVALINKA A 3 0.079

KREPINSKY JC 3 0.079

KRUPA A 3 0.079

KULLAK-UBLICK GA 3 0.079

KUNDU A 3 0.079

KUROKAWA K 3 0.079

KWAK MK 3 0.079

KWON G 3 0.079

KWON MH 3 0.079

KWON SH 3 0.079

LAI FMM 3 0.079

LAI KB 3 0.079

LAM S 3 0.079

LANTING LL 3 0.079

LAVERMAN GD 3 0.079

LEADER JP 3 0.079

LEASK A 3 0.079

LEDBETTER SR 3 0.079

LEE BM 3 0.079

LEE CH 3 0.079

LEE EY 3 0.079

LEE G 3 0.079

LEE MJ 3 0.079

LEE YH 3 0.079

LEE YM 3 0.079

LEI CT 3 0.079

LEMLEY KV 3 0.079

LI BY 3 0.079

LI CC 3 0.079

LI G 3 0.079

LI HD 3 0.079

LI HZ 3 0.079

LI JZ 3 0.079

LI K 3 0.079

LI WP 3 0.079

LI ZG 3 0.079

LIANG XB 3 0.079

LIAO YJ 3 0.079

LILES JT 3 0.079

LIM Y 3 0.079

LINDENMEYER M 3 0.079

LING GH 3 0.079

LIU D 3 0.079

LIU GH 3 0.079

LIU JP 3 0.079

LIU JX 3 0.079

LIU KY 3 0.079

LIU LM 3 0.079

LIU SM 3 0.079

LIU SY 3 0.079

LIU XS 3 0.079

LIU XT 3 0.079

LIU YC 3 0.079

LIU YN 3 0.079

LIU YP 3 0.079

LIU ZZ 3 0.079

LLOBERAS N 3 0.079

LOGAN A 3 0.079

LORDAN JL 3 0.079

LORENZEN JM 3 0.079

LU M 3 0.079

LU WP 3 0.079

LU X 3 0.079

LU XG 3 0.079

LU ZY 3 0.079

LUCIA S 3 0.079

LUK CCW 3 0.079

LUNGKAPHIN A 3 0.079

LUO C 3 0.079

LUO JH 3 0.079

LUO YH 3 0.079

LV W 3 0.079

LV WS 3 0.079

LYONS KM 3 0.079

MA QY 3 0.079

MA TJ 3 0.079

MA TT 3 0.079

MAACHI H 3 0.079

MAESHIMA Y 3 0.079

MAESTRONI S 3 0.079

MALHEIROS DMAC 3 0.079

MANKHEY RW 3 0.079

MANO T 3 0.079

MARAI M 3 0.079

MARCUSSEN N 3 0.079

MARIC-BILKAN C 3 0.079

MARK PB 3 0.079

MARTIN-CLEARY C 3 0.079

MARTINI S 3 0.079

MAS S 3 0.079

MASON RM 3 0.079

MATSUMOTO M 3 0.079

MATUSZ P 3 0.079

MAYER B 3 0.079

MCKNIGHT AJ 3 0.079

MEACHERY G 3 0.079

MEAS-YEDID V 3 0.079

MEI CL 3 0.079

MEI WJ 3 0.079

MIHATSCH MJ 3 0.079

MILAS O 3 0.079

MILNE GT 3 0.079

MIRCESCU G 3 0.079

MISHRA R 3 0.079

MIYATA K 3 0.079

MIYAZAKI T 3 0.079

MOHAN M 3 0.079

MOHAN V 3 0.079

MORALES E 3 0.079

MORESO F 3 0.079

MORI M 3 0.079

MORIYA H 3 0.079

MOROTE J 3 0.079

MORRISEY K 3 0.079

MOU S 3 0.079

MOU X 3 0.079

MREICH E 3 0.079

MU W 3 0.079

MULLER CA 3 0.079

MULLER DN 3 0.079

MULLINS JJ 3 0.079

MUNOZ MC 3 0.079

MURATA I 3 0.079

MURUVE DA 3 0.079

MUSSO G 3 0.079

NAJARIAN JS 3 0.079

NAKANO D 3 0.079

NAKAYAMA M 3 0.079

NAM BY 3 0.079

NARITA T 3 0.079

NASR SH 3 0.079

NASRALLAH R 3 0.079

NEILSON EG 3 0.079

NGUYEN TV 3 0.079

NICHOLAS SB 3 0.079

NIE HB 3 0.079

NIKOLIC-PATERSON D 3 0.079

NINICHUK V 3 0.079

NISTALA R 3 0.079

NOCHY D 3 0.079

NOEL LH 3 0.079

NORMAN JT 3 0.079

OBERBAUER R 3 0.079

OGAWA Y 3 0.079

OH H 3 0.079

OH S 3 0.079

OHNO T 3 0.079

ORANGER A 3 0.079

ORCHARD T 3 0.079

OSMAN M 3 0.079

PACZEK L 3 0.079

PANG XX 3 0.079

PAPALE M 3 0.079

PARIKH CR 3 0.079

PARK ASD 3 0.079

PARRISH AR 3 0.079

PARRY G 3 0.079

PATSCHAN S 3 0.079

PAULUS WJ 3 0.079

PEDERSEN M 3 0.079

PEDRAZA-CHAVERRI J 3 0.079

PENG XF 3 0.079

PENG XR 3 0.079

PEREZ-GOMEZ MV 3 0.079

PERICO N 3 0.079

PETERSON R 3 0.079

PFLUEGER A 3 0.079

PILZ S 3 0.079

PINACH S 3 0.079

PIPPIN JW 3 0.079

PIRKLBAUER M 3 0.079

PLACIER S 3 0.079

POMPOSIELLO S 3 0.079

PONGCHAIDECHA A 3 0.079

PONTICELLI C 3 0.079

PONTRELLI P 3 0.079

POPESCU R 3 0.079

POWELL DW 3 0.079

POWER DA 3 0.079

PRAGA M 3 0.079

PRASAD PV 3 0.079

PRICE GM 3 0.079

PRICE GW 3 0.079

PRUZANSKI M 3 0.079

PUGLIESE G 3 0.079

PULAKAT L 3 0.079

PUSZTAI AM 3 0.079

PUTTA S 3 0.079

QI SS 3 0.079

QI XM 3 0.079

QIAN JC 3 0.079

RAHMAN K 3 0.079

RAKUGI H 3 0.079

RASTALDI MP 3 0.079

REICHETZEDER C 3 0.079

REINDERS MEJ 3 0.079

RICARDO SD 3 0.079

RICCI C 3 0.079

RITTER JK 3 0.079

RODRIGUES-DIEZ RR 3 0.079

ROESTENBERG P 3 0.079

RONCO C 3 0.079

RONG S 3 0.079

RONG XL 3 0.079

ROPS ALWMM 3 0.079

ROSSINI M 3 0.079

ROSTAING L 3 0.079

ROTTOLI D 3 0.079

ROXBURGH SA 3 0.079

ROZEN-ZVI B 3 0.079

RUAN XZ 3 0.079

RUSSO LM 3 0.079

SAHA A 3 0.079

SALTE K 3 0.079

SANCHEZ-LOZADA LG 3 0.079

SANTORO D 3 0.079

SANZ A 3 0.079

SARKOZI R 3 0.079

SARNO R 3 0.079

SASAKI K 3 0.079

SASAKI T 3 0.079

SATO E 3 0.079

SATO N 3 0.079

SATOH M 3 0.079

SAVAGE DA 3 0.079

SAWYER RT 3 0.079

SCHERZER P 3 0.079

SCHIFFRIN EL 3 0.079

SCHLONDORFF D 3 0.079

SCHNAPER HW 3 0.079

SCHNELLMANN RG 3 0.079

SCHWALM S 3 0.079

SECARA A 3 0.079

SECCIA TM 3 0.079

SEO JY 3 0.079

SERVAIS A 3 0.079

SETHI S 3 0.079

SHAH SV 3 0.079

SHAO ML 3 0.079

SHEA CM 3 0.079

SHENG JQ 3 0.079

SHI YJ 3 0.079

SHI YM 3 0.079

SHIBAGAKI Y 3 0.079

SHIKATA K 3 0.079

SHIMIZU T 3 0.079

SHIN D 3 0.079

SIMULESCU A 3 0.079

SINGH AK 3 0.079

SIRIN Y 3 0.079

SKILL NJ 3 0.079

SLATTERY C 3 0.079

SMITHIES O 3 0.079

SODERBERG M 3 0.079

SONG HK 3 0.079

SONG SH 3 0.079

SPARDING N 3 0.079

SRIVASTAVA A 3 0.079

STADLER K 3 0.079

STANTON RC 3 0.079

STECKELINGS UM 3 0.079

STEFANOVIC N 3 0.079

STOKES MB 3 0.079

STRUTZ F 3 0.079

SU DM 3 0.079

SU Z 3 0.079

SUGIMOTO H 3 0.079

SUGIMOTO T 3 0.079

SUGIYAMA H 3 0.079

SUN AL 3 0.079

SUN W 3 0.079

SUZUKI N 3 0.079

SZABO AJ 3 0.079

SZETO HH 3 0.079

TAGUCHI T 3 0.079

TAKAGAKI Y 3 0.079

TAKAHASHI H 3 0.079

TAKAHASHI K 3 0.079

TAKAHASHI Y 3 0.079

TAKASU T 3 0.079

TAKEI Y 3 0.079

TAKEMURA G 3 0.079

TAM J 3 0.079

TAMPE D 3 0.079

TAN RJ 3 0.079

TANABE K 3 0.079

TANAKA S 3 0.079

TANG RN 3 0.079

TANG WB 3 0.079

TANIMOTO M 3 0.079

TAO J 3 0.079

TAPIA E 3 0.079

THERVET E 3 0.079

THOMPSON RH 3 0.079

THONGBOONKERD V 3 0.079

TIAN HS 3 0.079

TIKOO K 3 0.079

TIMAR R 3 0.079

TOBA H 3 0.079

TOMINO Y 3 0.079

TORFFVIT O 3 0.079

TOYAMA T 3 0.079

TRILLA E 3 0.079

TRIONFINI P 3 0.079

TROXELL ML 3 0.079

TU Y 3 0.079

TUNG CW 3 0.079

UDDIN MJ 3 0.079

UNGER T 3 0.079

URSONIU S 3 0.079

VALDERRAMA G 3 0.079

VALDIVIELSO JM 3 0.079

VALERI AM 3 0.079

VAN ZONNEVELD AJ 3 0.079

VERHAGEN NAM 3 0.079

VIELHAUER V 3 0.079

VLAD A 3 0.079

VLAD D 3 0.079

VOORS AA 3 0.079

WAN YG 3 0.079

WANG CH 3 0.079

WANG CY 3 0.079

WANG FY 3 0.079

WANG JJ 3 0.079

WANG JN 3 0.079

WANG JX 3 0.079

WANG LL 3 0.079

WANG SD 3 0.079

WANG SL 3 0.079

WANG SW 3 0.079

WANG WD 3 0.079

WANG WJ 3 0.079

WANG WL 3 0.079

WANG XH 3 0.079

WANG XJ 3 0.079

WANG XX 3 0.079

WANG YN 3 0.079

WANG YP 3 0.079

WANG ZJ 3 0.079

WANG ZW 3 0.079

WANG ZY 3 0.079

WANNER C 3 0.079

WARD L 3 0.079

WATANABE M 3 0.079

WATANABE S 3 0.079

WATANABE T 3 0.079

WEENING JJ 3 0.079

WEI J 3 0.079

WEI JL 3 0.079

WEI T 3 0.079

WELLS CC 3 0.079

WEN XY 3 0.079

WHALEY-CONNELL A 3 0.079

WHITE KE 3 0.079

WIDOMSKI D 3 0.079

WILKINSON-BERKA JL 3 0.079

WILLIAMS SJ 3 0.079

WU B 3 0.079

WU BJ 3 0.079

WU LL 3 0.079

WU MJ 3 0.079

WU PF 3 0.079

WU TQ 3 0.079

WU W 3 0.079

WU WJ 3 0.079

WU XM 3 0.079

WU YY 3 0.079

XIAO HM 3 0.079

XIAO QF 3 0.079

XIAO TL 3 0.079

XIE L 3 0.079

XIE P 3 0.079

XIE Y 3 0.079

XIONG X 3 0.079

XU BJ 3 0.079

XU D 3 0.079

XU HH 3 0.079

XU JY 3 0.079

XU LP 3 0.079

XU LT 3 0.079

XU M 3 0.079

XU P 3 0.079

XU WF 3 0.079

XU XL 3 0.079

YABUKI A 3 0.079

YAMADA M 3 0.079

YAMAMOTO Y 3 0.079

YAMAZAKI S 3 0.079

YAN HD 3 0.079

YAN P 3 0.079

YAN Q 3 0.079

YANG G 3 0.079

YANG K 3 0.079

YANG R 3 0.079

YANG T 3 0.079

YANG YH 3 0.079

YANG YY 3 0.079

YAO D 3 0.079

YAO HK 3 0.079

YI B 3 0.079

YI TG 3 0.079

YOO TH 3 0.079

YU R 3 0.079

YU WH 3 0.079

YU XW 3 0.079

YUAN H 3 0.079

YUAN J 3 0.079

YUAN Q 3 0.079

YUAN XH 3 0.079

YUAN YY 3 0.079

YUEN DA 3 0.079

ZAMMIT SC 3 0.079

ZATZ R 3 0.079

ZEHNDER D 3 0.079

ZEISBERG EM 3 0.079

ZHANG AH 3 0.079

ZHANG CH 3 0.079

ZHANG FF 3 0.079

ZHANG GY 3 0.079

ZHANG HL 3 0.079

ZHANG JB 3 0.079

ZHANG JD 3 0.079

ZHANG JF 3 0.079

ZHANG JG 3 0.079

ZHANG K 3 0.079

ZHANG LL 3 0.079

ZHANG SP 3 0.079

ZHANG WK 3 0.079

ZHANG XJ 3 0.079

ZHANG YF 3 0.079

ZHANG YH 3 0.079

ZHANG YX 3 0.079

ZHAO BH 3 0.079

ZHAO HL 3 0.079

ZHAO M 3 0.079

ZHAO SL 3 0.079

ZHAO YL 3 0.079

ZHAO ZZ 3 0.079

ZHENG DX 3 0.079

ZHENG F 3 0.079

ZHOU D 3 0.079

ZHOU DY 3 0.079

ZHOU M 3 0.079

ZHOU QL 3 0.079

ZHOU SS 3 0.079

ZHOU TB 3 0.079

ZHOU XC 3 0.079

ZHOU XJ 3 0.079

ZHOU YH 3 0.079

ZHU B 3 0.079

ZHU HJ 3 0.079

ZHU J 3 0.079

ZHU YC 3 0.079

AA JY 2 0.052

ABASCAL JM 2 0.052

ABASSI Z 2 0.052

ABDULLA MA 2 0.052

ABDULLAH NA 2 0.052

ABE H 2 0.052

ABE T 2 0.052

ADACHI H 2 0.052

ADACHI M 2 0.052

AGHADAVOD E 2 0.052

AGODOA L 2 0.052

AHN SH 2 0.052

AITKEN ML 2 0.052

AKDENIZ A 2 0.052

AKDENIZ T 2 0.052

AKHTAR S 2 0.052

AKIZAWA T 2 0.052

AKTER S 2 0.052

AL SALAM S 2 0.052

AL SULEIMANI Y 2 0.052

AL-ASSAF S 2 0.052

ALBERT A 2 0.052

ALEXANDER GJM 2 0.052

ALEXANDER SI 2 0.052

ALHENC-GELAS F 2 0.052

ALI BH 2 0.052

ALIQUE M 2 0.052

ALPEROVICH G 2 0.052

ALTER ML 2 0.052

ALVAREZ ML 2 0.052

AN Y 2 0.052

ANDERSON PW 2 0.052

ANDREUCCI M 2 0.052

ANGLANI F 2 0.052

ANKER SD 2 0.052

ANNARATONE L 2 0.052

ANTOINE MH 2 0.052

ARAKI SI 2 0.052

ARDILES L 2 0.052

ARELLANO-BUENDIA AS 2 0.052

ARTHUR JM 2 0.052

ARTUNC F 2 0.052

ARUMUGAM S 2 0.052

ASAKURA M 2 0.052

ASANO T 2 0.052

ASANO Y 2 0.052

ASICO L 2 0.052

ASICO LD 2 0.052

ASSAF N 2 0.052

ASTOR BC 2 0.052

ATEF H 2 0.052

AUCOUTURIER P 2 0.052

AUSTIN PF 2 0.052

AVCI E 2 0.052

AWAD AS 2 0.052

AZUMA J 2 0.052

BABAZONO T 2 0.052

BACH LA 2 0.052

BAELDE JJ 2 0.052

BAGGIO B 2 0.052

BAGNASCO SM 2 0.052

BAI F 2 0.052

BAI H 2 0.052

BAID-AGRAWAL S 2 0.052

BAILEY MA 2 0.052

BAIN SC 2 0.052

BAJEMA IM 2 0.052

BAKER MA 2 0.052

BALASUBRAMANYAM M 2 0.052

BALDE N 2 0.052

BALOGH DB 2 0.052

BANIJAMALI AR 2 0.052

BANKER ME 2 0.052

BAO BY 2 0.052

BARBARI A 2 0.052

BARBERATO SH 2 0.052

BARISONI L 2 0.052

BARRY M 2 0.052

BASTACKY S 2 0.052

BAUM P 2 0.052

BAUMANN M 2 0.052

BAYRAKTAR F 2 0.052

BECK S 2 0.052

BECKER JU 2 0.052

BECKMANN J 2 0.052

BEHULIAK M 2 0.052

BEIGE J 2 0.052

BELLINI G 2 0.052

BELTON O 2 0.052

BENEDIKTSSON H 2 0.052

BENITO-MARTIN A 2 0.052

BENYAJATI S 2 0.052

BERDEN JHM 2 0.052

BERENGUER J 2 0.052

BERNAL S 2 0.052

BERNARDI S 2 0.052

BERNIER SG 2 0.052

BERNIS C 2 0.052

BERNOBICH E 2 0.052

BERTELSEN LB 2 0.052

BERTINI A 2 0.052

BETTAIEB A 2 0.052

BETZ B 2 0.052

BEUSCART O 2 0.052

BHATT K 2 0.052

BHOWMICK S 2 0.052

BHREATHNACH U 2 0.052

BHUIYAN AS 2 0.052

BIAN Q 2 0.052

BIAN XH 2 0.052

BIANCHI M 2 0.052

BIRNER C 2 0.052

BLEICH M 2 0.052

BLOKZIJL H 2 0.052

BLOM IE 2 0.052

BLOUIN K 2 0.052

BOBADILLA NA 2 0.052

BODHANKAR SL 2 0.052

BOHLE A 2 0.052

BOLANOS N 2 0.052

BOLTON WK 2 0.052

BOMBARDI C 2 0.052

BONEGIO R 2 0.052

BORG DJ 2 0.052

BORGES FT 2 0.052

BOSCARINO JA 2 0.052

BOTTINGER EP 2 0.052

BOULANGER E 2 0.052

BOUSTANY-KARI CM 2 0.052

BOVENSCHEN N 2 0.052

BOWEN T 2 0.052

BRAET F 2 0.052

BRANDES RP 2 0.052

BRAUNWALD E 2 0.052

BRENCHLEY PEC 2 0.052

BREYER JA 2 0.052

BREYER MD 2 0.052

BREZNICEANU ML 2 0.052

BRINKKOETTER PT 2 0.052

BRIZZI MF 2 0.052

BROEKHUIZEN R 2 0.052

BROWN NJ 2 0.052

BRUNSKILL NJ 2 0.052

BRUSCHI M 2 0.052

BUCHLER M 2 0.052

BUDD DC 2 0.052

BUELLI S 2 0.052

BUIKEMA H 2 0.052

BULEON M 2 0.052

BURGESS DS 2 0.052

BURHAN I 2 0.052

BURKE GW 2 0.052

BUTTER LM 2 0.052

CACACE A 2 0.052

CAGLAYAN B 2 0.052

CAI GY 2 0.052

CAI J 2 0.052

CAI JJ 2 0.052

CAI T 2 0.052

CAI YY 2 0.052

CALKIN AC 2 0.052

CALLERA GE 2 0.052

CAMARA NOS 2 0.052

CAMPBELL F 2 0.052

CAMPBELL KN 2 0.052

CAMPESE VM 2 0.052

CAMPOS AH 2 0.052

CANG Z 2 0.052

CAO AL 2 0.052

CAO QH 2 0.052

CAO X 2 0.052

CAO YZ 2 0.052

CAPASSO G 2 0.052

CAROCCIA B 2 0.052

CARPENTER AJ 2 0.052

CARR JC 2 0.052

CAUBET C 2 0.052

CAUFFIEZ C 2 0.052

CAVAGLIERI RC 2 0.052

CELEC P 2 0.052

CERNARO V 2 0.052

CHA JH 2 0.052

CHA JJ 2 0.052

CHACHIN M 2 0.052

CHADE AR 2 0.052

CHAGNAC A 2 0.052

CHAN GCW 2 0.052

CHAN JCN 2 0.052

CHAN SC 2 0.052

CHAN TM 2 0.052

CHANG C 2 0.052

CHANG HR 2 0.052

CHANG YK 2 0.052

CHANG YP 2 0.052

CHAPMAN JR 2 0.052

CHATSUDTHIPONG V 2 0.052

CHATTIPAKORN N 2 0.052

CHAUDHARI S 2 0.052

CHAUDHARY K 2 0.052

CHEN A 2 0.052

CHEN CC 2 0.052

CHEN GJ 2 0.052

CHEN HL 2 0.052

CHEN JL 2 0.052

CHEN JW 2 0.052

CHEN JY 2 0.052

CHEN M 2 0.052

CHEN MJ 2 0.052

CHEN ML 2 0.052

CHEN PC 2 0.052

CHEN PY 2 0.052

CHEN QJ 2 0.052

CHEN QQ 2 0.052

CHEN SX 2 0.052

CHEN TH 2 0.052

CHEN WD 2 0.052

CHEN XJ 2 0.052

CHEN XP 2 0.052

CHEN XQ 2 0.052

CHEN XW 2 0.052

CHEN YH 2 0.052

CHEN YW 2 0.052

CHEN YX 2 0.052

CHEN ZJ 2 0.052

CHENG AC 2 0.052

CHENG C 2 0.052

CHENG CY 2 0.052

CHENG D 2 0.052

CHENG GY 2 0.052

CHENG LJ 2 0.052

CHENG PMS 2 0.052

CHENG W 2 0.052

CHENG XY 2 0.052

CHENG YQ 2 0.052

CHEW P 2 0.052

CHI YF 2 0.052

CHIEN YT 2 0.052

CHIN HJ 2 0.052

CHIU YW 2 0.052

CHO A 2 0.052

CHO BS 2 0.052

CHO KH 2 0.052

CHOE MS 2 0.052

CHOI BH 2 0.052

CHOI DE 2 0.052

CHOI ES 2 0.052

CHOI P 2 0.052

CHOI YH 2 0.052

CHOI YJ 2 0.052

CHOONG YS 2 0.052

CHOU JY 2 0.052

CHOW BSM 2 0.052

CHUANG CT 2 0.052

CHUANG PY 2 0.052

CIANCIO G 2 0.052

CIBULSKYTE D 2 0.052

CINAR R 2 0.052

CLARK S 2 0.052

CLAUSS S 2 0.052

CLEVELAND WH 2 0.052

COBAN J 2 0.052

COBBS A 2 0.052

COCA SG 2 0.052

COCKWELL P 2 0.052

COHNEY S 2 0.052

COLLINS JD 2 0.052

COLOMBO C 2 0.052

COMPER WD 2 0.052

CONG WT 2 0.052

CONGET P 2 0.052

CONTI DJ 2 0.052

CONTI F 2 0.052

CONTI G 2 0.052

CONTI S 2 0.052

CONVENTO MB 2 0.052

CONWAY SP 2 0.052

COOPER GJS 2 0.052

CORDASIC N 2 0.052

CORNISH TC 2 0.052

CORREA JW 2 0.052

COSENZI A 2 0.052

COSTA S 2 0.052

COVIC A 2 0.052

COX A 2 0.052

COX RD 2 0.052

COZZOLINO M 2 0.052

CRETU O 2 0.052

CRUZ PE 2 0.052

CUI FQ 2 0.052

CUI Y 2 0.052

CUMMINS TD 2 0.052

CUNARD R 2 0.052

CUSI D 2 0.052

D'HAESE PC 2 0.052

DABELEA D 2 0.052

DAI WD 2 0.052

DAIDA YG 2 0.052

DANG WY 2 0.052

DANTAS M 2 0.052

DARGIE HJ 2 0.052

DARK JH 2 0.052

DARLEY-USMAR V 2 0.052

DAROUX M 2 0.052

DAVARPANAH AH 2 0.052

DAVIS B 2 0.052

DAVIS G 2 0.052

DAY RT 2 0.052

DE BLASIO MJ 2 0.052

DE BOER IH 2 0.052

DE BORST MH 2 0.052

DE CAESTECKER MP 2 0.052

DE CHIARA L 2 0.052

DE FEO V 2 0.052

DE FRUTOS S 2 0.052

DE LA MOTTE C 2 0.052

DE MICHIELI F 2 0.052

DE ZEEUW D 2 0.052

DEAN RG 2 0.052

DEEG M 2 0.052

DEELMAN LE 2 0.052

DEL PRETE D 2 0.052

DELANGHE JR 2 0.052

DELCAYRE C 2 0.052

DELIC D 2 0.052

DEMIR T 2 0.052

DENBY L 2 0.052

DENG YY 2 0.052

DENG ZY 2 0.052

DENICHILO M 2 0.052

DENIS C 2 0.052

DEREGIBUS MC 2 0.052

DEROSA G 2 0.052

DEROUICHE S 2 0.052

DEVARAPU SK 2 0.052

DEVUYST O 2 0.052

DI BELLO V 2 0.052

DING CG 2 0.052

DING JL 2 0.052

DING T 2 0.052

DING XM 2 0.052

DING Y 2 0.052

DISTEFANO JK 2 0.052

DIXON A 2 0.052

DOBRINSKIKH E 2 0.052

DOCKRELL MEC 2 0.052

DOI S 2 0.052

DOLAN V 2 0.052

DONAGHUE KC 2 0.052

DONG L 2 0.052

DONG LC 2 0.052

DONG Q 2 0.052

DONG WP 2 0.052

DONG XY 2 0.052

DONG Y 2 0.052

DONG ZF 2 0.052

DOS SANTOS M 2 0.052

DOUNOUSI E 2 0.052

DOWLING J 2 0.052

DRECHSLER C 2 0.052

DRORI A 2 0.052

DU CY 2 0.052

DU XG 2 0.052

DU YC 2 0.052

DU YM 2 0.052

DU YX 2 0.052

DUAN XJ 2 0.052

DUAN YY 2 0.052

DUBOURG L 2 0.052

DUCHAMPT A 2 0.052

DULAK J 2 0.052

DULLAART RPF 2 0.052

DUNBAR DR 2 0.052

DUNBAR PR 2 0.052

DUNCKER DJ 2 0.052

DUNI A 2 0.052

DUNN TB 2 0.052

DURAND D 2 0.052

DURLIK M 2 0.052

DURUPT S 2 0.052

DVORNIKOV A 2 0.052

DWYER KM 2 0.052

ECELBARGER CM 2 0.052

EDDY S 2 0.052

EDENHOFER I 2 0.052

EID S 2 0.052

EIRIN A 2 0.052

EITNER F 2 0.052

EKINCI EI 2 0.052

EL NAHAS M 2 0.052

EL-OSTA A 2 0.052

EL-YAMANY MF 2 0.052

ELLER J 2 0.052

ELSEWEIDY MM 2 0.052

EMMETT N 2 0.052

EREN Z 2 0.052

ESAKI M 2 0.052

ESCANO CS 2 0.052

ESQUENA S 2 0.052

ESTEBAN V 2 0.052

FALCAO-PIRES I 2 0.052

FAN CX 2 0.052

FAN D 2 0.052

FAN J 2 0.052

FAN JJ 2 0.052

FAN XF 2 0.052

FAN XM 2 0.052

FAN YY 2 0.052

FAN Z 2 0.052

FANELLI C 2 0.052

FANG S 2 0.052

FANG YD 2 0.052

FARRELL FX 2 0.052

FEIGERLOVA E 2 0.052

FELDMAN HI 2 0.052

FELLSTROM BC 2 0.052

FENG J 2 0.052

FENG LH 2 0.052

FENG LX 2 0.052

FENG YL 2 0.052

FENG YX 2 0.052

FENG ZD 2 0.052

FENG ZG 2 0.052

FENNING A 2 0.052

FERRARIO CM 2 0.052

FERRAZ MLG 2 0.052

FERRI C 2 0.052

FERVENZA FC 2 0.052

FEUTREN G 2 0.052

FILIPOVIC N 2 0.052

FILLAT C 2 0.052

FLAQUER M 2 0.052

FONSECA VA 2 0.052

FORD BM 2 0.052

FOTHERINGHAM AK 2 0.052

FRANCOS B 2 0.052

FRANKE S 2 0.052

FRANQUESA M 2 0.052

FRASER DJ 2 0.052

FREDERSDORF S 2 0.052

FRIDELL JA 2 0.052

FRIED LF 2 0.052

FRIEDLI I 2 0.052

FRIEDRICH B 2 0.052

FUJITA K 2 0.052

FUJITA S 2 0.052

FUKUDA K 2 0.052

FUKUHARA S 2 0.052

FUTTERWEIT S 2 0.052

GABBIANI G 2 0.052

GACIONG Z 2 0.052

GALAS D 2 0.052

GALLAZZINI M 2 0.052

GALLO LA 2 0.052

GAMBA G 2 0.052

GAN XX 2 0.052

GANESAN D 2 0.052

GAO BH 2 0.052

GAO C 2 0.052

GAO JL 2 0.052

GAO Q 2 0.052

GAO SH 2 0.052

GAO X 2 0.052

GAO XY 2 0.052

GAO YH 2 0.052

GARCIA-ARROYO FE 2 0.052

GARG N 2 0.052

GARIMELLA PS 2 0.052

GARMAN JH 2 0.052

GARUD MS 2 0.052

GE HT 2 0.052

GE Y 2 0.052

GEJYO F 2 0.052

GERALDES P 2 0.052

GERMANO P 2 0.052

GESCHWINDNER S 2 0.052

GHAHRAMANI N 2 0.052

GHAYUR A 2 0.052

GHONEIM HA 2 0.052

GHOSH A 2 0.052

GILL GV 2 0.052

GILLINGHAM KJ 2 0.052

GIMENO J 2 0.052

GIORGI D 2 0.052

GIORGINO F 2 0.052

GIROLAMI JP 2 0.052

GIUSTI C 2 0.052

GJORGJIEVA M 2 0.052

GLASSOCK R 2 0.052

GLASTRAS SJ 2 0.052

GLOWACKI F 2 0.052

GLUCK C 2 0.052

GLUHOVSCHI G 2 0.052

GODO M 2 0.052

GOLDBERG IJ 2 0.052

GOLDSMITH D 2 0.052

GONDALIYA P 2 0.052

GONG ZC 2 0.052

GONZALEZ AA 2 0.052

GONZALEZ-CADAVID NF 2 0.052

GORDON JR 2 0.052

GOSS E 2 0.052

GOTO T 2 0.052

GOU R 2 0.052

GOW RM 2 0.052

GRAHAM-BROWN MPM 2 0.052

GRANGE C 2 0.052

GRANQVIST AB 2 0.052

GRATTON E 2 0.052

GRAUL R 2 0.052

GRAY SP 2 0.052

GREENE EL 2 0.052

GREENE T 2 0.052

GREGOREVIC P 2 0.052

GRIERA M 2 0.052

GRIFFIN MD 2 0.052

GRIMALDI S 2 0.052

GRONBAEK H 2 0.052

GRUNFELD JP 2 0.052

GRUZDEV A 2 0.052

GU CM 2 0.052

GU JL 2 0.052

GU YH 2 0.052

GU YY 2 0.052

GUAN X 2 0.052

GUAN XF 2 0.052

GUIRY PJ 2 0.052

GUL R 2 0.052

GUNAL MY 2 0.052

GUNGOR G 2 0.052

GUO CC 2 0.052

GUO CY 2 0.052

GUO JM 2 0.052

GUO L 2 0.052

GUO QS 2 0.052

GUO TT 2 0.052

GUO YY 2 0.052

GUPTA A 2 0.052

GUPTA P 2 0.052

GURLEY SB 2 0.052

GUZMAN NJ 2 0.052

HADAR R 2 0.052

HADJILIADIS D 2 0.052

HAHNEL B 2 0.052

HAIMBACH R 2 0.052

HAJ FG 2 0.052

HALIMI JM 2 0.052

HALLER VM 2 0.052

HALLOWS KR 2 0.052

HALUSHKA MK 2 0.052

HAMASAKI Y 2 0.052

HAMMOCK BD 2 0.052

HAN B 2 0.052

HAN M 2 0.052

HAN XT 2 0.052

HAN Y 2 0.052

HAN YC 2 0.052

HANSEN HE 2 0.052

HANSEN TW 2 0.052

HARA A 2 0.052

HARADA M 2 0.052

HARIMA M 2 0.052

HARRIS DCH 2 0.052

HARTNER A 2 0.052

HASAN AA 2 0.052

HASAN Q 2 0.052

HASCALL V 2 0.052

HATTERSLEY A 2 0.052

HAUSKNECHT B 2 0.052

HAUSWIRTH WW 2 0.052

HAWES JW 2 0.052

HAYASHI K 2 0.052

HAYASHI T 2 0.052

HAYASHIDA T 2 0.052

HAYATA M 2 0.052

HAYLOR JL 2 0.052

HAZUE R 2 0.052

HE D 2 0.052

HE FF 2 0.052

HE JL 2 0.052

HE L 2 0.052

HE SM 2 0.052

HE X 2 0.052

HE XY 2 0.052

HE YY 2 0.052

HE ZK 2 0.052

HEEMANN U 2 0.052

HELLBERG O 2 0.052

HEMMI N 2 0.052

HENG C 2 0.052

HENLE T 2 0.052

HENNING RH 2 0.052

HENRIKSEN K 2 0.052

HENZE E 2 0.052

HERMAN JG 2 0.052

HEUCHEL RL 2 0.052

HEYMANS S 2 0.052

HICKEY FB 2 0.052

HICKSON LJ 2 0.052

HIGGINS CE 2 0.052

HIGGINS GC 2 0.052

HILLER C 2 0.052

HILLIARD LM 2 0.052

HINAMOTO N 2 0.052

HIRATA K 2 0.052

HIRATA M 2 0.052

HITOMI H 2 0.052

HO JE 2 0.052

HODGIN JB 2 0.052

HODREA J 2 0.052

HOEK M 2 0.052

HOHENSTEIN B 2 0.052

HOLMBERG SD 2 0.052

HOLZMAN LB 2 0.052

HONDA K 2 0.052

HONG SW 2 0.052

HORI M 2 0.052

HORINO T 2 0.052

HORITA S 2 0.052

HORIUCHI S 2 0.052

HOSOMI N 2 0.052

HOSSER H 2 0.052

HOSSZU A 2 0.052

HOSTETTER TH 2 0.052

HOTTER G 2 0.052

HOU J 2 0.052

HOU T 2 0.052

HOUGHTON DC 2 0.052

HRUSKA K 2 0.052

HSIEH PF 2 0.052

HSIEH YC 2 0.052

HSU CT 2 0.052

HSU HH 2 0.052

HSU MF 2 0.052

HU C 2 0.052

HU CP 2 0.052

HU CY 2 0.052

HU JC 2 0.052

HU MS 2 0.052

HU QS 2 0.052

HU SW 2 0.052

HU W 2 0.052

HU XF 2 0.052

HU YL 2 0.052

HU ZX 2 0.052

HUANG CC 2 0.052

HUANG CM 2 0.052

HUANG D 2 0.052

HUANG LJ 2 0.052

HUANG LL 2 0.052

HUANG Q 2 0.052

HUANG RB 2 0.052

HUANG SH 2 0.052

HUANG SM 2 0.052

HUANG SZ 2 0.052

HUANG TC 2 0.052

HUANG X 2 0.052

HUANG YH 2 0.052

HUANG YP 2 0.052

HUANG YW 2 0.052

HUANG Z 2 0.052

HUANG ZF 2 0.052

HUANG ZJ 2 0.052

HUANG ZM 2 0.052

HUBNER A 2 0.052

HUGHSON MD 2 0.052

HUGO C 2 0.052

HUGO CPM 2 0.052

HUMPHREYS BD 2 0.052

HUNG CN 2 0.052

HUSSAIN A 2 0.052

HUSSON C 2 0.052

HUTCHENS ZM 2 0.052

HUYNH P 2 0.052

HWANG I 2 0.052

HWANG SD 2 0.052

HYODO N 2 0.052

IBRAHIM HN 2 0.052

ICHIHARA A 2 0.052

ICHII O 2 0.052

ICHIKAWA D 2 0.052

IGLESIAS-DE LA CRUZ MC 2 0.052

IHM CG 2 0.052

IIDA M 2 0.052

IJPELAAR DHT 2 0.052

IKEDA K 2 0.052

IMAFUKU A 2 0.052

IMAI N 2 0.052

IMAMAKI H 2 0.052

INGRAM AJ 2 0.052

INSERRA F 2 0.052

ISHIGAMI A 2 0.052

ISMAIL-BEIGI F 2 0.052

ITANO S 2 0.052

ITO H 2 0.052

ITO K 2 0.052

ITOH M 2 0.052

IWAHASHI M 2 0.052

IWAMOTO Y 2 0.052

IWAO H 2 0.052

IYER A 2 0.052

JACOB HJ 2 0.052

JACOBSON S 2 0.052

JACQUOT C 2 0.052

JADHAV SS 2 0.052

JAFFA AA 2 0.052

JAIKUMKAO K 2 0.052

JAIN M 2 0.052

JANSSEN U 2 0.052

JAQUET V 2 0.052

JARAMILLO C 2 0.052

JARDINE AG 2 0.052

JAYACHANDRAN I 2 0.052

JEFFERIES C 2 0.052

JENA G 2 0.052

JENSEN BL 2 0.052

JEON JH 2 0.052

JEON JS 2 0.052

JEONG HY 2 0.052

JEONG JY 2 0.052

JEONG KH 2 0.052

JEONG LS 2 0.052

JETTEN AM 2 0.052

JI H 2 0.052

JI XJ 2 0.052

JIA DX 2 0.052

JIA M 2 0.052

JIA WJ 2 0.052

JIA X 2 0.052

JIANG HW 2 0.052

JIANG HX 2 0.052

JIANG LH 2 0.052

JIANG N 2 0.052

JIANG WH 2 0.052

JIANG XH 2 0.052

JIANG XS 2 0.052

JIANG YP 2 0.052

JIANG ZZ 2 0.052

JIANU DC 2 0.052

JIN H 2 0.052

JIN L 2 0.052

JIN LB 2 0.052

JIN LT 2 0.052

JIN SZ 2 0.052

JIN YL 2 0.052

JO K 2 0.052

JOARDAR S 2 0.052

JOHNSON JH 2 0.052

JOHNSON MS 2 0.052

JONES C 2 0.052

JONES JE 2 0.052

JONES SE 2 0.052

JOSHUA IG 2 0.052

JOZKOWICZ A 2 0.052

JU DW 2 0.052

JU YH 2 0.052

JUNG HJ 2 0.052

KA SM 2 0.052

KACHRU N 2 0.052

KADOYA H 2 0.052

KAIMORI JY 2 0.052

KAKEI M 2 0.052

KAKIZOE Y 2 0.052

KALIA K 2 0.052

KAN WC 2 0.052

KAN YQ 2 0.052

KANBAY M 2 0.052

KANDASWAMY R 2 0.052

KANG DH 2 0.052

KANG HS 2 0.052

KANG JS 2 0.052

KANG L 2 0.052

KANG P 2 0.052

KANNO Y 2 0.052

KANTARCI G 2 0.052

KASHYAP S 2 0.052

KASSIM NM 2 0.052

KATAGIRI D 2 0.052

KATAYAMA S 2 0.052

KATO N 2 0.052

KATO S 2 0.052

KATO Y 2 0.052

KATOH M 2 0.052

KATSUNO T 2 0.052

KATTA K 2 0.052

KATTLA JJ 2 0.052

KAUTER K 2 0.052

KAVANAGH DH 2 0.052

KAVUKCU S 2 0.052

KAWADA M 2 0.052

KAWADA T 2 0.052

KAWAGUCHI T 2 0.052

KELLEY DE 2 0.052

KELLY D 2 0.052

KERN TS 2 0.052

KES P 2 0.052

KETTELER M 2 0.052

KHAIROUN M 2 0.052

KHAN MAH 2 0.052

KHAZIM K 2 0.052

KIDOKORO K 2 0.052

KILIC E 2 0.052

KIM B 2 0.052

KIM D 2 0.052

KIM IS 2 0.052

KIM IY 2 0.052

KIM M 2 0.052

KIM NH 2 0.052

KIM SH 2 0.052

KIM SJ 2 0.052

KIM SR 2 0.052

KIM SW 2 0.052

KIM YW 2 0.052

KIM-MITSUYAMA S 2 0.052

KIMMEL PL 2 0.052

KIMURA H 2 0.052

KIMURA N 2 0.052

KINASHI H 2 0.052

KING GL 2 0.052

KIRK KA 2 0.052

KIRKLAND JL 2 0.052

KIRSCH T 2 0.052

KISS LP 2 0.052

KITAMURA K 2 0.052

KLANKE B 2 0.052

KLIMONTOV VV 2 0.052

KNOWLER WC 2 0.052

KO JY 2 0.052

KOBARA H 2 0.052

KOBARA M 2 0.052

KOBAYASHI T 2 0.052

KOENERS MP 2 0.052

KOENTGEN F 2 0.052

KOEPSELL H 2 0.052

KOH P 2 0.052

KOIKE K 2 0.052

KOIKE S 2 0.052

KOJIMA N 2 0.052

KOK HM 2 0.052

KOLB M 2 0.052

KOLLING M 2 0.052

KOMADA T 2 0.052

KONDO Y 2 0.052

KONOMI A 2 0.052

KORBUT AI 2 0.052

KORRAPATI MC 2 0.052

KOSAI K 2 0.052

KOSTIC S 2 0.052

KOSZEGI S 2 0.052

KOWALEWSKA J 2 0.052

KOZAWA E 2 0.052

KRALL P 2 0.052

KRAMER AB 2 0.052

KRAMER BK 2 0.052

KRAUSE-RELLE K 2 0.052

KRIPPNER G 2 0.052

KRISHNAKUMAR A 2 0.052

KRIZ W 2 0.052

KU E 2 0.052

KU SK 2 0.052

KUBIK A 2 0.052

KULKARNI O 2 0.052

KUMAR A 2 0.052

KUMAR N 2 0.052

KUMAR S 2 0.052

KUMARI M 2 0.052

KUNDU S 2 0.052

KURUMATANI H 2 0.052

KUSEK JW 2 0.052

KUSUNOKI H 2 0.052

KWAN T 2 0.052

KWON TH 2 0.052

LACQUANITI A 2 0.052

LAI JN 2 0.052

LAI JY 2 0.052

LAI LL 2 0.052

LAI MN 2 0.052

LAKSHMANAN AP 2 0.052

LAPPIN DWP 2 0.052

LARSSON E 2 0.052

LASKOWSKI A 2 0.052

LASSILA M 2 0.052

LAUSTSEN C 2 0.052

LAVIOLA L 2 0.052

LAWRENCE DA 2 0.052

LAWSON JS 2 0.052

LEBLEU V 2 0.052

LEBRANCHU Y 2 0.052

LECRU L 2 0.052

LEDBETTER S 2 0.052

LEDET T 2 0.052

LEE A 2 0.052

LEE BH 2 0.052

LEE CI 2 0.052

LEE DW 2 0.052

LEE DY 2 0.052

LEE EJ 2 0.052

LEE HW 2 0.052

LEE JA 2 0.052

LEE JY 2 0.052

LEE KW 2 0.052

LEE M 2 0.052

LEE SB 2 0.052

LEE SJ 2 0.052

LEE SR 2 0.052

LEE TW 2 0.052

LEE VS 2 0.052

LEGENDRE C 2 0.052

LEI JS 2 0.052

LEI L 2 0.052

LEI YT 2 0.052

LEIBOVICH BC 2 0.052

LEIBOWITZ G 2 0.052

LEIERER J 2 0.052

LEITE-MOREIRA AF 2 0.052

LEITGES M 2 0.052

LENART L 2 0.052

LEONG-POI H 2 0.052

LERMAN A 2 0.052

LEVI J 2 0.052

LEWIS A 2 0.052

LEWIS L 2 0.052

LEYS L 2 0.052

LI AL 2 0.052

LI CH 2 0.052

LI CX 2 0.052

LI CZ 2 0.052

LI D 2 0.052

LI DD 2 0.052

LI DX 2 0.052

LI FG 2 0.052

LI GS 2 0.052

LI HX 2 0.052

LI LH 2 0.052

LI LP 2 0.052

LI LS 2 0.052

LI LX 2 0.052

LI PL 2 0.052

LI SS 2 0.052

LI TT 2 0.052

LI WH 2 0.052

LI WX 2 0.052

LI WY 2 0.052

LI XB 2 0.052

LI XR 2 0.052

LI XS 2 0.052

LI XW 2 0.052

LI XX 2 0.052

LI YG 2 0.052

LI YR 2 0.052

LI YS 2 0.052

LI ZL 2 0.052

LIAKOPOULOS V 2 0.052

LIAN JD 2 0.052

LIANG B 2 0.052

LIANG K 2 0.052

LIANG MY 2 0.052

LIANG XJ 2 0.052

LIANG XL 2 0.052

LIAO TD 2 0.052

LIAO YH 2 0.052

LIM AI 2 0.052

LIM BJ 2 0.052

LIM CS 2 0.052

LIN CC 2 0.052

LIN CY 2 0.052

LIN HC 2 0.052

LIN HL 2 0.052

LIN HY 2 0.052

LIN JG 2 0.052

LIN JW 2 0.052

LIN KD 2 0.052

LIN L 2 0.052

LIN LY 2 0.052

LIN MY 2 0.052

LIN MZ 2 0.052

LIN PH 2 0.052

LIN Q 2 0.052

LIN S 2 0.052

LIN SL 2 0.052

LIN SY 2 0.052

LIN TY 2 0.052

LIN Y 2 0.052

LIN YC 2 0.052

LIN YH 2 0.052

LIN ZM 2 0.052

LINDSCHAU C 2 0.052

LING H 2 0.052

LIU B 2 0.052

LIU CT 2 0.052

LIU DY 2 0.052

LIU GY 2 0.052

LIU JF 2 0.052

LIU JL 2 0.052

LIU JT 2 0.052

LIU K 2 0.052

LIU KK 2 0.052

LIU MD 2 0.052

LIU MJ 2 0.052

LIU PH 2 0.052

LIU QJ 2 0.052

LIU RJ 2 0.052

LIU RX 2 0.052

LIU SJ 2 0.052

LIU TH 2 0.052

LIU W 2 0.052

LIU XF 2 0.052

LIU XG 2 0.052

LIU XN 2 0.052

LIU XR 2 0.052

LIU XY 2 0.052

LIU YM 2 0.052

LIU YX 2 0.052

LIU YY 2 0.052

LIU YZ 2 0.052

LIU ZW 2 0.052

LIU ZY 2 0.052

LIVINGSTON MJ 2 0.052

LIVINGSTON NJ 2 0.052

LIZOTTE F 2 0.052

LOBODA A 2 0.052

LOCATELLI F 2 0.052

LOCATELLI M 2 0.052

LOH YW 2 0.052

LOPEZ-FRANCO O 2 0.052

LOPEZ-GUISA JM 2 0.052

LOPEZ-PARRA V 2 0.052

LOPEZ-SANZ L 2 0.052

LOREDO-MENDOZA ML 2 0.052

LORENZO O 2 0.052

LORIA P 2 0.052

LOU TQ 2 0.052

LOU Y 2 0.052

LOVETT DH 2 0.052

LU CL 2 0.052

LU HB 2 0.052

LU L 2 0.052

LU LM 2 0.052

LU MX 2 0.052

LU QL 2 0.052

LU S 2 0.052

LU SY 2 0.052

LU YC 2 0.052

LU YH 2 0.052

LUAN JY 2 0.052

LUCHNER A 2 0.052

LUNO J 2 0.052

LUO CW 2 0.052

LUO L 2 0.052

LUO LT 2 0.052

LUO N 2 0.052

LUO Q 2 0.052

LUO R 2 0.052

LUO RN 2 0.052

LUTZ J 2 0.052

LV ZM 2 0.052

MA C 2 0.052

MA F 2 0.052

MA FZ 2 0.052

MA JF 2 0.052

MA L 2 0.052

MA M 2 0.052

MA R 2 0.052

MA TK 2 0.052

MA TKW 2 0.052

MA XJ 2 0.052

MA YH 2 0.052

MA YL 2 0.052

MACCONI D 2 0.052

MADSEN M 2 0.052

MAFFEI P 2 0.052

MAGAZZU G 2 0.052

MAHADEVAN N 2 0.052

MAINBOURG S 2 0.052

MAKRIYANNIS A 2 0.052

MALGORZEWICZ S 2 0.052

MANDACHE E 2 0.052

MANICKAM N 2 0.052

MANN J 2 0.052

MANNA P 2 0.052

MANNING JR 2 0.052

MANOJ P 2 0.052

MANRIQUE-ACEVEDO CM 2 0.052

MANSFIELD BC 2 0.052

MANSON SR 2 0.052

MAO ZM 2 0.052

MARCH DS 2 0.052

MARCHANT V 2 0.052

MARGETTS PJ 2 0.052

MARKO L 2 0.052

MARRE M 2 0.052

MARTIN FIR 2 0.052

MARTIN J 2 0.052

MARTIN M 2 0.052

MARTIN P 2 0.052

MARTIN RS 2 0.052

MARTINI A 2 0.052

MARUYAMA R 2 0.052

MARUYAMA S 2 0.052

MARZ W 2 0.052

MATAS AJ 2 0.052

MATAVELLI LC 2 0.052

MATHEW J 2 0.052

MATSUI T 2 0.052

MATSUMARU T 2 0.052

MATSUMURA Y 2 0.052

MATSUSAKA T 2 0.052

MAYER-HAMBLETT N 2 0.052

MCAINCH AJ 2 0.052

MCARDLE E 2 0.052

MCCARTHY D 2 0.052

MCCARTHY KJ 2 0.052

MCCULLOUGH PA 2 0.052

MCGARAUGHTY S 2 0.052

MCGOWAN TA 2 0.052

MCGRATH RT 2 0.052

MCMAHON R 2 0.052

MCMORROW T 2 0.052

MEHMOOD S 2 0.052

MEI XB 2 0.052

MEIJER E 2 0.052

MELAMED ML 2 0.052

MELDRUM KK 2 0.052

MELIN J 2 0.052

MENDOZA P 2 0.052

MENG LH 2 0.052

MENG XF 2 0.052

MENG XL 2 0.052

MENGEL M 2 0.052

MENINI S 2 0.052

MESSAOUDI S 2 0.052

MIAO CX 2 0.052

MIAO NJ 2 0.052

MIAO XY 2 0.052

MIGLIORINI A 2 0.052

MIKAME K 2 0.052

MIN HS 2 0.052

MINATOGUCHI S 2 0.052

MITHIEUX G 2 0.052

MITORI H 2 0.052

MITU G 2 0.052

MIYASATO Y 2 0.052

MIYATAKE A 2 0.052

MIYAZAKI-ANZAI S 2 0.052

MIYOSHI T 2 0.052

MIZUKAMI K 2 0.052

MIZUMOTO T 2 0.052

MIZUNO M 2 0.052

MOHAN S 2 0.052

MOLINA-JIJON E 2 0.052

MOON SH 2 0.052

MOORMAN AC 2 0.052

MORADPOUR D 2 0.052

MORGADO-PASCUAL JL 2 0.052

MORI T 2 0.052

MORII K 2 0.052

MORINAGA J 2 0.052

MORISHITA R 2 0.052

MORIYAMA T 2 0.052

MORONI G 2 0.052

MORTENSEN J 2 0.052

MOTA M 2 0.052

MOUGENOT B 2 0.052

MOUNT PF 2 0.052

MROWKA R 2 0.052

MUCHE M 2 0.052

MUDERS F 2 0.052

MUKAI Y 2 0.052

MULLEN W 2 0.052

MUNOZ-FELIX JM 2 0.052

MUREA M 2 0.052

MURRAY M 2 0.052

MUSCOGIURI G 2 0.052

MUTO S 2 0.052

MYAKALA K 2 0.052

NA KR 2 0.052

NAGAI K 2 0.052

NAGATA M 2 0.052

NAIR V 2 0.052

NAITO Y 2 0.052

NAJMABADI F 2 0.052

NAKAGAWA Y 2 0.052

NAKAI K 2 0.052

NAKAMURA J 2 0.052

NAKANISHI K 2 0.052

NAKASHIMA A 2 0.052

NAKATA T 2 0.052

NAKAYAMA Y 2 0.052

NAKAZAWA J 2 0.052

NAMGUNG S 2 0.052

NAMIKOSHI T 2 0.052

NAMORADO-TONIX C 2 0.052

NARA M 2 0.052

NARASIMHAN R 2 0.052

NASH EF 2 0.052

NAST CC 2 0.052

NASTASE MV 2 0.052

NASU T 2 0.052

NATH KA 2 0.052

NAVAR LG 2 0.052

NAYAK BK 2 0.052

NAZER R 2 0.052

NDISANG JF 2 0.052

NEALE TJ 2 0.052

NEAU E 2 0.052

NEMIROVSKI A 2 0.052

NEUMAYER HH 2 0.052

NEUSSER MA 2 0.052

NEUWIRT H 2 0.052

NEVEN E 2 0.052

NGUYEN V 2 0.052

NI J 2 0.052

NI WJ 2 0.052

NICHOLLS K 2 0.052

NICHOLSON A 2 0.052

NIE J 2 0.052

NIE L 2 0.052

NIE Q 2 0.052

NIE YN 2 0.052

NIELSEN PM 2 0.052

NIKKEL AL 2 0.052

NING JP 2 0.052

NING WB 2 0.052

NISHI S 2 0.052

NISHII N 2 0.052

NISHIMURA R 2 0.052

NIU AL 2 0.052

NIU JY 2 0.052

NOPPERT SJ 2 0.052

NORIKURA T 2 0.052

NORTIER JL 2 0.052

NOTO T 2 0.052

NOVE-JOSSERAND R 2 0.052

O'CONNELL PJ 2 0.052

O'CONNOR CM 2 0.052

OATES PJ 2 0.052

OBARA K 2 0.052

OBERDORF-MAASS SU 2 0.052

ODA T 2 0.052

OE Y 2 0.052

OGAWA H 2 0.052

OGAWA S 2 0.052

OH E 2 0.052

OH YS 2 0.052

OHASHI N 2 0.052

OHATA K 2 0.052

OHIGASHI M 2 0.052

OHMURA T 2 0.052

OHTAKE T 2 0.052

OKA M 2 0.052

OKADA T 2 0.052

OKAMOTO T 2 0.052

OKAMURA DM 2 0.052

OKANOUE T 2 0.052

OLIVO-MARIN JC 2 0.052

OLSEN S 2 0.052

OLSON L 2 0.052

ONISHI Y 2 0.052

OOSTERHUIS NR 2 0.052

ORABY MA 2 0.052

OREJUDO M 2 0.052

ORTIZ-MUNOZ G 2 0.052

OSICKA T 2 0.052

OSORIO-ALONSO H 2 0.052

OSTERBY R 2 0.052

OTA T 2 0.052

OUDIT GY 2 0.052

OUYANG X 2 0.052

OZBAY AB 2 0.052

OZGUL RK 2 0.052

PACKER CS 2 0.052

PAKFETRAT M 2 0.052

PALAU V 2 0.052

PAN XQ 2 0.052

PAN Y 2 0.052

PANCHAL SK 2 0.052

PANDEY KN 2 0.052

PANG RY 2 0.052

PARK EJ 2 0.052

PARK JS 2 0.052

PARK JY 2 0.052

PARK KM 2 0.052

PARK MJ 2 0.052

PARK SK 2 0.052

PARK SY 2 0.052

PARK TS 2 0.052

PARK YK 2 0.052

PASCUAL M 2 0.052

PASERO G 2 0.052

PASTOR-SOLER NM 2 0.052

PATEL V 2 0.052

PATERNI M 2 0.052

PATTERSON CC 2 0.052

PAUL E 2 0.052

PAVENSTADT H 2 0.052

PAYNE WD 2 0.052

PECOITS R 2 0.052

PEDERSEN TX 2 0.052

PEI Y 2 0.052

PELLEGRINO S 2 0.052

PENA MJ 2 0.052

PENESCU M 2 0.052

PENFOLD SA 2 0.052

PENG A 2 0.052

PENG H 2 0.052

PENG JC 2 0.052

PENG LL 2 0.052

PENG RY 2 0.052

PENG YX 2 0.052

PENG ZQ 2 0.052

PERBAL B 2 0.052

PEREZ G 2 0.052

PEREZ-BARRIOCANAL F 2 0.052

PESSINA AC 2 0.052

PESSOA EA 2 0.052

PETE J 2 0.052

PETERS H 2 0.052

PETERS HP 2 0.052

PETERSON DR 2 0.052

PETERSON EL 2 0.052

PETERSON MC 2 0.052

PEZZOLESI MG 2 0.052

PHILLIPS GO 2 0.052

PIAO S 2 0.052

PIAO SG 2 0.052

PIAO YL 2 0.052

PIAZZA M 2 0.052

PICHETTE V 2 0.052

PIECHA G 2 0.052

PINI A 2 0.052

PINTO S 2 0.052

PISCITELLI F 2 0.052

PITT B 2 0.052

PLAISIER E 2 0.052

POCZATEK MH 2 0.052

PODESTA L 2 0.052

POLHILL TS 2 0.052

POLLOCK JS 2 0.052

POMPOSELLI F 2 0.052

POPESCU A 2 0.052

PORTA M 2 0.052

POUDYAL H 2 0.052

POWELL D 2 0.052

PRAKOSO D 2 0.052

PRESSLER T 2 0.052

PRESTES TRR 2 0.052

PROCTOR G 2 0.052

PROFY AT 2 0.052

PUELLES VG 2 0.052

PUGLIESE F 2 0.052

PULLMAN J 2 0.052

QI J 2 0.052

QI JP 2 0.052

QI Y 2 0.052

QI YF 2 0.052

QIAN DW 2 0.052

QIAN Q 2 0.052

QIAN YY 2 0.052

QIAO ZD 2 0.052

QIN LH 2 0.052

QIN XG 2 0.052

QIU LR 2 0.052

QIU MC 2 0.052

QUAGGIN SE 2 0.052

QUARLES CC 2 0.052

QUINN MT 2 0.052

QUIROGA B 2 0.052

QUON BS 2 0.052

RADHAKRISHNAN J 2 0.052

RADU D 2 0.052

RAHMAN A 2 0.052

RAHNENFUHRER J 2 0.052

RAJ D 2 0.052

RAJAGOPALAN S 2 0.052

RAJARAM RD 2 0.052

RAJAS F 2 0.052

RAMACHANDRARAO SP 2 0.052

RAMBOW JA 2 0.052

RAMIREZ V 2 0.052

RANJIT S 2 0.052

RAPHAEL KL 2 0.052

RASCALOU S 2 0.052

RASHID A 2 0.052

READ NC 2 0.052

REGNER KR 2 0.052

REICH HN 2 0.052

REISER J 2 0.052

REN DJ 2 0.052

REN ST 2 0.052

REN SY 2 0.052

REN YZ 2 0.052

RENKE M 2 0.052

RESCH M 2 0.052

REUDELHUBER TL 2 0.052

REYES JL 2 0.052

RHALEB NE 2 0.052

RIAHI Y 2 0.052

RIEGGER GA 2 0.052

RIENSTRA H 2 0.052

RIPPE B 2 0.052

RISER ML 2 0.052

RISER SC 2 0.052

RISLER T 2 0.052

RITCHIE RH 2 0.052

ROBERTSON SJ 2 0.052

ROBSON SC 2 0.052

RODICIO JL 2 0.052

RODRIGUEZ-MUNOZ R 2 0.052

RODRIGUEZ-PUYOL D 2 0.052

RODRIGUEZ-PUYOL M 2 0.052

ROELOFS JJTH 2 0.052

ROMAGNANI P 2 0.052

RONCAL CA 2 0.052

ROOZBEH J 2 0.052

ROSENBERG A 2 0.052

ROSSERT J 2 0.052

ROSSIGNOL P 2 0.052

ROUSSEL R 2 0.052

ROUTH RE 2 0.052

RUPP LB 2 0.052

RUTKOWSKI B 2 0.052

RUTKOWSKI P 2 0.052

RUTTER WC 2 0.052

RYAN MP 2 0.052

RYU DR 2 0.052

RYU S 2 0.052

SADLIER DM 2 0.052

SAFAR MM 2 0.052

SAGE EH 2 0.052

SAIGUSA T 2 0.052

SAITO D 2 0.052

SAKAGUCHI M 2 0.052

SAKATA N 2 0.052

SALAH EM 2 0.052

SALEEM MA 2 0.052

SALEH MA 2 0.052

SALLUSTIO F 2 0.052

SALVATORE SP 2 0.052

SAMUEL CS 2 0.052

SANDRES-SAUNE K 2 0.052

SANKRITYAYAN H 2 0.052

SANO M 2 0.052

SANO T 2 0.052

SANTAMARIA H 2 0.052

SARI FR 2 0.052

SARIOGLU S 2 0.052

SATAKE E 2 0.052

SATARANATARAJAN K 2 0.052

SATHYAPALAN T 2 0.052

SATIRAPOJ B 2 0.052

SATO-HORIGUCHI C 2 0.052

SATTAR MZA 2 0.052

SAUNDERS HJ 2 0.052

SAWAI A 2 0.052

SCARPELLINI A 2 0.052

SCHAEFER F 2 0.052

SCHALKWIJK CG 2 0.052

SCHELLING JR 2 0.052

SCHIFFER M 2 0.052

SCHILLER A 2 0.052

SCHLINGEMANN RO 2 0.052

SCHMADERER C 2 0.052

SCHMID H 2 0.052

SCHMIDT MA 2 0.052

SCHMIEDER RE 2 0.052

SCHONBAUER R 2 0.052

SCHOR N 2 0.052

SCHRIER RW 2 0.052

SCHROEDER N 2 0.052

SCHROTEN NF 2 0.052

SECCHI MF 2 0.052

SEIDLER R 2 0.052

SEINO Y 2 0.052

SEKIMOTO A 2 0.052

SELGAS R 2 0.052

SELVIN E 2 0.052

SEN K 2 0.052

SEN S 2 0.052

SEO JB 2 0.052

SEONG EY 2 0.052

SERAFINCEANU C 2 0.052

SERNIA C 2 0.052

SHAN XO 2 0.052

SHANER BE 2 0.052

SHANG HX 2 0.052

SHANKLAND SJ 2 0.052

SHAO H 2 0.052

SHAO J 2 0.052

SHAO XH 2 0.052

SHARKOVSKA Y 2 0.052

SHAW MA 2 0.052

SHEN FX 2 0.052

SHEN HH 2 0.052

SHEN M 2 0.052

SHEN YC 2 0.052

SHEN YT 2 0.052

SHENG J 2 0.052

SHENG SF 2 0.052

SHENG X 2 0.052

SHEU ML 2 0.052

SHI BL 2 0.052

SHI JB 2 0.052

SHI JX 2 0.052

SHI LL 2 0.052

SHI Q 2 0.052

SHI YF 2 0.052

SHIBATA R 2 0.052

SHIBATA S 2 0.052

SHIELD CF 2 0.052

SHIMIZU Y 2 0.052

SHIMOKATA K 2 0.052

SHIMOKAWA H 2 0.052

SHIMOTOMAI T 2 0.052

SHINOHARA M 2 0.052

SHU FL 2 0.052

SHU SS 2 0.052

SHUI H 2 0.052

SIAMANTOURAS E 2 0.052

SIERRA-MONDRAGON E 2 0.052

SILVA AEB 2 0.052

SIMA A 2 0.052

SIMONE S 2 0.052

SINGH G 2 0.052

SINGH P 2 0.052

SINGH S 2 0.052

SIRLI R 2 0.052

SISKIND LJ 2 0.052

SIWY J 2 0.052

SKIBBA M 2 0.052

SKUPIEN J 2 0.052

SMITH A 2 0.052

SMITH KD 2 0.052

SMITH MC 2 0.052

SNELSON M 2 0.052

SOBCZAK M 2 0.052

SOEJIMA Y 2 0.052

SOETIKNO V 2 0.052

SOHN E 2 0.052

SOLIS N 2 0.052

SON JY 2 0.052

SONG GF 2 0.052

SONG JB 2 0.052

SONG S 2 0.052

SONG YR 2 0.052

SONG YY 2 0.052

SORICE GP 2 0.052

SOUMA T 2 0.052

SPATARO BC 2 0.052

SPEECKAERT MM 2 0.052

SPEECKAERT R 2 0.052

SPIRES D 2 0.052

SPRADLING PR 2 0.052

STAESSEN JA 2 0.052

STAHL RAK 2 0.052

STANCU S 2 0.052

STANGENBERG S 2 0.052

STEEDMAN T 2 0.052

STEFAN G 2 0.052

STEFANSKI A 2 0.052

STEFFES MW 2 0.052

STEGALL MD 2 0.052

STELLA I 2 0.052

STILLMAN IE 2 0.052

STORINO FAA 2 0.052

STRIKER GE 2 0.052

SU SL 2 0.052

SU W 2 0.052

SU XX 2 0.052

SUAREZ R 2 0.052

SUE YM 2 0.052

SUGANAMI T 2 0.052

SUI MS 2 0.052

SUKOWSKI E 2 0.052

SUMIDA T 2 0.052

SUMIDA Y 2 0.052

SUMMERS SA 2 0.052

SUN BK 2 0.052

SUN C 2 0.052

SUN CB 2 0.052

SUN F 2 0.052

SUN GD 2 0.052

SUN H 2 0.052

SUN IO 2 0.052

SUN JP 2 0.052

SUN RB 2 0.052

SUN X 2 0.052

SUN XM 2 0.052

SUN YL 2 0.052

SUN YM 2 0.052

SUN YY 2 0.052

SUN ZJ 2 0.052

SUNDARARAJAN S 2 0.052

SUNG HY 2 0.052

SURENDRAN K 2 0.052

SUTARIYA B 2 0.052

SYKES K 2 0.052

SYME HM 2 0.052

SZEDERKENYI E 2 0.052

SZENASI G 2 0.052

SZENOHRADSZKY P 2 0.052

SZETO SG 2 0.052

TACK I 2 0.052

TAHA D 2 0.052

TAHARA A 2 0.052

TAKAGI S 2 0.052

TAKAHASHI A 2 0.052

TAKAHASHI M 2 0.052

TAKAHASHI S 2 0.052

TAKAHASHI-FUJIGASAKI J 2 0.052

TAKASHI Y 2 0.052

TAKEBAYASHI S 2 0.052

TAKEDA M 2 0.052

TAKEI K 2 0.052

TAKENAKA T 2 0.052

TAKIYAMA Y 2 0.052

TAM FWK 2 0.052

TAMURA M 2 0.052

TAN ALY 2 0.052

TAN CYR 2 0.052

TAN KB 2 0.052

TAN KCB 2 0.052

TAN RY 2 0.052

TANABE J 2 0.052

TANAKA J 2 0.052

TANAKA M 2 0.052

TANAKA Y 2 0.052

TANEDA S 2 0.052

TANG AN 2 0.052

TANG CS 2 0.052

TANG JQ 2 0.052

TANG K 2 0.052

TANG LQ 2 0.052

TANG PCT 2 0.052

TANG S 2 0.052

TANG SM 2 0.052

TANG XL 2 0.052

TANG Y 2 0.052

TANIGUCHI K 2 0.052

TANIGUCHI S 2 0.052

TANIYAMA Y 2 0.052

TAO BB 2 0.052

TAO L 2 0.052

TASKESEN M 2 0.052

TATE M 2 0.052

TATSUKAWA S 2 0.052

TCHKONIA T 2 0.052

TELES F 2 0.052

TEN DIJKE P 2 0.052

TENCER J 2 0.052

TEPEL M 2 0.052

TESCH G 2 0.052

TESHALE EH 2 0.052

TESHIGAWARA S 2 0.052

TETTA C 2 0.052

THALLAS V 2 0.052

THARAUX PL 2 0.052

THIBODEAU JF 2 0.052

THOMSON SC 2 0.052

TIAN M 2 0.052

TIAN PX 2 0.052

TIAN Y 2 0.052

TIAN YY 2 0.052

TIWARI S 2 0.052

TOBIN JV 2 0.052

TOFFOLI B 2 0.052

TOFOVIC SP 2 0.052

TOLKOFF-RUBIN N 2 0.052

TOMASCHITZ A 2 0.052

TONG C 2 0.052

TONG Y 2 0.052

TOPLEY N 2 0.052

TORRA R 2 0.052

TOUPANCE O 2 0.052

TOYODA M 2 0.052

TREVISAN R 2 0.052

TROOST JP 2 0.052

TSAI HC 2 0.052

TSAI TJ 2 0.052

TSUCHIYA K 2 0.052

TSUNENARI I 2 0.052

TUGNAIT M 2 0.052

TURYN D 2 0.052

TWIGG SM 2 0.052

TYAGI SC 2 0.052

TYLICKI L 2 0.052

UCHIDA K 2 0.052

UCHIMURA K 2 0.052

UDI S 2 0.052

UIL M 2 0.052

UJIKE H 2 0.052

ULUCAN C 2 0.052

URQUHART R 2 0.052

UTTARWAR L 2 0.052

VALDERRAMA E 2 0.052

VALINO-RIVAS L 2 0.052

VALLEE JP 2 0.052

VAN DEN BERG EH 2 0.052

VAN DEN HEUVEL MC 2 0.052

VAN DER GEEST RN 2 0.052

VAN DER GIEZEN DM 2 0.052

VAN DER HARST P 2 0.052

VAN DER VELDE AR 2 0.052

VAN DER VELDEN J 2 0.052

VAN KOPPEN A 2 0.052

VAN KUPPEVELT TH 2 0.052

VANDERMEERSCH S 2 0.052

VANDESOMPELE J 2 0.052

VANES LA 2 0.052

VANHOLDER R 2 0.052

VANNAY A 2 0.052

VARATHARAJAN R 2 0.052

VARGA ZV 2 0.052

VARKONYI T 2 0.052

VASANTH S 2 0.052

VENKATESAN B 2 0.052

VERDERIO EAM 2 0.052

VERGARA A 2 0.052

VERHULST A 2 0.052

VERMA A 2 0.052

VERONESE FV 2 0.052

VIANNA R 2 0.052

VIDAL A 2 0.052

VIEIRA JM 2 0.052

VILLALBA JM 2 0.052

VLAD M 2 0.052

VLAHOU A 2 0.052

VLASSARA H 2 0.052

VLEMING LJ 2 0.052

VUKOJEVIC K 2 0.052

WAIKAR SS 2 0.052

WAKEFIELD L 2 0.052

WALLWORK J 2 0.052

WALSH DW 2 0.052

WALSH J 2 0.052

WALSH K 2 0.052

WALTHER T 2 0.052

WAN J 2 0.052

WAN ZY 2 0.052

WANG CC 2 0.052

WANG CS 2 0.052

WANG FC 2 0.052

WANG FL 2 0.052

WANG GJ 2 0.052

WANG HP 2 0.052

WANG HS 2 0.052

WANG JD 2 0.052

WANG JF 2 0.052

WANG LC 2 0.052

WANG LF 2 0.052

WANG LN 2 0.052

WANG LP 2 0.052

WANG LW 2 0.052

WANG MH 2 0.052

WANG PH 2 0.052

WANG QH 2 0.052

WANG SC 2 0.052

WANG SF 2 0.052

WANG WN 2 0.052

WANG XT 2 0.052

WANG ZC 2 0.052

WANG ZD 2 0.052

WANG ZG 2 0.052

WANG ZM 2 0.052

WANIBUCHI H 2 0.052

WARD MS 2 0.052

WATANABE A 2 0.052

WATANABE Y 2 0.052

WATATANI H 2 0.052

WATSON A 2 0.052

WATSON AMD 2 0.052

WEBER M 2 0.052

WEHRMANN M 2 0.052

WEI BA 2 0.052

WEI CG 2 0.052

WEI JB 2 0.052

WEI M 2 0.052

WEI QQ 2 0.052

WEI TT 2 0.052

WEI YZ 2 0.052

WEIR MR 2 0.052

WEITZ S 2 0.052

WELCH WJ 2 0.052

WEN JG 2 0.052

WEN P 2 0.052

WEN X 2 0.052

WEN YM 2 0.052

WENG HB 2 0.052

WENG WC 2 0.052

WENG WY 2 0.052

WENZEL U 2 0.052

WESTENBRINK BD 2 0.052

WESTENDORP RGJ 2 0.052

WETZELS JF 2 0.052

WHEELER-JONES CPD 2 0.052

WHITEHOUSE JL 2 0.052

WIECH F 2 0.052

WILCOX CS 2 0.052

WILLIAM-OLSSON L 2 0.052

WILLIAMS ME 2 0.052

WILLIAMS V 2 0.052

WILMER WA 2 0.052

WINBANKS CE 2 0.052

WOLTERBEEK R 2 0.052

WONG MYW 2 0.052

WOOKEY PJ 2 0.052

WOOLLARD JR 2 0.052

WU CY 2 0.052

WU GL 2 0.052

WU GY 2 0.052

WU JD 2 0.052

WU JH 2 0.052

WU JN 2 0.052

WU JS 2 0.052

WU KD 2 0.052

WU LN 2 0.052

WU P 2 0.052

WU PL 2 0.052

WU PW 2 0.052

WU QL 2 0.052

WU TF 2 0.052

WU TH 2 0.052

WU WF 2 0.052

WU WY 2 0.052

WU XC 2 0.052

WU XQ 2 0.052

WU YF 2 0.052

WYGRECKA M 2 0.052

XI WW 2 0.052

XI Y 2 0.052

XIA AZ 2 0.052

XIANG L 2 0.052

XIANG XH 2 0.052

XIANG Y 2 0.052

XIAO FX 2 0.052

XIAO XC 2 0.052

XIAO YH 2 0.052

XIAO YQ 2 0.052

XIAO YW 2 0.052

XIE D 2 0.052

XIE RJ 2 0.052

XIE SH 2 0.052

XIE W 2 0.052

XIN P 2 0.052

XIN W 2 0.052

XING CY 2 0.052

XING J 2 0.052

XIONG HB 2 0.052

XIONG J 2 0.052

XIONG JC 2 0.052

XIONG XF 2 0.052

XU B 2 0.052

XU C 2 0.052

XU GS 2 0.052

XU JL 2 0.052

XU JW 2 0.052

XU JX 2 0.052

XU LJ 2 0.052

XU LQ 2 0.052

XU LX 2 0.052

XU Q 2 0.052

XU QH 2 0.052

XU SJ 2 0.052

XU WL 2 0.052

XU XX 2 0.052

XU ZJ 2 0.052

XU ZP 2 0.052

XUE H 2 0.052

XUE HY 2 0.052

XUE WJ 2 0.052

YADAV D 2 0.052

YAGI K 2 0.052

YAMADA H 2 0.052

YAMAGUCHI S 2 0.052

YAMAHARA J 2 0.052

YAMAMOTO K 2 0.052

YAMAMOTO S 2 0.052

YAMAMOTO T 2 0.052

YAMASAKI H 2 0.052

YAMASHITA M 2 0.052

YAMASHITA T 2 0.052

YAMATE J 2 0.052

YAMAZAKI M 2 0.052

YAN B 2 0.052

YAN S 2 0.052

YAN XQ 2 0.052

YAN XX 2 0.052

YAN YL 2 0.052

YANEZ A 2 0.052

YANG B 2 0.052

YANG D 2 0.052

YANG DH 2 0.052

YANG JY 2 0.052

YANG LC 2 0.052

YANG MJ 2 0.052

YANG N 2 0.052

YANG P 2 0.052

YANG SF 2 0.052

YANG SY 2 0.052

YANG TT 2 0.052

YANG W 2 0.052

YANG XD 2 0.052

YANG XF 2 0.052

YANG XY 2 0.052

YANG YP 2 0.052

YANG YS 2 0.052

YAO J 2 0.052

YAO WJ 2 0.052

YAO XM 2 0.052

YAO XQ 2 0.052

YAP FYT 2 0.052

YARD B 2 0.052

YASUI K 2 0.052

YE MY 2 0.052

YEGER H 2 0.052

YEH HI 2 0.052

YENER S 2 0.052

YESIL S 2 0.052

YI F 2 0.052

YI WY 2 0.052

YI YE 2 0.052

YILMAZ B 2 0.052

YIN DH 2 0.052

YIN HM 2 0.052

YIN HY 2 0.052

YIN J 2 0.052

YIN JJ 2 0.052

YIN PH 2 0.052

YOKORO M 2 0.052

YOKOYAMA H 2 0.052

YOKOZAWA T 2 0.052

YONEDA M 2 0.052

YOON HE 2 0.052

YOON KH 2 0.052

YOSHIDA Y 2 0.052

YOSHIMURA M 2 0.052

YOSHIOKA N 2 0.052

YOSHIOKA T 2 0.052

YOU H 2 0.052

YU FX 2 0.052

YU H 2 0.052

YU JL 2 0.052

YU JW 2 0.052

YU JY 2 0.052

YU L 2 0.052

YU WC 2 0.052

YU X 2 0.052

YU YL 2 0.052

YUAN CJ 2 0.052

YUAN F 2 0.052

YUAN FY 2 0.052

YUAN K 2 0.052

YUAN L 2 0.052

YUAN LP 2 0.052

YUAN XN 2 0.052

YUAN YG 2 0.052

YUEN DYC 2 0.052

YURISTA SR 2 0.052

ZAITONE SA 2 0.052

ZAKY AA 2 0.052

ZANDBERGEN M 2 0.052

ZELDIN DC 2 0.052

ZENG LF 2 0.052

ZENG O 2 0.052

ZENG XJ 2 0.052

ZENG Y 2 0.052

ZENG YM 2 0.052

ZENNARO C 2 0.052

ZERUTH G 2 0.052

ZHA DQ 2 0.052

ZHA Y 2 0.052

ZHAN HK 2 0.052

ZHAN HY 2 0.052

ZHAN J 2 0.052

ZHAN M 2 0.052

ZHAN YT 2 0.052

ZHANG CM 2 0.052

ZHANG CZ 2 0.052

ZHANG EL 2 0.052

ZHANG HF 2 0.052

ZHANG HQ 2 0.052

ZHANG HX 2 0.052

ZHANG JS 2 0.052

ZHANG JW 2 0.052

ZHANG JY 2 0.052

ZHANG LW 2 0.052

ZHANG MC 2 0.052

ZHANG MF 2 0.052

ZHANG MM 2 0.052

ZHANG QB 2 0.052

ZHANG QD 2 0.052

ZHANG QX 2 0.052

ZHANG RY 2 0.052

ZHANG SJ 2 0.052

ZHANG SQ 2 0.052

ZHANG TY 2 0.052

ZHANG WB 2 0.052

ZHANG WG 2 0.052

ZHANG WN 2 0.052

ZHANG WT 2 0.052

ZHANG XW 2 0.052

ZHANG YM 2 0.052

ZHANG YS 2 0.052

ZHAO CY 2 0.052

ZHAO F 2 0.052

ZHAO G 2 0.052

ZHAO HR 2 0.052

ZHAO JX 2 0.052

ZHAO P 2 0.052

ZHAO Q 2 0.052

ZHAO SQ 2 0.052

ZHAO XL 2 0.052

ZHAO XM 2 0.052

ZHAO XS 2 0.052

ZHAO YJ 2 0.052

ZHEN JH 2 0.052

ZHENG C 2 0.052

ZHENG FP 2 0.052

ZHENG H 2 0.052

ZHENG J 2 0.052

ZHENG W 2 0.052

ZHONG M 2 0.052

ZHONG S 2 0.052

ZHONG Y 2 0.052

ZHONG YF 2 0.052

ZHONG YN 2 0.052

ZHOU B 2 0.052

ZHOU F 2 0.052

ZHOU LP 2 0.052

ZHOU LT 2 0.052

ZHOU P 2 0.052

ZHOU S 2 0.052

ZHOU SN 2 0.052

ZHOU T 2 0.052

ZHOU XH 2 0.052

ZHOU YF 2 0.052

ZHOU YX 2 0.052

ZHOU ZH 2 0.052

ZHU CZ 2 0.052

ZHU FX 2 0.052

ZHU HB 2 0.052

ZHU HP 2 0.052

ZHU HY 2 0.052

ZHU L 2 0.052

ZHU N 2 0.052

ZHU XW 2 0.052

ZHU YB 2 0.052

ZHU YH 2 0.052

ZHU ZM 2 0.052

ZHUANG AW 2 0.052

ZHUANG F 2 0.052

ZHUO H 2 0.052

ZIEMANN M 2 0.052

ZINGERMAN B 2 0.052

ZINKER B 2 0.052

ZOU CB 2 0.052

ZOU SZ 2 0.052

ZOU X 2 0.052

ZOU YT 2 0.052

ZURBIG P 2 0.052

AAGAARD A 1 0.026

AARABI M 1 0.026

ABBAS N 1 0.026

ABBAS NAT 1 0.026

ABBAS TM 1 0.026

ABBASI A 1 0.026

ABBASIAN N 1 0.026

ABBATE A 1 0.026

ABBATE M 1 0.026

ABBOUDT HE 1 0.026

ABD EL MOTTELEB DM 1 0.026

ABD EL-AZIZ TA 1 0.026

ABDEL-AZIZ HR 1 0.026

ABDEL-HAKEEM EA 1 0.026

ABDELATY AMSEK 1 0.026

ABDELRAHMAN AM 1 0.026

ABDI R 1 0.026

ABDOLLAHI M 1 0.026

ABDOU NEA 1 0.026

ABDULLAH N 1 0.026

ABE N 1 0.026

ABEDI-AZAR S 1 0.026

ABEL ED 1 0.026

ABHAYARATNA K 1 0.026

ABHAYARATNA W 1 0.026

ABO-ZENAH H 1 0.026

ABOU-AYACHE R 1 0.026

ABOU-HANY HO 1 0.026

ABOU-KHEIR W 1 0.026

ABOUEZZEDDINE OF 1 0.026

ABRAHAM AA 1 0.026

ABRAHAM DJ 1 0.026

ABRAHAM E 1 0.026

ABRAHAM VC 1 0.026

ABRAHAMS AC 1 0.026

ABRAMOVICH C 1 0.026

ABRAMOWITZ MK 1 0.026

ABREU C 1 0.026

ABROON S 1 0.026

ABU JAWDEH BG 1 0.026

ABU-ELSAAD NM 1 0.026

ABUDUREYIMU M 1 0.026

ACKERMANN M 1 0.026

ACOU WJ 1 0.026

ACUNA-HERNANDEZ MA 1 0.026

ADACHI K 1 0.026

ADAMS LA 1 0.026

ADDEPALLI V 1 0.026

ADDY C 1 0.026

ADEGHATE E 1 0.026

ADESINA S 1 0.026

ADEY DB 1 0.026

ADHIKARI K 1 0.026

ADHIKARY L 1 0.026

ADIJIANG A 1 0.026

ADLER GK 1 0.026

ADOURIAN A 1 0.026

AERTS R 1 0.026

AFIONE S 1 0.026

AFONSO J 1 0.026

AFRIN R 1 0.026

AFSAR B 1 0.026

AFSHARIANI R 1 0.026

AFZAL A 1 0.026

AGAPOVA O 1 0.026

AGELLON LB 1 0.026

AGGARWAL D 1 0.026

AGOUNI A 1 0.026

AGRICOLA T 1 0.026

AGUAYO-CANELA M 1 0.026

AGUDO-CONDE C 1 0.026

AGUERO R 1 0.026

AGUIRRE L 1 0.026

AGUIRRE R 1 0.026

AHADZADEH E 1 0.026

AHLQVIST E 1 0.026

AHLUWALIA TS 1 0.026

AHMAD B 1 0.026

AHMAD M 1 0.026

AHMAD SNS 1 0.026

AHMED F 1 0.026

AHMED FZ 1 0.026

AHMED IA 1 0.026

AHMED L 1 0.026

AHMED MU 1 0.026

AHMED SB 1 0.026

AHN C 1 0.026

AHN ER 1 0.026

AHN HJ 1 0.026

AHN JH 1 0.026

AHN KO 1 0.026

AHN KS 1 0.026

AHN SY 1 0.026

AHN YB 1 0.026

AHN YM 1 0.026

AHOLA AJ 1 0.026

AHONEN L 1 0.026

AHUJA S 1 0.026

AI N 1 0.026

AI XP 1 0.026

AIBARA Y 1 0.026

AIELLO V 1 0.026

AIRLEY R 1 0.026

AIZAWA K 1 0.026

AJAYI EIO 1 0.026

AKAMATA K 1 0.026

AKAMINE T 1 0.026

AKAN P 1 0.026

AKAO S 1 0.026

AKBARI S 1 0.026

AKBARIAN F 1 0.026

AKBARIAN N 1 0.026

AKCA O 1 0.026

AKCAY A 1 0.026

AKESON AL 1 0.026

AKHTAR SS 1 0.026

AKIMOTO Y 1 0.026

AKIYAMA Y 1 0.026

AKKERMAN N 1 0.026

AKLA N 1 0.026

AKOLKAR B 1 0.026

AKSAN S 1 0.026

AKSU SI 1 0.026

AKYILDIZ ZI 1 0.026

AKYUREK LM 1 0.026

AL BESHER S 1 0.026

AL MAMUN A 1 0.026

AL ZA'ABI M 1 0.026

AL-ADLANY MAA 1 0.026

AL-AJMI HN 1 0.026

AL-AMIN MA 1 0.026

AL-GAYYAR MMH 1 0.026

AL-HUSSAINI H 1 0.026

AL-MALKI AL 1 0.026

AL-OBAIDI N 1 0.026

AL-RASHEED N 1 0.026

AL-RASHEED NM 1 0.026

AL-ROWAIS H 1 0.026

AL-SAIEGH Y 1 0.026

AL-SHARE QY 1 0.026

AL-TRAD B 1 0.026

AL-WAILI H 1 0.026

AL-WAILI N 1 0.026

AL-WAILI T 1 0.026

ALAJBEGOVIC A 1 0.026

ALAJMI MF 1 0.026

ALAM ML 1 0.026

ALAM N 1 0.026

ALARAJ M 1 0.026

ALARCON P 1 0.026

ALASHKAR F 1 0.026

ALBAI A 1 0.026

ALBAYRAK O 1 0.026

ALBERT C 1 0.026

ALBERTAZZI A 1 0.026

ALBERTONI GA 1 0.026

ALBINO-TEIXEIRA A 1 0.026

ALBINSSON S 1 0.026

ALCANTARA D 1 0.026

ALDAMIZ-ECHEVARRIA L 1 0.026

ALEKSANDROWICZ E 1 0.026

ALEKSANDROWICZ-WRONA E 1 0.026

ALENINA N 1 0.026

ALESUTAN I 1 0.026

ALEXANDER IE 1 0.026

ALEXANDER JJ 1 0.026

ALFAIFI MY 1 0.026

ALGE JL 1 0.026

ALHADIDY AM 1 0.026

ALHASHIM A 1 0.026

ALI AA 1 0.026

ALI I 1 0.026

ALI T 1 0.026

ALICANDRO G 1 0.026

ALIEV G 1 0.026

ALIMARDANI-BESSETTE M 1 0.026

ALKANDARI A 1 0.026

ALKHALAF A 1 0.026

ALKHANSA S 1 0.026

ALKHOULI M 1 0.026

ALKHUDHAYRI A 1 0.026

ALLAIRE NE 1 0.026

ALLARD J 1 0.026

ALLEN NB 1 0.026

ALLEN P 1 0.026

ALLEN RDM 1 0.026

ALLER R 1 0.026

ALLERT S 1 0.026

ALLINOVI M 1 0.026

ALLIONE A 1 0.026

ALM P 1 0.026

ALMAGUER M 1 0.026

ALMAS K 1 0.026

ALMEIDA LF 1 0.026

ALOBEID B 1 0.026

ALONSO HO 1 0.026

ALPINI G 1 0.026

ALRIC L 1 0.026

ALROB OA 1 0.026

ALSAAD KO 1 0.026

ALSADY M 1 0.026

ALSAYARI A 1 0.026

ALSUABEYL MS 1 0.026

ALTAHER A 1 0.026

ALTAY C 1 0.026

ALTER M 1 0.026

ALTINTEPE L 1 0.026

ALTUHAIFI T 1 0.026

ALTUNOREN O 1 0.026

ALVAREZ-AGUILAR C 1 0.026

ALVAREZ-LLAMAS G 1 0.026

ALVES SA 1 0.026

ALZOUBI KH 1 0.026

AMADOR CA 1 0.026

AMANI A 1 0.026

AMANN B 1 0.026

AMANN KU 1 0.026

AMANO S 1 0.026

AMANZADEH M 1 0.026

AMARA FM 1 0.026

AMARAL LSD 1 0.026

AMARO M 1 0.026

AMASHEH S 1 0.026

AMAT R 1 0.026

AMAYA JC 1 0.026

AMBATI CR 1 0.026

AMBINATHAN JPN 1 0.026

AMBROISINE ML 1 0.026

AMBROSIO GB 1 0.026

AMBROSIUS WT 1 0.026

AMBU S 1 0.026

AMER A 1 0.026

AMER AM 1 0.026

AMER H 1 0.026

AMERICA YGCJ 1 0.026

AMIR S 1 0.026

AMMINI AC 1 0.026

AMOEDO ML 1 0.026

AMOS LA 1 0.026

AMPUERO J 1 0.026

AMREIN C 1 0.026

AMRELIA PM 1 0.026

AN CM 1 0.026

AN EJ 1 0.026

AN HY 1 0.026

AN WS 1 0.026

AN ZP 1 0.026

ANAGNOSTIS P 1 0.026

ANANTHAPADMANABHAN K 1 0.026

ANAYAMA H 1 0.026

ANDEEN NK 1 0.026

ANDERBERG RJ 1 0.026

ANDERLUH M 1 0.026

ANDERSEN H 1 0.026

ANDERSEN HU 1 0.026

ANDERSEN S 1 0.026

ANDERSON G 1 0.026

ANDERSON S 1 0.026

ANDERSSON AK 1 0.026

ANDIAPPAN R 1 0.026

ANDO A 1 0.026

ANDRADE CF 1 0.026

ANDRADE F 1 0.026

ANDRADE H 1 0.026

ANDRADE L 1 0.026

ANDRADE RJ 1 0.026

ANDRES-HERNANDO A 1 0.026

ANDREWS J 1 0.026

ANDRIANESIS V 1 0.026

ANDRIKOPOULOS S 1 0.026

ANGELETTI A 1 0.026

ANGELKORT B 1 0.026

ANGELOTTI ML 1 0.026

ANGLICHEAU D 1 0.026

ANGUIANO L 1 0.026

ANGUS PW 1 0.026

ANILKUMAR N 1 0.026

ANORGA S 1 0.026

ANRAKU M 1 0.026

ANSARI A 1 0.026

ANSTEY NM 1 0.026

ANTIGNAC C 1 0.026

ANTINOZZI PA 1 0.026

ANTOHE F 1 0.026

ANTOINE C 1 0.026

ANTOINE DJ 1 0.026

ANTONENKO V 1 0.026

ANTONI D 1 0.026

ANTONIADI G 1 0.026

ANTONIOLI E 1 0.026

ANTUNES AMM 1 0.026

ANTUNES PE 1 0.026

ANTUNES TT 1 0.026

ANURADHA CV 1 0.026

ANVERSA P 1 0.026

ANZAI N 1 0.026

AO C 1 0.026

AO X 1 0.026

AO Y 1 0.026

AO ZH 1 0.026

AODAOFU A 1 0.026

AOKI J 1 0.026

AOKI T 1 0.026

AOYAGI D 1 0.026

AOYAGI R 1 0.026

AOYAMA M 1 0.026

AOYAMA T 1 0.026

AOZASA N 1 0.026

APITZSCH JC 1 0.026

APPEL GB 1 0.026

AQIE KR 1 0.026

ARA JM 1 0.026

ARAI K 1 0.026

ARAKAWA M 1 0.026

ARAKAWA S 1 0.026

ARAKAWA T 1 0.026

ARAKELYAN A 1 0.026

ARAKI A 1 0.026

ARAMBURU-BODAS O 1 0.026

ARAN JM 1 0.026

ARANGALAGE D 1 0.026

ARANGO-RODRIGUEZ ML 1 0.026

ARAOKA T 1 0.026

ARATA S 1 0.026

ARATA Y 1 0.026

ARAUJO ES 1 0.026

ARAUJO SRR 1 0.026

ARAUZ-PACHECO C 1 0.026

ARBEENY C 1 0.026

ARBOGAST H 1 0.026

ARBONES-MAINAR JM 1 0.026

ARCE L 1 0.026

ARCHILA LR 1 0.026

ARCOS-SACRAMENTO VG 1 0.026

ARDALAN M 1 0.026

ARDENKJAER-LARSEN JH 1 0.026

ARDHANARI S 1 0.026

ARDURA JA 1 0.026

AREND L 1 0.026

ARESTI V 1 0.026

ARESU L 1 0.026

AREVALO M 1 0.026

AREVALO MA 1 0.026

ARGAIZ ER 1 0.026

ARGANI H 1 0.026

ARGENZIANO M 1 0.026

ARGOUD K 1 0.026

ARGUELLO-GARCIA R 1 0.026

ARGYROPOULOS C 1 0.026

ARGYROPOULOS CP 1 0.026

ARI E 1 0.026

ARIAS M 1 0.026

ARIAS SCA 1 0.026

ARIAS-JIMENEZ JL 1 0.026

ARIGA T 1 0.026

ARIHIRO K 1 0.026

ARIKAWA E 1 0.026

ARIMURA A 1 0.026

ARITA S 1 0.026

ARITAKE K 1 0.026

ARLETH AJ 1 0.026

ARLT VM 1 0.026

ARMBRUSTER FP 1 0.026

ARMELLONI S 1 0.026

ARMSTRONG MJ 1 0.026

ARMUGAM A 1 0.026

ARNABOLDI L 1 0.026

ARNLOV J 1 0.026

ARNOLD JMO 1 0.026

ARNONI CP 1 0.026

ARNSTEIN M 1 0.026

AROEIRA LS 1 0.026

ARONSON PS 1 0.026

AROOR A 1 0.026

ARORA MK 1 0.026

ARORA S 1 0.026

AROS C 1 0.026

AROUNE D 1 0.026

AROZAL W 1 0.026

ARROYO D 1 0.026

ARROYO FEG 1 0.026

ARRUDA JAL 1 0.026

ARSENA R 1 0.026

ARSENIJEVIC Y 1 0.026

ARTHAM S 1 0.026

ARTURI F 1 0.026

ARTZ N 1 0.026

ARUN A 1 0.026

ARYA A 1 0.026

ARYA DS 1 0.026

ASADA M 1 0.026

ASADA N 1 0.026

ASAHARA T 1 0.026

ASAKURA J 1 0.026

ASANO K 1 0.026

ASANUMA H 1 0.026

ASANUMA K 1 0.026

ASANUMA M 1 0.026

ASCHAUER S 1 0.026

ASCHENBACH JR 1 0.026

ASCHNER P 1 0.026

ASCI G 1 0.026

ASEMI Z 1 0.026

ASHANKYTY IM 1 0.026

ASHIQUE M 1 0.026

ASHOKKUMAR N 1 0.026

ASHRAFI-JIGHEH Z 1 0.026

ASHRAFIAN H 1 0.026

ASHRAFIZADEH M 1 0.026

ASIAEE F 1 0.026

ASKANASE A 1 0.026

ASKAR ME 1 0.026

ASKARI B 1 0.026

ASLAM AA 1 0.026

ASLAM S 1 0.026

ASLAN S 1 0.026

ASLANI S 1 0.026

ASPICHUETA P 1 0.026

ASSA S 1 0.026

ASSAN R 1 0.026

ASSAREHZADEGAN MA 1 0.026

ASSMANN TS 1 0.026

ASTIZ S 1 0.026

ATABEK ME 1 0.026

ATALAY H 1 0.026

ATAMAN E 1 0.026

ATAR D 1 0.026

ATASEVEN H 1 0.026

ATHANASIOU T 1 0.026

ATHYROS VG 1 0.026

ATIK T 1 0.026

ATIYA A 1 0.026

ATKIN SL 1 0.026

ATKINSON J 1 0.026

ATKINSON JM 1 0.026

ATKINSON M 1 0.026

ATKINSON MA 1 0.026

ATOBATELE AG 1 0.026

ATOH K 1 0.026

ATTA MG 1 0.026

ATTA SN 1 0.026

ATTIE AD 1 0.026

ATTIE-BITACH T 1 0.026

ATWA MA 1 0.026

ATZPODIEN EA 1 0.026

AU E 1 0.026

AU YW 1 0.026

AUBE J 1 0.026

AUDOLLENT S 1 0.026

AUER S 1 0.026

AUERBACH AD 1 0.026

AUGE J 1 0.026

AUGER-MESSIER M 1 0.026

AUGUSTINE JJ 1 0.026

AUKRUST P 1 0.026

AUNG HH 1 0.026

AUTIOHARMAINEN H 1 0.026

AVANIADI D 1 0.026

AVASARE RS 1 0.026

AVCI GA 1 0.026

AVETA T 1 0.026

AVILA C 1 0.026

AVILA G 1 0.026

AVILA-DIAZ M 1 0.026

AVILA-FERRUFINO A 1 0.026

AVILA-RODRIGUEZ MF 1 0.026

AVRAM D 1 0.026

AWAD A 1 0.026

AWAD AM 1 0.026

AWAD MM 1 0.026

AXELSON C 1 0.026

AYERS C 1 0.026

AZAMI N 1 0.026

AZANCOT MA 1 0.026

AZEVEDO RD 1 0.026

AZIBANI F 1 0.026

AZIMAITI M 1 0.026

AZIRIOVA S 1 0.026

AZIZ F 1 0.026

AZIZ MAA 1 0.026

AZZARONE B 1 0.026

AZZI J 1 0.026

BABA I 1 0.026

BABA K 1 0.026

BABA R 1 0.026

BABAN B 1 0.026

BABANY G 1 0.026

BABAYAN R 1 0.026

BABEL N 1 0.026

BACH JF 1 0.026

BACHAALANY S 1 0.026

BACHMANN S 1 0.026

BACKENROTH R 1 0.026

BACON SL 1 0.026

BADAL SS 1 0.026

BADARY DM 1 0.026

BADGER HS 1 0.026

BAE EH 1 0.026

BAE JH 1 0.026

BAE JS 1 0.026

BAE KH 1 0.026

BAE SS 1 0.026

BAE YK 1 0.026

BAE YU 1 0.026

BAEK SH 1 0.026

BAEZA-BERMEJILLO C 1 0.026

BAGATURIYA ET 1 0.026

BAGI Z 1 0.026

BAGNELL CR 1 0.026

BAGNOLI P 1 0.026

BAGREE S 1 0.026

BAGROV AY 1 0.026

BAHAMONDE J 1 0.026

BAHEKAR RH 1 0.026

BAHMANI M 1 0.026

BAHRA M 1 0.026

BAHRAMBEIGI S 1 0.026

BAI B 1 0.026

BAI CH 1 0.026

BAI CX 1 0.026

BAI Q 1 0.026

BAI ST 1 0.026

BAI X 1 0.026

BAIGENT C 1 0.026

BAILLET S 1 0.026

BAILLY F 1 0.026

BAIN RP 1 0.026

BAJWA J 1 0.026

BAK M 1 0.026

BAKAEEN FG 1 0.026

BAKEINE GJ 1 0.026

BAKER AH 1 0.026

BAKER D 1 0.026

BAKER JV 1 0.026

BAKER RJ 1 0.026

BAKER ST 1 0.026

BAKHREBAH MA 1 0.026

BAKIR EA 1 0.026

BAKR MA 1 0.026

BAKRIS G 1 0.026

BAKRIS GL 1 0.026

BALASUBRAMANIAN M 1 0.026

BALBI APC 1 0.026

BALDWIN MD 1 0.026

BALFAGON G 1 0.026

BALFE JW 1 0.026

BALIOVA M 1 0.026

BALL SD 1 0.026

BALLA S 1 0.026

BALOCHNEJADMOJARRAD T 1 0.026

BALOGH A 1 0.026

BALTATU OC 1 0.026

BAMGBOLA O 1 0.026

BANAL C 1 0.026

BANAS MC 1 0.026

BANBURY L 1 0.026

BANDIN F 1 0.026

BANDYOPADHYAY D 1 0.026

BANERJEE J 1 0.026

BANERJEE K 1 0.026

BANFI E 1 0.026

BANFI G 1 0.026

BANG BK 1 0.026

BANG C 1 0.026

BANG K 1 0.026

BANGA A 1 0.026

BANGALORE S 1 0.026

BANGSTAD HJ 1 0.026

BANIAHMAD A 1 0.026

BANKOVIC-CALIC N 1 0.026

BANOS G 1 0.026

BANSAL N 1 0.026

BANU K 1 0.026

BAO HK 1 0.026

BAO K 1 0.026

BAO LP 1 0.026

BAO NN 1 0.026

BAO RY 1 0.026

BAO WF 1 0.026

BAO XB 1 0.026

BAO YS 1 0.026

BAO YX 1 0.026

BAO ZY 1 0.026

BARADARAN A 1 0.026

BARAGHITHY S 1 0.026

BARALDI O 1 0.026

BARANY P 1 0.026

BARANYAI T 1 0.026

BARAT A 1 0.026

BARATI M 1 0.026

BARBAR M 1 0.026

BARBERIS AM 1 0.026

BARBET C 1 0.026

BARBOSA APF 1 0.026

BARBOSA MF 1 0.026

BARBU A 1 0.026

BARGMAN JM 1 0.026

BARICOS WH 1 0.026

BARIETY J 1 0.026

BARMA S 1 0.026

BARMAN S 1 0.026

BARNES J 1 0.026

BARNETT AH 1 0.026

BARNETT LMA 1 0.026

BAROLI A 1 0.026

BARON W 1 0.026

BARONI EA 1 0.026

BARR S 1 0.026

BARRERA F 1 0.026

BARRETO GE 1 0.026

BARRIERE DA 1 0.026

BARROS C 1 0.026

BARRY B 1 0.026

BARSOTTI P 1 0.026

BARTOLOMAEUS H 1 0.026

BARTRAM MP 1 0.026

BARZAGHI F 1 0.026

BARZILAY JI 1 0.026

BASARALI MK 1 0.026

BASHIR M 1 0.026

BASIC-JUKIC N 1 0.026

BASIRI A 1 0.026

BASSERI B 1 0.026

BASSI R 1 0.026

BASSIOUNI YA 1 0.026

BASSO M 1 0.026

BASSUK JH 1 0.026

BASTA B 1 0.026

BASTACKY SI 1 0.026

BATAL I 1 0.026

BATHGATE RAD 1 0.026

BATLLE D 1 0.026

BATTAGLIA Y 1 0.026

BATTAGLIA-HSU SF 1 0.026

BATTISHA A 1 0.026

BATUMAN V 1 0.026

BATZ C 1 0.026

BAUER AC 1 0.026

BAUER C 1 0.026

BAUERLE T 1 0.026

BAUMGARTEN R 1 0.026

BAUWENS M 1 0.026

BAX JJ 1 0.026

BAYARRE HD 1 0.026

BAYLISS G 1 0.026

BAYNES J 1 0.026

BAYNES JW 1 0.026

BAYOMI HS 1 0.026

BAYRAMOGLU A 1 0.026

BAZIN R 1 0.026

BEARD DR 1 0.026

BEATON H 1 0.026

BEATTIE PE 1 0.026

BEAUDET N 1 0.026

BEAUDREUIL S 1 0.026

BEBU I 1 0.026

BECHERUCCI F 1 0.026

BECHTEL-WALZ W 1 0.026

BECHTOLD SM 1 0.026

BECK B 1 0.026

BECK PL 1 0.026

BECKER G 1 0.026

BECKERMAN P 1 0.026

BECKERS A 1 0.026

BEDIR A 1 0.026

BEIROWSKI B 1 0.026

BEITZKE D 1 0.026

BEKER MC 1 0.026

BEKFILAVIOGLU G 1 0.026

BEKTAS O 1 0.026

BELAIR MF 1 0.026

BELENCHIA A 1 0.026

BELFAIR N 1 0.026

BELIO-CARO F 1 0.026

BELL SC 1 0.026

BELLAMY CO 1 0.026

BELLEVILLE K 1 0.026

BELLIN G 1 0.026

BELLINGHIERI G 1 0.026

BELLON JEM 1 0.026

BELLOVICH KA 1 0.026

BELLUMKONDA L 1 0.026

BELTRAMO E 1 0.026

BEN NASR M 1 0.026

BEN-DOV IZ 1 0.026

BENALI S 1 0.026

BENARDEAU A 1 0.026

BENCHETRIT S 1 0.026

BENDE RJ 1 0.026

BENDERSKY N 1 0.026

BENER A 1 0.026

BENETOS A 1 0.026

BENEZECH C 1 0.026

BENHASSINE S 1 0.026

BENITO-MARTINEZ S 1 0.026

BENIZIO E 1 0.026

BENJARAM S 1 0.026

BENNET W 1 0.026

BENNETT BL 1 0.026

BENNETT N 1 0.026

BENTER IF 1 0.026

BENTLEY L 1 0.026

BENWAY CJ 1 0.026

BENZER M 1 0.026

BENZING T 1 0.026

BERARDI C 1 0.026

BERCHTOLD L 1 0.026

BERCHUCK JE 1 0.026

BERE L 1 0.026

BERECEK KH 1 0.026

BERENGUER M 1 0.026

BEREZOV TT 1 0.026

BERG KH 1 0.026

BERG T 1 0.026

BERG TJ 1 0.026

BERGER SP 1 0.026

BERGMAN SJ 1 0.026

BERGMARK BA 1 0.026

BERK M 1 0.026

BERNABEU C 1 0.026

BERNARD C 1 0.026

BERNARDO J 1 0.026

BERNARDO JF 1 0.026

BERNARDO V 1 0.026

BERNICH P 1 0.026

BERNSTEIN EA 1 0.026

BERNSTEIN KE 1 0.026

BERNTHALER A 1 0.026

BERRERA M 1 0.026

BERRETTA AA 1 0.026

BERRUTTI M 1 0.026

BERTAGLIA E 1 0.026

BERTANI T 1 0.026

BERTASSOLI BM 1 0.026

BERTHIER CC 1 0.026

BERTINAT R 1 0.026

BERTOLOTTI A 1 0.026

BERTRAM A 1 0.026

BERTRAM CC 1 0.026

BERTRAM T 1 0.026

BERTRAND-MICHEL J 1 0.026

BERTSCH S 1 0.026

BESSA SSE 1 0.026

BESSHO R 1 0.026

BESZTERCEI B 1 0.026

BETENSKY R 1 0.026

BETENSKY RA 1 0.026

BETHUNAICKAN R 1 0.026

BETJES MGH 1 0.026

BEVINGTON A 1 0.026

BEX S 1 0.026

BEYER S 1 0.026

BEZERRA ELM 1 0.026

BHADAURIA D 1 0.026

BHAMBHANI V 1 0.026

BHANDARI S 1 0.026

BHANDARU M 1 0.026

BHANDARY B 1 0.026

BHANGAL G 1 0.026

BHANGUI P 1 0.026

BHANSALI A 1 0.026

BHANWER AJS 1 0.026

BHAT MA 1 0.026

BHAT ZY 1 0.026

BHATIA J 1 0.026

BHATT U 1 0.026

BHATTA A 1 0.026

BHATTACHARJEE S 1 0.026

BHATTACHARYA P 1 0.026

BHAVSAR NA 1 0.026

BHEDA-MALGE A 1 0.026

BI C 1 0.026

BI G 1 0.026

BI JG 1 0.026

BI KS 1 0.026

BI LQ 1 0.026

BI TT 1 0.026

BI XJ 1 0.026

BI XM 1 0.026

BI YL 1 0.026

BI-CHENG L 1 0.026

BIAN Y 1 0.026

BIANCARI F 1 0.026

BIANCHI A 1 0.026

BIANCHI C 1 0.026

BIANCHI F 1 0.026

BIANCHIMANO B 1 0.026

BIBEROGLU S 1 0.026

BIDASEE K 1 0.026

BIENAIME F 1 0.026

BIENIAS B 1 0.026

BIERHAUS A 1 0.026

BIESSEN EAL 1 0.026

BIGE N 1 0.026

BIGLEY A 1 0.026

BIHOREAU MT 1 0.026

BIJKERK R 1 0.026

BILAN VP 1 0.026

BILDACI TB 1 0.026

BILGIC MA 1 0.026

BILKAN CM 1 0.026

BILKENROTH U 1 0.026

BILLAH MM 1 0.026

BILO HJG 1 0.026

BILOUS R 1 0.026

BILOUS RW 1 0.026

BILTON D 1 0.026

BIN HONG S 1 0.026

BIN MUHSINAH A 1 0.026

BIN W 1 0.026

BINDA V 1 0.026

BINDER C 1 0.026

BINDER J 1 0.026

BINGHAM V 1 0.026

BINGOL SA 1 0.026

BIONDI ML 1 0.026

BIRENDRA R 1 0.026

BIRNBAUM MJ 1 0.026

BIRNBAUMER L 1 0.026

BISGAARD LS 1 0.026

BISHOP K 1 0.026

BISPO RKD 1 0.026

BISWAS R 1 0.026

BISWAS S 1 0.026

BITZAN M 1 0.026

BIWERSI J 1 0.026

BIYIK M 1 0.026

BJORNSSON ES 1 0.026

BLACKWELL TS 1 0.026

BLADES EW 1 0.026

BLAEKER H 1 0.026

BLAHO M 1 0.026

BLANC T 1 0.026

BLANCHARD E 1 0.026

BLANCHARD O 1 0.026

BLANCHER C 1 0.026

BLAND E 1 0.026

BLANKENSTEIN KI 1 0.026

BLANKESTIJN PJ 1 0.026

BLOCHER R 1 0.026

BLOOM RD 1 0.026

BLOOMFIELD DM 1 0.026

BLUMENTHAL S 1 0.026

BLYTH KG 1 0.026

BOA AN 1 0.026

BOCHI GV 1 0.026

BODANSKY HJ 1 0.026

BODNER J 1 0.026

BOEHMER K 1 0.026

BOEHMER KP 1 0.026

BOELS MGS 1 0.026

BOER MW 1 0.026

BOER P 1 0.026

BOERO R 1 0.026

BOERTIEN WE 1 0.026

BOETTCHER C 1 0.026

BOGA S 1 0.026

BOGENSCHUTZ O 1 0.026

BOGER CA 1 0.026

BOHLAND C 1 0.026

BOHLE RM 1 0.026

BOHLENDER JM 1 0.026

BOHM M 1 0.026

BOHUSLAVOVA R 1 0.026

BOIM MA 1 0.026

BOIMVASER S 1 0.026

BOING AN 1 0.026

BOINI KM 1 0.026

BOITARD C 1 0.026

BOJKOVA M 1 0.026

BOKER K 1 0.026

BOLDIN M 1 0.026

BOLES TH 1 0.026

BOLETIS JN 1 0.026

BOLETTA A 1 0.026

BOLKENT S 1 0.026

BOLLI P 1 0.026

BOLLICK YS 1 0.026

BOLLINENI S 1 0.026

BOLLINGER E 1 0.026

BOLLMANN A 1 0.026

BOLLOW E 1 0.026

BOMBELLI S 1 0.026

BOMMAYA G 1 0.026

BONA SR 1 0.026

BOND GJ 1 0.026

BONDERMAN D 1 0.026

BONDEVA T 1 0.026

BONG Z 1 0.026

BONGIOVANNI D 1 0.026

BONINO LD 1 0.026

BONNEFOY A 1 0.026

BONNET F 1 0.026

BONNY O 1 0.026

BONOMINI F 1 0.026

BOON MR 1 0.026

BOONLOH K 1 0.026

BOOTH JW 1 0.026

BORATYNSKA M 1 0.026

BORCHI B 1 0.026

BOREM PM 1 0.026

BORGES AH 1 0.026

BORGES RL 1 0.026

BORGHETTI G 1 0.026

BORIGHT AP 1 0.026

BORLAND TM 1 0.026

BORLAUG BA 1 0.026

BOROUMAND B 1 0.026

BORRELLI S 1 0.026

BORRI A 1 0.026

BORROWS RJ 1 0.026

BORSCHEWSKI A 1 0.026

BORZAN V 1 0.026

BOSC LVG 1 0.026

BOSCH E 1 0.026

BOSE M 1 0.026

BOSOMWORTH M 1 0.026

BOSSE Y 1 0.026

BOSSI F 1 0.026

BOSTEEN MH 1 0.026

BOTA S 1 0.026

BOTEANU RM 1 0.026

BOTELHO-SANTOS GA 1 0.026

BOTEY A 1 0.026

BOTTAI M 1 0.026

BOTTINGER E 1 0.026

BOTTIO T 1 0.026

BOU-GHARIOS G 1 0.026

BOUCHER G 1 0.026

BOUCHER GG 1 0.026

BOUGNERES P 1 0.026

BOULANGER JM 1 0.026

BOUSCHBACHER M 1 0.026

BOUTROS PC 1 0.026

BOUVET C 1 0.026

BOVER J 1 0.026

BOVO G 1 0.026

BOWDEN DW 1 0.026

BOWMAN LJ 1 0.026

BOWSKILL BB 1 0.026

BOWSMAN LM 1 0.026

BOYD A 1 0.026

BOYER N 1 0.026

BOYLAN M 1 0.026

BOZDOG G 1 0.026

BOZKURT A 1 0.026

BRAAM B 1 0.026

BRABANT G 1 0.026

BRACHEMI S 1 0.026

BRACQUART D 1 0.026

BRADY J 1 0.026

BRAGGION C 1 0.026

BRAHA A 1 0.026

BRAHIMI N 1 0.026

BRAMANTI P 1 0.026

BRAND K 1 0.026

BRANDAN E 1 0.026

BRANDENBURG V 1 0.026

BRANDINA R 1 0.026

BRANDS M 1 0.026

BRANDS MW 1 0.026

BRANDT M 1 0.026

BRANDT S 1 0.026

BRANTON MH 1 0.026

BRAULT A 1 0.026

BRAUNAGEL M 1 0.026

BRAVIN AM 1 0.026

BREAUX C 1 0.026

BREIDENBACH JD 1 0.026

BRENNAN DC 1 0.026

BRENNAN MF 1 0.026

BRENNEMAN J 1 0.026

BRENNER BM 1 0.026

BRESCI S 1 0.026

BREUIL B 1 0.026

BREUNIG F 1 0.026

BREVET M 1 0.026

BREZZI B 1 0.026

BRIDEAU G 1 0.026

BRIDOUX F 1 0.026

BRIMBLE MA 1 0.026

BRINGS S 1 0.026

BRINKERHOFF BT 1 0.026

BRIONES AM 1 0.026

BRISCO MA 1 0.026

BRISKIN D 1 0.026

BRISKIN R 1 0.026

BRISMAR K 1 0.026

BRIVIO R 1 0.026

BROCCA A 1 0.026

BROCHERIOU I 1 0.026

BROCHERIOU-SPELLE I 1 0.026

BROCHET C 1 0.026

BROCHIER C 1 0.026

BRODSKY SV 1 0.026

BROMBERG J 1 0.026

BROOKS CR 1 0.026

BROUEILH M 1 0.026

BROUSSEAU T 1 0.026

BROWN CB 1 0.026

BROWN D 1 0.026

BROWN H 1 0.026

BROWN RS 1 0.026

BROWN S 1 0.026

BROWN TP 1 0.026

BRUBAKER C 1 0.026

BRUCE A 1 0.026

BRUCKNER P 1 0.026

BRUECKMANN M 1 0.026

BRUEMMER D 1 0.026

BRUENSING J 1 0.026

BRUHL B 1 0.026

BRUIJIN JA 1 0.026

BRUNATI AM 1 0.026

BRUNNER-LA ROCCA HP 1 0.026

BRUNS H 1 0.026

BRUNSKILL EW 1 0.026

BRUSEGHIN M 1 0.026

BRYN J 1 0.026

BU H 1 0.026

BU LL 1 0.026

BU PL 1 0.026

BUCALA R 1 0.026

BUCCELLA V 1 0.026

BUCH A 1 0.026

BUCH MH 1 0.026

BUCHARLES S 1 0.026

BUCHTA P 1 0.026

BUDAJ A 1 0.026

BUDAKOVIC A 1 0.026

BUDAS GR 1 0.026

BUEMI A 1 0.026

BUENDGENS L 1 0.026

BUENDIA ASA 1 0.026

BUFFIN-MEYER B 1 0.026

BUHL KB 1 0.026

BUKOSZA EN 1 0.026

BUKOSZA N 1 0.026

BULL SB 1 0.026

BULLSHOE T 1 0.026

BULUS N 1 0.026

BULWER BE 1 0.026

BUMGARDNER GL 1 0.026

BUNDAK R 1 0.026

BUNEL V 1 0.026

BUNNAPRADIST S 1 0.026

BUOB D 1 0.026

BUQUE X 1 0.026

BURCHILL L 1 0.026

BURCKHARDT BC 1 0.026

BURCKHARDT G 1 0.026

BURCZYNSKI FJ 1 0.026

BURDMANN EA 1 0.026

BURFORD JL 1 0.026

BURGESS DR 1 0.026

BURGESS E 1 0.026

BURGHI V 1 0.026

BURGOS D 1 0.026

BURGOS-SANTAMARIA D 1 0.026

BURLET-SCHILTZ O 1 0.026

BURNS-KURTIS CL 1 0.026

BURTIN M 1 0.026

BUSCEMI S 1 0.026

BUSCH AE 1 0.026

BUSCH M 1 0.026

BUSSOLATI B 1 0.026

BUTT AA 1 0.026

BUTT N 1 0.026

BUTTNER R 1 0.026

BUTUROVIC-PONIKVAR J 1 0.026

BUYUKBAS S 1 0.026

BUZAS R 1 0.026

BUZKOVA P 1 0.026

BYARD RW 1 0.026

BYUN DW 1 0.026

CABARKAPA VS 1 0.026

CABELLO M 1 0.026

CABEZAS F 1 0.026

CABIEDES-CONTRERAS J 1 0.026

CABRERA WM 1 0.026

CACHOFEIRO V 1 0.026

CACOUB P 1 0.026

CADAMURO J 1 0.026

CAGARELLI T 1 0.026

CAGNEY G 1 0.026

CAI CJ 1 0.026

CAI FH 1 0.026

CAI HB 1 0.026

CAI HD 1 0.026

CAI HQ 1 0.026

CAI KR 1 0.026

CAI Q 1 0.026

CAI RJ 1 0.026

CAI SY 1 0.026

CAI WB 1 0.026

CAI WJ 1 0.026

CAI XJ 1 0.026

CAI XT 1 0.026

CAI YC 1 0.026

CAI YP 1 0.026

CAI YT 1 0.026

CAI YW 1 0.026

CAI ZW 1 0.026

CAIRNS C 1 0.026

CAKALAGAOGLU F 1 0.026

CALABRESE MF 1 0.026

CALDAS Y 1 0.026

CALDWELL RB 1 0.026

CALDWELL RW 1 0.026

CALERO D 1 0.026

CALISE D 1 0.026

CALLE P 1 0.026

CALLERA G 1 0.026

CALLEROS L 1 0.026

CALLES J 1 0.026

CALMUS Y 1 0.026

CALO LA 1 0.026

CALOSI L 1 0.026

CALUO-RUBIO M 1 0.026

CALVIN AD 1 0.026

CALVO-RUBIO M 1 0.026

CAMARGO EG 1 0.026

CAMERON A 1 0.026

CAMERON JS 1 0.026

CAMERON KO 1 0.026

CAMMALLERI M 1 0.026

CAMP JR 1 0.026

CAMPA C 1 0.026

CAMPANHOLLE G 1 0.026

CAMPBELL MS 1 0.026

CAMPBELL NRC 1 0.026

CAMPBELL TS 1 0.026

CAMPBELL WB 1 0.026

CAMPISI P 1 0.026

CAMPISTOL JM 1 0.026

CAMPOS LA 1 0.026

CAMPOY AHG 1 0.026

CAMSARI T 1 0.026

CAMUS M 1 0.026

CAN B 1 0.026

CANALE D 1 0.026

CANBAY A 1 0.026

CANDAN S 1 0.026

CANDIANO G 1 0.026

CANDIDO R 1 0.026

CANETTA PA 1 0.026

CANNATA-ANDIA JB 1 0.026

CANNING P 1 0.026

CANNON CP 1 0.026

CANNON MV 1 0.026

CANO-PENALVER JL 1 0.026

CANTARELL C 1 0.026

CANTERO-NAVARRO E 1 0.026

CANTIN JF 1 0.026

CANTLEY L 1 0.026

CAO AH 1 0.026

CAO D 1 0.026

CAO H 1 0.026

CAO J 1 0.026

CAO JL 1 0.026

CAO JW 1 0.026

CAO KJ 1 0.026

CAO LY 1 0.026

CAO M 1 0.026

CAO P 1 0.026

CAO Q 1 0.026

CAO R 1 0.026

CAO S 1 0.026

CAO T 1 0.026

CAO WF 1 0.026

CAO WJ 1 0.026

CAO XL 1 0.026

CAO Y 1 0.026

CAO YC 1 0.026

CAO YF 1 0.026

CAO YH 1 0.026

CAO YL 1 0.026

CAO YP 1 0.026

CAO ZY 1 0.026

CAPASSO JM 1 0.026

CAPLAN NM 1 0.026

CAPOLONGO G 1 0.026

CAPPUCCILLI M 1 0.026

CAPUTO P 1 0.026

CARAMORI MLA 1 0.026

CARBONE M 1 0.026

CARCASSI A 1 0.026

CAREW R 1 0.026

CARINPENOT E 1 0.026

CARLINI G 1 0.026

CARLOS CP 1 0.026

CARLSON EC 1 0.026

CARLSSON AC 1 0.026

CARLSSON PO 1 0.026

CARMACK T 1 0.026

CARMELIET P 1 0.026

CARMONA-ESCAMILLA MA 1 0.026

CARMOSINO M 1 0.026

CAROLI A 1 0.026

CARON K 1 0.026

CARON M 1 0.026

CARON N 1 0.026

CARPENE C 1 0.026

CARPENTIER AC 1 0.026

CARRARA F 1 0.026

CARRASCO S 1 0.026

CARRASCO-SANCHEZ FJ 1 0.026

CARRERA M 1 0.026

CARRERA SPP 1 0.026

CARRERO A 1 0.026

CARRERO JJ 1 0.026

CARRETTA D 1 0.026

CARRETTA R 1 0.026

CARROLL TJ 1 0.026

CARRUTHERS SG 1 0.026

CARTLAND SP 1 0.026

CARVAJAL G 1 0.026

CARVALHO A 1 0.026

CARVALHO AB 1 0.026

CARVALHO C 1 0.026

CARVALHO RJ 1 0.026

CARVALHO-FILHO RJ 1 0.026

CAS AD 1 0.026

CASABAR E 1 0.026

CASALENA G 1 0.026

CASARETTO A 1 0.026

CASSIDY D 1 0.026

CASSIS P 1 0.026

CASTANEDA-LARA LG 1 0.026

CASTEDAL M 1 0.026

CASTELLANO G 1 0.026

CASTELLAZZI L 1 0.026

CASTONGUAY L 1 0.026

CASTRO CH 1 0.026

CASTRO N 1 0.026

CASTRO NE 1 0.026

CAT AND 1 0.026

CATALDI C 1 0.026

CATANIA JM 1 0.026

CATANOZI S 1 0.026

CATTAN V 1 0.026

CATTANEO D 1 0.026

CATTRAN D 1 0.026

CATURELLI G 1 0.026

CAULEY JA 1 0.026

CAVAGLIERI RD 1 0.026

CAVAGLIERII RC 1 0.026

CAVALLARI LH 1 0.026

CAVALLI A 1 0.026

CAVALLI R 1 0.026

CAWOOD TJ 1 0.026

CAYIR A 1 0.026

CEBALLOS G 1 0.026

CEBALLOS-REYES G 1 0.026

CECCHINI AL 1 0.026

CECCHINI L 1 0.026

CEDERBAUM SD 1 0.026

CEDRINO M 1 0.026

CELEGHINI C 1 0.026

CELIE JWAM 1 0.026

CEMBRANEL BM 1 0.026

CEMLYN-JONES J 1 0.026

CENA T 1 0.026

CEOL M 1 0.026

CERASI E 1 0.026

CERASOLA G 1 0.026

CERIELLO A 1 0.026

CERQUEIRA R 1 0.026

CERULLO D 1 0.026

CERVANTES LG 1 0.026

CERVEANU-HOGAS A 1 0.026

CERVELLATI C 1 0.026

CERVENKA L 1 0.026

CERYCHOVA R 1 0.026

CESANA B 1 0.026

CESCUTTI J 1 0.026

CETINCAKMAK MG 1 0.026

CEVHER SC 1 0.026

CEVIK H 1 0.026

CEVIKBAS F 1 0.026

CHA EK 1 0.026

CHA SH 1 0.026

CHACKO BK 1 0.026

CHAE DW 1 0.026

CHAHAL PS 1 0.026

CHAHED S 1 0.026

CHAI CZ 1 0.026

CHAI L 1 0.026

CHAI LJ 1 0.026

CHAI S 1 0.026

CHAI XL 1 0.026

CHAI XP 1 0.026

CHAKRABORTY P 1 0.026

CHAKRAPANI LN 1 0.026

CHALMERS L 1 0.026

CHAMANI E 1 0.026

CHAMBERLIN MP 1 0.026

CHAN AWH 1 0.026

CHAN BM 1 0.026

CHAN CM 1 0.026

CHAN GSW 1 0.026

CHAN HC 1 0.026

CHAN HLY 1 0.026

CHAN JY 1 0.026

CHAN KC 1 0.026

CHAN SF 1 0.026

CHAN WK 1 0.026

CHAN YK 1 0.026

CHANDAN JS 1 0.026

CHANDARANA H 1 0.026

CHANDRA T 1 0.026

CHANDRASEKARAN K 1 0.026

CHANDRASHEKAR K 1 0.026

CHANG AS 1 0.026

CHANG BC 1 0.026

CHANG CC 1 0.026

CHANG CH 1 0.026

CHANG CM 1 0.026

CHANG CT 1 0.026

CHANG CY 1 0.026

CHANG DM 1 0.026

CHANG DY 1 0.026

CHANG FP 1 0.026

CHANG FR 1 0.026

CHANG H 1 0.026

CHANG HB 1 0.026

CHANG J 1 0.026

CHANG L 1 0.026

CHANG MS 1 0.026

CHANG MY 1 0.026

CHANG WH 1 0.026

CHANG WX 1 0.026

CHANG XT 1 0.026

CHANG YH 1 0.026

CHANG YM 1 0.026

CHANTY A 1 0.026

CHAO J 1 0.026

CHAO L 1 0.026

CHAO LK 1 0.026

CHAO SP 1 0.026

CHAO TK 1 0.026

CHAO XJ 1 0.026

CHAPDELAINE H 1 0.026

CHAPMAN A 1 0.026

CHARATCHAROENWITTHAYA P 1 0.026

CHARBGOO F 1 0.026

CHARLOUX A 1 0.026

CHARLTON M 1 0.026

CHARONIS A 1 0.026

CHARONIS AS 1 0.026

CHARPENTIER B 1 0.026

CHARTURVEDI N 1 0.026

CHARYTAN DM 1 0.026

CHATTIPAKORN SC 1 0.026

CHAU MKM 1 0.026

CHAUDHARY L 1 0.026

CHAUDHRY S 1 0.026

CHAUVEAU D 1 0.026

CHAVEZ Y 1 0.026

CHAWLA A 1 0.026

CHAWLA LS 1 0.026

CHAZOT PL 1 0.026

CHE XJ 1 0.026

CHEE GM 1 0.026

CHEEMA BS 1 0.026

CHEN BB 1 0.026

CHEN BC 1 0.026

CHEN BP 1 0.026

CHEN CCA 1 0.026

CHEN CD 1 0.026

CHEN CJ 1 0.026

CHEN CY 1 0.026

CHEN DF 1 0.026

CHEN DL 1 0.026

CHEN E 1 0.026

CHEN EY 1 0.026

CHEN FF 1 0.026

CHEN FM 1 0.026

CHEN FZ 1 0.026

CHEN GT 1 0.026

CHEN GZ 1 0.026

CHEN HA 1 0.026

CHEN HF 1 0.026

CHEN HX 1 0.026

CHEN HZ 1 0.026

CHEN IJ 1 0.026

CHEN JJ 1 0.026

CHEN JN 1 0.026

CHEN JT 1 0.026

CHEN KR 1 0.026

CHEN KX 1 0.026

CHEN LC 1 0.026

CHEN LG 1 0.026

CHEN LP 1 0.026

CHEN LW 1 0.026

CHEN ME 1 0.026

CHEN MH 1 0.026

CHEN MMHM 1 0.026

CHEN MY 1 0.026

CHEN P 1 0.026

CHEN PP 1 0.026

CHEN PS 1 0.026

CHEN QK 1 0.026

CHEN QO 1 0.026

CHEN QY 1 0.026

CHEN R 1 0.026

CHEN RJ 1 0.026

CHEN SS 1 0.026

CHEN T 1 0.026

CHEN WC 1 0.026

CHEN WM 1 0.026

CHEN XD 1 0.026

CHEN XX 1 0.026

CHEN YF 1 0.026

CHEN YS 1 0.026

CHEN YZ 1 0.026

CHEN ZG 1 0.026

CHEN ZL 1 0.026

CHEN ZM 1 0.026

CHENG A 1 0.026

CHENG AS 1 0.026

CHENG CM 1 0.026

CHENG FY 1 0.026

CHENG GM 1 0.026

CHENG HF 1 0.026

CHENG HT 1 0.026

CHENG HW 1 0.026

CHENG JF 1 0.026

CHENG JW 1 0.026

CHENG K 1 0.026

CHENG KC 1 0.026

CHENG KH 1 0.026

CHENG MC 1 0.026

CHENG MF 1 0.026

CHENG NN 1 0.026

CHENG PN 1 0.026

CHENG Q 1 0.026

CHENG S 1 0.026

CHENG SB 1 0.026

CHENG SJ 1 0.026

CHENG SW 1 0.026

CHENG TH 1 0.026

CHENG XB 1 0.026

CHENG XD 1 0.026

CHENG XN 1 0.026

CHENG XS 1 0.026

CHENG YF 1 0.026

CHENG YH 1 0.026

CHENG YX 1 0.026

CHENG Z 1 0.026

CHERCHI GM 1 0.026

CHERIF M 1 0.026

CHERNEY DZ 1 0.026

CHERNEY DZI 1 0.026

CHERTOW GM 1 0.026

CHERUKURI A 1 0.026

CHESNEY RW 1 0.026

CHESNUT K 1 0.026

CHEUK-CHUN S 1 0.026

CHEUNG CL 1 0.026

CHEUNG WW 1 0.026

CHEVALIER J 1 0.026

CHEVALIER JM 1 0.026

CHEVALIER P 1 0.026

CHI C 1 0.026

CHI LZ 1 0.026

CHI TT 1 0.026

CHI TY 1 0.026

CHI YQ 1 0.026

CHIANG HS 1 0.026

CHIANG MH 1 0.026

CHIANG PF 1 0.026

CHIANG SL 1 0.026

CHIARELLI F 1 0.026

CHIASSON JL 1 0.026

CHIBA S 1 0.026

CHIBA Y 1 0.026

CHIBBAR R 1 0.026

CHICKERING J 1 0.026

CHICKERING JG 1 0.026

CHIEN CT 1 0.026

CHIEN HP 1 0.026

CHIEN HY 1 0.026

CHIEN YS 1 0.026

CHIGNIER E 1 0.026

CHIKENJI T 1 0.026

CHILES MC 1 0.026

CHILTON R 1 0.026

CHIMA A 1 0.026

CHIN SH 1 0.026

CHIN YE 1 0.026

CHINELLO C 1 0.026

CHING-HA KB 1 0.026

CHINGA F 1 0.026

CHINNAKOTLA S 1 0.026

CHIOCCHETTI A 1 0.026

CHIOCCHINI ALC 1 0.026

CHIODO VA 1 0.026

CHIRINOS JA 1 0.026

CHIU HC 1 0.026

CHIU HW 1 0.026

CHIU YC 1 0.026

CHIU YH 1 0.026

CHIU YT 1 0.026

CHMURA A 1 0.026

CHO JH 1 0.026

CHO M 1 0.026

CHO SH 1 0.026

CHOCKALINGAM A 1 0.026

CHOI C 1 0.026

CHOI CW 1 0.026

CHOI DH 1 0.026

CHOI HJ 1 0.026

CHOI HS 1 0.026

CHOI JH 1 0.026

CHOI JS 1 0.026

CHOI JW 1 0.026

CHOI JY 1 0.026

CHOI KB 1 0.026

CHOI KC 1 0.026

CHOI MS 1 0.026

CHOI SR 1 0.026

CHOI WJ 1 0.026

CHOI YW 1 0.026

CHONCHOL M 1 0.026

CHOTAI M 1 0.026

CHOU CH 1 0.026

CHOU CK 1 0.026

CHOU HC 1 0.026

CHOU RH 1 0.026

CHOU SY 1 0.026

CHOU TC 1 0.026

CHOU YJ 1 0.026

CHOUDHARY NS 1 0.026

CHOUDHURI J 1 0.026

CHOUDHURY D 1 0.026

CHOUDHURY S 1 0.026

CHOUINARD P 1 0.026

CHOW J 1 0.026

CHOWDHURY MRH 1 0.026

CHOWDHURY UWK 1 0.026

CHOY SW 1 0.026

CHRISTEN U 1 0.026

CHRISTENSEN EI 1 0.026

CHRISTENSEN PK 1 0.026

CHRISTENSON RH 1 0.026

CHRISTOU GA 1 0.026

CHRISTOV M 1 0.026

CHU C 1 0.026

CHU CS 1 0.026

CHU D 1 0.026

CHU L 1 0.026

CHU M 1 0.026

CHU NA 1 0.026

CHU QQ 1 0.026

CHU SC 1 0.026

CHU TS 1 0.026

CHU WS 1 0.026

CHU YD 1 0.026

CHUA S 1 0.026

CHUANG KH 1 0.026

CHUANG P 1 0.026

CHUANG PC 1 0.026

CHUANG ST 1 0.026

CHUANG YL 1 0.026

CHUBAREV VN 1 0.026

CHUEAKULA N 1 0.026

CHUGH J 1 0.026

CHUGH S 1 0.026

CHUI DH 1 0.026

CHUN BMC 1 0.026

CHUN J 1 0.026

CHUNG BH 1 0.026

CHUNG CM 1 0.026

CHUNG FM 1 0.026

CHUNG H 1 0.026

CHUNG HF 1 0.026

CHUNG I 1 0.026

CHUNG KW 1 0.026

CHUNG SD 1 0.026

CHUNG SH 1 0.026

CHUNG SJ 1 0.026

CHUNG YH 1 0.026

CHUNG YS 1 0.026

CHUNG YY 1 0.026

CHURCH GM 1 0.026

CHURCHWELL MD 1 0.026

CHUTIPONGTANATE S 1 0.026

CHUU JJ 1 0.026

CHYAU CC 1 0.026

CIALDEA K 1 0.026

CIALKOWSKA-RYSZ A 1 0.026

CICOIRA M 1 0.026

CIGNI A 1 0.026

CIL Z 1 0.026

CIMADEVILLA C 1 0.026

CIMPONERIU D 1 0.026

CINA DP 1 0.026

CIOFFI G 1 0.026

CIPOLLA-NETO J 1 0.026

CIPU D 1 0.026

CIVANTOS E 1 0.026

CLARK AL 1 0.026

CLARK NR 1 0.026

CLARK RA 1 0.026

CLARKE J 1 0.026

CLARKE MV 1 0.026

CLAUT L 1 0.026

CLAVEL MA 1 0.026

CLAVREUL N 1 0.026

CLAY G 1 0.026

CLECH L 1 0.026

CLELAND E 1 0.026

CLELAND JG 1 0.026

CLELAND JGF 1 0.026

CLEMENS TL 1 0.026

CLEMENTE N 1 0.026

CLEMENTI A 1 0.026

CLERMONT AC 1 0.026

CLIFF CL 1 0.026

CLIFRON GD 1 0.026

CLIFTON I 1 0.026

CLOTET S 1 0.026

CLOUTIER L 1 0.026

COBLYN JS 1 0.026

COCA S 1 0.026

COCHAT P 1 0.026

COCKCROFT JR 1 0.026

CODOGNO I 1 0.026

CODREANU I 1 0.026

COELHO NR 1 0.026

COFFEY NJ 1 0.026

COGHLAN M 1 0.026

COHEN C 1 0.026

COHEN DJ 1 0.026

COHEN EP 1 0.026

COHEN M 1 0.026

COHEN-SOLAL A 1 0.026

COKORINOS E 1 0.026

COLHOUN HM 1 0.026

COLLIER JD 1 0.026

COLLIER T 1 0.026

COLLIGHAN R 1 0.026

COLLIGHAN RJ 1 0.026

COLLINS AR 1 0.026

COLLINS S 1 0.026

COLLINS-RACIE L 1 0.026

COLON S 1 0.026

COLVILLE-NASH P 1 0.026

COLZANI M 1 0.026

COMAI G 1 0.026

COMLEKCI A 1 0.026

CONCAS G 1 0.026

CONCHA M 1 0.026

CONDE-AGUDELO A 1 0.026

CONG XD 1 0.026

CONG YK 1 0.026

CONGET PA 1 0.026

CONGIU T 1 0.026

CONLEY SM 1 0.026

CONLIN CC 1 0.026

CONNAIRE J 1 0.026

CONNOR K 1 0.026

CONSTANTINESCU S 1 0.026

CONTTI MM 1 0.026

COOK T 1 0.026

COOL BL 1 0.026

COOMBES JD 1 0.026

COOMBES JS 1 0.026

COON JJ 1 0.026

COONEY SK 1 0.026

COOPER M 1 0.026

COPE L 1 0.026

COPPOLA L 1 0.026

CORAZZA U 1 0.026

CORBELLI A 1 0.026

CORBETT JA 1 0.026

CORBETTA B 1 0.026

CORCORAN JB 1 0.026

CORDEIRO BC 1 0.026

CORDEIRO VDC 1 0.026

CORDONNIER D 1 0.026

CORESH J 1 0.026

CORIC M 1 0.026

CORKEY BK 1 0.026

CORNEC-LE GALL E 1 0.026

CORNELIUS DC 1 0.026

CORNS PA 1 0.026

CORRADI B 1 0.026

CORREA FOB 1 0.026

CORREA JD 1 0.026

CORREA RRM 1 0.026

CORREA-GIANNELLA ML 1 0.026

CORREIA MJ 1 0.026

CORREIA TML 1 0.026

CORREMANS R 1 0.026

CORTES P 1 0.026

CORTEZ SL 1 0.026

CORTEZ-PINTO H 1 0.026

CORTVRINDT C 1 0.026

COSA F 1 0.026

COSELLI JS 1 0.026

COSGROVE D 1 0.026

COSKRAN TM 1 0.026

COSMI F 1 0.026

COSTA DDF 1 0.026

COSTA HD 1 0.026

COSTA-PESSOA JM 1 0.026

COTE AM 1 0.026

COTE N 1 0.026

COTRIM MD 1 0.026

COTTE L 1 0.026

COTTER G 1 0.026

COTTONE S 1 0.026

COUPES BM 1 0.026

COUSIN C 1 0.026

COUSIN M 1 0.026

COUTTS IGC 1 0.026

COUTURE C 1 0.026

COVENTRY SC 1 0.026

COWAN PJ 1 0.026

COWIE MR 1 0.026

COWLING BJ 1 0.026

COX EJ 1 0.026

COX JM 1 0.026

CRAIG JC 1 0.026

CRAIG K 1 0.026

CRAMER MJ 1 0.026

CRASS RL 1 0.026

CRAVEDI P 1 0.026

CRAVEN T 1 0.026

CRAVER L 1 0.026

CREAN D 1 0.026

CREPIN T 1 0.026

CRESCENZI M 1 0.026

CRESPO M 1 0.026

CRETU OM 1 0.026

CREW RJ 1 0.026

CRISPIM D 1 0.026

CRISTADORO S 1 0.026

CRISTOBAL-GARCIA M 1 0.026

CROFT KD 1 0.026

CROSSMAN DJ 1 0.026

CROUE A 1 0.026

CRUZ C 1 0.026

CRUZ E 1 0.026

CSETE M 1 0.026

CUCAK H 1 0.026

CUDNOCH-JEDRZEJEWSKA A 1 0.026

CUESTA C 1 0.026

CUEVAS CA 1 0.026

CUEVAS S 1 0.026

CUFI S 1 0.026

CUI DL 1 0.026

CUI DX 1 0.026

CUI HX 1 0.026

CUI J 1 0.026

CUI MJ 1 0.026

CUI NX 1 0.026

CUI R 1 0.026

CUI S 1 0.026

CUI SY 1 0.026

CUI TX 1 0.026

CUI WT 1 0.026

CUI YJ 1 0.026

CUI YM 1 0.026

CULVER S 1 0.026

CULVER SA 1 0.026

CUMMINGS BS 1 0.026

CUMMINS EP 1 0.026

CUMPELIK A 1 0.026

CUNHA OM 1 0.026

CUNNINGHAM EC 1 0.026

CUROVIC VR 1 0.026

CURRO G 1 0.026

CUTHBERT JJ 1 0.026

CVIJIC ME 1 0.026

CYBULSKY AV 1 0.026

CZARZASTA K 1 0.026

CZEKAY RP 1 0.026

CZERNICHOW P 1 0.026

D'ADDIO F 1 0.026

D'ALESSANDRO-GABAZZA CN 1 0.026

D'ALU F 1 0.026

D'ELIA JA 1 0.026

D'EMDEN H 1 0.026

DA COSTA CA 1 0.026

DA CRUZ FP 1 0.026

DA CUNHA FX 1 0.026

DA FONSECA RN 1 0.026

DA GLORIA MA 1 0.026

DA JJ 1 0.026

DA MOTA RK 1 0.026

DA ROCHA APM 1 0.026

DA SILVA AAS 1 0.026

DA SILVA CGA 1 0.026

DA SILVA DA 1 0.026

DA SILVA GB 1 0.026

DA SILVA KS 1 0.026

DA SILVA TM 1 0.026

DA SILVA VD 1 0.026

DA SILVA WIC 1 0.026

DA SILVA-LODGE M 1 0.026

DA SILVEIRA KD 1 0.026

DAAR ZS 1 0.026

DACCO V 1 0.026

DADKHAH-CHIMEH M 1 0.026

DAEHN I 1 0.026

DAGDELEN S 1 0.026

DAGENAIS F 1 0.026

DAGGUBATI R 1 0.026

DAHAB M 1 0.026

DAHAN I 1 0.026

DAHER ED 1 0.026

DAHLOF B 1 0.026

DAHLQVIST U 1 0.026

DAHLY AJ 1 0.026

DAHLY-VERNON A 1 0.026

DAI A 1 0.026

DAI C 1 0.026

DAI DF 1 0.026

DAI GY 1 0.026

DAI HZ 1 0.026

DAI L 1 0.026

DAI M 1 0.026

DAI QM 1 0.026

DAI TN 1 0.026

DAI XY 1 0.026

DAI ZK 1 0.026

DAKNA M 1 0.026

DAL MONTE M 1 0.026

DALIA M 1 0.026

DALLA VESTRA M 1 0.026

DALLAH ED 1 0.026

DALLEMAGNE C 1 0.026

DALLINGA-THIE GM 1 0.026

DAMASER MS 1 0.026

DAMMAN K 1 0.026

DAN C 1 0.026

DAN Y 1 0.026

DAN YY 1 0.026

DANCOSST DA 1 0.026

DANE MJC 1 0.026

DANELLI L 1 0.026

DANESHBOD Y 1 0.026

DANESHGARI F 1 0.026

DANESHPOUR N 1 0.026

DANG YY 1 0.026

DANGWAL S 1 0.026

DANIELS RD 1 0.026

DANIELSEN H 1 0.026

DANILOV SM 1 0.026

DANJUMA MI 1 0.026

DANKOWSKI R 1 0.026

DANTA CC 1 0.026

DAO HH 1 0.026

DAOUD G 1 0.026

DARAKHSHAN S 1 0.026

DARCIN T 1 0.026

DARENDELILER F 1 0.026

DARZACQ X 1 0.026

DARZI A 1 0.026

DAS B 1 0.026

DAS SAHA K 1 0.026

DASARE A 1 0.026

DASH D 1 0.026

DASHKIN MV 1 0.026

DASILVA I 1 0.026

DASILVA-JARDINE P 1 0.026

DASKALOPOULOU SS 1 0.026

DAVIDGE B 1 0.026

DAVIDSOHN N 1 0.026

DAVIDSON A 1 0.026

DAVIES MJ 1 0.026

DAVIES MR 1 0.026

DAVIES S 1 0.026

DAVILA RAV 1 0.026

DAVIS BJ 1 0.026

DAVIS J 1 0.026

DAVIS JC 1 0.026

DAVIS LK 1 0.026

DAVIS ME 1 0.026

DAVIS PF 1 0.026

DAVIS RJ 1 0.026

DAVIS SJ 1 0.026

DAVISON BA 1 0.026

DAWES M 1 0.026

DAY JR 1 0.026

DAY ML 1 0.026

DE ANDRADE LGM 1 0.026

DE ANGELIS N 1 0.026

DE ARRIBA G 1 0.026

DE BEER VJ 1 0.026

DE BEM GF 1 0.026

DE BOER HC 1 0.026

DE BRAGANCA AC 1 0.026

DE BRAY A 1 0.026

DE BRITO T 1 0.026

DE BROE ME 1 0.026

DE CAESTECKER CR 1 0.026

DE CAESTECKER M 1 0.026

DE CAL M 1 0.026

DE CARVALHO AAD 1 0.026

DE CARVALHO JAM 1 0.026

DE CARVALHO JJ 1 0.026

DE CARVALHO LCRM 1 0.026

DE CAVANAGH EMV 1 0.026

DE COSMO S 1 0.026

DE FRANCO E 1 0.026

DE GEUS HRH 1 0.026

DE GOUVILLE A 1 0.026

DE GROOT T 1 0.026

DE JAGER PL 1 0.026

DE KEULENAER GW 1 0.026

DE LAY MW 1 0.026

DE LAZZARI M 1 0.026

DE LEEUW AE 1 0.026

DE LEMOS ET 1 0.026

DE LEMOS JA 1 0.026

DE MAGALHAES ACM 1 0.026

DE MARCO S 1 0.026

DE MARE A 1 0.026

DE MEIJER VE 1 0.026

DE MELLO JCP 1 0.026

DE MIGUEL C 1 0.026

DE MOURA RS 1 0.026

DE MURO P 1 0.026

DE NICOLA L 1 0.026

DE NUCCI G 1 0.026

DE OLIVEIRA JC 1 0.026

DE OLIVEIRA SG 1 0.026

DE PASQUALE CG 1 0.026

DE PAULO LF 1 0.026

DE PREZ E 1 0.026

DE PREZ EG 1 0.026

DE RECHTER S 1 0.026

DE ROSA M 1 0.026

DE SEQUERA P 1 0.026

DE SIMONE G 1 0.026

DE SMEDT H 1 0.026

DE SOUZA BM 1 0.026

DE SOUZA ML 1 0.026

DE SOYZA A 1 0.026

DE TORRES I 1 0.026

DE VINUESA AG 1 0.026

DE VINUESA SG 1 0.026

DE VRIES DK 1 0.026

DE VRIES JJJ 1 0.026

DE VRIESE AS 1 0.026

DEAN H 1 0.026

DEAR JW 1 0.026

DEBELLE FD 1 0.026

DEBL K 1 0.026

DEBOER RA 1 0.026

DECHEND R 1 0.026

DECKERS M 1 0.026

DECRAMER S 1 0.026

DECUYPERE JP 1 0.026

DEDHAR S 1 0.026

DEDRICK RL 1 0.026

DEEB M 1 0.026

DEEKAJORNDECH T 1 0.026

DEELMAN L 1 0.026

DEEN PMT 1 0.026

DEFRONZO R 1 0.026

DEGRELL P 1 0.026

DEHILLOTTE C 1 0.026

DEHOUX M 1 0.026

DEIERHOI MH 1 0.026

DEININGER N 1 0.026

DEJONG S 1 0.026

DEL RE E 1 0.026

DEL RIZZO P 1 0.026

DEL TACCA M 1 0.026

DELEZOIDE AL 1 0.026

DELGADO G 1 0.026

DELGADO R 1 0.026

DELGADO V 1 0.026

DELGUSTE F 1 0.026

DELITSIKOU V 1 0.026

DELJANIN-ILIC M 1 0.026

DELLE H 1 0.026

DELLEGROTTAGLIE S 1 0.026

DELLES C 1 0.026

DELSANTE M 1 0.026

DELTOMBE C 1 0.026

DEMARCO V 1 0.026

DEMIR A 1 0.026

DEMIRCI MS 1 0.026

DEMIREL GY 1 0.026

DEMISSEI BG 1 0.026

DEN HARTOGH DJ 1 0.026

DEN HOEDT CH 1 0.026

DENC H 1 0.026

DENG AG 1 0.026

DENG BC 1 0.026

DENG F 1 0.026

DENG H 1 0.026

DENG K 1 0.026

DENG L 1 0.026

DENG MH 1 0.026

DENG QW 1 0.026

DENG TT 1 0.026

DENG WP 1 0.026

DENG WQ 1 0.026

DENG WW 1 0.026

DENG XQ 1 0.026

DENG XY 1 0.026

DENG Y 1 0.026

DENHEZ B 1 0.026

DENIS JA 1 0.026

DENT H 1 0.026

DENTELLI P 1 0.026

DENTI V 1 0.026

DEO M 1 0.026

DEPREM T 1 0.026

DERBALA M 1 0.026

DERIC M 1 0.026

DERKS H 1 0.026

DERSCHEID R 1 0.026

DESAI SS 1 0.026

DESAULNIERS J 1 0.026

DESCHENES I 1 0.026

DESCHODT-LANCKMAN MM 1 0.026

DESHMUKH HA 1 0.026

DESHMUKH PV 1 0.026

DESHPANDE SD 1 0.026

DESILVA K 1 0.026

DESMEDT S 1 0.026

DESMEDT V 1 0.026

DESMOND L 1 0.026

DESMOND M 1 0.026

DESSOUKY AA 1 0.026

DESTERKE C 1 0.026

DESVERGNE B 1 0.026

DETRAIT E 1 0.026

DETTLING A 1 0.026

DEVANEY JM 1 0.026

DEVARAJ S 1 0.026

DEVARAJAN P 1 0.026

DEVASIA A 1 0.026

DEVASSY JG 1 0.026

DEVOCELLE A 1 0.026

DEY A 1 0.026

DEYLGAT B 1 0.026

DHADUK R 1 0.026

DHANARAJ SA 1 0.026

DHAR P 1 0.026

DHAS Y 1 0.026

DI BARTOLO P 1 0.026

DI BELGIOJOSO GB 1 0.026

DI FABRIZIO E 1 0.026

DI FRAIA G 1 0.026

DI J 1 0.026

DI LEMBO S 1 0.026

DI MARIO U 1 0.026

DI PALMA A 1 0.026

DI PAOLO S 1 0.026

DI STEFANO V 1 0.026

DIACONU L 1 0.026

DIANZANI U 1 0.026

DIAO G 1 0.026

DIAZ MAS 1 0.026

DIAZ V 1 0.026

DIAZ-ENCARNACION MM 1 0.026

DIAZ-FLORES M 1 0.026

DIBLASIO-SMITH L 1 0.026

DICK EJ 1 0.026

DICKSON Z 1 0.026

DIEKMAN T 1 0.026

DIETERLE CD 1 0.026

DIEZ RRR 1 0.026

DIJKMAN H 1 0.026

DIKALOV SI 1 0.026

DILLY SA 1 0.026

DIMAGNO EP 1 0.026

DIMAGNO MJ 1 0.026

DINDA AK 1 0.026

DING CY 1 0.026

DING D 1 0.026

DING F 1 0.026

DING GJ 1 0.026

DING GL 1 0.026

DING HF 1 0.026

DING HH 1 0.026

DING J 1 0.026

DING JJ 1 0.026

DING JX 1 0.026

DING JY 1 0.026

DING KK 1 0.026

DING P 1 0.026

DING R 1 0.026

DING SS 1 0.026

DING W 1 0.026

DING XQ 1 0.026

DING YQ 1 0.026

DING Z 1 0.026

DING ZY 1 0.026

DINIZ C 1 0.026

DINOV B 1 0.026

DIRICAN A 1 0.026

DISTLER A 1 0.026

DITE P 1 0.026

DITTRICH A 1 0.026

DITTRICH H 1 0.026

DIVELLA C 1 0.026

DIWAKAR R 1 0.026

DIXIT V 1 0.026

DJOUSSE L 1 0.026

DJUDJAJ S 1 0.026

DJURIC M 1 0.026

DO NASCIMENTO PRP 1 0.026

DOBBERFUHL AD 1 0.026

DOBRIAN AD 1 0.026

DOBYNS A 1 0.026

DODESINI AR 1 0.026

DOEVENDANS PAFM 1 0.026

DOHERTY H 1 0.026

DOI K 1 0.026

DOI SAR 1 0.026

DOKTER MM 1 0.026

DOLADE N 1 0.026

DOLINSKI BM 1 0.026

DOLLER A 1 0.026

DOLLFUS H 1 0.026

DOM ZIM 1 0.026

DOMIENIK-KARLOWICZ J 1 0.026

DOMIN J 1 0.026

DOMINGUEZ AR 1 0.026

DOMINGUEZ GCS 1 0.026

DOMINGUEZ J 1 0.026

DOMINGUEZ JM 1 0.026

DOMINGUEZ-GIL B 1 0.026

DOMINIC EA 1 0.026

DOMON A 1 0.026

DOMSCH C 1 0.026

DON AS 1 0.026

DONA C 1 0.026

DONAGHUE K 1 0.026

DONALD JL 1 0.026

DONATI A 1 0.026

DONATO V 1 0.026

DONG C 1 0.026

DONG G 1 0.026

DONG H 1 0.026

DONG HB 1 0.026

DONG JC 1 0.026

DONG LJ 1 0.026

DONG LS 1 0.026

DONG QL 1 0.026

DONG XH 1 0.026

DONG XM 1 0.026

DONG YF 1 0.026

DONG YH 1 0.026

DONG YJ 1 0.026

DONG YP 1 0.026

DONG ZH 1 0.026

DONG ZY 1 0.026

DONGZHIM Z 1 0.026

DONNELLY-ROBERTS D 1 0.026

DONNELLY-ROBERTS DL 1 0.026

DONNER DG 1 0.026

DONNERT E 1 0.026

DONOHUE M 1 0.026

DOOLEY S 1 0.026

DORAN P 1 0.026

DORAN PP 1 0.026

DORGAN DJ 1 0.026

DORIA A 1 0.026

DORMANESH B 1 0.026

DOS REIS MA 1 0.026

DOS SANTOS-MACEDO F 1 0.026

DOTSCH J 1 0.026

DOU HC 1 0.026

DOU HJ 1 0.026

DOUCET L 1 0.026

DOUGLAS-DENTON R 1 0.026

DOUMAS M 1 0.026

DOUPIS J 1 0.026

DOUSDAMPANIS P 1 0.026

DOWDALL JF 1 0.026

DOWER K 1 0.026

DOWLING JP 1 0.026

DOWMAN JK 1 0.026

DOWNEY DG 1 0.026

DOWNTON M 1 0.026

DOYLE K 1 0.026

DOYLE KJ 1 0.026

DREL V 1 0.026

DRESSEL A 1 0.026

DRESSER GK 1 0.026

DREYZIN A 1 0.026

DRISCOLL KA 1 0.026

DROUIN D 1 0.026

DROUIN M 1 0.026

DRUCKER DJ 1 0.026

DRUMMEN GPC 1 0.026

DRYER SE 1 0.026

DSCHIETZIG TB 1 0.026

DU BOULLAY OT 1 0.026

DU CG 1 0.026

DU JH 1 0.026

DU JL 1 0.026

DU JW 1 0.026

DU LF 1 0.026

DU N 1 0.026

DU PC 1 0.026

DU X 1 0.026

DU XN 1 0.026

DU Y 1 0.026

DUAN AS 1 0.026

DUAN L 1 0.026

DUAN LJ 1 0.026

DUAN N 1 0.026

DUAN Q 1 0.026

DUAN SF 1 0.026

DUAN YQ 1 0.026

DUAN YR 1 0.026

DUAN ZY 1 0.026

DUANN P 1 0.026

DUARTE DB 1 0.026

DUARTE V 1 0.026

DUBANSKY BH 1 0.026

DUBE G 1 0.026

DUBEY NK 1 0.026

DUCA F 1 0.026

DUCASA GM 1 0.026

DUCASSE L 1 0.026

DUCHENE J 1 0.026

DUCKERS H 1 0.026

DUCLOS A 1 0.026

DUCLOUX D 1 0.026

DUEZ P 1 0.026

DUFF HJ 1 0.026

DUFFIN KL 1 0.026

DUGAS CM 1 0.026

DUGAST-DARZACQ C 1 0.026

DUGBARTEY GJ 1 0.026

DUHLI N 1 0.026

DUHRSEN U 1 0.026

DUIJS JMGJ 1 0.026

DUIN M 1 0.026

DUITMAN J 1 0.026

DULTZ G 1 0.026

DUMITRU RB 1 0.026

DUNEA G 1 0.026

DUNLAY SM 1 0.026

DUNN AC 1 0.026

DUNNE PD 1 0.026

DUPUY A 1 0.026

DURAND F 1 0.026

DURR M 1 0.026

DURRBACH A 1 0.026

DUSABIMANA T 1 0.026

DUTA C 1 0.026

DUVAL X 1 0.026

DUZENLI S 1 0.026

DWEEP H 1 0.026

DWIVEDI RS 1 0.026

DWORKIN LD 1 0.026

EARDLEY KS 1 0.026

EARLEY B 1 0.026

EASTWOOD JB 1 0.026

EATON DM 1 0.026

EBADI Z 1 0.026

EBER E 1 0.026

EBERHARDT W 1 0.026

EBID R 1 0.026

EBNER F 1 0.026

EBRAHIM N 1 0.026

EBY BK 1 0.026

ECHIDA Y 1 0.026

ECHOUFFO-TCHEUGUI JB 1 0.026

ECHTERMEYER F 1 0.026

ECKARDT KU 1 0.026

ECKERT AW 1 0.026

ECKHARDT H 1 0.026

ECONOMIDES AN 1 0.026

ECUYER C 1 0.026

EDELING M 1 0.026

EDELMAN RR 1 0.026

EDELMANN F 1 0.026

EDELSTEIN MH 1 0.026

EDER IE 1 0.026

EDER S 1 0.026

EDMAN K 1 0.026

EDMONDS DJ 1 0.026

EGEDY M 1 0.026

EGI Y 1 0.026

EGRANOV SD 1 0.026

EGUCHI Y 1 0.026

EHARA T 1 0.026

EHLING J 1 0.026

EHNERT S 1 0.026

EHRENSHAFT M 1 0.026

EHRICH JHH 1 0.026

EHRINPREIS M 1 0.026

EI I 1 0.026

EI-MAS MM 1 0.026

EICHINGER F 1 0.026

EICHNER G 1 0.026

EIGENTLER TK 1 0.026

EINOLLAHI B 1 0.026

EISELE C 1 0.026

EISELE G 1 0.026

EISER AR 1 0.026

EISSA LA 1 0.026

EKANAYAKE P 1 0.026

EKIMCI N 1 0.026

EKSTEDT M 1 0.026

EL CHAAR M 1 0.026

EL DEIN ABS 1 0.026

EL ESSAWY B 1 0.026

EL GAZZAR WB 1 0.026

EL HAFIDI M 1 0.026

EL KOSSI M 1 0.026

EL MOGHRABI S 1 0.026

EL SALEM A 1 0.026

EL-AOUFI S 1 0.026

EL-BASSOSSY HM 1 0.026

EL-BAZ MA 1 0.026

EL-GAYAR AM 1 0.026

EL-HUSSEINI A 1 0.026

EL-KADEM AH 1 0.026

EL-MAHDY NA 1 0.026

EL-MARAGHY NN 1 0.026

EL-MEANAWVY A 1 0.026

EL-REMESSY AB 1 0.026

EL-SAYAD ME 1 0.026

EL-SHERBINY M 1 0.026

EL-ZOGHBY ZM 1 0.026

ELACHOURI M 1 0.026

ELAGROUDY AE 1 0.026

ELBEIN SC 1 0.026

ELBORN JS 1 0.026

ELCIOGLU N 1 0.026

ELDOSOKY M 1 0.026

ELEFTHERIADIS T 1 0.026

ELETTI M 1 0.026

ELHASSAN E 1 0.026

ELKASHEF HA 1 0.026

ELKHAMMAS EA 1 0.026

ELKRIEF L 1 0.026

ELLARD S 1 0.026

ELLEZ MJT 1 0.026

ELLI A 1 0.026

ELLINGSEN AR 1 0.026

ELLIOT C 1 0.026

ELLIOTT JA 1 0.026

ELLIS D 1 0.026

ELLIS T 1 0.026

ELLISON DH 1 0.026

ELNAHAS AM 1 0.026

ELS V 1 0.026

ELSAED WM 1 0.026

ELSHAER SL 1 0.026

ELSHAZLY AM 1 0.026

ELSHERBINY H 1 0.026

ELSWEFY SE 1 0.026

ELTRICH N 1 0.026

EMANCIPATOR SN 1 0.026

EMANS ME 1 0.026

EMMERT A 1 0.026

EMRE S 1 0.026

EMREN SV 1 0.026

EMTERZ CA 1 0.026

EN-NIA A 1 0.026

ENAYATI P 1 0.026

ENCHA-RAZAVI F 1 0.026

ENCISO-MORENO JA 1 0.026

ENDEMANN DH 1 0.026

ENDLICH K 1 0.026

ENDLICH N 1 0.026

ENDO K 1 0.026

ENDO M 1 0.026

ENDO S 1 0.026

ENDO T 1 0.026

ENDOH D 1 0.026

ENDRE Z 1 0.026

ENGBERG A 1 0.026

ENGBERG AS 1 0.026

ENGEL S 1 0.026

ENGGAARD C 1 0.026

ENHESUREN MM 1 0.026

ENJOJI M 1 0.026

ENOMOTO T 1 0.026

ENRICH J 1 0.026

EO H 1 0.026

EPSTEIN M 1 0.026

ERBAS T 1 0.026

EREMINA V 1 0.026

EREN M 1 0.026

EREZ N 1 0.026

EREZ O 1 0.026

ERGENE O 1 0.026

ERHARDT M 1 0.026

ERICKSEN J 1 0.026

ERICSSON A 1 0.026

ERIGUCHI M 1 0.026

ERLANDSEN EJ 1 0.026

ERLICH JH 1 0.026

ERMER T 1 0.026

EROGLU FK 1 0.026

EROL I 1 0.026

ERTILAV M 1 0.026

ERWA W 1 0.026

ESBENSHADE T 1 0.026

ESBRIT P 1 0.026

ESCH KJ 1 0.026

ESCHER F 1 0.026

ESCOBALES N 1 0.026

ESCULPAVIT C 1 0.026

ESEN H 1 0.026

ESEN I 1 0.026

ESKENS BJM 1 0.026

ESMAEILI M 1 0.026

ESPINER EA 1 0.026

ESPINOSA V 1 0.026

ESPINOZA PA 1 0.026

ESPINOZA-PERALTA D 1 0.026

ESSALIHI R 1 0.026

ESSAWY M 1 0.026

ESTAQUE S 1 0.026

ESTEBAN H 1 0.026

ESTELLAT C 1 0.026

ETCHEVERS H 1 0.026

ETHEMOGLU S 1 0.026

ETHERINGTON C 1 0.026

ETHIER J 1 0.026

ETIENNE I 1 0.026

ETTEMA MAB 1 0.026

EUDY R 1 0.026

EUDY RJ 1 0.026

EULBERG D 1 0.026

EURICH D 1 0.026

EURICH F 1 0.026

EVAN AP 1 0.026

EVANGELISTA I 1 0.026

EVANS RA 1 0.026

EVSIKOV AV 1 0.026

EWART L 1 0.026

EWEN S 1 0.026

EYMIEUX S 1 0.026

EZAKI T 1 0.026

EZEKOWITZ JA 1 0.026

EZQUER FE 1 0.026

EZQUER ME 1 0.026

FABER KN 1 0.026

FABRE NT 1 0.026

FABRIS B 1 0.026

FABRIZI F 1 0.026

FACCHIN D 1 0.026

FACCHIN L 1 0.026

FACOETTI A 1 0.026

FACTOR SM 1 0.026

FADAEI R 1 0.026

FADER CM 1 0.026

FADI H 1 0.026

FAEDDA R 1 0.026

FAGA T 1 0.026

FAGERHOLM E 1 0.026

FAHERTY N 1 0.026

FAHMY H 1 0.026

FAIN S 1 0.026

FAIVRE A 1 0.026

FAKHOURI F 1 0.026

FALCK JR 1 0.026

FALCONE JC 1 0.026

FALCOZ PE 1 0.026

FALHAMMAR H 1 0.026

FALKE L 1 0.026

FALKNER B 1 0.026

FALLAH S 1 0.026

FALLAHZADEH MK 1 0.026

FALLO F 1 0.026

FAN CQ 1 0.026

FAN FL 1 0.026

FAN GP 1 0.026

FAN HW 1 0.026

FAN JH 1 0.026

FAN JM 1 0.026

FAN JS 1 0.026

FAN JY 1 0.026

FAN L 1 0.026

FAN LX 1 0.026

FAN M 1 0.026

FAN WX 1 0.026

FAN X 1 0.026

FAN XB 1 0.026

FAN XD 1 0.026

FAN XY 1 0.026

FAN ZC 1 0.026

FANDRIKS L 1 0.026

FANG DL 1 0.026

FANG HW 1 0.026

FANG HY 1 0.026

FANG J 1 0.026

FANG M 1 0.026

FANG ML 1 0.026

FANG MM 1 0.026

FANG MR 1 0.026

FANG Q 1 0.026

FANG QJ 1 0.026

FANG W 1 0.026

FANG XF 1 0.026

FANG XS 1 0.026

FANG Y 1 0.026

FANG YF 1 0.026

FANG YL 1 0.026

FANTAUZZI CB 1 0.026

FARBER E 1 0.026

FARD TK 1 0.026

FARHANGKHOEE H 1 0.026

FARIAS RE 1 0.026

FARID AS 1 0.026

FARINA M 1 0.026

FARKAS D 1 0.026

FARKAS L 1 0.026

FARKHONDEH T 1 0.026

FARNEY AC 1 0.026

FAROUK SS 1 0.026

FARRALL M 1 0.026

FARZANEH SH 1 0.026

FASCHING A 1 0.026

FASHINGBATTER LA 1 0.026

FASSIO F 1 0.026

FAULKNER J 1 0.026

FAULKNER M 1 0.026

FAURHOLT-JEPSEN D 1 0.026

FAVERO G 1 0.026

FAVI E 1 0.026

FAZIO MR 1 0.026

FEARON N 1 0.026

FEDER D 1 0.026

FEDOROVA OV 1 0.026

FEHRENBACH P 1 0.026

FEI S 1 0.026

FEI X 1 0.026

FEIFEL R 1 0.026

FEIN FS 1 0.026

FELDER TK 1 0.026

FELDMAN RD 1 0.026

FELDNER ACCA 1 0.026

FELDT S 1 0.026

FELIX S 1 0.026

FELKER GM 1 0.026

FELSEN D 1 0.026

FENG BY 1 0.026

FENG GW 1 0.026

FENG H 1 0.026

FENG LP 1 0.026

FENG M 1 0.026

FENG P 1 0.026

FENG Q 1 0.026

FENG X 1 0.026

FENG XY 1 0.026

FENG XZ 1 0.026

FENG YB 1 0.026

FENG YJ 1 0.026

FENG ZJ 1 0.026

FENG ZW 1 0.026

FENJVES ES 1 0.026

FENKEL JM 1 0.026

FERDER L 1 0.026

FERDER LF 1 0.026

FERDINANDY P 1 0.026

FERGUSON CM 1 0.026

FERGUSON N 1 0.026

FERGUSON RM 1 0.026

FERLICOT S 1 0.026

FERNANDES GS 1 0.026

FERNANDES KBP 1 0.026

FERNANDES R 1 0.026

FERNANDES TO 1 0.026

FERNANDES-SANTOS C 1 0.026

FERNANDEZ E 1 0.026

FERNANDEZ-JUAREZ G 1 0.026

FERNANDEZ-MARTIN JL 1 0.026

FERNANDEZ-MARTINEZ AB 1 0.026

FERNANDEZ-RODRIGUEZ CM 1 0.026

FERNANDEZ-VIZARRA P 1 0.026

FERRAJOLO C 1 0.026

FERRANDI M 1 0.026

FERRARESI M 1 0.026

FERRARIO C 1 0.026

FERREIRA AJ 1 0.026

FERREIRA AP 1 0.026

FERREIRA JP 1 0.026

FERRINI MG 1 0.026

FERRO CJ 1 0.026

FESHARAKI MG 1 0.026

FEUERSTEIN GZ 1 0.026

FEUILLET G 1 0.026

FICKER E 1 0.026

FIDLER ME 1 0.026

FIEDLER L 1 0.026

FIELD A 1 0.026

FIELD J 1 0.026

FIELD MJ 1 0.026

FIENGO L 1 0.026

FIGAROLA JL 1 0.026

FIGUEIRA MF 1 0.026

FIGUEROA S 1 0.026

FIGUEROA SM 1 0.026

FILIPPATOS G 1 0.026

FILIPPATOS GS 1 0.026

FILLAUS J 1 0.026

FINCH J 1 0.026

FINE DM 1 0.026

FINE LG 1 0.026

FINER G 1 0.026

FINGER EB 1 0.026

FINK HA 1 0.026

FINKELSTEIN F 1 0.026

FINN P 1 0.026

FINNEY C 1 0.026

FINOTTI BB 1 0.026

FIORENTINO TV 1 0.026

FIORINA P 1 0.026

FISCHER C 1 0.026

FISCHER M 1 0.026

FISHER DA 1 0.026

FIUZAT M 1 0.026

FIZER B 1 0.026

FLAMANT M 1 0.026

FLASCH AK 1 0.026

FLAVELL RA 1 0.026

FLEISCHER S 1 0.026

FLEMING JT 1 0.026

FLEMING LK 1 0.026

FLEMMING NB 1 0.026

FLISER D 1 0.026

FLOREANI A 1 0.026

FLORES JMM 1 0.026

FLORES O 1 0.026

FLORES S 1 0.026

FLORES WM 1 0.026

FLORESCU C 1 0.026

FLORESCU DF 1 0.026

FLORESCU MC 1 0.026

FLOREZ JC 1 0.026

FLORIJN BW 1 0.026

FLOTTE T 1 0.026

FLOTTE TR 1 0.026

FLUCHER K 1 0.026

FLUITT MB 1 0.026

FLYNN E 1 0.026

FODOR G 1 0.026

FOG-TONNESEN M 1 0.026

FOGARI R 1 0.026

FOLEY R 1 0.026

FOLLER M 1 0.026

FOLTZ W 1 0.026

FOMISON-NURSE IC 1 0.026

FONAROW GC 1 0.026

FONSECA DA 1 0.026

FONTECHA-BARRIUSO M 1 0.026

FONTOURA D 1 0.026

FORBES J 1 0.026

FORBES MS 1 0.026

FORDHAM M 1 0.026

FORETZ M 1 0.026

FORINO M 1 0.026

FORK C 1 0.026

FORLANI G 1 0.026

FORNONI A 1 0.026

FORSBLOM C 1 0.026

FORSLUND SK 1 0.026

FORSSMANN WG 1 0.026

FORTIN PR 1 0.026

FORTRIE G 1 0.026

FOSTER CS 1 0.026

FOSTER JE 1 0.026

FOULKE L 1 0.026

FOUNDS HW 1 0.026

FOUQUE D 1 0.026

FOX CS 1 0.026

FRADIN C 1 0.026

FRAGA CG 1 0.026

FRAGIADAKI M 1 0.026

FRAMARIN L 1 0.026

FRANCA-SILVA N 1 0.026

FRANCI B 1 0.026

FRANCIOSI J 1 0.026

FRANCIS H 1 0.026

FRANCISCO FA 1 0.026

FRANCO I 1 0.026

FRANCOZ C 1 0.026

FRANCQUE S 1 0.026

FRANCZYK B 1 0.026

FRANK AM 1 0.026

FRANKE FE 1 0.026

FRANKEL AH 1 0.026

FRANSSEN CF 1 0.026

FRANZEN S 1 0.026

FRAPPIER JY 1 0.026

FRASER I 1 0.026

FRASER SA 1 0.026

FRAYSSE J 1 0.026

FREEMAN M 1 0.026

FREI U 1 0.026

FRENAY ARS 1 0.026

FRENCH DM 1 0.026

FRENCH J 1 0.026

FRENCH JK 1 0.026

FRESU P 1 0.026

FREUND J 1 0.026

FREY H 1 0.026

FRIBOURG M 1 0.026

FRIED L 1 0.026

FRIEDE T 1 0.026

FRIEDERICH-PERSSON M 1 0.026

FRIEDLANDER G 1 0.026

FRIEDMAN GC 1 0.026

FRIEDMAN RA 1 0.026

FRIEDRICHS WE 1 0.026

FRIIS UG 1 0.026

FRIMAT M 1 0.026

FRIMODT-MOLLER M 1 0.026

FRIZZI R 1 0.026

FROISSART M 1 0.026

FROMM M 1 0.026

FROSSI B 1 0.026

FROVA L 1 0.026

FU BT 1 0.026

FU C 1 0.026

FU FH 1 0.026

FU GS 1 0.026

FU HX 1 0.026

FU HY 1 0.026

FU JF 1 0.026

FU JJ 1 0.026

FU JX 1 0.026

FU M 1 0.026

FU MX 1 0.026

FU Q 1 0.026

FU SS 1 0.026

FU TT 1 0.026

FU XG 1 0.026

FU XH 1 0.026

FU XQ 1 0.026

FU YQ 1 0.026

FUCHS A 1 0.026

FUCHS L 1 0.026

FUENTES-CALVO I 1 0.026

FUFAA G 1 0.026

FUFAA GD 1 0.026

FUIANO G 1 0.026

FUJIGAKI Y 1 0.026

FUJII S 1 0.026

FUJIKAWA A 1 0.026

FUJIKURA T 1 0.026

FUJIMIYA M 1 0.026

FUJIMORI A 1 0.026

FUJIMOTO K 1 0.026

FUJIMOTO S 1 0.026

FUJIMURA T 1 0.026

FUJINO H 1 0.026

FUJISAKI K 1 0.026

FUJISAWA G 1 0.026

FUJISAWA M 1 0.026

FUJISHIRO M 1 0.026

FUJITA M 1 0.026

FUJITA N 1 0.026

FUJITA Y 1 0.026

FUJIWARA K 1 0.026

FUKAHORI H 1 0.026

FUKASAWA H 1 0.026

FUKATSU A 1 0.026

FUKUDA H 1 0.026

FUKUDA M 1 0.026

FUKUDA N 1 0.026

FUKUHARA H 1 0.026

FUKUI K 1 0.026

FUKUI M 1 0.026

FUKUMA S 1 0.026

FUKUMOTO M 1 0.026

FUKUMOTO Y 1 0.026

FUKUNAGA M 1 0.026

FUKUOKA N 1 0.026

FUKUSHIMA K 1 0.026

FUKUSHIMA M 1 0.026

FUKUTA K 1 0.026

FULCHER GR 1 0.026

FULLADOSA X 1 0.026

FULLER KNZ 1 0.026

FULTON D 1 0.026

FUNAHASHI T 1 0.026

FUNAOKA M 1 0.026

FUNCK M 1 0.026

FUNDER JW 1 0.026

FUNG CLS 1 0.026

FUNG E 1 0.026

FUNG SH 1 0.026

FUNK JM 1 0.026

FUNK RHW 1 0.026

FURCI L 1 0.026

FURIC-CUNKO V 1 0.026

FURINI G 1 0.026

FURLANELLO F 1 0.026

FURNESS PN 1 0.026

FURTH EE 1 0.026

FURUKAWA M 1 0.026

FURUTA T 1 0.026

FUSHIMA T 1 0.026

FUTRAKUL N 1 0.026

FUTRAKUL P 1 0.026

FYFE B 1 0.026

GABAZZA EC 1 0.026

GABDRAKHMANOVA L 1 0.026

GABER L 1 0.026

GABRIELYAN O 1 0.026

GADEGBEKU CA 1 0.026

GAEDEKE J 1 0.026

GAESTEL M 1 0.026

GAFFNEY A 1 0.026

GAFOR AHA 1 0.026

GAGLIARDI I 1 0.026

GAGNON L 1 0.026

GAIKWAD N 1 0.026

GAILLARD CAJM 1 0.026

GAILLARD D 1 0.026

GAIVIN R 1 0.026

GAJJALA PR 1 0.026

GALCERAN JM 1 0.026

GALENKO-YAROSHEVSKY PA 1 0.026

GALIC S 1 0.026

GALINDO MJ 1 0.026

GALINDO RJ 1 0.026

GALISTEO-ALMEDA L 1 0.026

GALLAGHER ST 1 0.026

GALLE PR 1 0.026

GALLEGO B 1 0.026

GALLEGOS-CORONA MA 1 0.026

GALLIENI M 1 0.026

GALLO J 1 0.026

GALLO S 1 0.026

GALLONE A 1 0.026

GALVAN V 1 0.026

GAMBARA V 1 0.026

GAMBOA F 1 0.026

GAMELLA-POZUELO L 1 0.026

GAMMAL A 1 0.026

GAN H 1 0.026

GAN R 1 0.026

GAN XD 1 0.026

GANAPATHY V 1 0.026

GANDHIRAJAN RK 1 0.026

GANDHOK H 1 0.026

GANG W 1 0.026

GANG XK 1 0.026

GANGULY A 1 0.026

GANIDAGLI B 1 0.026

GANS ROB 1 0.026

GANTNER F 1 0.026

GAO FF 1 0.026

GAO FL 1 0.026

GAO G 1 0.026

GAO HC 1 0.026

GAO HJ 1 0.026

GAO HX 1 0.026

GAO HY 1 0.026

GAO J 1 0.026

GAO JJ 1 0.026

GAO JQ 1 0.026

GAO LP 1 0.026

GAO M 1 0.026

GAO MY 1 0.026

GAO PJ 1 0.026

GAO PP 1 0.026

GAO RF 1 0.026

GAO T 1 0.026

GAO WJ 1 0.026

GAO WK 1 0.026

GAO WW 1 0.026

GAO XD 1 0.026

GAO XH 1 0.026

GAO YX 1 0.026

GAO ZQ 1 0.026

GAORA PO 1 0.026

GARATE-CARRILLO A 1 0.026

GARBARINO F 1 0.026

GARBAY S 1 0.026

GARBER SL 1 0.026

GARBIN HI 1 0.026

GARCIA C 1 0.026

GARCIA G 1 0.026

GARCIA I 1 0.026

GARCIA L 1 0.026

GARCIA NH 1 0.026

GARCIA RA 1 0.026

GARCIA XA 1 0.026

GARCIA YM 1 0.026

GARCIA-BUITRAGO M 1 0.026

GARCIA-CALVO M 1 0.026

GARCIA-CARRO C 1 0.026

GARCIA-CONTRERAS C 1 0.026

GARCIA-COVARRUBIAS A 1 0.026

GARCIA-COVARRUBIAS L 1 0.026

GARCIA-FERNANDEZ N 1 0.026

GARCIA-HUETE L 1 0.026

GARCIA-LOPEZ E 1 0.026

GARCIA-ORTIZ L 1 0.026

GARCIA-PASTOR C 1 0.026

GARCIA-ROIG M 1 0.026

GARDLIK R 1 0.026

GARDNER HA 1 0.026

GARG D 1 0.026

GAROFALO C 1 0.026

GAROVIC VD 1 0.026

GARRIGUES L 1 0.026

GARROUSTE C 1 0.026

GARSEN M 1 0.026

GARTNER V 1 0.026

GARTSHTEYN Y 1 0.026

GARZA C 1 0.026

GARZON F 1 0.026

GASBARRO G 1 0.026

GASIOR M 1 0.026

GASPAR G 1 0.026

GASPARI S 1 0.026

GASPER WJ 1 0.026

GASPERT A 1 0.026

GASTALDELLO A 1 0.026

GATAGONOVA TM 1 0.026

GATTO M 1 0.026

GATTONE V 1 0.026

GAUER S 1 0.026

GAUGUIER D 1 0.026

GAUTAM D 1 0.026

GAUTHIER C 1 0.026

GAVA E 1 0.026

GAXATTE C 1 0.026

GAYNOR JJ 1 0.026

GAYSINA D 1 0.026

GAZIANO JM 1 0.026

GE J 1 0.026

GE MY 1 0.026

GE Q 1 0.026

GE RR 1 0.026

GE RW 1 0.026

GE SW 1 0.026

GE TW 1 0.026

GE YC 1 0.026

GE YN 1 0.026

GEARA A 1 0.026

GEBERTH S 1 0.026

GEDIKBASI A 1 0.026

GEE I 1 0.026

GEENEN DL 1 0.026

GEIGER H 1 0.026

GEISSLINGER G 1 0.026

GELAPE CL 1 0.026

GELLIBERT F 1 0.026

GEMMATI D 1 0.026

GENDERINI A 1 0.026

GENG JA 1 0.026

GENG JG 1 0.026

GENG JN 1 0.026

GENG LH 1 0.026

GENSCHEL C 1 0.026

GENTA SB 1 0.026

GENTRY M 1 0.026

GENUNG NE 1 0.026

GEORGIUS P 1 0.026

GERACI C 1 0.026

GERALDINO-PARDILLA L 1 0.026

GERARDUZZI C 1 0.026

GERASIMOVA M 1 0.026

GERL M 1 0.026

GERON M 1 0.026

GEROSA G 1 0.026

GERRITS T 1 0.026

GERTH J 1 0.026

GEWIN LS 1 0.026

GEZGINCI-OKTAYOGLU S 1 0.026

GHADIEH HE 1 0.026

GHAI V 1 0.026

GHE C 1 0.026

GHERGHICEANU M 1 0.026

GHIGGERI G 1 0.026

GHIGGERI GM 1 0.026

GHIRARDELLO A 1 0.026

GHOLAMINEJAD A 1 0.026

GHONEIM MA 1 0.026

GHORBANI A 1 0.026

GHOSH-CHOUDHURY G 1 0.026

GHOSSEIN J 1 0.026

GHULE AE 1 0.026

GIACCARI A 1 0.026

GIANI MGP 1 0.026

GIANNOPOULOU M 1 0.026

GIARDINO LA 1 0.026

GIBBS P 1 0.026

GIBSON KJ 1 0.026

GIEBEL J 1 0.026

GIERMAKOWSKA W 1 0.026

GIFFORD CC 1 0.026

GIGANTE M 1 0.026

GIGLIOTTI G 1 0.026

GIL CL 1 0.026

GIL-BERNABE P 1 0.026

GIL-VERNET S 1 0.026

GILARDI F 1 0.026

GILBERT JD 1 0.026

GILBEY SG 1 0.026

GILES RH 1 0.026

GILET M 1 0.026

GILI M 1 0.026

GILL AJ 1 0.026

GILLESPIE BW 1 0.026

GILLET M 1 0.026

GILLIGAN S 1 0.026

GILLIS K 1 0.026

GIMSON AES 1 0.026

GIOCO F 1 0.026

GIOLLO A 1 0.026

GIORDA CB 1 0.026

GIORDANO L 1 0.026

GIORGIONE V 1 0.026

GIPSON DS 1 0.026

GIRALT-LOPEZ A 1 0.026

GIRARDI JM 1 0.026

GIRARDIN SE 1 0.026

GIRERD N 1 0.026

GIRERD S 1 0.026

GIRIBABU N 1 0.026

GIRICZ Z 1 0.026

GIRON-MICHEL J 1 0.026

GIROT R 1 0.026

GIUNTI S 1 0.026

GIUS DR 1 0.026

GIVERTZ MM 1 0.026

GLASER S 1 0.026

GLEADLE JM 1 0.026

GLEASON RE 1 0.026

GLIELMI CB 1 0.026

GLOBKE B 1 0.026

GLORIA MA 1 0.026

GLORIEUX G 1 0.026

GLUBA-BRZOZKA A 1 0.026

GLUSHAKOVA OY 1 0.026

GLYBOCHKO PV 1 0.026

GLYKOFRIDI S 1 0.026

GLYN-JONES S 1 0.026

GNANASEKARAN G 1 0.026

GNEMMI V 1 0.026

GO AS 1 0.026

GO G 1 0.026

GOBEL H 1 0.026

GODET J 1 0.026

GODLEWSKI G 1 0.026

GODOY P 1 0.026

GODSON-TREACY M 1 0.026

GOEL M 1 0.026

GOICOECHEA M 1 0.026

GOJA S 1 0.026

GOK M 1 0.026

GOKTAY Y 1 0.026

GOKTURK H 1 0.026

GOLABI P 1 0.026

GOLDENBERG NM 1 0.026

GOLDSCHMEDING RG 1 0.026

GOLTSMAN I 1 0.026

GOMA-I-FREIXANET M 1 0.026

GOMES RM 1 0.026

GOMES-NETO AW 1 0.026

GOMEZ D 1 0.026

GOMEZ IG 1 0.026

GOMEZ LA 1 0.026

GOMEZ MF 1 0.026

GOMEZ X 1 0.026

GOMEZ-CAMARERO J 1 0.026

GOMEZ-MARCOS MA 1 0.026

GOMEZ-SANCHEZ CE 1 0.026

GOMEZ-SANCHEZ CM 1 0.026

GOMEZ-SANCHEZ EP 1 0.026

GONCALVES ARR 1 0.026

GONCALVES N 1 0.026

GONCALVES-DIAS C 1 0.026

GONDER JR 1 0.026

GONG A 1 0.026

GONG CX 1 0.026

GONG D 1 0.026

GONG DM 1 0.026

GONG DY 1 0.026

GONG EY 1 0.026

GONG J 1 0.026

GONG JH 1 0.026

GONG KZ 1 0.026

GONG M 1 0.026

GONG MJ 1 0.026

GONG MM 1 0.026

GONG Q 1 0.026

GONG QY 1 0.026

GONG WJ 1 0.026

GONG X 1 0.026

GONG XH 1 0.026

GONG YH 1 0.026

GONG YX 1 0.026

GONG Z 1 0.026

GONG ZJ 1 0.026

GONZALES M 1 0.026

GONZALEZ A 1 0.026

GONZALEZ JM 1 0.026

GONZALEZ MT 1 0.026

GONZALEZ O 1 0.026

GONZALEZ-BULNES A 1 0.026

GONZALEZ-FERNANDEZ E 1 0.026

GONZALEZ-GARCIA J 1 0.026

GONZALEZ-GUERRERO C 1 0.026

GONZALEZ-LAFUENTE L 1 0.026

GONZALEZ-MOLINA M 1 0.026

GONZALEZ-NUNEZ M 1 0.026

GONZALEZ-RIVERA T 1 0.026

GONZALEZ-SEGURA C 1 0.026

GONZALEZ-VERGARA A 1 0.026

GONZALEZ-VILLALOBOS RA 1 0.026

GOOD DM 1 0.026

GOODERHAM NJ 1 0.026

GOOZ M 1 0.026

GOPALAKRISHNAN M 1 0.026

GOPALAKRISHNAN MM 1 0.026

GOPALAKRISHNAN S 1 0.026

GOPPELT-STRUEBE M 1 0.026

GORACKE N 1 0.026

GORDAT M 1 0.026

GORDIN D 1 0.026

GORDISH-DRESSMAN H 1 0.026

GORDON BAJ 1 0.026

GORDON DA 1 0.026

GORE JC 1 0.026

GORE-HYER E 1 0.026

GORES PF 1 0.026

GORGE AF 1 0.026

GORIN MA 1 0.026

GORIN YC 1 0.026

GORLACH A 1 0.026

GORRIZ JL 1 0.026

GOSWAMI C 1 0.026

GOTH L 1 0.026

GOTO E 1 0.026

GOTO K 1 0.026

GOTO R 1 0.026

GOTO S 1 0.026

GOTTWALD-HOSTALEK U 1 0.026

GOU F 1 0.026

GOU SJ 1 0.026

GOUDEFROYE G 1 0.026

GOULD FK 1 0.026

GOULD KF 1 0.026

GOUMENOS DS 1 0.026

GOURDY P 1 0.026

GOW R 1 0.026

GOYA C 1 0.026

GOYAL P 1 0.026

GOYAL S 1 0.026

GOZAL E 1 0.026

GRABIAS BM 1 0.026

GRACHEV SV 1 0.026

GRAHAMMER F 1 0.026

GRAHOVAC G 1 0.026

GRAMMATIKOS G 1 0.026

GRANATA C 1 0.026

GRANATA F 1 0.026

GRANBERG KL 1 0.026

GRANCIC P 1 0.026

GRANDALIANO G 1 0.026

GRANT R 1 0.026

GRANTER SR 1 0.026

GRANTHAM CE 1 0.026

GRASSI C 1 0.026

GRASSIN-DELYLE S 1 0.026

GRATIGNY M 1 0.026

GRAUPE M 1 0.026

GRAVELINE A 1 0.026

GRAVESEN E 1 0.026

GRAY CE 1 0.026

GRAY J 1 0.026

GRAY S 1 0.026

GRAZIOTTO R 1 0.026

GREEN J 1 0.026

GREENHILL NS 1 0.026

GREENIDGE AR 1 0.026

GREENWOOD JP 1 0.026

GREGORIO BM 1 0.026

GREGORIO EP 1 0.026

GREGORY JW 1 0.026

GREMMELS H 1 0.026

GRENET D 1 0.026

GRENIER N 1 0.026

GRENON MS 1 0.026

GRIENDLING K 1 0.026

GRIEVE DJ 1 0.026

GRIFFIN JP 1 0.026

GRIFFIN TP 1 0.026

GRIGGS D 1 0.026

GRIGGS K 1 0.026

GRILL JF 1 0.026

GRILLARI J 1 0.026

GRILLARI-VOGLAUER R 1 0.026

GRIM C 1 0.026

GRISK O 1 0.026

GROBMAYR R 1 0.026

GRODIN JL 1 0.026

GROENENDYK J 1 0.026

GROENNING BA 1 0.026

GROMA V 1 0.026

GRONA HJ 1 0.026

GRONE EF 1 0.026

GROOP L 1 0.026

GROPPOLI T 1 0.026

GROSCHNER LN 1 0.026

GROSS ML 1 0.026

GROSSMAN LI 1 0.026

GROSU I 1 0.026

GROSU L 1 0.026

GROTEGUT CA 1 0.026

GROTENDORST G 1 0.026

GROUIX B 1 0.026

GROVER S 1 0.026

GROVER SA 1 0.026

GRUBLER MR 1 0.026

GRUENWALD A 1 0.026

GRUNG P 1 0.026

GRYGIELKO ET 1 0.026

GU HC 1 0.026

GU HW 1 0.026

GU J 1 0.026

GU JN 1 0.026

GU L 1 0.026

GU LF 1 0.026

GU LH 1 0.026

GU LJ 1 0.026

GU M 1 0.026

GU SS 1 0.026

GU TW 1 0.026

GU XK 1 0.026

GU XM 1 0.026

GU XX 1 0.026

GU ZH 1 0.026

GU ZY 1 0.026

GUAN N 1 0.026

GUAN QN 1 0.026

GUAN TH 1 0.026

GUAN TJ 1 0.026

GUAN WM 1 0.026

GUAN XL 1 0.026

GUAN XX 1 0.026

GUAN Y 1 0.026

GUAN YH 1 0.026

GUAN YT 1 0.026

GUARDIOLA JM 1 0.026

GUARNER V 1 0.026

GUARNERI M 1 0.026

GUARNERIO S 1 0.026

GUARRERA J 1 0.026

GUASTI D 1 0.026

GUAY A 1 0.026

GUBLER MC 1 0.026

GUDERIAN F 1 0.026

GUELINCKX I 1 0.026

GUERCI B 1 0.026

GUERRA G 1 0.026

GUERROT D 1 0.026

GUERZONI E 1 0.026

GUEVARA-GONZALEZ RG 1 0.026

GUGGINO WB 1 0.026

GUI DK 1 0.026

GUI T 1 0.026

GUIDA P 1 0.026

GUIDUCCI C 1 0.026

GUILLEMAIN R 1 0.026

GUILLOU H 1 0.026

GUIMARAES CSO 1 0.026

GUIONAUD S 1 0.026

GUIRY P 1 0.026

GUITERAS R 1 0.026

GULLAPUDI L 1 0.026

GULLBERG D 1 0.026

GULLESTAD L 1 0.026

GULMEZ N 1 0.026

GULMI FA 1 0.026

GULSIN GS 1 0.026

GULTEKIN ND 1 0.026

GUNAYDIN ZY 1 0.026

GUNEY I 1 0.026

GUNGOR O 1 0.026

GUNN NT 1 0.026

GUNNARSSON A 1 0.026

GUNNING WT 1 0.026

GUNSON BK 1 0.026

GUNZEL D 1 0.026

GUO C 1 0.026

GUO CH 1 0.026

GUO FB 1 0.026

GUO JC 1 0.026

GUO JP 1 0.026

GUO KW 1 0.026

GUO LL 1 0.026

GUO LM 1 0.026

GUO M 1 0.026

GUO MH 1 0.026

GUO MJ 1 0.026

GUO MQ 1 0.026

GUO MZ 1 0.026

GUO P 1 0.026

GUO Q 1 0.026

GUO QY 1 0.026

GUO R 1 0.026

GUO S 1 0.026

GUO SZ 1 0.026

GUO TK 1 0.026

GUO WG 1 0.026

GUO WK 1 0.026

GUO X 1 0.026

GUO XF 1 0.026

GUO XH 1 0.026

GUO XN 1 0.026

GUO YF 1 0.026

GUO YH 1 0.026

GUO YL 1 0.026

GUO ZJ 1 0.026

GUO ZS 1 0.026

GUPTA J 1 0.026

GUPTA N 1 0.026

GUPTA R 1 0.026

GURBUZ F 1 0.026

GURUNG P 1 0.026

GUSBETH-TATOMIR P 1 0.026

GUSCETTI F 1 0.026

GUSMAO JB 1 0.026

GUTBERLET M 1 0.026

GUTHRIE K 1 0.026

GUTIERREZ C 1 0.026

GUTIERREZ RMP 1 0.026

GUTIERREZ-GARCIA ML 1 0.026

GUZEL A 1 0.026

GUZEL FB 1 0.026

GUZMAN J 1 0.026

GUZMAN-GRENFELL AM 1 0.026

HA HK 1 0.026

HA SJ 1 0.026

HA SK 1 0.026

HA SO 1 0.026

HA SW 1 0.026

HAAS JS 1 0.026

HAAS M 1 0.026

HABBOUT A 1 0.026

HABEB AM 1 0.026

HABIB AM 1 0.026

HABIB M 1 0.026

HABUCHI T 1 0.026

HACENI R 1 0.026

HACK B 1 0.026

HACKAM DG 1 0.026

HADCOCK JR 1 0.026

HADDAD G 1 0.026

HADDAD PS 1 0.026

HADDEN MJ 1 0.026

HADJIYANNAKIS S 1 0.026

HAFEZ HM 1 0.026

HAFLIOADOTTIR S 1 0.026

HAGA H 1 0.026

HAGAMAN J 1 0.026

HAGEGE I 1 0.026

HAGG PM 1 0.026

HAGG PO 1 0.026

HAGHJO AG 1 0.026

HAGMANN H 1 0.026

HAGSTROM H 1 0.026

HAHM K 1 0.026

HAHN WH 1 0.026

HAIMING X 1 0.026

HAJAGE D 1 0.026

HAKROUSH S 1 0.026

HALE LJ 1 0.026

HALIMI S 1 0.026

HALL G 1 0.026

HALL RG 1 0.026

HALLER ST 1 0.026

HALLORAN PF 1 0.026

HALPERN Z 1 0.026

HALPRYN B 1 0.026

HALUZIK M 1 0.026

HAMADA K 1 0.026

HAMADA T 1 0.026

HAMAGUCHI A 1 0.026

HAMAGUCHI K 1 0.026

HAMAGUCHI M 1 0.026

HAMANO H 1 0.026

HAMANO T 1 0.026

HAMBLIN MR 1 0.026

HAMDY A 1 0.026

HAMET P 1 0.026

HAMIDI C 1 0.026

HAMIDIAN A 1 0.026

HAMM C 1 0.026

HAMMER MH 1 0.026

HAMMES M 1 0.026

HAMMING I 1 0.026

HAMMOND E 1 0.026

HAMMOUD SH 1 0.026

HAMOUD S 1 0.026

HAMZIC LF 1 0.026

HAN DH 1 0.026

HAN DN 1 0.026

HAN DS 1 0.026

HAN EH 1 0.026

HAN HJ 1 0.026

HAN HL 1 0.026

HAN JB 1 0.026

HAN JK 1 0.026

HAN JR 1 0.026

HAN P 1 0.026

HAN PF 1 0.026

HAN Q 1 0.026

HAN QX 1 0.026

HAN S 1 0.026

HAN SJ 1 0.026

HAN SP 1 0.026

HAN SS 1 0.026

HAN WH 1 0.026

HAN WK 1 0.026

HAN WQ 1 0.026

HAN X 1 0.026

HAN XB 1 0.026

HAN XC 1 0.026

HAN XR 1 0.026

HAN YL 1 0.026

HAN YS 1 0.026

HAN Z 1 0.026

HANAFUSA T 1 0.026

HANAOKA K 1 0.026

HANAUER G 1 0.026

HANDISURYA A 1 0.026

HANKE W 1 0.026

HANNA C 1 0.026

HANNEDOUCHE T 1 0.026

HANSELL P 1 0.026

HANSEN MH 1 0.026

HANSEN MK 1 0.026

HANSEN PBL 1 0.026

HANSEN TK 1 0.026

HANSMANN C 1 0.026

HANSON L 1 0.026

HANSSEN KF 1 0.026

HANSSEN L 1 0.026

HANUT A 1 0.026

HAO C 1 0.026

HAO CM 1 0.026

HAO JB 1 0.026

HAO YM 1 0.026

HAO YR 1 0.026

HAO ZM 1 0.026

HARA M 1 0.026

HARADA A 1 0.026

HARADA T 1 0.026

HARAGUCHI K 1 0.026

HARDING JW 1 0.026

HARDUNG L 1 0.026

HARIBABU B 1 0.026

HARING R 1 0.026

HARJUTSALO V 1 0.026

HARMON B 1 0.026

HARPER GM 1 0.026

HARRISON AC 1 0.026

HARRISON JC 1 0.026

HARRISON L 1 0.026

HARRISON S 1 0.026

HARSKAMP LR 1 0.026

HARTLEIB-GESCHWINDNER J 1 0.026

HARTMAN RE 1 0.026

HARTMANN G 1 0.026

HARTMANN M 1 0.026

HARTMANN RW 1 0.026

HARTOG JWL 1 0.026

HARTONO S 1 0.026

HARTONO SP 1 0.026

HARTUNG D 1 0.026

HARUMI T 1 0.026

HARUNA Y 1 0.026

HARVIE BM 1 0.026

HASAN GM 1 0.026

HASAN IH 1 0.026

HASAN MN 1 0.026

HASAN R 1 0.026

HASAN SS 1 0.026

HASCHKE-BECHER E 1 0.026

HASE M 1 0.026

HASEGAWA G 1 0.026

HASEGAWA H 1 0.026

HASEGAWA J 1 0.026

HASEGAWA K 1 0.026

HASEGAWA S 1 0.026

HASEGAWA Y 1 0.026

HASHEMI M 1 0.026

HASHIMOTO T 1 0.026

HASHIMOTO Y 1 0.026

HASNAOUI M 1 0.026

HASSAN A 1 0.026

HASSAN K 1 0.026

HASSAN MI 1 0.026

HASSANEIN H 1 0.026

HASSLACHER C 1 0.026

HATAI A 1 0.026

HATAKEYAMA E 1 0.026

HATAKEYAMA K 1 0.026

HATAKEYAMA Y 1 0.026

HATANAKA M 1 0.026

HATEM-VAQUERO M 1 0.026

HATHAWAY CK 1 0.026

HATTAPOGLU S 1 0.026

HATTERSLEY AT 1 0.026

HATTORI R 1 0.026

HAUG M 1 0.026

HAUGEN EN 1 0.026

HAUKE T 1 0.026

HAUSER C 1 0.026

HAUSER IA 1 0.026

HAUSER P 1 0.026

HAVASI A 1 0.026

HAVILL N 1 0.026

HAWES L 1 0.026

HAWFIELD AT 1 0.026

HAWKE DH 1 0.026

HAWKES D 1 0.026

HAWKINS JJ 1 0.026

HAWKINS UA 1 0.026

HAYAKAWA A 1 0.026

HAYAKAWA M 1 0.026

HAYAKAWA S 1 0.026

HAYASHI M 1 0.026

HAYASHI S 1 0.026

HAYASHI Y 1 0.026

HAYASHIDA A 1 0.026

HAYASHINO Y 1 0.026

HAYASHIZAKI-SOMEYA Y 1 0.026

HAYEM C 1 0.026

HAYLOR J 1 0.026

HAYNES JS 1 0.026

HAYNES R 1 0.026

HAYWARD A 1 0.026

HAZAMA M 1 0.026

HAZEN-MARTIN D 1 0.026

HE AL 1 0.026

HE CJ 1 0.026

HE CZ 1 0.026

HE F 1 0.026

HE H 1 0.026

HE HJ 1 0.026

HE HX 1 0.026

HE JT 1 0.026

HE JX 1 0.026

HE LQ 1 0.026

HE P 1 0.026

HE SR 1 0.026

HE SY 1 0.026

HE TL 1 0.026

HE TW 1 0.026

HE WH 1 0.026

HE WM 1 0.026

HE XC 1 0.026

HE XJ 1 0.026

HE YC 1 0.026

HE YJ 1 0.026

HE YQ 1 0.026

HE YX 1 0.026

HE YZ 1 0.026

HE Z 1 0.026

HE ZB 1 0.026

HE ZG 1 0.026

HE ZW 1 0.026

HEAD CA 1 0.026

HEALY H 1 0.026

HEARN T 1 0.026

HEASMAN S 1 0.026

HEATHCOTE J 1 0.026

HEBERT LA 1 0.026

HECOX D 1 0.026

HEDAYAT FA 1 0.026

HEEGER PS 1 0.026

HEENAN HF 1 0.026

HEERINGA P 1 0.026

HEFFEMAN SJ 1 0.026

HEGELE RA 1 0.026

HEGGERMONT WA 1 0.026

HEHER E 1 0.026

HEIDEMPERGHER M 1 0.026

HEIDET L 1 0.026

HEIFETS M 1 0.026

HEIJERMAN HGM 1 0.026

HEIJS B 1 0.026

HEIKKILA O 1 0.026

HEINE RP 1 0.026

HEINER-FOKKEMA MR 1 0.026

HEININGER D 1 0.026

HEINONEN I 1 0.026

HEITZ F 1 0.026

HEITZMANN D 1 0.026

HELENIAK Z 1 0.026

HELIN H 1 0.026

HELJIC M 1 0.026

HELLWIG A 1 0.026

HELLWIG M 1 0.026

HELMCHEN U 1 0.026

HELMOL FR 1 0.026

HELO S 1 0.026

HEMMELDER MH 1 0.026

HEMMELGARN BR 1 0.026

HENDERSON BC 1 0.026

HENDERSON J 1 0.026

HENDERSON NC 1 0.026

HENG YY 1 0.026

HENGER A 1 0.026

HENGSTENBERG C 1 0.026

HENJAKOVIC M 1 0.026

HENKE N 1 0.026

HENLEY N 1 0.026

HENNEDIGE T 1 0.026

HENNEQUIN M 1 0.026

HENNESSY A 1 0.026

HENNRIKUS MT 1 0.026

HENRY ML 1 0.026

HENSEY C 1 0.026

HENSHAW DJE 1 0.026

HENZE A 1 0.026

HEO JH 1 0.026

HEO JY 1 0.026

HERBERT KE 1 0.026

HERDER C 1 0.026

HERICH-TERHURNE D 1 0.026

HERKNER H 1 0.026

HERMAN RJ 1 0.026

HERMANN M 1 0.026

HERMANS C 1 0.026

HERMANS R 1 0.026

HERNANDEZ A 1 0.026

HERNANDEZ D 1 0.026

HERNANDEZ FJL 1 0.026

HERNANDEZ REC 1 0.026

HERNANDEZ-GUERRA M 1 0.026

HERNANDEZ-HERNANDEZ ME 1 0.026

HERNANDEZ-REYES P 1 0.026

HERNANDEZ-RIVER JC 1 0.026

HERRERA M 1 0.026

HERRERA R 1 0.026

HERRERA-ABARCA JE 1 0.026

HERRERA-PEREZ Z 1 0.026

HERRMANN SM 1 0.026

HERTZOG P 1 0.026

HERZOG CA 1 0.026

HESP AC 1 0.026

HEUDES D 1 0.026

HEUSER M 1 0.026

HEWISON M 1 0.026

HEWITSON T 1 0.026

HEWITT R 1 0.026

HEWITT SM 1 0.026

HEYMAN SN 1 0.026

HIAI H 1 0.026

HIBI C 1 0.026

HICKEY F 1 0.026

HICKS JJ 1 0.026

HIDA K 1 0.026

HIETALA K 1 0.026

HIGA EMS 1 0.026

HIGASHI AY 1 0.026

HIGASHI K 1 0.026

HIGASHI Y 1 0.026

HIGASHIJIMA Y 1 0.026

HIGASHIMOTO Y 1 0.026

HIGASHIUESATO Y 1 0.026

HIGGINS C 1 0.026

HIGGINS D 1 0.026

HIGGINS DF 1 0.026

HIGUCHI C 1 0.026

HIJONA E 1 0.026

HILE KL 1 0.026

HILHORST M 1 0.026

HILL A 1 0.026

HILL DJ 1 0.026

HILL G 1 0.026

HILL GS 1 0.026

HILL J 1 0.026

HILL MD 1 0.026

HILL N 1 0.026

HILL P 1 0.026

HILLEBRAND DJ 1 0.026

HILLER S 1 0.026

HIMMELFARB J 1 0.026

HIMMERKUS N 1 0.026

HINDERLITER AL 1 0.026

HINDRICKS G 1 0.026

HINES WH 1 0.026

HINMAN EG 1 0.026

HINOJOSA-KIRSCHENBAUM F 1 0.026

HIRAGUSHI K 1 0.026

HIRAI H 1 0.026

HIRAISHI K 1 0.026

HIRAKAWA N 1 0.026

HIRAMATSU-ITO M 1 0.026

HIRANO I 1 0.026

HIRANO K 1 0.026

HIRAOKA-YAMAMOTO J 1 0.026

HIRATA S 1 0.026

HIRATA T 1 0.026

HIRATSUKA K 1 0.026

HIROMURA K 1 0.026

HIROSE J 1 0.026

HIROSE T 1 0.026

HIROTSUKA M 1 0.026

HIRSCH E 1 0.026

HIRSCHHORN JN 1 0.026

HISAMICHI M 1 0.026

HISHIDA A 1 0.026

HISHIKI M 1 0.026

HITSUMOTO T 1 0.026

HO F 1 0.026

HO J 1 0.026

HO LC 1 0.026

HO LY 1 0.026

HO WT 1 0.026

HOAGLAND KM 1 0.026

HOARE M 1 0.026

HOCKER B 1 0.026

HODI Z 1 0.026

HODNETT PA 1 0.026

HODOSY J 1 0.026

HODSON J 1 0.026

HOERLYCK A 1 0.026

HOFER S 1 0.026

HOFFMAN BB 1 0.026

HOFFMAN GS 1 0.026

HOFFMAN J 1 0.026

HOFFMAN RM 1 0.026

HOFFMANN B 1 0.026

HOFFMANN SC 1 0.026

HOFMAN-BANG J 1 0.026

HOFSTETTER L 1 0.026

HOFT A 1 0.026

HOGL S 1 0.026

HOGUE J 1 0.026

HOHENADEL D 1 0.026

HOHL M 1 0.026

HOHMANN M 1 0.026

HOHNE M 1 0.026

HOJNA S 1 0.026

HOKE U 1 0.026

HOLBROOK JT 1 0.026

HOLDERIED A 1 0.026

HOLDSWORTH SR 1 0.026

HOLKAR A 1 0.026

HOLL RW 1 0.026

HOLLENBERG NK 1 0.026

HOLLER S 1 0.026

HOLMAN ER 1 0.026

HOLMES E 1 0.026

HOLT SG 1 0.026

HOLTZ M 1 0.026

HOLZMANN Y 1 0.026

HOMBREBUENO JR 1 0.026

HOMER BL 1 0.026

HOMMA K 1 0.026

HOMMA T 1 0.026

HOMMA Y 1 0.026

HOMMEL KA 1 0.026

HOMMOS MS 1 0.026

HONEYBOURNE D 1 0.026

HONG BA 1 0.026

HONG BF 1 0.026

HONG GL 1 0.026

HONG MH 1 0.026

HONG MQ 1 0.026

HONG SM 1 0.026

HONG X 1 0.026

HONG YJ 1 0.026

HONG Z 1 0.026

HONG ZZ 1 0.026

HONJO H 1 0.026

HONJO J 1 0.026

HONJO S 1 0.026

HONKANEN T 1 0.026

HONMA T 1 0.026

HONORATO VH 1 0.026

HONORE SM 1 0.026

HONOS G 1 0.026

HOOD KK 1 0.026

HOOFNAGLE AN 1 0.026

HOOKHAM MB 1 0.026

HOORN EJ 1 0.026

HOPFER S 1 0.026

HOPFER U 1 0.026

HOPKINS LJ 1 0.026

HOPPE AK 1 0.026

HOPPE B 1 0.026

HOPPENER JW 1 0.026

HORAN GS 1 0.026

HORI S 1 0.026

HORI Y 1 0.026

HORIE S 1 0.026

HORIKE H 1 0.026

HORINOUCHI Y 1 0.026

HORN A 1 0.026

HORN T 1 0.026

HOSHI M 1 0.026

HOSONO T 1 0.026

HOSOYA M 1 0.026

HOSOYA T 1 0.026

HOSSAIN MI 1 0.026

HOSSEINI SM 1 0.026

HOTTA O 1 0.026

HOU B 1 0.026

HOU CJY 1 0.026

HOU FL 1 0.026

HOU FY 1 0.026

HOU JH 1 0.026

HOU LM 1 0.026

HOU MC 1 0.026

HOU XJ 1 0.026

HOU XL 1 0.026

HOU Y 1 0.026

HOU YJ 1 0.026

HOUGH TA 1 0.026

HOULIHAN CA 1 0.026

HOUNG JY 1 0.026

HOUSER SR 1 0.026

HOVIND P 1 0.026

HOWARD V 1 0.026

HOWARTH FC 1 0.026

HOWELL LA 1 0.026

HOWELL LH 1 0.026

HOWENSTINE M 1 0.026

HOWIE AJ 1 0.026

HOWLETT JG 1 0.026

HOWLIN J 1 0.026

HOWSAM M 1 0.026

HOYER P 1 0.026

HRENAK J 1 0.026

HSIA CCW 1 0.026

HSIAO CC 1 0.026

HSIAO HD 1 0.026

HSIAO PJ 1 0.026

HSIEH CC 1 0.026

HSIEH LTH 1 0.026

HSIEH SC 1 0.026

HSIEH SL 1 0.026

HSIEH YP 1 0.026

HSIEH YS 1 0.026

HSING CH 1 0.026

HSU C 1 0.026

HSU CY 1 0.026

HSU FY 1 0.026

HSU HT 1 0.026

HSU JD 1 0.026

HSU MS 1 0.026

HSU SC 1 0.026

HSU T 1 0.026

HSU YJ 1 0.026

HSUEH WA 1 0.026

HSUEH YT 1 0.026

HU A 1 0.026

HU B 1 0.026

HU DD 1 0.026

HU FC 1 0.026

HU G 1 0.026

HU GC 1 0.026

HU HB 1 0.026

HU HC 1 0.026

HU HL 1 0.026

HU HY 1 0.026

HU JB 1 0.026

HU JL 1 0.026

HU JP 1 0.026

HU JX 1 0.026

HU JY 1 0.026

HU K 1 0.026

HU KB 1 0.026

HU KQ 1 0.026

HU L 1 0.026

HU LM 1 0.026

HU LT 1 0.026

HU MH 1 0.026

HU QZ 1 0.026

HU RM 1 0.026

HU RZ 1 0.026

HU S 1 0.026

HU SS 1 0.026

HU SX 1 0.026

HU TT 1 0.026

HU XJ 1 0.026

HU XT 1 0.026

HU XY 1 0.026

HU Y 1 0.026

HU YB 1 0.026

HU YH 1 0.026

HU YM 1 0.026

HU YR 1 0.026

HU YY 1 0.026

HU ZB 1 0.026

HU ZL 1 0.026

HU ZY 1 0.026

HUA B 1 0.026

HUA F 1 0.026

HUA HL 1 0.026

HUA W 1 0.026

HUA ZH 1 0.026

HUANG BR 1 0.026

HUANG BY 1 0.026

HUANG CX 1 0.026

HUANG DG 1 0.026

HUANG DY 1 0.026

HUANG F 1 0.026

HUANG FX 1 0.026

HUANG GX 1 0.026

HUANG GY 1 0.026

HUANG HC 1 0.026

HUANG HT 1 0.026

HUANG HZ 1 0.026

HUANG JC 1 0.026

HUANG JJ 1 0.026

HUANG JL 1 0.026

HUANG KH 1 0.026

HUANG LC 1 0.026

HUANG MC 1 0.026

HUANG MM 1 0.026

HUANG NJ 1 0.026

HUANG PH 1 0.026

HUANG R 1 0.026

HUANG RM 1 0.026

HUANG RS 1 0.026

HUANG RY 1 0.026

HUANG SC 1 0.026

HUANG SF 1 0.026

HUANG SG 1 0.026

HUANG SJ 1 0.026

HUANG SY 1 0.026

HUANG T 1 0.026

HUANG THW 1 0.026

HUANG WH 1 0.026

HUANG WY 1 0.026

HUANG XH 1 0.026

HUANG XM 1 0.026

HUANG XS 1 0.026

HUANG XW 1 0.026

HUANG YB 1 0.026

HUANG YR 1 0.026

HUANG YY 1 0.026

HUANG YZ 1 0.026

HUANG ZX 1 0.026

HUART V 1 0.026

HUBCHAK SC 1 0.026

HUBER RM 1 0.026

HUBER TB 1 0.026

HUBERT D 1 0.026

HUBERT-BURON A 1 0.026

HUDKINS K 1 0.026

HUDSON B 1 0.026

HUEPER K 1 0.026

HUET S 1 0.026

HUFFAM SE 1 0.026

HUGHES FM 1 0.026

HUGHES JM 1 0.026

HUGHES SV 1 0.026

HUH J 1 0.026

HUH JY 1 0.026

HUH TL 1 0.026

HUH W 1 0.026

HUH YH 1 0.026

HUI Y 1 0.026

HUIKURI HV 1 0.026

HULL KL 1 0.026

HULTCRANTZ R 1 0.026

HUMAR A 1 0.026

HUMMEL A 1 0.026

HUMPHRIES D 1 0.026

HUNG CH 1 0.026

HUNG CL 1 0.026

HUNG MC 1 0.026

HUNG TW 1 0.026

HUNTER J 1 0.026

HUNTZICKER EG 1 0.026

HUNZIKER W 1 0.026

HUO JC 1 0.026

HUO RR 1 0.026

HUO YQ 1 0.026

HUPFELD C 1 0.026

HUPPE D 1 0.026

HUR DY 1 0.026

HURAULT DB 1 0.026

HURST LA 1 0.026

HUSEN B 1 0.026

HUSHMANDI K 1 0.026

HUSSEIN AM 1 0.026

HUSSEIN MRA 1 0.026

HUSSEIN TA 1 0.026

HUSSIEN NI 1 0.026

HUSSON CP 1 0.026

HUTTEMANN M 1 0.026

HUTTL M 1 0.026

HUTTON HL 1 0.026

HUYNH K 1 0.026

HWANG EHE 1 0.026

HWANG H 1 0.026

HWANG JS 1 0.026

HWANG JY 1 0.026

HWANG PA 1 0.026

HWANG S 1 0.026

HYDER S 1 0.026

HYODO T 1 0.026

HYOGO H 1 0.026

HYON JY 1 0.026

HYUN YY 1 0.026

IABLOKOV V 1 0.026

IACCARINO L 1 0.026

IACOMINI J 1 0.026

IBERNON M 1 0.026

IBRAHIM NHM 1 0.026

IBRAHIM TM 1 0.026

IBRAHIM YM 1 0.026

ICHIDA T 1 0.026

ICHIMARU N 1 0.026

ICHIMURA Y 1 0.026

ICZKOWSKI KA 1 0.026

IDO A 1 0.026

IEKUSHI K 1 0.026

IELAPI N 1 0.026

IENI A 1 0.026

IERVOLINO A 1 0.026

IGARASHI P 1 0.026

IGARASHI Y 1 0.026

IGNARSKI M 1 0.026

IHARA K 1 0.026

IHARA T 1 0.026

IHARA Y 1 0.026

IIJIMA K 1 0.026

IKARI A 1 0.026

IKEBUCHI F 1 0.026

IKEDA H 1 0.026

IKEDA Y 1 0.026

IKEGAYA N 1 0.026

ILAGAN R 1 0.026

ILAN N 1 0.026

ILICETO S 1 0.026

IM GYJ 1 0.026

IM K 1 0.026

IM SS 1 0.026

IMAGAWA A 1 0.026

IMAI E 1 0.026

IMAI K 1 0.026

IMAI M 1 0.026

IMAI T 1 0.026

IMAJO K 1 0.026

IMAKFIRE T 1 0.026

IMAMURA Y 1 0.026

IMARUOKA K 1 0.026

IMAZU M 1 0.026

IMIKIRENE L 1 0.026

IMRAN M 1 0.026

IMRAN TF 1 0.026

INADA C 1 0.026

INAGAKI Y 1 0.026

INAMI M 1 0.026

INAN O 1 0.026

INCEOGLU B 1 0.026

INGERSKI LM 1 0.026

INO F 1 0.026

INOGUCHI T 1 0.026

INOKUCHI T 1 0.026

INOMATA S 1 0.026

INOUE A 1 0.026

INOUE MK 1 0.026

INUKAI K 1 0.026

INVERARDI L 1 0.026

INVERNIZZI L 1 0.026

IOANA M 1 0.026

IOZZO RV 1 0.026

IP TP 1 0.026

IPPOLITO S 1 0.026

IRIFUKU T 1 0.026

IRINOPOULOU T 1 0.026

ISAAC AT 1 0.026

ISAACS SM 1 0.026

ISABEL B 1 0.026

ISAKOVA T 1 0.026

ISEKI K 1 0.026

ISERI K 1 0.026

ISHIBASHI K 1 0.026

ISHIBASHI S 1 0.026

ISHIBASHI-UEDA H 1 0.026

ISHIGAKI S 1 0.026

ISHIGURO YS 1 0.026

ISHIHARA M 1 0.026

ISHIHARA S 1 0.026

ISHII I 1 0.026

ISHII N 1 0.026

ISHIKAWA H 1 0.026

ISHIKAWA M 1 0.026

ISHIKAWA N 1 0.026

ISHIKI T 1 0.026

ISHIMOTO T 1 0.026

ISHIMURA T 1 0.026

ISHIZAWA K 1 0.026

ISHIZUKA N 1 0.026

ISLAM MN 1 0.026

ISLAM R 1 0.026

ISOBE S 1 0.026

ISRAELI T 1 0.026

ITABASHI N 1 0.026

ITAQUY TP 1 0.026

ITO M 1 0.026

ITOH N 1 0.026

ITOH T 1 0.026

ITOH Y 1 0.026

ITTO R 1 0.026

IVAN L 1 0.026

IVANAC-JANKOVIC R 1 0.026

IVANOVA E 1 0.026

IVANOVA M 1 0.026

IVANOVSKI N 1 0.026

IVANYI B 1 0.026

IVANYI S 1 0.026

IWABAYASHI M 1 0.026

IWAI N 1 0.026

IWAKAWA K 1 0.026

IWAKURA T 1 0.026

IWAMOTO T 1 0.026

IWANO M 1 0.026

IWAO M 1 0.026

IWAO Y 1 0.026

IWASHITA M 1 0.026

IWASHITA T 1 0.026

IWATA Y 1 0.026

IWATANI H 1 0.026

IWATANI R 1 0.026

IYENGAR S 1 0.026

IYENGAR SK 1 0.026

IYODA M 1 0.026

IZAWA-ISHIZAWA Y 1 0.026

IZQUIERDO MC 1 0.026

IZUHARA Y 1 0.026

JAAFAR A 1 0.026

JABANDZIEV P 1 0.026

JABLONOWSKI Z 1 0.026

JABLONSKI M 1 0.026

JACKSON EK 1 0.026

JACKSON S 1 0.026

JACOB A 1 0.026

JACOBS JM 1 0.026

JACOBS L 1 0.026

JACOBSEN PK 1 0.026

JACOBY DL 1 0.026

JACUPS SP 1 0.026

JADHAV A 1 0.026

JADOUL M 1 0.026

JAFFA A 1 0.026

JAGADEESH G 1 0.026

JAIMES EA 1 0.026

JAIN D 1 0.026

JAIN G 1 0.026

JAIN MR 1 0.026

JAIN P 1 0.026

JAIN S 1 0.026

JAIRAJPURI DS 1 0.026

JAKLOFSKY M 1 0.026

JALA VR 1 0.026

JALANKO H 1 0.026

JAMAL R 1 0.026

JAMAL W 1 0.026

JAMES AH 1 0.026

JAMES LR 1 0.026

JAMIE MF 1 0.026

JAMIESON NV 1 0.026

JANG HR 1 0.026

JANG JE 1 0.026

JANG KY 1 0.026

JANG SH 1 0.026

JANG WS 1 0.026

JANG Y 1 0.026

JANI P 1 0.026

JANICOVA A 1 0.026

JANKOWSKA EA 1 0.026

JANKOWSKI K 1 0.026

JANKOWSKI P 1 0.026

JANKOWSKI V 1 0.026

JANKUN J 1 0.026

JANSEN-DUERR P 1 0.026

JANSSEN MR 1 0.026

JANSSON L 1 0.026

JANSSON-LOFMARK R 1 0.026

JANTOS J 1 0.026

JAO TM 1 0.026

JARAMILLO K 1 0.026

JARDEL S 1 0.026

JAROLIM P 1 0.026

JAROLIMEK W 1 0.026

JATKAR A 1 0.026

JATTI K 1 0.026

JAULERRY C 1 0.026

JAVADOV S 1 0.026

JAVED A 1 0.026

JAVED F 1 0.026

JAYACHANDRAN M 1 0.026

JAYO M 1 0.026

JE J 1 0.026

JEANNE-PASQUIER C 1 0.026

JEANNERET VA 1 0.026

JECMENICA J 1 0.026

JELSING J 1 0.026

JENKIN KA 1 0.026

JENKINS J 1 0.026

JENKINS R 1 0.026

JENKINS RH 1 0.026

JENNE CN 1 0.026

JENNETTE JC 1 0.026

JENNINGS DL 1 0.026

JENSSEN T 1 0.026

JEON HJ 1 0.026

JEON SM 1 0.026

JEON Y 1 0.026

JEON YJ 1 0.026

JEONG BY 1 0.026

JEONG HG 1 0.026

JEONG JG 1 0.026

JEONG JU 1 0.026

JEONG K 1 0.026

JEPPSSON A 1 0.026

JERCAN O 1 0.026

JERMUTUS L 1 0.026

JERONIMO SMB 1 0.026

JEVNIKAR AM 1 0.026

JEYASEELAN K 1 0.026

JHANG HR 1 0.026

JHANG YR 1 0.026

JHONSA D 1 0.026

JHUANG WJ 1 0.026

JHUO SJ 1 0.026

JI HF 1 0.026

JI JQ 1 0.026

JI LQ 1 0.026

JI M 1 0.026

JI QH 1 0.026

JI SY 1 0.026

JI SZ 1 0.026

JI T 1 0.026

JI X 1 0.026

JI XF 1 0.026

JI XH 1 0.026

JI XM 1 0.026

JI XP 1 0.026

JI XQ 1 0.026

JI YQ 1 0.026

JIA FY 1 0.026

JIA GH 1 0.026

JIA GL 1 0.026

JIA HP 1 0.026

JIA JH 1 0.026

JIA JS 1 0.026

JIA LJ 1 0.026

JIA T 1 0.026

JIA WP 1 0.026

JIA XJ 1 0.026

JIA YL 1 0.026

JIA ZJ 1 0.026

JIALAL I 1 0.026

JIAN WX 1 0.026

JIANG B 1 0.026

JIANG CH 1 0.026

JIANG CX 1 0.026

JIANG HJ 1 0.026

JIANG HL 1 0.026

JIANG J 1 0.026

JIANG JM 1 0.026

JIANG JY 1 0.026

JIANG K 1 0.026

JIANG LM 1 0.026

JIANG MH 1 0.026

JIANG MN 1 0.026

JIANG MQ 1 0.026

JIANG O 1 0.026

JIANG QH 1 0.026

JIANG SN 1 0.026

JIANG SZ 1 0.026

JIANG TT 1 0.026

JIANG WB 1 0.026

JIANG WD 1 0.026

JIANG X 1 0.026

JIANG XB 1 0.026

JIANG XL 1 0.026

JIANG XY 1 0.026

JIANG YC 1 0.026

JIANG YF 1 0.026

JIANG YJ 1 0.026

JIANG YM 1 0.026

JIANG YX 1 0.026

JIANG ZH 1 0.026

JIANG ZQ 1 0.026

JIANG ZX 1 0.026

JIAO B 1 0.026

JIAO FZ 1 0.026

JIAO Y 1 0.026

JIAO YJ 1 0.026

JIE R 1 0.026

JIE XN 1 0.026

JIG GY 1 0.026

JIM B 1 0.026

JIMENEZ F 1 0.026

JIMENEZ-CASTILLA L 1 0.026

JIN BMWJ 1 0.026

JIN CX 1 0.026

JIN HG 1 0.026

JIN HM 1 0.026

JIN HY 1 0.026

JIN JZ 1 0.026

JIN MW 1 0.026

JIN O 1 0.026

JIN R 1 0.026

JIN SY 1 0.026

JIN W 1 0.026

JIN WJ 1 0.026

JIN XD 1 0.026

JIN XH 1 0.026

JIN XJ 1 0.026

JIN XP 1 0.026

JIN YS 1 0.026

JIN YY 1 0.026

JIN ZH 1 0.026

JING KP 1 0.026

JING X 1 0.026

JING Y 1 0.026

JING YH 1 0.026

JING ZY 1 0.026

JIRONDA C 1 0.026

JIVISHOV E 1 0.026

JJANG XY 1 0.026

JO CH 1 0.026

JO HA 1 0.026

JO M 1 0.026

JOCHMANS I 1 0.026

JOEKEN S 1 0.026

JOFFE MM 1 0.026

JOHANSEN TT 1 0.026

JOHARAPURKAR A 1 0.026

JOHARAPURKAR AA 1 0.026

JOHN AMSP 1 0.026

JOHN NT 1 0.026

JOHNSON BG 1 0.026

JOHNSON EC 1 0.026

JOHNSON EK 1 0.026

JOHNSON J 1 0.026

JOHNSON M 1 0.026

JOHNSON R 1 0.026

JOHNSON T 1 0.026

JOHNSTON CI 1 0.026

JOHNSTON N 1 0.026

JOKI Y 1 0.026

JOLY L 1 0.026

JONAS M 1 0.026

JONASSON L 1 0.026

JONES CL 1 0.026

JONES HB 1 0.026

JONES M 1 0.026

JONES R 1 0.026

JONES RA 1 0.026

JONES S 1 0.026

JONES SC 1 0.026

JONES SG 1 0.026

JOO H 1 0.026

JOO KW 1 0.026

JOO SK 1 0.026

JORDA M 1 0.026

JORDAN J 1 0.026

JORGENSEN PE 1 0.026

JORNET AR 1 0.026

JOSEPH AEA 1 0.026

JOSEPH J 1 0.026

JOTWANI V 1 0.026

JOUANNEAU C 1 0.026

JOUBERT M 1 0.026

JOUBERT P 1 0.026

JOURDAN T 1 0.026

JOVEN J 1 0.026

JU JM 1 0.026

JU JN 1 0.026

JU KD 1 0.026

JU W 1 0.026

JU ZC 1 0.026

JUAN SH 1 0.026

JUAREZ-ROJAS JG 1 0.026

JUDGE P 1 0.026

JUGOLD M 1 0.026

JULIO SMC 1 0.026

JUN HH 1 0.026

JUNAID A 1 0.026

JUNCOS LA 1 0.026

JUNG DH 1 0.026

JUNG DS 1 0.026

JUNG DY 1 0.026

JUNG ES 1 0.026

JUNG HW 1 0.026

JUNG JH 1 0.026

JUNG JM 1 0.026

JUNG JY 1 0.026

JUNG KA 1 0.026

JUNG KJ 1 0.026

JUNG KS 1 0.026

JUNG N 1 0.026

JUNG O 1 0.026

JUNG SC 1 0.026

JUNG SK 1 0.026

JUNG YJ 1 0.026

JUNTTILA MJ 1 0.026

JUSDADO JJ 1 0.026

KABIR MG 1 0.026

KABUYE D 1 0.026

KACER P 1 0.026

KACEROVA T 1 0.026

KACEW S 1 0.026

KACSO G 1 0.026

KACSO IM 1 0.026

KADAPPU KK 1 0.026

KADDAI V 1 0.026

KADIAN S 1 0.026

KADLECOVA M 1 0.026

KADOMATSU K 1 0.026

KADONO T 1 0.026

KADOR PF 1 0.026

KADOWAKI D 1 0.026

KADOWAKI T 1 0.026

KAESEMEYER WH 1 0.026

KAEWARPAI T 1 0.026

KAHAER A 1 0.026

KAHAN BD 1 0.026

KAHKONEN D 1 0.026

KAHKOSKA AR 1 0.026

KAHNG KW 1 0.026

KAI-MING C 1 0.026

KAIKINI AA 1 0.026

KAISAKI PJ 1 0.026

KAISSLING B 1 0.026

KAJIKAWA M 1 0.026

KAKIMOTO T 1 0.026

KAKOKI M 1 0.026

KALECHMAN Y 1 0.026

KALGUTKAR AS 1 0.026

KALISZ KR 1 0.026

KALITA J 1 0.026

KALITA-DE CROFT P 1 0.026

KALLSKOG O 1 0.026

KALTENBACH R 1 0.026

KALYANARAMAN B 1 0.026

KAM KKH 1 0.026

KAM-TAO LP 1 0.026

KAMADA Y 1 0.026

KAMALI-SARVESTANI E 1 0.026

KAMALOV M 1 0.026

KAMBARA T 1 0.026

KAMBHAM N 1 0.026

KAMEL G 1 0.026

KAMEL S 1 0.026

KAMEZAKI K 1 0.026

KAMGAR M 1 0.026

KAMIMURA D 1 0.026

KAMIMURA K 1 0.026

KAMIYA K 1 0.026

KAMMERLANDER AA 1 0.026

KAMOUCHI M 1 0.026

KAMPS JAAM 1 0.026

KAN M 1 0.026

KANAAN H 1 0.026

KANAI T 1 0.026

KANAZAWA N 1 0.026

KANCHAN DM 1 0.026

KANCHANA K 1 0.026

KANDA E 1 0.026

KANDALAM V 1 0.026

KANDASAMY N 1 0.026

KANEDA K 1 0.026

KANEKO K 1 0.026

KANEKO M 1 0.026

KANEKO R 1 0.026

KANEKO Y 1 0.026

KANEMATSU A 1 0.026

KANEMATSU T 1 0.026

KANEMOTO K 1 0.026

KANEYAMA T 1 0.026

KANFER A 1 0.026

KANG CM 1 0.026

KANG H 1 0.026

KANG HM 1 0.026

KANG IJ 1 0.026

KANG JH 1 0.026

KANG KP 1 0.026

KANG KS 1 0.026

KANG SH 1 0.026

KANG SY 1 0.026

KANG YX 1 0.026

KANLAYA R 1 0.026

KANN M 1 0.026

KAOUK JH 1 0.026

KAPOOR A 1 0.026

KAPOOR S 1 0.026

KAPOOR T 1 0.026

KAPOSZTAS Z 1 0.026

KAPS L 1 0.026

KAPTURCZAK M 1 0.026

KAPUS A 1 0.026

KARA O 1 0.026

KARACA E 1 0.026

KARADI Z 1 0.026

KARAGIANNI F 1 0.026

KARAGOZ A 1 0.026

KARALLIEDDE J 1 0.026

KARATAS A 1 0.026

KARATAS MB 1 0.026

KARBOWSKA A 1 0.026

KARDOS M 1 0.026

KARETSKAYA L 1 0.026

KARI JA 1 0.026

KARIBE S 1 0.026

KARIHALOO A 1 0.026

KARIM K 1 0.026

KARLBERG N 1 0.026

KARLBERG S 1 0.026

KARMAKAR S 1 0.026

KARMOUS-BENAILLY H 1 0.026

KARNIB HH 1 0.026

KAROLINA DS 1 0.026

KAROLKO B 1 0.026

KARP R 1 0.026

KARPMAN E 1 0.026

KARRAS A 1 0.026

KARTHA GK 1 0.026

KARUMANCHI SA 1 0.026

KARUNASAGARA S 1 0.026

KARUPARTHI PR 1 0.026

KARUPPAGOUNDER V 1 0.026

KASAI N 1 0.026

KASAPOGLU B 1 0.026

KASCHINA E 1 0.026

KASHIWABARA H 1 0.026

KASISKE B 1 0.026

KASKEL FJ 1 0.026

KASPER M 1 0.026

KASPI A 1 0.026

KASRAIE A 1 0.026

KASSIANOS AJ 1 0.026

KASSIRI Z 1 0.026

KASSOUF HK 1 0.026

KASUGA H 1 0.026

KATAOKA K 1 0.026

KATAOKA S 1 0.026

KATAOKA Y 1 0.026

KATAYAMA A 1 0.026

KATAYAMA K 1 0.026

KATERELOS M 1 0.026

KATO A 1 0.026

KATO H 1 0.026

KATO I 1 0.026

KATO T 1 0.026

KATOPODIS K 1 0.026

KATSIKI N 1 0.026

KATSUBE F 1 0.026

KATSUDA Y 1 0.026

KATSUMATA Y 1 0.026

KATSUOKA K 1 0.026

KATSURADA A 1 0.026

KATZ A 1 0.026

KATZ R 1 0.026

KATZENSTEIN TL 1 0.026

KAUFMAN L 1 0.026

KAUFMANN TJ 1 0.026

KAUL A 1 0.026

KAUPKE CJ 1 0.026

KAUR R 1 0.026

KAUSALYA PJ 1 0.026

KAUSHAL GP 1 0.026

KAUSMAN JY 1 0.026

KAUTZKY-WILLER A 1 0.026

KAVA BR 1 0.026

KAVANAGH D 1 0.026

KAVURMA MM 1 0.026

KAVVADAS P 1 0.026

KAWACHI H 1 0.026

KAWADA N 1 0.026

KAWAI T 1 0.026

KAWAKAMI T 1 0.026

KAWAMURA T 1 0.026

KAWANAKA M 1 0.026

KAWANISHI T 1 0.026

KAWANO KI 1 0.026

KAWANO Y 1 0.026

KAWASAKI E 1 0.026

KAWATO Y 1 0.026

KAWSARA A 1 0.026

KAYA A 1 0.026

KAYA C 1 0.026

KAYE DM 1 0.026

KAZA V 1 0.026

KAZAKOV A 1 0.026

KAZE AD 1 0.026

KAZMI I 1 0.026

KE BX 1 0.026

KE LY 1 0.026

KE QQ 1 0.026

KEATING A 1 0.026

KEBAPCILAR L 1 0.026

KEBIS A 1 0.026

KECHAGIAS S 1 0.026

KECHAVARZI BD 1 0.026

KEKRE N 1 0.026

KEL B 1 0.026

KELLER BJ 1 0.026

KELLER MP 1 0.026

KELLEY KM 1 0.026

KELLEY R 1 0.026

KELLY JP 1 0.026

KEMENY E 1 0.026

KEMMOCHI Y 1 0.026

KEMP BE 1 0.026

KEMPEGOWDA P 1 0.026

KEMPERMAN H 1 0.026

KEMPF H 1 0.026

KENNEDY CR 1 0.026

KENNEDY J 1 0.026

KENTTA T 1 0.026

KEPECS D 1 0.026

KEPECS DM 1 0.026

KEPPEL MH 1 0.026

KER YB 1 0.026

KERI G 1 0.026

KERROCH M 1 0.026

KERSCHER C 1 0.026

KERTESZ D 1 0.026

KESSAI A 1 0.026

KESTENBAUM BR 1 0.026

KEUNG K 1 0.026

KEVIL CG 1 0.026

KHAKOO NS 1 0.026

KHALIL A 1 0.026

KHAMAYSI I 1 0.026

KHAMMAR A 1 0.026

KHAN F 1 0.026

KHAN M 1 0.026

KHAN MAF 1 0.026

KHAN MN 1 0.026

KHAN MSH 1 0.026

KHAN NA 1 0.026

KHAN NH 1 0.026

KHAN SI 1 0.026

KHAN SS 1 0.026

KHAN Z 1 0.026

KHANDOGA A 1 0.026

KHANDOGA AG 1 0.026

KHANRA R 1 0.026

KHASIGOV PZ 1 0.026

KHATTAK S 1 0.026

KHAZAELI M 1 0.026

KHEIRI B 1 0.026

KHODADADI S 1 0.026

KHOKHAR M 1 0.026

KHOO JB 1 0.026

KHULLAR M 1 0.026

KHUTSISHVILI K 1 0.026

KHWAJA A 1 0.026

KIELSTEIN JT 1 0.026

KIHARA S 1 0.026

KIHM LP 1 0.026

KIKKAWA R 1 0.026

KIKUCHI S 1 0.026

KIKUMOTO Y 1 0.026

KIKUTA T 1 0.026

KILARI EK 1 0.026

KILARKAJE N 1 0.026

KILINC F 1 0.026

KILLEEN A 1 0.026

KILMARTIN G 1 0.026

KIM AH 1 0.026

KIM BK 1 0.026

KIM CH 1 0.026

KIM CT 1 0.026

KIM DI 1 0.026

KIM DS 1 0.026

KIM E 1 0.026

KIM ES 1 0.026

KIM EY 1 0.026

KIM GH 1 0.026

KIM JI 1 0.026

KIM JJ 1 0.026

KIM JR 1 0.026

KIM JW 1 0.026

KIM JY 1 0.026

KIM K 1 0.026

KIM KM 1 0.026

KIM KP 1 0.026

KIM KT 1 0.026

KIM MS 1 0.026

KIM NY 1 0.026

KIM SD 1 0.026

KIM SG 1 0.026

KIM SI 1 0.026

KIM SS 1 0.026

KIM SU 1 0.026

KIM TN 1 0.026

KIM TW 1 0.026

KIM TY 1 0.026

KIM UH 1 0.026

KIM YL 1 0.026

KIM YO 1 0.026

KIMACHI T 1 0.026

KIMOTO M 1 0.026

KIMURA A 1 0.026

KIMURA M 1 0.026

KIMURA T 1 0.026

KINAE N 1 0.026

KINDERMANN I 1 0.026

KING RJ 1 0.026

KING ST 1 0.026

KING-AHMAD A 1 0.026

KINLEY B 1 0.026

KINNE RW 1 0.026

KINNEALLY TL 1 0.026

KINOMURA M 1 0.026

KINOSHITA J 1 0.026

KINOWAKI K 1 0.026

KINSEY GR 1 0.026

KIORTSIS DN 1 0.026

KIPAR A 1 0.026

KIRBY BJ 1 0.026

KIRBY K 1 0.026

KIRCHER S 1 0.026

KIRIAZIS H 1 0.026

KIRIMCA F 1 0.026

KIRPALANI A 1 0.026

KIRWAN PD 1 0.026

KISER M 1 0.026

KISHI S 1 0.026

KISHIMOTO I 1 0.026

KISHIMOTO S 1 0.026

KISHORE BK 1 0.026

KISHORE K 1 0.026

KISTLER A 1 0.026

KITA T 1 0.026

KITABAYASHI C 1 0.026

KITAGAWA K 1 0.026

KITAGAWA M 1 0.026

KITAJIMA M 1 0.026

KITAKAZE M 1 0.026

KITAMUR K 1 0.026

KITAYAMA T 1 0.026

KITAZONO T 1 0.026

KITO N 1 0.026

KITSUNAI H 1 0.026

KITTEN GT 1 0.026

KIVINIEMI AM 1 0.026

KIYOSAWA K 1 0.026

KJAER A 1 0.026

KJELDSEN K 1 0.026

KLAASSEN I 1 0.026

KLAMER S 1 0.026

KLASS C 1 0.026

KLAVINS K 1 0.026

KLEBER ME 1 0.026

KLEEMANN R 1 0.026

KLEIN JD 1 0.026

KLEIN R 1 0.026

KLEIN RL 1 0.026

KLESSENS CQF 1 0.026

KLIMPFINGER M 1 0.026

KLINGENBERG NC 1 0.026

KLINGER M 1 0.026

KLINGLER H 1 0.026

KLINKER H 1 0.026

KLOMJIT N 1 0.026

KLOTMAN PE 1 0.026

KLOUVAS G 1 0.026

KLUSSMANN S 1 0.026

KLUTH D 1 0.026

KLUTH DC 1 0.026

KNAFL K 1 0.026

KNAUF F 1 0.026

KNEBELMANN B 1 0.026

KNEPPER MA 1 0.026

KNUCHEL R 1 0.026

KNUDSEN BE 1 0.026

KNUDSEN ST 1 0.026

KNYAZER B 1 0.026

KO GJ 1 0.026

KO JR 1 0.026

KO KS 1 0.026

KO M 1 0.026

KO SH 1 0.026

KO YC 1 0.026

KOAL T 1 0.026

KOBAYASHI E 1 0.026

KOBAYASHI M 1 0.026

KOBAYASHI N 1 0.026

KOBES S 1 0.026

KOBOLD ACM 1 0.026

KOBUSIAK-PROKOPOWICZ M 1 0.026

KOC M 1 0.026

KOCH A 1 0.026

KOCH KA 1 0.026

KOCIC G 1 0.026

KODAMA G 1 0.026

KODERA R 1 0.026

KOELLING M 1 0.026

KOELSCH K 1 0.026

KOEPKE ML 1 0.026

KOESTERS R 1 0.026

KOGOT-LEVIN A 1 0.026

KOH DM 1 0.026

KOH ES 1 0.026

KOH GY 1 0.026

KOH HJ 1 0.026

KOH J 1 0.026

KOH TS 1 0.026

KOHAGURA K 1 0.026

KOHDA Y 1 0.026

KOHJIMA M 1 0.026

KOHL M 1 0.026

KOHLER A 1 0.026

KOHLI HS 1 0.026

KOHLI P 1 0.026

KOHN O 1 0.026

KOHNO N 1 0.026

KOIBUCHI N 1 0.026

KOIDE K 1 0.026

KOIKE N 1 0.026

KOIKE T 1 0.026

KOIRALA J 1 0.026

KOITKA-WEBER A 1 0.026

KOIVISTO VA 1 0.026

KOJIMA H 1 0.026

KOJIMA K 1 0.026

KOJURI J 1 0.026

KOK SW 1 0.026

KOKA V 1 0.026

KOKTZOGLOU I 1 0.026

KOKUBO S 1 0.026

KOLA I 1 0.026

KOLATSI-JOANNOU M 1 0.026

KOLB L 1 0.026

KOLKHOF P 1 0.026

KOLODKIN-GAL I 1 0.026

KOLSET SO 1 0.026

KOMATSU K 1 0.026

KOMATSUDA A 1 0.026

KOMINAMI R 1 0.026

KOMIYA C 1 0.026

KOMUNE N 1 0.026

KOMUNE S 1 0.026

KOMURA N 1 0.026

KOMURO I 1 0.026

KON V 1 0.026

KON Y 1 0.026

KONARI N 1 0.026

KONCSOS G 1 0.026

KONDETI VK 1 0.026

KONDO T 1 0.026

KONENKOV VI 1 0.026

KONG APS 1 0.026

KONG DY 1 0.026

KONG F 1 0.026

KONG KH 1 0.026

KONG MY 1 0.026

KONG Q 1 0.026

KONG YZ 1 0.026

KONG ZL 1 0.026

KONGSUPHOL P 1 0.026

KONGYINGYOES B 1 0.026

KONIGER J 1 0.026

KONKALMATT P 1 0.026

KONO K 1 0.026

KONO N 1 0.026

KONO T 1 0.026

KONRAD D 1 0.026

KONRAD K 1 0.026

KONRAD M 1 0.026

KONSTANTOPOULOS K 1 0.026

KONWAR N 1 0.026

KOO BK 1 0.026

KOOISTRA T 1 0.026

KOOTI W 1 0.026

KOPKAN L 1 0.026

KOPP J 1 0.026

KOPPEL H 1 0.026

KOPPLE JD 1 0.026

KOPYLOV P 1 0.026

KOR CT 1 0.026

KORNEJ J 1 0.026

KORRAPATI M 1 0.026

KORRER S 1 0.026

KOSAKA T 1 0.026

KOSAKI K 1 0.026

KOSCHUTNIK M 1 0.026

KOSEKI M 1 0.026

KOSHIMURA J 1 0.026

KOSIERADZKI M 1 0.026

KOSIUK J 1 0.026

KOSMALA W 1 0.026

KOSOWSKI J 1 0.026

KOSTEV K 1 0.026

KOSUGI T 1 0.026

KOTOH K 1 0.026

KOTOLLOSHI R 1 0.026

KOTSIMBOS T 1 0.026

KOTTGEN A 1 0.026

KOTWICE T 1 0.026

KOU DH 1 0.026

KOU WX 1 0.026

KOUGIAS P 1 0.026

KOULIS C 1 0.026

KOUNTOURAS J 1 0.026

KOUTROULIS I 1 0.026

KOWALCZUK L 1 0.026

KOYAMA A 1 0.026

KOZAKOVA M 1 0.026

KOZINSKI M 1 0.026

KRAFT F 1 0.026

KRAFT R 1 0.026

KRALIK PM 1 0.026

KRAMER J 1 0.026

KRAMER U 1 0.026

KRANE V 1 0.026

KRANZLIN B 1 0.026

KRAPPE J 1 0.026

KRAUS AC 1 0.026

KRAUSE KH 1 0.026

KRAUSE VL 1 0.026

KRAUSZ KW 1 0.026

KRAZIT ST 1 0.026

KREBS C 1 0.026

KREDIET RT 1 0.026

KREEGER JM 1 0.026

KREIS H 1 0.026

KRENNING G 1 0.026

KRESTIN GP 1 0.026

KRETSCHMAR C 1 0.026

KRETSCHMER A 1 0.026

KRETZ O 1 0.026

KRIBBEN A 1 0.026

KRINTUS M 1 0.026

KRISHAN P 1 0.026

KRISHNAMOORTHY P 1 0.026

KRISTENSEN DH 1 0.026

KRISTENSEN JH 1 0.026

KRIZOVA A 1 0.026

KROMBACH F 1 0.026

KRONBICHLER A 1 0.026

KRUEGER B 1 0.026

KRUGER C 1 0.026

KRUGER M 1 0.026

KRUGER WA 1 0.026

KSHIRSAGAR SG 1 0.026

KSOURI S 1 0.026

KTZOEVA SA 1 0.026

KU B 1 0.026

KU CH 1 0.026

KU CL 1 0.026

KU PM 1 0.026

KUBICA J 1 0.026

KUBO K 1 0.026

KUBO M 1 0.026

KUBOTA E 1 0.026

KUBOTA N 1 0.026

KUBUROVIC N 1 0.026

KUBUROVIC V 1 0.026

KUDER JF 1 0.026

KUDO S 1 0.026

KUGITA M 1 0.026

KUHL D 1 0.026

KUIPER EJ 1 0.026

KUKAN M 1 0.026

KUKLA A 1 0.026

KUKONGVIRIYAPAN U 1 0.026

KUKONGVIRIYAPAN V 1 0.026

KUKULL B 1 0.026

KULA AJ 1 0.026

KULISZEWSKI MA 1 0.026

KULKARNI OP 1 0.026

KULLER JA 1 0.026

KUMAGAI H 1 0.026

KUMAGAI T 1 0.026

KUMAKURA S 1 0.026

KUMAR D 1 0.026

KUMAR KMP 1 0.026

KUMAR MSA 1 0.026

KUMAR SA 1 0.026

KUMAR SV 1 0.026

KUMAR V 1 0.026

KUMARSWAMY R 1 0.026

KUMATORI A 1 0.026

KUMPERS P 1 0.026

KUNISCH E 1 0.026

KUNORI S 1 0.026

KUNOS G 1 0.026

KUNOVSKY L 1 0.026

KUNTER U 1 0.026

KUNZELMANN K 1 0.026

KUO CS 1 0.026

KUO CW 1 0.026

KUO HL 1 0.026

KUO HT 1 0.026

KUO JY 1 0.026

KUO TM 1 0.026

KUO WH 1 0.026

KUO YR 1 0.026

KUO YT 1 0.026

KUPIN W 1 0.026

KURA-NAKAMURA N 1 0.026

KURODA K 1 0.026

KUROSAKI E 1 0.026

KUROSAWA M 1 0.026

KURUMBAIL RG 1 0.026

KURZROCK EA 1 0.026

KUSAKA H 1 0.026

KUSAKA Y 1 0.026

KUSANO E 1 0.026

KUSCHE-GULLBERG M 1 0.026

KUSHIYAMA A 1 0.026

KUSHIYAMA T 1 0.026

KUSZTAL M 1 0.026

KUTALA B 1 0.026

KUTBAY NO 1 0.026

KUTIL B 1 0.026

KUTSAL A 1 0.026

KUWAJIMA M 1 0.026

KUWATSUKA Y 1 0.026

KUYPERS D 1 0.026

KWABI-ADDO B 1 0.026

KWAK HB 1 0.026

KWAK IS 1 0.026

KWAK JH 1 0.026

KWAK SJ 1 0.026

KWAN JYY 1 0.026

KWAN L 1 0.026

KWAN TK 1 0.026

KWO PY 1 0.026

KWOK R 1 0.026

KWON GY 1 0.026

KWON HK 1 0.026

KWON K 1 0.026

KWON MK 1 0.026

KWON OS 1 0.026

KWONG ASK 1 0.026

KWUN IS 1 0.026

KYLE PB 1 0.026

KYNDT X 1 0.026

KYTO J 1 0.026

KYULO NL 1 0.026

LA MANNA G 1 0.026

LA XJ 1 0.026

LABENZ C 1 0.026

LABRUNE P 1 0.026

LACARIA A 1 0.026

LACAVA V 1 0.026

LACHIN JM 1 0.026

LACOMBE MJ 1 0.026

LACORTE JM 1 0.026

LACY S 1 0.026

LADENVALL C 1 0.026

LADEROUTE KR 1 0.026

LAGARES D 1 0.026

LAGER D 1 0.026

LAGIEWSKA B 1 0.026

LAGOUTTE E 1 0.026

LAHERA V 1 0.026

LAHERMO P 1 0.026

LAI CS 1 0.026

LAI EY 1 0.026

LAI FF 1 0.026

LAI JYC 1 0.026

LAI KTA 1 0.026

LAI S 1 0.026

LAI SS 1 0.026

LAI TY 1 0.026

LAI XR 1 0.026

LAJER M 1 0.026

LAJTAR E 1 0.026

LAKAT T 1 0.026

LAKKIS J 1 0.026

LALAU JD 1 0.026

LAM CSP 1 0.026

LAM JKY 1 0.026

LAM MF 1 0.026

LAM YY 1 0.026

LAMARRE-CLICHE M 1 0.026

LAMAS ME 1 0.026

LAMAS S 1 0.026

LAMB IM 1 0.026

LAMBE S 1 0.026

LAMBETH JD 1 0.026

LAMERATO L 1 0.026

LAMMEY ML 1 0.026

LAN L 1 0.026

LAN TA 1 0.026

LAN WQ 1 0.026

LAN Z 1 0.026

LAN ZH 1 0.026

LANASPA MA 1 0.026

LANDA CEM 1 0.026

LANDGRAF R 1 0.026

LANDHEER SW 1 0.026

LANDIS RC 1 0.026

LANDOLT-MARTICORENA C 1 0.026

LANDRAY MJ 1 0.026

LANDRIANI N 1 0.026

LANDRY C 1 0.026

LANG H 1 0.026

LANGA C 1 0.026

LANGE A 1 0.026

LANGE K 1 0.026

LANGE M 1 0.026

LANGE S 1 0.026

LANGHAM R 1 0.026

LANGWORTHY MM 1 0.026

LANKARANI KB 1 0.026

LANNG S 1 0.026

LANSDON EB 1 0.026

LANTING LD 1 0.026

LANZONI VP 1 0.026

LAO L 1 0.026

LAPING N 1 0.026

LAPING NJ 1 0.026

LAPPIN D 1 0.026

LARCZYNSKI W 1 0.026

LAROCHELLE P 1 0.026

LARRIVEE B 1 0.026

LARROQUE B 1 0.026

LARSEN AK 1 0.026

LARSEN PW 1 0.026

LARSEN S 1 0.026

LARSON JJ 1 0.026

LARSON MG 1 0.026

LARSON TS 1 0.026

LARSSON A 1 0.026

LASAGNI L 1 0.026

LASH JP 1 0.026

LASKER S 1 0.026

LASSALLE L 1 0.026

LASTRA-GONZALEZ G 1 0.026

LATEEF MA 1 0.026

LATHAM PS 1 0.026

LATHROP M 1 0.026

LATINA M 1 0.026

LATINI R 1 0.026

LATORRE M 1 0.026

LATOSINSKA A 1 0.026

LATTENIST L 1 0.026

LATTMANN T 1 0.026

LAU A 1 0.026

LAU CJ 1 0.026

LAU ESH 1 0.026

LAU K 1 0.026

LAU R 1 0.026

LAUAR AO 1 0.026

LAUFER N 1 0.026

LAUFS U 1 0.026

LAUGESEN CS 1 0.026

LAUKENS D 1 0.026

LAUR O 1 0.026

LAURIN LP 1 0.026

LAURIN P 1 0.026

LAUZIER B 1 0.026

LAVALLIE ER 1 0.026

LAVAUD S 1 0.026

LAVIN D 1 0.026

LAW W 1 0.026

LAWSON G 1 0.026

LAZAR L 1 0.026

LAZARETH I 1 0.026

LAZAREVIC J 1 0.026

LAZICH I 1 0.026

LAZZARI P 1 0.026

LAZZERI E 1 0.026

LBERNON M 1 0.026

LE C 1 0.026

LE HTAY S 1 0.026

LE MERCIER A 1 0.026

LE MERRER M 1 0.026

LE MEUR Y 1 0.026

LE POGAM MA 1 0.026

LE POGAMP P 1 0.026

LE ROUX CW 1 0.026

LE RR 1 0.026

LE T 1 0.026

LE WB 1 0.026

LE WU J 1 0.026

LE Y 1 0.026

LEAF IA 1 0.026

LEAHY P 1 0.026

LEBBAH S 1 0.026

LEBEL M 1 0.026

LEBLOND FA 1 0.026

LEBOUCHER S 1 0.026

LECH M 1 0.026

LECH MP 1 0.026

LECHNER SM 1 0.026

LEDENT C 1 0.026

LEDFORD KL 1 0.026

LEDO N 1 0.026

LEDUC C 1 0.026

LEDUC M 1 0.026

LEE ACH 1 0.026

LEE AK 1 0.026

LEE CC 1 0.026

LEE CP 1 0.026

LEE CS 1 0.026

LEE CT 1 0.026

LEE D 1 0.026

LEE DH 1 0.026

LEE DR 1 0.026

LEE DS 1 0.026

LEE EM 1 0.026

LEE GT 1 0.026

LEE HC 1 0.026

LEE HK 1 0.026

LEE HM 1 0.026

LEE JM 1 0.026

LEE JU 1 0.026

LEE KC 1 0.026

LEE KKH 1 0.026

LEE KM 1 0.026

LEE KSS 1 0.026

LEE KU 1 0.026

LEE KY 1 0.026

LEE ML 1 0.026

LEE NH 1 0.026

LEE PL 1 0.026

LEE T 1 0.026

LEE TY 1 0.026

LEE VWS 1 0.026

LEE WY 1 0.026

LEE YC 1 0.026

LEE YL 1 0.026

LEE YS 1 0.026

LEEMANS JC 1 0.026

LEESON-BEEVERS K 1 0.026

LEFAUCHEUR C 1 0.026

LEFEBVRE P 1 0.026

LEGIDO-QUIGLEY C 1 0.026

LEHTO M 1 0.026

LEI DM 1 0.026

LEI M 1 0.026

LEI S 1 0.026

LEI T 1 0.026

LEI YF 1 0.026

LEIRISALOREPO M 1 0.026

LEITE KRM 1 0.026

LEITER LA 1 0.026

LEJARS M 1 0.026

LEMAIRE SA 1 0.026

LEMAITRE V 1 0.026

LEMETAIS G 1 0.026

LEMOINE C 1 0.026

LEMTALSI T 1 0.026

LENG EN 1 0.026

LENNON R 1 0.026

LENSEN JFM 1 0.026

LENTSCH N 1 0.026

LEOF EB 1 0.026

LEON M 1 0.026

LEON-REYES G 1 0.026

LEONCINI F 1 0.026

LEONE DR 1 0.026

LEONE N 1 0.026

LEOPIZZI M 1 0.026

LEOW SE 1 0.026

LEPANTALO M 1 0.026

LEPENIES J 1 0.026

LEPOJARVI ES 1 0.026

LEPORE G 1 0.026

LERCH MM 1 0.026

LERNER SM 1 0.026

LERTORA JJL 1 0.026

LESCUYER P 1 0.026

LETO G 1 0.026

LEU SJ 1 0.026

LEUNG C 1 0.026

LEUNG CB 1 0.026

LEUNG G 1 0.026

LEUVENINK HGD 1 0.026

LEV N 1 0.026

LEVEDIANOS G 1 0.026

LEVELS JH 1 0.026

LEVEY AS 1 0.026

LEVIN NW 1 0.026

LEVINE DM 1 0.026

LEVINE JS 1 0.026

LEVTCHENKO E 1 0.026

LEVY D 1 0.026

LEVY J 1 0.026

LEVY MT 1 0.026

LEWANCZUK R 1 0.026

LEWIN E 1 0.026

LEWIN M 1 0.026

LEWIS EJ 1 0.026

LEWIS JB 1 0.026

LEWIS S 1 0.026

LEWIS SJ 1 0.026

LEWIS-JONES MS 1 0.026

LI AQ 1 0.026

LI BJ 1 0.026

LI CG 1 0.026

LI CJ 1 0.026

LI CM 1 0.026

LI CY 1 0.026

LI DH 1 0.026

LI DJ 1 0.026

LI DL 1 0.026

LI DT 1 0.026

LI DY 1 0.026

LI FA 1 0.026

LI FY 1 0.026

LI GD 1 0.026

LI GF 1 0.026

LI GL 1 0.026

LI GP 1 0.026

LI HB 1 0.026

LI HG 1 0.026

LI HN 1 0.026

LI HP 1 0.026

LI HQ 1 0.026

LI HW 1 0.026

LI JA 1 0.026

LI JB 1 0.026

LI JC 1 0.026

LI JD 1 0.026

LI JF 1 0.026

LI JR 1 0.026

LI KTP 1 0.026

LI KX 1 0.026

LI LJ 1 0.026

LI LK 1 0.026

LI LM 1 0.026

LI LQ 1 0.026

LI MH 1 0.026

LI MR 1 0.026

LI MS 1 0.026

LI MZ 1 0.026

LI NN 1 0.026

LI NQ 1 0.026

LI PJ 1 0.026

LI PP 1 0.026

LI QA 1 0.026

LI QB 1 0.026

LI QC 1 0.026

LI QF 1 0.026

LI QX 1 0.026

LI QY 1 0.026

LI RB 1 0.026

LI RL 1 0.026

LI RP 1 0.026

LI RT 1 0.026

LI RY 1 0.026

LI RZ 1 0.026

LI SN 1 0.026

LI SP 1 0.026

LI ST 1 0.026

LI SW 1 0.026

LI TH 1 0.026

LI WC 1 0.026

LI WL 1 0.026

LI WN 1 0.026

LI WQ 1 0.026

LI WS 1 0.026

LI WT 1 0.026

LI XC 1 0.026

LI XM 1 0.026

LI XN 1 0.026

LI YD 1 0.026

LI YF 1 0.026

LI YL 1 0.026

LI YN 1 0.026

LI YP 1 0.026

LI YW 1 0.026

LI YZ 1 0.026

LI ZB 1 0.026

LI ZC 1 0.026

LI ZD 1 0.026

LI ZN 1 0.026

LI ZP 1 0.026

LI ZQ 1 0.026

LI ZW 1 0.026

LI ZX 1 0.026

LIAN FL 1 0.026

LIAN H 1 0.026

LIAN M 1 0.026

LIAN QZ 1 0.026

LIAN Y 1 0.026

LIANG DD 1 0.026

LIANG DF 1 0.026

LIANG H 1 0.026

LIANG HK 1 0.026

LIANG HL 1 0.026

LIANG J 1 0.026

LIANG JX 1 0.026

LIANG KV 1 0.026

LIANG M 1 0.026

LIANG RF 1 0.026

LIANG SM 1 0.026

LIANG SS 1 0.026

LIANG ST 1 0.026

LIANG SY 1 0.026

LIANG WW 1 0.026

LIANG WX 1 0.026

LIANG XY 1 0.026

LIANG YZ 1 0.026

LIANG Z 1 0.026

LIAO C 1 0.026

LIAO D 1 0.026

LIAO GG 1 0.026

LIAO GN 1 0.026

LIAO H 1 0.026

LIAO J 1 0.026

LIAO LF 1 0.026

LIAO M 1 0.026

LIAO MC 1 0.026

LIAO MJ 1 0.026

LIAO MY 1 0.026

LIAO PY 1 0.026

LIAO RL 1 0.026

LIAO SZ 1 0.026

LIAO XH 1 0.026

LIAO YX 1 0.026

LIAO ZZ 1 0.026

LIBDIRI F 1 0.026

LIBIANTO R 1 0.026

LIBORIO AB 1 0.026

LICASTRO N 1 0.026

LICHT C 1 0.026

LICHTI-KAISER K 1 0.026

LIEBERTHAL W 1 0.026

LIEHR T 1 0.026

LIEN KR 1 0.026

LIEVERS E 1 0.026

LIFSHITZ T 1 0.026

LIGHEZAN D 1 0.026

LIGHTLE AR 1 0.026

LIM KHT 1 0.026

LIM SJ 1 0.026

LIM SK 1 0.026

LIMA ALM 1 0.026

LIMA DY 1 0.026

LIMAL N 1 0.026

LIMONE P 1 0.026

LIMPENS RWAL 1 0.026

LIN B 1 0.026

LIN C 1 0.026

LIN CE 1 0.026

LIN CR 1 0.026

LIN DL 1 0.026

LIN F 1 0.026

LIN FJ 1 0.026

LIN H 1 0.026

LIN HF 1 0.026

LIN HT 1 0.026

LIN HYH 1 0.026

LIN J 1 0.026

LIN JH 1 0.026

LIN KH 1 0.026

LIN KQ 1 0.026

LIN LR 1 0.026

LIN LT 1 0.026

LIN QB 1 0.026

LIN QZ 1 0.026

LIN SD 1 0.026

LIN SE 1 0.026

LIN SJ 1 0.026

LIN ST 1 0.026

LIN TJ 1 0.026

LIN TT 1 0.026

LIN WH 1 0.026

LIN WQ 1 0.026

LIN XF 1 0.026

LIN YF 1 0.026

LIN YT 1 0.026

LIN Z 1 0.026

LINCOLN TM 1 0.026

LINDBLOM RSJ 1 0.026

LINDHARDT M 1 0.026

LINDHOLM B 1 0.026

LINDOR KD 1 0.026

LINDSAY MP 1 0.026

LINDSAY RT 1 0.026

LINDSEY ML 1 0.026

LING YL 1 0.026

LINGVAY I 1 0.026

LINKE WA 1 0.026

LINZ D 1 0.026

LINZ W 1 0.026

LIPI L 1 0.026

LIPPAI R 1 0.026

LIPSANEN-NYMAN M 1 0.026

LIPSO K 1 0.026

LIPTAJ T 1 0.026

LISIK W 1 0.026

LITHOVIUS R 1 0.026

LITTLE MH 1 0.026

LIU BH 1 0.026

LIU BJ 1 0.026

LIU BY 1 0.026

LIU CH 1 0.026

LIU CL 1 0.026

LIU CP 1 0.026

LIU CW 1 0.026

LIU DX 1 0.026

LIU E 1 0.026

LIU FF 1 0.026

LIU FT 1 0.026

LIU GC 1 0.026

LIU GX 1 0.026

LIU HC 1 0.026

LIU HH 1 0.026

LIU HJ 1 0.026

LIU HL 1 0.026

LIU HW 1 0.026

LIU HX 1 0.026

LIU IM 1 0.026

LIU JB 1 0.026

LIU JK 1 0.026

LIU JM 1 0.026

LIU JY 1 0.026

LIU KH 1 0.026

LIU LH 1 0.026

LIU LY 1 0.026

LIU M 1 0.026

LIU MC 1 0.026

LIU MH 1 0.026

LIU MX 1 0.026

LIU NF 1 0.026

LIU NQ 1 0.026

LIU PL 1 0.026

LIU PP 1 0.026

LIU PY 1 0.026

LIU QF 1 0.026

LIU QZ 1 0.026

LIU R 1 0.026

LIU RB 1 0.026

LIU RC 1 0.026

LIU RG 1 0.026

LIU SL 1 0.026

LIU SQ 1 0.026

LIU WC 1 0.026

LIU WL 1 0.026

LIU WY 1 0.026

LIU XD 1 0.026

LIU XW 1 0.026

LIU YD 1 0.026

LIU YF 1 0.026

LIU YR 1 0.026

LIU YS 1 0.026

LIU ZD 1 0.026

LIU ZG 1 0.026

LIU ZM 1 0.026

LIU ZP 1 0.026

LIU ZQ 1 0.026

LIZAKOWSKI S 1 0.026

LJIMANI A 1 0.026

LJUTIC D 1 0.026

LLARENA M 1 0.026

LLOVERAS G 1 0.026

LLOYD C 1 0.026

LO A 1 0.026

LO KC 1 0.026

LO KY 1 0.026

LO MC 1 0.026

LO MW 1 0.026

LO RE V 1 0.026

LO SL 1 0.026

LOARCA-PINA G 1 0.026

LOBB RR 1 0.026

LOBERANT N 1 0.026

LOBOS C 1 0.026

LOCKWOOD CM 1 0.026

LOERA S 1 0.026

LOETSCHER P 1 0.026

LOEWE C 1 0.026

LOGANATHAN S 1 0.026

LOGANATHAN TS 1 0.026

LOGET P 1 0.026

LOHI J 1 0.026

LOHMEIER H 1 0.026

LOILER S 1 0.026

LOMBARDO G 1 0.026

LONG CL 1 0.026

LONG CM 1 0.026

LONG J 1 0.026

LONG K 1 0.026

LONG KZ 1 0.026

LONG YB 1 0.026

LONG YJ 1 0.026

LONG Z 1 0.026

LONGARETTI L 1 0.026

LONGERAS R 1 0.026

LONGHINI R 1 0.026

LONGO M 1 0.026

LONGPRE JM 1 0.026

LONIE E 1 0.026

LOOMBA R 1 0.026

LOPARDO G 1 0.026

LOPASCHUK GD 1 0.026

LOPES TG 1 0.026

LOPEZ CL 1 0.026

LOPEZ LRLY 1 0.026

LOPEZ V 1 0.026

LOPEZ-HERNANDEZ FJ 1 0.026

LOPEZ-MARIN L 1 0.026

LORENZEN J 1 0.026

LORENZI S 1 0.026

LORITO MC 1 0.026

LORTAT-JACOB H 1 0.026

LOSSO MH 1 0.026

LOSURDO P 1 0.026

LOTSPEICH DF 1 0.026

LOU DX 1 0.026

LOU HX 1 0.026

LOU JN 1 0.026

LOU JQ 1 0.026

LOU Z 1 0.026

LOURENCO AP 1 0.026

LOURO TM 1 0.026

LOVCIC V 1 0.026

LOZADA LGS 1 0.026

LOZANO M 1 0.026

LU CC 1 0.026

LU CS 1 0.026

LU CY 1 0.026

LU DW 1 0.026

LU F 1 0.026

LU FH 1 0.026

LU GY 1 0.026

LU H 1 0.026

LU HQ 1 0.026

LU JR 1 0.026

LU JS 1 0.026

LU JW 1 0.026

LU K 1 0.026

LU KM 1 0.026

LU KQ 1 0.026

LU LH 1 0.026

LU LL 1 0.026

LU LX 1 0.026

LU SM 1 0.026

LU SX 1 0.026

LU T 1 0.026

LU W 1 0.026

LU WC 1 0.026

LU WG 1 0.026

LU WH 1 0.026

LU WX 1 0.026

LU XH 1 0.026

LU XL 1 0.026

LU XZ 1 0.026

LU YF 1 0.026

LU YM 1 0.026

LU YW 1 0.026

LU YY 1 0.026

LU ZB 1 0.026

LU ZG 1 0.026

LU ZS 1 0.026

LU ZZ 1 0.026

LUAN PP 1 0.026

LUAN X 1 0.026

LUCANI B 1 0.026

LUCANTO C 1 0.026

LUCANTO MC 1 0.026

LUCAS GM 1 0.026

LUCAS R 1 0.026

LUCIO-CAZANA FJ 1 0.026

LUCIO-CAZANA J 1 0.026

LUCKHAUS C 1 0.026

LUCKOW B 1 0.026

LUENGO A 1 0.026

LUI SL 1 0.026

LUIS-LIMA S 1 0.026

LUK AOY 1 0.026

LUKASHEV ME 1 0.026

LUKATS B 1 0.026

LUKENDA V 1 0.026

LUM HZ 1 0.026

LUM-NAIHE K 1 0.026

LUMBERS ER 1 0.026

LUN A 1 0.026

LUND IK 1 0.026

LUND RJ 1 0.026

LUNDGREN JD 1 0.026

LUNT H 1 0.026

LUNZ J 1 0.026

LUO CL 1 0.026

LUO DL 1 0.026

LUO FL 1 0.026

LUO G 1 0.026

LUO HW 1 0.026

LUO HY 1 0.026

LUO K 1 0.026

LUO LB 1 0.026

LUO M 1 0.026

LUO QM 1 0.026

LUO QY 1 0.026

LUO SG 1 0.026

LUO SK 1 0.026

LUO SS 1 0.026

LUO T 1 0.026

LUO W 1 0.026

LUO XQ 1 0.026

LUO YK 1 0.026

LUO YM 1 0.026

LUO YS 1 0.026

LUO ZF 1 0.026

LUO ZJ 1 0.026

LUO ZM 1 0.026

LUPICA R 1 0.026

LUQMANI RA 1 0.026

LUSTER AD 1 0.026

LUTZ TA 1 0.026

LUZ MAM 1 0.026

LUZI L 1 0.026

LV B 1 0.026

LV CA 1 0.026

LV CY 1 0.026

LV FK 1 0.026

LV FP 1 0.026

LV GD 1 0.026

LV J 1 0.026

LV JC 1 0.026

LV JL 1 0.026

LV JR 1 0.026

LV JW 1 0.026

LV LN 1 0.026

LV R 1 0.026

LV TT 1 0.026

LV X 1 0.026

LV XW 1 0.026

LV Y 1 0.026

LV YC 1 0.026

LV YH 1 0.026

LV ZW 1 0.026

LYDEN E 1 0.026

LYMPANY PA 1 0.026

LYNCH MR 1 0.026

LYONS K 1 0.026

LYSIAK-SZYDLOWSKA W 1 0.026

LYTVYN Y 1 0.026

LYU BQ 1 0.026

LYU DY 1 0.026

LYU FP 1 0.026

LYU HY 1 0.026

LYU JX 1 0.026

LYU LK 1 0.026

LYU LL 1 0.026

LYU XF 1 0.026

LYV FP 1 0.026

M SH 1 0.026

MA A 1 0.026

MA B 1 0.026

MA BX 1 0.026

MA CG 1 0.026

MA CR 1 0.026

MA CY 1 0.026

MA DW 1 0.026

MA DX 1 0.026

MA FF 1 0.026

MA GR 1 0.026

MA H 1 0.026

MA HC 1 0.026

MA HJ 1 0.026

MA HX 1 0.026

MA JC 1 0.026

MA JM 1 0.026

MA JN 1 0.026

MA JY 1 0.026

MA KK 1 0.026

MA LL 1 0.026

MA LN 1 0.026

MA LQ 1 0.026

MA MF 1 0.026

MA MH 1 0.026

MA MKM 1 0.026

MA P 1 0.026

MA PL 1 0.026

MA QQ 1 0.026

MA RC 1 0.026

MA RF 1 0.026

MA RX 1 0.026

MA RZ 1 0.026

MA S 1 0.026

MA SC 1 0.026

MA SF 1 0.026

MA WJ 1 0.026

MA X 1 0.026

MA XQ 1 0.026

MA Y 1 0.026

MA YB 1 0.026

MA YJ 1 0.026

MA YM 1 0.026

MA YR 1 0.026

MA ZM 1 0.026

MA ZX 1 0.026

MA'AYAN A 1 0.026

MAALOUF R 1 0.026

MAAMRA M 1 0.026

MAAWAD M 1 0.026

MABILLE M 1 0.026

MAC-MOUNE LF 1 0.026

MACE ML 1 0.026

MACEDO E 1 0.026

MACEDO M 1 0.026

MACGREGOR AM 1 0.026

MACHADO FG 1 0.026

MACHADO JR 1 0.026

MACHADO M 1 0.026

MACHADO UF 1 0.026

MACHET MC 1 0.026

MACIA L 1 0.026

MACIA M 1 0.026

MACIEJEWSKI BS 1 0.026

MACIEL FR 1 0.026

MACIEL TT 1 0.026

MACISAAC RJ 1 0.026

MACKAY CR 1 0.026

MACKENSENHAEN S 1 0.026

MACKEY RH 1 0.026

MACKIE K 1 0.026

MACKRAJ I 1 0.026

MACNAUGHT G 1 0.026

MACOSKA JA 1 0.026

MADDEN VJ 1 0.026

MADDIPATI KR 1 0.026

MADERDRUT JL 1 0.026

MADERO M 1 0.026

MADESH M 1 0.026

MADHURAPANTULA RS 1 0.026

MADORE F 1 0.026

MADRIGAL-MATUTE J 1 0.026

MADSEN K 1 0.026

MAEDA K 1 0.026

MAEDA M 1 0.026

MAEDA S 1 0.026

MAEJIMA I 1 0.026

MAESAKA J 1 0.026

MAESATO K 1 0.026

MAESATOO K 1 0.026

MAESTRONI A 1 0.026

MAFI A 1 0.026

MAGALHAES JA 1 0.026

MAGANA S 1 0.026

MAGARD D 1 0.026

MAGEE C 1 0.026

MAGILNICK N 1 0.026

MAGISTRONI R 1 0.026

MAGNANI C 1 0.026

MAGNI F 1 0.026

MAGRO CM 1 0.026

MAGUIRE J 1 0.026

MAHADEVA S 1 0.026

MAHADY SE 1 0.026

MAHAPATRA S 1 0.026

MAHESHWARI M 1 0.026

MAHFOUD F 1 0.026

MAHIMKAR R 1 0.026

MAHJOUB N 1 0.026

MAHMOOD A 1 0.026

MAHMOUD AM 1 0.026

MAHNKEN AH 1 0.026

MAHON JL 1 0.026

MAI YF 1 0.026

MAILLARD-LEFEBVRE H 1 0.026

MAIMAITIYIMING H 1 0.026

MAISEL A 1 0.026

MAJOR AM 1 0.026

MAJOR M 1 0.026

MAK SK 1 0.026

MAK TSK 1 0.026

MAKINEN VP 1 0.026

MAKINO Y 1 0.026

MAKITA Y 1 0.026

MAKRIDAKIS M 1 0.026

MAKSIMOWSKI N 1 0.026

MAKUUCHI M 1 0.026

MALAESCU DG 1 0.026

MALAKAUSKAS SM 1 0.026

MALHOTRA A 1 0.026

MALHOTRA K 1 0.026

MALIK S 1 0.026

MALINSKA H 1 0.026

MALKOC E 1 0.026

MALL G 1 0.026

MALLELA SK 1 0.026

MALLIPATTU SK 1 0.026

MALLOLAS J 1 0.026

MALUF DG 1 0.026

MAMAS MA 1 0.026

MAN Y 1 0.026

MAN YL 1 0.026

MANABE I 1 0.026

MANABE M 1 0.026

MANDARIM-DE-LACERDA CA 1 0.026

MANDAVIA CH 1 0.026

MANDELBROT DA 1 0.026

MANDET C 1 0.026

MANDORFER M 1 0.026

MANDRUP-POULSEN T 1 0.026

MANGI AA 1 0.026

MANHIANI M 1 0.026

MANIERO C 1 0.026

MANIGRASSO M 1 0.026

MANLEY P 1 0.026

MANN D 1 0.026

MANN E 1 0.026

MANN JFE 1 0.026

MANN K 1 0.026

MANNA K 1 0.026

MANNING RD 1 0.026

MANNON RB 1 0.026

MANOHARAN M 1 0.026

MANOJKUMAR R 1 0.026

MANOLIS AA 1 0.026

MANOLIS AS 1 0.026

MANOLIS TA 1 0.026

MANOME Y 1 0.026

MANONELLES A 1 0.026

MANRIQUE-ACEVEDO C 1 0.026

MANSIER P 1 0.026

MANSOUR SG 1 0.026

MANSOURI E 1 0.026

MANTI R 1 0.026

MANTZARIS I 1 0.026

MANTZOROS CS 1 0.026

MANZ F 1 0.026

MAO DD 1 0.026

MAO GX 1 0.026

MAO JC 1 0.026

MAO JF 1 0.026

MAO LL 1 0.026

MAO M 1 0.026

MAO Q 1 0.026

MAO RW 1 0.026

MAO S 1 0.026

MAO W 1 0.026

MAO X 1 0.026

MAO XY 1 0.026

MAOUCHE B 1 0.026

MAQUIGUSSA E 1 0.026

MARANO G 1 0.026

MARBURY DC 1 0.026

MARCELLIN P 1 0.026

MARCH KL 1 0.026

MARCHEQUE J 1 0.026

MARCO S 1 0.026

MARCOVECCHIO ML 1 0.026

MARCY TR 1 0.026

MARGETTS P 1 0.026

MARGOLIN S 1 0.026

MARIAGER CO 1 0.026

MARIAKUTTIKAN J 1 0.026

MARIANI LH 1 0.026

MARIN JCO 1 0.026

MARIN R 1 0.026

MARIN TA 1 0.026

MARINAKI S 1 0.026

MARINO S 1 0.026

MARIOTTONI B 1 0.026

MARK M 1 0.026

MARKHAM A 1 0.026

MARKIEWICZ M 1 0.026

MARKOVA I 1 0.026

MARKOWITZ G 1 0.026

MARKUSSEN S 1 0.026

MARNEY AM 1 0.026

MAROFKA F 1 0.026

MAROTTA F 1 0.026

MARQUEZ-EXPOSITO L 1 0.026

MARRONI NP 1 0.026

MARS WM 1 0.026

MARSAL D 1 0.026

MARSAN NA 1 0.026

MARSCHNER JA 1 0.026

MARSDEN PA 1 0.026

MARSHALL RP 1 0.026

MARSZALEK R 1 0.026

MARTARELLO L 1 0.026

MARTENS PP 1 0.026

MARTENSSON G 1 0.026

MARTIN CA 1 0.026

MARTIN L 1 0.026

MARTIN PY 1 0.026

MARTIN TN 1 0.026

MARTIN WP 1 0.026

MARTIN-CASTILLO B 1 0.026

MARTIN-MATEOS RM 1 0.026

MARTIN-SANCHEZ D 1 0.026

MARTIN-VENTURA JL 1 0.026

MARTINEZ F 1 0.026

MARTINEZ JR 1 0.026

MARTINEZ RV 1 0.026

MARTINEZ-CASTELAO A 1 0.026

MARTINEZ-LEMUS L 1 0.026

MARTINEZ-REYES C 1 0.026

MARTINEZ-VASQUEZ D 1 0.026

MARTINEZMALDONADO M 1 0.026

MARTINOVIC J 1 0.026

MARUBASHI S 1 0.026

MARUHASHI T 1 0.026

MARUYAMA H 1 0.026

MARUYAMA N 1 0.026

MARUYAMA T 1 0.026

MARWAY M 1 0.026

MARZI A 1 0.026

MAS VR 1 0.026

MASALA A 1 0.026

MASAOUTIS C 1 0.026

MASCARENHAS S 1 0.026

MASCARENHAS-MELO F 1 0.026

MASCHERBAUER J 1 0.026

MASCHIO G 1 0.026

MASELLA C 1 0.026

MASOUDI FA 1 0.026

MASOUMI A 1 0.026

MASSRY SG 1 0.026

MASTROROBERTO P 1 0.026

MASUDA K 1 0.026

MASUDA T 1 0.026

MASUI Y 1 0.026

MASUOKA N 1 0.026

MASUYAMA T 1 0.026

MATAFOME PN 1 0.026

MATAR RN 1 0.026

MATAS A 1 0.026

MATHAROO K 1 0.026

MATHENGE N 1 0.026

MATHER A 1 0.026

MATHEW A 1 0.026

MATHEW AB 1 0.026

MATHEW AV 1 0.026

MATHEW S 1 0.026

MATHIAS PCD 1 0.026

MATHIESEN ER 1 0.026

MATHIESON P 1 0.026

MATHIEU P 1 0.026

MATHIEU T 1 0.026

MATIMOTO RL 1 0.026

MATKAR PN 1 0.026

MATONDO A 1 0.026

MATSUBARA H 1 0.026

MATSUBARA T 1 0.026

MATSUDA H 1 0.026

MATSUDA J 1 0.026

MATSUDA M 1 0.026

MATSUDA T 1 0.026

MATSUFUJI S 1 0.026

MATSUHASHI T 1 0.026

MATSUI F 1 0.026

MATSUI I 1 0.026

MATSUI K 1 0.026

MATSUI Y 1 0.026

MATSUKAWA Y 1 0.026

MATSUMOTO H 1 0.026

MATSUMOTO Y 1 0.026

MATSUNAGA Y 1 0.026

MATSUO K 1 0.026

MATSUO T 1 0.026

MATSUOKA H 1 0.026

MATSUSHIMA K 1 0.026

MATSUSHITA K 1 0.026

MATSUSHITA M 1 0.026

MATSUSHITA Y 1 0.026

MATSUYAMA T 1 0.026

MATSUZAWA Y 1 0.026

MATTAR AL 1 0.026

MATTHAY MA 1 0.026

MATTIAZZI A 1 0.026

MATTILA I 1 0.026

MATTINZOLI D 1 0.026

MAU SC 1 0.026

MAUER SM 1 0.026

MAURER MM 1 0.026

MAURER TS 1 0.026

MAURICE MJ 1 0.026

MAURICIO ADV 1 0.026

MAURY JM 1 0.026

MAUSS S 1 0.026

MAY E 1 0.026

MAYER-DAVIS EJ 1 0.026

MAYER-EZELL R 1 0.026

MAYERS RM 1 0.026

MAYES JT 1 0.026

MAYMON E 1 0.026

MAYNARD M 1 0.026

MAYOUX E 1 0.026

MAYYAS F 1 0.026

MAZAGOVA M 1 0.026

MAZARIEGOS GV 1 0.026

MAZUR A 1 0.026

MAZUREK J 1 0.026

MAZZA M 1 0.026

MAZZOLA A 1 0.026

MAZZONE P 1 0.026

MAZZOTTI A 1 0.026

MCCAFFREY TA 1 0.026

MCCANN GP 1 0.026

MCCARTHY DA 1 0.026

MCCLARTY S 1 0.026

MCCORMACK J 1 0.026

MCCORMICK JA 1 0.026

MCCOY RG 1 0.026

MCCRANN DJ 1 0.026

MCCUE P 1 0.026

MCCULLY B 1 0.026

MCCUNE WJ 1 0.026

MCDERMOTT B 1 0.026

MCDONALD S 1 0.026

MCDONNELL KP 1 0.026

MCEVOY C 1 0.026

MCFANN KK 1 0.026

MCFARLANE PA 1 0.026

MCGAHAN G 1 0.026

MCGARAUGHTY SP 1 0.026

MCGILL JB 1 0.026

MCGREGOR B 1 0.026

MCGREGOR J 1 0.026

MCKAY DW 1 0.026

MCKELVEY M 1 0.026

MCKEON C 1 0.026

MCKEON D 1 0.026

MCKIE PM 1 0.026

MCLEAN D 1 0.026

MCLENNAN SV 1 0.026

MCMAHON GM 1 0.026

MCMANAMAN JL 1 0.026

MCNEIL K 1 0.026

MCNEILL H 1 0.026

MCPHERSON KC 1 0.026

MCQUARRIE EP 1 0.026

MCROBERT A 1 0.026

MEAD PA 1 0.026

MEDA I 1 0.026

MEDEI E 1 0.026

MEDINA-NAVARRO R 1 0.026

MEDINA-PESTANA JO 1 0.026

MEEHAN DT 1 0.026

MEEK RL 1 0.026

MEEKS CJ 1 0.026

MEERMEIER NP 1 0.026

MEGA C 1 0.026

MEGYESI JK 1 0.026

MEHMETI F 1 0.026

MEHTA A 1 0.026

MEHTA JL 1 0.026

MEHTA N 1 0.026

MEHTA RA 1 0.026

MEHTA RL 1 0.026

MEI M 1 0.026

MEIKLE PJ 1 0.026

MEILEI H 1 0.026

MEINICKE T 1 0.026

MEISE D 1 0.026

MEISE N 1 0.026

MEISS L 1 0.026

MEJIA-RODRIGUEZ O 1 0.026

MEKAHLI D 1 0.026

MELA MG 1 0.026

MELANDER O 1 0.026

MELCHIORRE K 1 0.026

MELENHORST WBWH 1 0.026

MELGAR A 1 0.026

MELHEM M 1 0.026

MELIAMBRO K 1 0.026

MELILLO F 1 0.026

MELISSOPOULOU M 1 0.026

MELITA H 1 0.026

MELLO A 1 0.026

MELLONI E 1 0.026

MEMMO A 1 0.026

MEMON KK 1 0.026

MENDES GEF 1 0.026

MENDEZ AJ 1 0.026

MENDEZ JB 1 0.026

MENDICHOVSZKY IA 1 0.026

MENDIZABAL M 1 0.026

MENDONCA LD 1 0.026

MENE P 1 0.026

MENEELY J 1 0.026

MENENDEZ JA 1 0.026

MENG FW 1 0.026

MENG FY 1 0.026

MENG JZ 1 0.026

MENG P 1 0.026

MENG QF 1 0.026

MENG S 1 0.026

MENG ZW 1 0.026

MENGHI V 1 0.026

MENGQIQIGE BM 1 0.026

MENN-JOSEPHY H 1 0.026

MENON MC 1 0.026

MENSAH FK 1 0.026

MENSHIKH A 1 0.026

MENTZ RJ 1 0.026

MENZIES RI 1 0.026

MEO SA 1 0.026

MERA K 1 0.026

MERAN S 1 0.026

MERCIER-ZUBER A 1 0.026

MERCURE C 1 0.026

MEREGALLI C 1 0.026

MERKEL PA 1 0.026

MERKUS D 1 0.026

MERSCHER S 1 0.026

MERTENS P 1 0.026

MERTENS PR 1 0.026

MESSA P 1 0.026

MESSCHENDORP AL 1 0.026

MESSIKA-ZEITOUN D 1 0.026

MESSING RO 1 0.026

MESTDAGH P 1 0.026

METRA M 1 0.026

METREVELI N 1 0.026

METREVELI NS 1 0.026

METZGER R 1 0.026

MEURISSE N 1 0.026

MEYER M 1 0.026

MEYERS KEC 1 0.026

MEZZA T 1 0.026

MI QS 1 0.026

MI XH 1 0.026

MIAILHES P 1 0.026

MIAN B 1 0.026

MIAN BM 1 0.026

MIAO H 1 0.026

MIAO HH 1 0.026

MIAO HZ 1 0.026

MIAO JF 1 0.026

MIAO JH 1 0.026

MIAO XJ 1 0.026

MIAO YH 1 0.026

MICHAEL A 1 0.026

MICHAEL DR 1 0.026

MICHAELIS OE 1 0.026

MICHALAK M 1 0.026

MICHALOPOULOS GK 1 0.026

MICHELLI A 1 0.026

MICHLI E 1 0.026

MICHOS ED 1 0.026

MICKELER E 1 0.026

MICLAUS G 1 0.026

MIDDLEDITCH M 1 0.026

MIEHE B 1 0.026

MIETTINEN JA 1 0.026

MIFFLIN TE 1 0.026

MIGALA M 1 0.026

MIGLIORE F 1 0.026

MIGLIORE N 1 0.026

MIGUEL M 1 0.026

MIHANFAR A 1 0.026

MIKOLASEVIC I 1 0.026

MILAN G 1 0.026

MILANI D 1 0.026

MILENKOVIC S 1 0.026

MILENKOVIC T 1 0.026

MILIC S 1 0.026

MILLER JD 1 0.026

MILLER R 1 0.026

MILLER RA 1 0.026

MILLER SS 1 0.026

MILLIEZ P 1 0.026

MILO G 1 0.026

MILOH T 1 0.026

MILOT A 1 0.026

MILUTINOVIC N 1 0.026

MIMURA I 1 0.026

MIN CY 1 0.026

MIN JY 1 0.026

MIN KD 1 0.026

MIN XQ 1 0.026

MIN YI 1 0.026

MIN YL 1 0.026

MINAMI S 1 0.026

MINDIKOGLU AL 1 0.026

MINEGISHI N 1 0.026

MINELLI E 1 0.026

MINNERUP J 1 0.026

MINTON JAL 1 0.026

MION F 1 0.026

MIQUELESTORENA-STANDLEY E 1 0.026

MIR M 1 0.026

MIRANDA AS 1 0.026

MIRANDA-SILVA D 1 0.026

MIREL DB 1 0.026

MIRHOSSEINI N 1 0.026

MIRIC G 1 0.026

MIRKOVIC K 1 0.026

MIROCHNIK Y 1 0.026

MIRZA DF 1 0.026

MIRZOYAN K 1 0.026

MISAKI T 1 0.026

MISHRA A 1 0.026

MISHRA N 1 0.026

MISHRA S 1 0.026

MISRA PS 1 0.026

MISRA S 1 0.026

MITARAI T 1 0.026

MITCHELL D 1 0.026

MITOBE Y 1 0.026

MITROFANOVA A 1 0.026

MITSUI T 1 0.026

MITU GM 1 0.026

MIURA K 1 0.026

MIURA M 1 0.026

MIURA Y 1 0.026

MIYABE M 1 0.026

MIYACHI Y 1 0.026

MIYAGAWA S 1 0.026

MIYAGAWA T 1 0.026

MIYAJIMA A 1 0.026

MIYAJIMA H 1 0.026

MIYAJIMA K 1 0.026

MIYAKE H 1 0.026

MIYAKE Y 1 0.026

MIYAMOTO L 1 0.026

MIYASHIRO J 1 0.026

MIYASHITA S 1 0.026

MIYATA N 1 0.026

MIYAUCHI M 1 0.026

MIYAZAKI I 1 0.026

MIYAZAKI Y 1 0.026

MIYOSHI I 1 0.026

MIYOSHI S 1 0.026

MIZOBUCHI M 1 0.026

MIZRACHI EB 1 0.026

MIZRAHI J 1 0.026

MIZUE Y 1 0.026

MIZUGUCHI Y 1 0.026

MIZUKI F 1 0.026

MIZUKOSHI F 1 0.026

MIZUNO N 1 0.026

MIZUNO-HORIKAWA Y 1 0.026

MIZUNUMA Y 1 0.026

MIZUTANI M 1 0.026

MIZUTANI S 1 0.026

MIZUTANI T 1 0.026

MO HY 1 0.026

MO ZH 1 0.026

MOBASHERI A 1 0.026

MOBINI M 1 0.026

MODENA F 1 0.026

MODI A 1 0.026

MODI AC 1 0.026

MOE G 1 0.026

MOE GW 1 0.026

MOECKEL GW 1 0.026

MOELLER MJ 1 0.026

MOERMAN A 1 0.026

MOFID A 1 0.026

MOGENSEN CK 1 0.026

MOGYOROSI A 1 0.026

MOHAMED HA 1 0.026

MOHAMED M 1 0.026

MOHAMED RH 1 0.026

MOHAMMAD T 1 0.026

MOHAMMADI M 1 0.026

MOHAMMADI S 1 0.026

MOHAMMED AK 1 0.026

MOHAN A 1 0.026

MOHAN T 1 0.026

MOHANKA M 1 0.026

MOHRING K 1 0.026

MOHSEN TA 1 0.026

MOISEYEV G 1 0.026

MOKASHI A 1 0.026

MOKHTARZADEH A 1 0.026

MOLINA J 1 0.026

MOLINA-VAN DEN BOSCH M 1 0.026

MOLINEAUX C 1 0.026

MOLITCH ME 1 0.026

MOLL AG 1 0.026

MOLLER C 1 0.026

MOLLESTON JP 1 0.026

MOLLET G 1 0.026

MOLLSTEN A 1 0.026

MOLMER M 1 0.026

MOLNAR A 1 0.026

MOLNAR GA 1 0.026

MOLNE J 1 0.026

MONAGHAN S 1 0.026

MONBALIU D 1 0.026

MONDEN M 1 0.026

MONETTI M 1 0.026

MONGA SP 1 0.026

MONIER F 1 0.026

MONKAWA T 1 0.026

MONNERAT-CAHLI G 1 0.026

MONNIER VM 1 0.026

MONNNO I 1 0.026

MONSARRAT B 1 0.026

MONTAGNINO G 1 0.026

MONTANARI CC 1 0.026

MONTANYA X 1 0.026

MONTEILLET L 1 0.026

MONTEIRO EC 1 0.026

MONTEIRO MB 1 0.026

MONTENEGRO LD 1 0.026

MONTERO A 1 0.026

MONTERO M 1 0.026

MONTERO RM 1 0.026

MONTERO-DUARTE K 1 0.026

MONTES ML 1 0.026

MONTEZANO A 1 0.026

MONTEZANO AC 1 0.026

MONTI S 1 0.026

MONTINI B 1 0.026

MOODLEY K 1 0.026

MOODY WE 1 0.026

MOON H 1 0.026

MOON HS 1 0.026

MOON SD 1 0.026

MOON SJ 1 0.026

MOOPPAN UMM 1 0.026

MOORE JD 1 0.026

MOORMAN D 1 0.026

MORA J 1 0.026

MORA-GUTIERREZ JM 1 0.026

MORAD MA 1 0.026

MORADI N 1 0.026

MORALES J 1 0.026

MORALES JM 1 0.026

MORALES MM 1 0.026

MORALES XA 1 0.026

MORALES-BLANHIR J 1 0.026

MORALES-ROMERO J 1 0.026

MORALES-RULL JL 1 0.026

MORATO M 1 0.026

MORDWINKIN NM 1 0.026

MORE R 1 0.026

MOREAU P 1 0.026

MOREAU R 1 0.026

MOREIRA MCV 1 0.026

MOREIRA-GONCALVES D 1 0.026

MOREL D 1 0.026

MOREL F 1 0.026

MORELLO J 1 0.026

MORELON E 1 0.026

MORELOS-GUZMAN M 1 0.026

MORENO-MANZANO V 1 0.026

MORESCO RN 1 0.026

MORETH K 1 0.026

MORGAN PE 1 0.026

MORGAN SH 1 0.026

MORI H 1 0.026

MORI J 1 0.026

MORI KP 1 0.026

MORI TA 1 0.026

MORI Y 1 0.026

MORIGI M 1 0.026

MORIGUCHI M 1 0.026

MORIGUCHI T 1 0.026

MORIKAWA S 1 0.026

MORIKAWA Y 1 0.026

MORIMOTO M 1 0.026

MORIMOTO S 1 0.026

MORINAGA H 1 0.026

MORINAGA Y 1 0.026

MORINE MJ 1 0.026

MORISHITA Y 1 0.026

MORITA M 1 0.026

MORITZ MJ 1 0.026

MORIUCHI A 1 0.026

MORIZANE R 1 0.026

MORIZZI J 1 0.026

MORIZZO C 1 0.026

MORLEY K 1 0.026

MORMINA E 1 0.026

MORNEX JF 1 0.026

MOROKATA T 1 0.026

MORRELL G 1 0.026

MORRELL GR 1 0.026

MORRISON MC 1 0.026

MORROW DA 1 0.026

MORSER J 1 0.026

MORTON AM 1 0.026

MORVAY Z 1 0.026

MOSENZON O 1 0.026

MOSHAGE H 1 0.026

MOSHAL KS 1 0.026

MOSNIER-PUDAR H 1 0.026

MOSTAFA O 1 0.026

MOSTOFA M 1 0.026

MOTA A 1 0.026

MOTA E 1 0.026

MOTAMED K 1 0.026

MOTOTANI Y 1 0.026

MOTRAPU M 1 0.026

MOTT R 1 0.026

MOU YR 1 0.026

MOU ZX 1 0.026

MOULD NN 1 0.026

MOULIN B 1 0.026

MOUNTS WM 1 0.026

MOURO MG 1 0.026

MOUSSA K 1 0.026

MOYER-MILEUR LJ 1 0.026

MOYERS JJ 1 0.026

MRAZEK C 1 0.026

MU J 1 0.026

MU L 1 0.026

MU SH 1 0.026

MU YT 1 0.026

MUBARAK MS 1 0.026

MUCHA O 1 0.026

MUCHANETAKUBARA EC 1 0.026

MUCINO MJ 1 0.026

MUDALIAR H 1 0.026

MUDALIAR S 1 0.026

MUEHLENBERG P 1 0.026

MUELLER T 1 0.026

MUGELLINI A 1 0.026

MUGESH G 1 0.026

MUHAMMAD RS 1 0.026

MUHE AM 1 0.026

MUHLBERG W 1 0.026

MUHLFELD A 1 0.026

MUISE ES 1 0.026

MUJAJ B 1 0.026

MUKAE H 1 0.026

MUKAE T 1 0.026

MUKAI K 1 0.026

MUKAIDA N 1 0.026

MUKAMAL KJ 1 0.026

MUKASA K 1 0.026

MUKDSI JH 1 0.026

MUKHI D 1 0.026

MUKHOPADHYAY M 1 0.026

MUKUNO A 1 0.026

MULATERO P 1 0.026

MULAY SR 1 0.026

MULDER S 1 0.026

MULE G 1 0.026

MULLAN AF 1 0.026

MULLEN Y 1 0.026

MULLER C 1 0.026

MULLER D 1 0.026

MULLER G 1 0.026

MULLER J 1 0.026

MULLER RU 1 0.026

MULLER V 1 0.026

MULLER-KREBS S 1 0.026

MULLER-WIEFEL DE 1 0.026

MULLINS J 1 0.026

MULLOY B 1 0.026

MULRONEY S 1 0.026

MULRONEY SE 1 0.026

MULVIHILL JJ 1 0.026

MUMMERY CL 1 0.026

MUNGAMURI SK 1 0.026

MUNIR MB 1 0.026

MUNIR SM 1 0.026

MUNKONDA MN 1 0.026

MUNNICH A 1 0.026

MUNOZ D 1 0.026

MUNOZ K 1 0.026

MUNOZ RM 1 0.026

MUNTEANU M 1 0.026

MUNTENDAM P 1 0.026

MUNTER K 1 0.026

MUNUSAMY S 1 0.026

MUORAH M 1 0.026

MURAD NAA 1 0.026

MURAI N 1 0.026

MURAKAMI K 1 0.026

MURAKAMI T 1 0.026

MURASE T 1 0.026

MURAY S 1 0.026

MURIITHI AK 1 0.026

MUROHARA T 1 0.026

MUROYA Y 1 0.026

MURPHY B 1 0.026

MURPHY BJ 1 0.026

MURPHY GJ 1 0.026

MURPHY MP 1 0.026

MURPHY P 1 0.026

MURPHY PJ 1 0.026

MURPHY S 1 0.026

MURRAY AJ 1 0.026

MURRAY AR 1 0.026

MURRAY B 1 0.026

MURRAY PT 1 0.026

MURRAY-MCINTOSH RP 1 0.026

MURTHI P 1 0.026

MUSETTI C 1 0.026

MUSI N 1 0.026

MUSO E 1 0.026

MUSTAPHA NRN 1 0.026

MUSTONEN J 1 0.026

MUTCHNICK M 1 0.026

MUTCHNICK S 1 0.026

MUTIG K 1 0.026

MUTIMER D 1 0.026

MUTIN M 1 0.026

MUTNURI S 1 0.026

MYARA I 1 0.026

MYCHALECKYJ JC 1 0.026

MYERS AK 1 0.026

MYERS MG 1 0.026

MYHRE S 1 0.026

MYLLYMAKI J 1 0.026

MYOJO K 1 0.026

MYRDA K 1 0.026

MYSIAK A 1 0.026

NA KY 1 0.026

NABOKOV A 1 0.026

NADASDY G 1 0.026

NADASDY T 1 0.026

NADKARNI GN 1 0.026

NAEEM H 1 0.026

NAFAR M 1 0.026

NAGAISHI K 1 0.026

NAGAO S 1 0.026

NAGAO T 1 0.026

NAGAOKA Y 1 0.026

NAGASAKA S 1 0.026

NAGASE M 1 0.026

NAGASU H 1 0.026

NAGATA D 1 0.026

NAGATA N 1 0.026

NAGAYA H 1 0.026

NAGINENI VV 1 0.026

NAGOYA T 1 0.026

NAGTEGAAL EJ 1 0.026

NAGY T 1 0.026

NAHAR L 1 0.026

NAHMIAS Y 1 0.026

NAHUELPAN Y 1 0.026

NAICKER S 1 0.026

NAIK G 1 0.026

NAIR M 1 0.026

NAITO T 1 0.026

NAJAFI M 1 0.026

NAJJAR SM 1 0.026

NAJJAR SS 1 0.026

NAKABAYASHI M 1 0.026

NAKAGAWA A 1 0.026

NAKAGAWA F 1 0.026

NAKAGAWA N 1 0.026

NAKAJIMA A 1 0.026

NAKAJIMA S 1 0.026

NAKAJIMA Y 1 0.026

NAKAJOU K 1 0.026

NAKAMURA H 1 0.026

NAKAMURA K 1 0.026

NAKAMURA M 1 0.026

NAKAMURA N 1 0.026

NAKAMUTA M 1 0.026

NAKANISHI T 1 0.026

NAKANO M 1 0.026

NAKAO T 1 0.026

NAKATSU Y 1 0.026

NAKAYAMA K 1 0.026

NAKAZATO Y 1 0.026

NAKHLEH RE 1 0.026

NAKHOUL F 1 0.026

NAKHOUL N 1 0.026

NAKHOUL R 1 0.026

NAKO H 1 0.026

NAKOPOULOU L 1 0.026

NAMBA T 1 0.026

NAMOVIC M 1 0.026

NAMOVIC MT 1 0.026

NAN H 1 0.026

NANDY A 1 0.026

NANKERVIS A 1 0.026

NANKIVELL BJ 1 0.026

NANO R 1 0.026

NARCISO-SCHIAVON JL 1 0.026

NARDI E 1 0.026

NARGESI AA 1 0.026

NARIAI T 1 0.026

NARITA Y 1 0.026

NART D 1 0.026

NARULA S 1 0.026

NARUSE M 1 0.026

NARUSE T 1 0.026

NASH DM 1 0.026

NASH K 1 0.026

NASH KL 1 0.026

NASHAWI M 1 0.026

NASRALLAH MP 1 0.026

NASRI P 1 0.026

NASS KJ 1 0.026

NASSAR MS 1 0.026

NASSIF J 1 0.026

NATALI MRM 1 0.026

NATH MC 1 0.026

NATIV O 1 0.026

NAUFAL M 1 0.026

NAUTA FL 1 0.026

NAVARRO-GONZALEZ JF 1 0.026

NAVIS GJ 1 0.026

NAWROTH PP 1 0.026

NAYIR A 1 0.026

NAYLOR P 1 0.026

NAZAREWICZ RR 1 0.026

NDIBALEMA AR 1 0.026

NEAR K 1 0.026

NEBULONI M 1 0.026

NEELAMEGAM K 1 0.026

NEELY BA 1 0.026

NEFF TB 1 0.026

NEGISHI K 1 0.026

NEGRI AL 1 0.026

NEGRO F 1 0.026

NEHME A 1 0.026

NEHME J 1 0.026

NEJAD-DEHBASHI F 1 0.026

NELSON J 1 0.026

NELSON PJ 1 0.026

NEMA V 1 0.026

NEMMAR A 1 0.026

NEPOMUCKA K 1 0.026

NERENZ DR 1 0.026

NERUP J 1 0.026

NESBIT MA 1 0.026

NETO OMV 1 0.026

NEUBERGER J 1 0.026

NEUHOFER W 1 0.026

NEUMILLER JJ 1 0.026

NEVES CQ 1 0.026

NEVILLE RD 1 0.026

NEWSOME PN 1 0.026

NEWTON E 1 0.026

NEY A 1 0.026

NG KF 1 0.026

NGA HS 1 0.026

NGO TT 1 0.026

NGOWI EE 1 0.026

NGUAN CYC 1 0.026

NGUYEN C 1 0.026

NGUYEN D 1 0.026

NGUYEN ITN 1 0.026

NGUYEN L 1 0.026

NGUYEN TK 1 0.026

NGUYEN TT 1 0.026

NI B 1 0.026

NI CL 1 0.026

NI DN 1 0.026

NI HF 1 0.026

NI HL 1 0.026

NI L 1 0.026

NI LS 1 0.026

NI PH 1 0.026

NI WC 1 0.026

NI XJ 1 0.026

NI YQ 1 0.026

NIAN H 1 0.026

NIAUDET P 1 0.026

NICHOLSON ML 1 0.026

NICK H 1 0.026

NICOLUCCI A 1 0.026

NICOSIA RF 1 0.026

NIE CJ 1 0.026

NIE S 1 0.026

NIE W 1 0.026

NIE YX 1 0.026

NIE ZY 1 0.026

NIELSEN CB 1 0.026

NIELSEN CH 1 0.026

NIELSEN FS 1 0.026

NIELSEN LB 1 0.026

NIELSEN SH 1 0.026

NIEMANN G 1 0.026

NIEMANN M 1 0.026

NIESSEN HWM 1 0.026

NIEUWLAND R 1 0.026

NIGRO M 1 0.026

NIHEI H 1 0.026

NIIMI A 1 0.026

NIIMURA F 1 0.026

NIIOKA T 1 0.026

NIIZUMA S 1 0.026

NIKI R 1 0.026

NIKKEL A 1 0.026

NIKOLAKOPOULOU A 1 0.026

NIKOLENKO V 1 0.026

NILES D 1 0.026

NILSSON F 1 0.026

NILSSON J 1 0.026

NIMMERJAHN F 1 0.026

NING C 1 0.026

NING J 1 0.026

NING Y 1 0.026

NIO Y 1 0.026

NIO-KOBAYASHI J 1 0.026

NIPA NS 1 0.026

NIRANJAN T 1 0.026

NISHAD R 1 0.026

NISHIDA C 1 0.026

NISHIDA M 1 0.026

NISHIJIMA F 1 0.026

NISHIKAWA T 1 0.026

NISHIKIMI T 1 0.026

NISHIMATSU H 1 0.026

NISHIMURA F 1 0.026

NISHIMURA H 1 0.026

NISHIMURA M 1 0.026

NISHINA H 1 0.026

NISHINA PM 1 0.026

NISHINO Y 1 0.026

NITSCHE C 1 0.026

NITSCHMANN E 1 0.026

NIU C 1 0.026

NIU CL 1 0.026

NIU CS 1 0.026

NIU HL 1 0.026

NIU HM 1 0.026

NIU HS 1 0.026

NIU JL 1 0.026

NIU JM 1 0.026

NIU L 1 0.026

NIU Q 1 0.026

NIU XY 1 0.026

NIWA Y 1 0.026

NOBAKHTHAGHIGHI N 1 0.026

NOBLE EP 1 0.026

NOBLE S 1 0.026

NODA K 1 0.026

NODA M 1 0.026

NODA S 1 0.026

NOGUEIRA A 1 0.026

NOGUEIRA GB 1 0.026

NOH JS 1 0.026

NOH MR 1 0.026

NOIRI E 1 0.026

NOIZET M 1 0.026

NOLAN CJ 1 0.026

NOLASCO NPC 1 0.026

NOLIN A 1 0.026

NOLL C 1 0.026

NOLTE GT 1 0.026

NOLTE IM 1 0.026

NOMIYA A 1 0.026

NOMURA W 1 0.026

NOONAN WT 1 0.026

NORDHOLM A 1 0.026

NORDIO M 1 0.026

NORDQUIST L 1 0.026

NORLIN J 1 0.026

NORMAN H 1 0.026

NORONHA ID 1 0.026

NORONHA IL 1 0.026

NORREGAARD R 1 0.026

NORTH CS 1 0.026

NORTH P 1 0.026

NORTIER J 1 0.026

NOSAKI T 1 0.026

NOSOVA E 1 0.026

NOTOHAMIPRODJO M 1 0.026

NOUREDDINE L 1 0.026

NOUTSIAS M 1 0.026

NOVEL-CATIN E 1 0.026

NOZAKI Y 1 0.026

NOZAWA E 1 0.026

NOZAWA M 1 0.026

NOZU K 1 0.026

NUALART F 1 0.026

NUGENT MM 1 0.026

NUKUI I 1 0.026

NUMAKURA K 1 0.026

NUNAMAKER EA 1 0.026

NUNES EC 1 0.026

NUNEZ RE 1 0.026

NUNEZ-ALVAREZ C 1 0.026

NUNEZ-GOMEZ E 1 0.026

NUSSDORFER GG 1 0.026

NUTI R 1 0.026

NUTTER FH 1 0.026

NWOKO RE 1 0.026

NYENGAARD JR 1 0.026

NYKAMP K 1 0.026

NYO YH 1 0.026

O ESTACIO R 1 0.026

O'CONNOR C 1 0.026

O'CONNOR CL 1 0.026

O'CONNOR JL 1 0.026

O'CONNOR SE 1 0.026

O'DONOGHUE ML 1 0.026

O'DONOVAN H 1 0.026

O'DONOVAN HC 1 0.026

O'HARE AM 1 0.026

O'MEARA Y 1 0.026

O'MEARA YM 1 0.026

O'NEIL K 1 0.026

O'SEAGHDHA CM 1 0.026

O'SHEA D 1 0.026

O'SHEA PM 1 0.026

O'SULLIVAN ED 1 0.026

O'SULLIVAN J 1 0.026

O'SULLIVAN MJ 1 0.026

OBANA M 1 0.026

OBARA N 1 0.026

OBAYASHI H 1 0.026

OBERKOFLER H 1 0.026

OBERMAYER-PIETSCH B 1 0.026

OBERMULLER N 1 0.026

OCCELLI P 1 0.026

OCTAVIA Y 1 0.026

ODA H 1 0.026

ODAKA H 1 0.026

ODDI G 1 0.026

OE YJ 1 0.026

OEI LS 1 0.026

OEMAR BS 1 0.026

OESTREICHER EM 1 0.026

OFFEN D 1 0.026

OGASAWARA N 1 0.026

OGATA K 1 0.026

OGAWA F 1 0.026

OGAWA K 1 0.026

OGAWA M 1 0.026

OGAWA O 1 0.026

OGBORN MR 1 0.026

OGILVIE RI 1 0.026

OGINO K 1 0.026

OGNIBENE DT 1 0.026

OGRODNIK M 1 0.026

OGUCHI A 1 0.026

OGURA M 1 0.026

OH AN 1 0.026

OH EY 1 0.026

OH HC 1 0.026

OH HY 1 0.026

OH KH 1 0.026

OH SH 1 0.026

OH YJ 1 0.026

OH YK 1 0.026

OH YW 1 0.026

OHARA K 1 0.026

OHASHI H 1 0.026

OHASHI J 1 0.026

OHLSTEIN EH 1 0.026

OHMI A 1 0.026

OHMORI K 1 0.026

OHNO K 1 0.026

OHNO S 1 0.026

OHNO Y 1 0.026

OHNUKI K 1 0.026

OHSAKI Y 1 0.026

OHSHIRO Y 1 0.026

OHTA H 1 0.026

OHTA T 1 0.026

OHTANI T 1 0.026

OHTSUKA S 1 0.026

OHYA Y 1 0.026

OIKAWA T 1 0.026

OISHI H 1 0.026

OK E 1 0.026

OKADA K 1 0.026

OKADA N 1 0.026

OKADA R 1 0.026

OKADA S 1 0.026

OKAMATO H 1 0.026

OKAMURA M 1 0.026

OKAMURA T 1 0.026

OKANO K 1 0.026

OKAYAMA K 1 0.026

OKAZAKI K 1 0.026

OKAZAKI S 1 0.026

OKEDA T 1 0.026

OKERBERG CV 1 0.026

OKETANI M 1 0.026

OKINO K 1 0.026

OKISHIO S 1 0.026

OKON JB 1 0.026

OKU M 1 0.026

OKUDA K 1 0.026

OKUDA T 1 0.026

OKUDAIRA S 1 0.026

OKUMURA M 1 0.026

OKUMURA T 1 0.026

OKUNO K 1 0.026

OKUSA MD 1 0.026

OKUSHI Y 1 0.026

OKUYAMA S 1 0.026

OKUYUCU A 1 0.026

OLDFIELD M 1 0.026

OLDFIELD MD 1 0.026

OLGAARD K 1 0.026

OLINGER E 1 0.026

OLIVEIRA BSD 1 0.026

OLIVEIRA CS 1 0.026

OLIVEIRA J 1 0.026

OLIVEIRA MV 1 0.026

OLIVEIRA PA 1 0.026

OLIVEIRA SM 1 0.026

OLIVEIRA-SOUZA M 1 0.026

OLIVER D 1 0.026

OLIVERAS A 1 0.026

OLIVERAS-FERRAROS C 1 0.026

OLIVETTI G 1 0.026

OLIVIERO G 1 0.026

OLIYARNYK O 1 0.026

OLSEN L 1 0.026

OLSON BA 1 0.026

OLSON JL 1 0.026

OLSON LE 1 0.026

OMAR AG 1 0.026

OMORI Y 1 0.026

ONAY H 1 0.026

ONDRIAS F 1 0.026

ONO H 1 0.026

ONO M 1 0.026

ONO Y 1 0.026

ONO-FUJISAKI A 1 0.026

ONODY A 1 0.026

ONOUE T 1 0.026

ONOZATO ML 1 0.026

OOKAWARA M 1 0.026

OPDEBEECK B 1 0.026

OPSAHL AC 1 0.026

ORANTES CM 1 0.026

ORBE J 1 0.026

ORENA S 1 0.026

OREOPOULOS DG 1 0.026

ORI Y 1 0.026

ORIGASSA CST 1 0.026

ORLIC L 1 0.026

ORLICKY D 1 0.026

ORLICKY DJ 1 0.026

ORLOV NB 1 0.026

OROOJALIAN F 1 0.026

OROUEI S 1 0.026

OROZCO-ORTEGA RA 1 0.026

ORTEGA A 1 0.026

ORTEGA E 1 0.026

ORTEGA MR 1 0.026

ORTMANN J 1 0.026

ORTUNO D 1 0.026

OSAKI K 1 0.026

OSHIMA N 1 0.026

OSIEKI NI 1 0.026

OSMAN E 1 0.026

OSORNIO-GARDUNO DS 1 0.026

OSTERGAARD JA 1 0.026

OSTERGAARD MV 1 0.026

OSTERHOLM AM 1 0.026

OSTOVAN MA 1 0.026

OSWALD F 1 0.026

OTAGIRI M 1 0.026

OTANI M 1 0.026

OTOMO H 1 0.026

OTSU R 1 0.026

OTSUJI Y 1 0.026

OTSUKA H 1 0.026

OTSUKA K 1 0.026

OTSUKA S 1 0.026

OTSUKI M 1 0.026

OTT IM 1 0.026

OTTE-HOLLER I 1 0.026

OTTLAKAN A 1 0.026

OTUKESH H 1 0.026

OU HT 1 0.026

OU YT 1 0.026

OUAHED JD 1 0.026

OUCHI N 1 0.026

OUJO B 1 0.026

OULKAR D 1 0.026

OURY TD 1 0.026

OUYANG CH 1 0.026

OVERHEU D 1 0.026

OWEN AR 1 0.026

OWENS CD 1 0.026

OWENS EP 1 0.026

OYABU T 1 0.026

OYAMA TT 1 0.026

OZA MJ 1 0.026

OZANTURK A 1 0.026

OZCAN MA 1 0.026

OZCELIK B 1 0.026

OZDEMIR D 1 0.026

OZDEMIR TR 1 0.026

OZDOGAN S 1 0.026

OZER CAKIR O 1 0.026

OZER E 1 0.026

OZILOU C 1 0.026

OZKAHYA M 1 0.026

OZTURK S 1 0.026

PAADUKAANA S 1 0.026

PABBIDI MR 1 0.026

PABLA N 1 0.026

PACAUD D 1 0.026

PACHER P 1 0.026

PACHOLCZYK M 1 0.026

PACINI G 1 0.026

PACKARD M 1 0.026

PACKER SC 1 0.026

PACORA P 1 0.026

PADDA R 1 0.026

PADILLA J 1 0.026

PADMANABHAN G 1 0.026

PADWAL RS 1 0.026

PAES-DE-ALMEIDA EC 1 0.026

PAEZ-RUBIO MI 1 0.026

PAGANO P 1 0.026

PAGE JE 1 0.026

PAGE P 1 0.026

PAGE S 1 0.026

PAGNIN E 1 0.026

PAI AL 1 0.026

PAIGE A 1 0.026

PAIK J 1 0.026

PAIK JH 1 0.026

PAIRA S 1 0.026

PAISEY R 1 0.026

PAISEY RB 1 0.026

PAIZIS G 1 0.026

PAK ES 1 0.026

PAL R 1 0.026

PALACIOS CRF 1 0.026

PALACIOS P 1 0.026

PALANISAMY N 1 0.026

PALAZZUOLI A 1 0.026

PALFFY R 1 0.026

PALLADINI G 1 0.026

PALLET N 1 0.026

PALMER AK 1 0.026

PALMER BF 1 0.026

PALMER C 1 0.026

PALOCZI J 1 0.026

PALOMBO C 1 0.026

PALSSON R 1 0.026

PAN BB 1 0.026

PAN CJ 1 0.026

PAN J 1 0.026

PAN K 1 0.026

PAN LY 1 0.026

PAN M 1 0.026

PAN MM 1 0.026

PAN QJ 1 0.026

PAN RH 1 0.026

PAN WJ 1 0.026

PAN X 1 0.026

PAN XD 1 0.026

PAN XP 1 0.026

PAN YH 1 0.026

PANAGIOTOPOULOS S 1 0.026

PANAITESCU B 1 0.026

PANDA S 1 0.026

PANDEY DK 1 0.026

PANDIS M 1 0.026

PANDURU NM 1 0.026

PANFILI Z 1 0.026

PANG J 1 0.026

PANG Q 1 0.026

PANG QQ 1 0.026

PANG WF 1 0.026

PANG WY 1 0.026

PANG XL 1 0.026

PANI A 1 0.026

PANIAGUA R 1 0.026

PANIZZON CPDB 1 0.026

PANNANGPETCH P 1 0.026

PANTERNE C 1 0.026

PAOLETTI S 1 0.026

PAP D 1 0.026

PAPALIA GA 1 0.026

PAPAZOVA DA 1 0.026

PAPE L 1 0.026

PAPINSKA AM 1 0.026

PAPPA M 1 0.026

PARADOWSKI M 1 0.026

PARAJULI S 1 0.026

PARAMASIVAM P 1 0.026

PARAMESHWAR J 1 0.026

PARAVICINI TM 1 0.026

PARDO F 1 0.026

PAREKH N 1 0.026

PARENTE R 1 0.026

PARIKH SM 1 0.026

PARILTAY E 1 0.026

PARK BW 1 0.026

PARK CH 1 0.026

PARK CK 1 0.026

PARK D 1 0.026

PARK HJ 1 0.026

PARK HK 1 0.026

PARK HS 1 0.026

PARK HY 1 0.026

PARK JC 1 0.026

PARK JE 1 0.026

PARK JW 1 0.026

PARK K 1 0.026

PARK KB 1 0.026

PARK KH 1 0.026

PARK KK 1 0.026

PARK KS 1 0.026

PARK M 1 0.026

PARK MH 1 0.026

PARK PK 1 0.026

PARK RW 1 0.026

PARK SR 1 0.026

PARK SW 1 0.026

PARK WD 1 0.026

PARK YJ 1 0.026

PARKAR N 1 0.026

PARKE KS 1 0.026

PARKER C 1 0.026

PARKER E 1 0.026

PARKER MD 1 0.026

PARKER T 1 0.026

PARKKONEN M 1 0.026

PARKS JK 1 0.026

PARKS JS 1 0.026

PARLATI L 1 0.026

PARNHAM S 1 0.026

PARRA EG 1 0.026

PARRA-HERRAN C 1 0.026

PARRISH A 1 0.026

PARSONS M 1 0.026

PARTHASARATHY P 1 0.026

PARVEZ F 1 0.026

PASCHETTA E 1 0.026

PASHA M 1 0.026

PASQUIER AL 1 0.026

PASSARELLI M 1 0.026

PASSERINI P 1 0.026

PASSLICK-DEETJEN J 1 0.026

PASSMORE JC 1 0.026

PASSOS CS 1 0.026

PASTERNACK A 1 0.026

PASTOR-ANGLADA M 1 0.026

PASTORI S 1 0.026

PASUPULATI AK 1 0.026

PATCH AM 1 0.026

PATEL A 1 0.026

PATEL AM 1 0.026

PATEL B 1 0.026

PATEL DM 1 0.026

PATEL EM 1 0.026

PATEL HM 1 0.026

PATEL JK 1 0.026

PATEL M 1 0.026

PATEL MS 1 0.026

PATEL RK 1 0.026

PATEL SK 1 0.026

PATEL TV 1 0.026

PATEL UD 1 0.026

PATEL VB 1 0.026

PATEL VJ 1 0.026

PATERSON A 1 0.026

PATERSON AD 1 0.026

PATHOMTHONGTAWEECHAI N 1 0.026

PATINHA D 1 0.026

PATO J 1 0.026

PATTERS AB 1 0.026

PATTERSON CE 1 0.026

PATZAK A 1 0.026

PAUDEL SD 1 0.026

PAUEKSAKON P 1 0.026

PAUL M 1 0.026

PAULIS L 1 0.026

PAULS K 1 0.026

PAUSCHINGER M 1 0.026

PAVLETIC-PERSIC M 1 0.026

PAVLINKOVA G 1 0.026

PAVONE V 1 0.026

PAWLICKI L 1 0.026

PAWLUCZYK I 1 0.026

PAYER BA 1 0.026

PAYRE B 1 0.026

PAZIK J 1 0.026

PAZOS F 1 0.026

PEACOCK TE 1 0.026

PEARCE D 1 0.026

PEARSON AL 1 0.026

PECCI V 1 0.026

PECHER C 1 0.026

PECKHAM DG 1 0.026

PEDAGOGOS E 1 0.026

PEDERSEN AA 1 0.026

PEDERSEN MH 1 0.026

PEDERSEN OL 1 0.026

PEDERSEN PJ 1 0.026

PEDRINO GR 1 0.026

PEGG K 1 0.026

PEHLIVANOGLU C 1 0.026

PEI CZ 1 0.026

PEI F 1 0.026

PEI XY 1 0.026

PELISSOU C 1 0.026

PELKONEN R 1 0.026

PELLEGRINI G 1 0.026

PELLETIER K 1 0.026

PELLETIER RP 1 0.026

PELLETIER S 1 0.026

PELLICANO V 1 0.026

PELLICORI P 1 0.026

PELLISSETTO C 1 0.026

PELTONEN S 1 0.026

PENA AM 1 0.026

PENA-POLANCO JE 1 0.026

PENDEM S 1 0.026

PENG CL 1 0.026

PENG FF 1 0.026

PENG KS 1 0.026

PENG KX 1 0.026

PENG WH 1 0.026

PENG WS 1 0.026

PENG WT 1 0.026

PENG X 1 0.026

PENG XQ 1 0.026

PENG YJ 1 0.026

PENG YQ 1 0.026

PENG ZN 1 0.026

PENG ZY 1 0.026

PENNER B 1 0.026

PENNINGTON A 1 0.026

PENNO G 1 0.026

PENZKOFER T 1 0.026

PEREGO RA 1 0.026

PEREIRA CD 1 0.026

PEREIRA LG 1 0.026

PEREIRA LMM 1 0.026

PEREIRA SA 1 0.026

PERELLO M 1 0.026

PEREZ J 1 0.026

PEREZ-CALVO JI 1 0.026

PEREZ-MATUTE P 1 0.026

PEREZ-RAMIREZ IF 1 0.026

PEREZ-ROQUE L 1 0.026

PEREZ-TORRES I 1 0.026

PERGOLA PE 1 0.026

PERIANDAVAN K 1 0.026

PERICACHO M 1 0.026

PERICO L 1 0.026

PERIN L 1 0.026

PERIN PC 1 0.026

PERIYASAMY R 1 0.026

PERKINS BA 1 0.026

PERKOVIC V 1 0.026

PERKOWSKA-PTASINSKA A 1 0.026

PERLINI S 1 0.026

PERLMAN AS 1 0.026

PERMYAKOVA A 1 0.026

PERNA A 1 0.026

PERNICONE E 1 0.026

PERRET-GUILLAUME C 1 0.026

PERRETTA-TEJEDOR N 1 0.026

PERRIE Y 1 0.026

PERRY A 1 0.026

PERRY R 1 0.026

PERSEGHIN G 1 0.026

PERSSON F 1 0.026

PERSSON P 1 0.026

PERTICONE F 1 0.026

PERTICONE M 1 0.026

PESANTEZ JL 1 0.026

PESAVENTO TE 1 0.026

PESCE F 1 0.026

PESCHOS D 1 0.026

PESCOVITZ MD 1 0.026

PESHATTIWAR VV 1 0.026

PESQUERO JB 1 0.026

PESSLER A 1 0.026

PETER MO 1 0.026

PETERO VG 1 0.026

PETERS D 1 0.026

PETERS DG 1 0.026

PETERS I 1 0.026

PETERS J 1 0.026

PETERS LJF 1 0.026

PETERS WN 1 0.026

PETERSEN CA 1 0.026

PETERSEN J 1 0.026

PETERSEN M 1 0.026

PETI-PETERDI J 1 0.026

PETRE N 1 0.026

PETRELLA RJ 1 0.026

PETRELLI L 1 0.026

PETRICA M 1 0.026

PETRIK J 1 0.026

PETRILLO F 1 0.026

PETTIT AR 1 0.026

PETUTSCHNIGG J 1 0.026

PEZZONE M 1 0.026

PHAM Y 1 0.026

PHANISH MK 1 0.026

PHILIPPE N 1 0.026

PHILIT F 1 0.026

PHILLIPS AN 1 0.026

PHILLIPS ARJ 1 0.026

PHILLIPS L 1 0.026

PIAO DX 1 0.026

PIAZZA-WAGGONER C 1 0.026

PIAZZI E 1 0.026

PICCOLI GB 1 0.026

PICHAIWONG W 1 0.026

PICHLER R 1 0.026

PICONI S 1 0.026

PIEK A 1 0.026

PIESKE B 1 0.026

PIGA I 1 0.026

PIGNATARO A 1 0.026

PIHLAJANIEMI T 1 0.026

PIIRA OP 1 0.026

PIJL H 1 0.026

PILLAY T 1 0.026

PILLEBOUT E 1 0.026

PINE E 1 0.026

PING F 1 0.026

PING Z 1 0.026

PINHEIRO ME 1 0.026

PINHEIRO SVB 1 0.026

PINKERTON LE 1 0.026

PIORNO P 1 0.026

PIOTROWSKI DW 1 0.026

PIOVAN S 1 0.026

PIRENNE J 1 0.026

PIRES MJ 1 0.026

PIRET SE 1 0.026

PIRSON Y 1 0.026

PISCHETSRIEDER M 1 0.026

PISSAS G 1 0.026

PITCHAIMANI V 1 0.026

PITKANIEMI J 1 0.026

PITTROW D 1 0.026

PIUCI CR 1 0.026

PIUS R 1 0.026

PLAKHT Y 1 0.026

PLANAS J 1 0.026

PLANT BJ 1 0.026

PLANT WD 1 0.026

PLATA C 1 0.026

PLATANIAS LC 1 0.026

PLATNICH JM 1 0.026

PLATT D 1 0.026

PLATT KA 1 0.026

PLAZA JJ 1 0.026

PLEIN S 1 0.026

PLESSIS G 1 0.026

PLETINCK A 1 0.026

PLOTKIN H 1 0.026

PODKALICKA P 1 0.026

POELMAN R 1 0.026

POIRIER B 1 0.026

POIRIER L 1 0.026

POKHAREL S 1 0.026

POL S 1 0.026

POLETTI PT 1 0.026

POLI FE 1 0.026

POLI V 1 0.026

POLIAK N 1 0.026

POLLIO DE 1 0.026

POLLOCK AS 1 0.026

POLO ML 1 0.026

POLONSKI L 1 0.026

POLYZOS SA 1 0.026

POMERO F 1 0.026

PONIKOWSKI P 1 0.026

PONS RA 1 0.026

PONS-VILLANUEVA J 1 0.026

PONTE E 1 0.026

PONTILLO C 1 0.026

PONTOGLIO M 1 0.026

POOMMIPANIT N 1 0.026

POON P 1 0.026

POORDAD F 1 0.026

POPOVTZER MM 1 0.026

POPP RA 1 0.026

POPPAS DP 1 0.026

PORFIRIO Z 1 0.026

PORPIGLIA F 1 0.026

PORRINI E 1 0.026

PORTER JR 1 0.026

PORTILLO MP 1 0.026

POSTORINO A 1 0.026

POTEMPA LA 1 0.026

POTENTA SE 1 0.026

POTHEN P 1 0.026

POTTER JA 1 0.026

POTTER SS 1 0.026

POTTIER A 1 0.026

POTTIER N 1 0.026

POUCHER SM 1 0.026

POUDEL B 1 0.026

POULSON K 1 0.026

POUPON-BOURDY S 1 0.026

POUR AB 1 0.026

POURREAU F 1 0.026

POVEDA J 1 0.026

POVEDA R 1 0.026

POVINEC P 1 0.026

POWELL AK 1 0.026

POWELL RH 1 0.026

POWELSON JA 1 0.026

POZDZIK AA 1 0.026

POZZA RD 1 0.026

POZZI A 1 0.026

POZZI C 1 0.026

POZZONI P 1 0.026

PRABHU A 1 0.026

PRADAT P 1 0.026

PRADDAUDE F 1 0.026

PRADEEP SR 1 0.026

PRADERE JP 1 0.026

PRADO-URIBE C 1 0.026

PRAKASH J 1 0.026

PRAKOURA N 1 0.026

PRASAD GVR 1 0.026

PRASAD N 1 0.026

PRASAD P 1 0.026

PRAT MV 1 0.026

PRAT V 1 0.026

PRATES HFA 1 0.026

PRATI U 1 0.026

PRATSCHKE J 1 0.026

PRATT B 1 0.026

PRATT JH 1 0.026

PRATT S 1 0.026

PRENNER SB 1 0.026

PRESCOTT G 1 0.026

PRESNE C 1 0.026

PRESNELL S 1 0.026

PREVOST G 1 0.026

PREZIUSO C 1 0.026

PRIBULA M 1 0.026

PRICCI F 1 0.026

PRICE O 1 0.026

PRICE SR 1 0.026

PRICKETT TCR 1 0.026

PRIEL A 1 0.026

PRIEM F 1 0.026

PRIETO MC 1 0.026

PRINCE S 1 0.026

PRINGLE KG 1 0.026

PRIOLETTA A 1 0.026

PRIOR S 1 0.026

PRISTAUTZ H 1 0.026

PROCACCIO M 1 0.026

PROCINO G 1 0.026

PROFY A 1 0.026

PROHL C 1 0.026

PROKOP J 1 0.026

PRONAYOVA N 1 0.026

PROSCHAK E 1 0.026

PROVENZANO M 1 0.026

PROVOOST AP 1 0.026

PRUETT TL 1 0.026

PRUSKY GT 1 0.026

PRUSZCZYK P 1 0.026

PRZEWLOCKA-KOSMALA M 1 0.026

PTILOVANCIV EON 1 0.026

PU XY 1 0.026

PU Y 1 0.026

PUCCI KRM 1 0.026

PUCHADES RENAU L 1 0.026

PUCHOWICZ MA 1 0.026

PUCILLO CEM 1 0.026

PUHR M 1 0.026

PUIA Z 1 0.026

PULIDO F 1 0.026

PULJAK L 1 0.026

PULLEN N 1 0.026

PULLEN S 1 0.026

PULLEN SS 1 0.026

PULLMAN JM 1 0.026

PUN N 1 0.026

PUNARO GR 1 0.026

PUNTHAMBAKER S 1 0.026

PUNTILLO E 1 0.026

PUNZI H 1 0.026

PUNZI L 1 0.026

PURCELL R 1 0.026

PUROHIT P 1 0.026

PURVES JT 1 0.026

PUSCASIU T 1 0.026

PUSEY CD 1 0.026

PUSHPAKUMAR S 1 0.026

PUSHPAKUMAR SB 1 0.026

PUTHUMANA J 1 0.026

PUTLURI N 1 0.026

PUTMAN B 1 0.026

PYE C 1 0.026

PYLE LL 1 0.026

PYLYPCHUK G 1 0.026

QI CY 1 0.026

QI DL 1 0.026

QI HL 1 0.026

QI JS 1 0.026

QI MY 1 0.026

QI QA 1 0.026

QI WE 1 0.026

QI WW 1 0.026

QI XY 1 0.026

QI YJ 1 0.026

QIAN BW 1 0.026

QIAN J 1 0.026

QIAN JQ 1 0.026

QIAN SH 1 0.026

QIAN ST 1 0.026

QIAN WW 1 0.026

QIAN XY 1 0.026

QIAN Y 1 0.026

QIAN YJ 1 0.026

QIANG L 1 0.026

QIAO J 1 0.026

QIAO R 1 0.026

QIAO W 1 0.026

QIAO WL 1 0.026

QIAO X 1 0.026

QIAO YH 1 0.026

QIN BJ 1 0.026

QIN CX 1 0.026

QIN H 1 0.026

QIN JG 1 0.026

QIN LD 1 0.026

QIN LF 1 0.026

QIN LL 1 0.026

QIN N 1 0.026

QIN NN 1 0.026

QIN QJ 1 0.026

QIN XT 1 0.026

QIN YH 1 0.026

QING H 1 0.026

QIU AD 1 0.026

QIU CY 1 0.026

QIU DH 1 0.026

QIU HL 1 0.026

QIU HY 1 0.026

QIU J 1 0.026

QIU KY 1 0.026

QIU S 1 0.026

QIU SF 1 0.026

QIU XH 1 0.026

QIU XR 1 0.026

QIU Y 1 0.026

QIU YY 1 0.026

QIU ZH 1 0.026

QU GW 1 0.026

QU LL 1 0.026

QU LX 1 0.026

QU W 1 0.026

QUADRI S 1 0.026

QUAGLIA M 1 0.026

QUAGLINI S 1 0.026

QUAN S 1 0.026

QUAN W 1 0.026

QUAN Y 1 0.026

QUARELLO F 1 0.026

QUE HF 1 0.026

QUELLARD N 1 0.026

QUEREDA C 1 0.026

QUERSIN V 1 0.026

QUIGG R 1 0.026

QUIGLEY JE 1 0.026

QUIMBY KR 1 0.026

QUINKLER M 1 0.026

QUINN CM 1 0.026

QUINN LM 1 0.026

QUINN RR 1 0.026

QUISNO A 1 0.026

QUOIX E 1 0.026

RAAGINEY D 1 0.026

RABAGLIA ME 1 0.026

RABANT M 1 0.026

RABE M 1 0.026

RABEKA M 1 0.026

RABI DM 1 0.026

RABKIN SW 1 0.026

RABOT N 1 0.026

RACCAH D 1 0.026

RACKI S 1 0.026

RADEKE HH 1 0.026

RADLINSKI M 1 0.026

RADOMSKA E 1 0.026

RAFFETSEDER U 1 0.026

RAFFIN M 1 0.026

RAFFIOTTA F 1 0.026

RAFIEIAN-KOPAEI M 1 0.026

RAGI G 1 0.026

RAGONE V 1 0.026

RAHBAR S 1 0.026

RAHMAN M 1 0.026

RAHMAN MH 1 0.026

RAHMAN MM 1 0.026

RAHMANIAN S 1 0.026

RAHMATI-YAMCHI M 1 0.026

RAI R 1 0.026

RAILA J 1 0.026

RAILE K 1 0.026

RAINA P 1 0.026

RAJ DS 1 0.026

RAJ DSC 1 0.026

RAJAMOHAN F 1 0.026

RAJANI R 1 0.026

RAJAVEL V 1 0.026

RAKIC S 1 0.026

RALPH SA 1 0.026

RAMA I 1 0.026

RAMACHANDRAN P 1 0.026

RAMACHANDRARAO S 1 0.026

RAMADESIKAN S 1 0.026

RAMALINGAM A 1 0.026

RAMANATHAN K 1 0.026

RAMANUJAM M 1 0.026

RAMASAMY R 1 0.026

RAMCHANDRA SK 1 0.026

RAMDAS M 1 0.026

RAMESH S 1 0.026

RAMEZANI A 1 0.026

RAMEZANI M 1 0.026

RAMILA D 1 0.026

RAMIREZ AM 1 0.026

RAMIREZ D 1 0.026

RAMIREZ E 1 0.026

RAMIREZ-PEREZ FI 1 0.026

RAMIREZ-SANCHEZ I 1 0.026

RAMIREZ-TORRES A 1 0.026

RAMOS-MONDRAGON R 1 0.026

RAMPALOVA J 1 0.026

RAMPRASATH T 1 0.026

RAN MX 1 0.026

RAN Z 1 0.026

RANCOULE C 1 0.026

RANDALL OS 1 0.026

RANDERS E 1 0.026

RANDLES MJ 1 0.026

RANDOLPH A 1 0.026

RANE S 1 0.026

RANERA MT 1 0.026

RANGAN GK 1 0.026

RANGANATH S 1 0.026

RANGANATHAN P 1 0.026

RANGANATHAN PV 1 0.026

RANTALA I 1 0.026

RAO AN 1 0.026

RAO JL 1 0.026

RAO PS 1 0.026

RAO PSS 1 0.026

RAO V 1 0.026

RAO W 1 0.026

RAO XR 1 0.026

RAO YK 1 0.026

RAPOLA J 1 0.026

RAPOSO NRB 1 0.026

RAPP J 1 0.026

RASCH R 1 0.026

RASCHACK M 1 0.026

RASCHER W 1 0.026

RASHEDI J 1 0.026

RASHID R 1 0.026

RASHTCHIZADEH N 1 0.026

RASKIN P 1 0.026

RASMUSSEN DGK 1 0.026

RASMUSSEN RW 1 0.026

RASMUSSEN SE 1 0.026

RASOOLY RS 1 0.026

RASTOGI A 1 0.026

RATNAIKE S 1 0.026

RATNER LE 1 0.026

RAUCHMAN M 1 0.026

RAUF A 1 0.026

RAUTOU PE 1 0.026

RAVAROTTO V 1 0.026

RAVASI T 1 0.026

RAVENTOS CX 1 0.026

RAVINAL RC 1 0.026

RAVINDRAN K 1 0.026

RAVINDRAN S 1 0.026

RAVISANKAR A 1 0.026

RAZEK AAKA 1 0.026

REAL MI 1 0.026

REBSOMEN L 1 0.026

REBULL M 1 0.026

RECAMONDE-MENDOZA M 1 0.026

RECIO-RODRIGUEZ JI 1 0.026

REDDY KR 1 0.026

REDDY N 1 0.026

REDDY S 1 0.026

REDFIELD MM 1 0.026

REDFIELD RR 1 0.026

REDMAN JE 1 0.026

REED AAC 1 0.026

REED JC 1 0.026

REED L 1 0.026

REEVES WB 1 0.026

REFAIE AF 1 0.026

REGAZZI R 1 0.026

REGEV A 1 0.026

REGINENSI A 1 0.026

REHMAN MT 1 0.026

REIBERGER T 1 0.026

REICH W 1 0.026

REICHE D 1 0.026

REILY C 1 0.026

REIN JL 1 0.026

REINHARD H 1 0.026

REINHARDT DP 1 0.026

REINHECKEL T 1 0.026

REINHOLT FP 1 0.026

REINKE P 1 0.026

REIS AAD 1 0.026

REIS F 1 0.026

REIS LA 1 0.026

REIS MA 1 0.026

REIS NG 1 0.026

REISIN E 1 0.026

REITER RJ 1 0.026

REMUZZI A 1 0.026

REN CX 1 0.026

REN DM 1 0.026

REN GL 1 0.026

REN GQ 1 0.026

REN H 1 0.026

REN HH 1 0.026

REN JF 1 0.026

REN K 1 0.026

REN LS 1 0.026

REN M 1 0.026

REN Q 1 0.026

REN W 1 0.026

REN YL 1 0.026

REN YQ 1 0.026

RENAUDIN K 1 0.026

RENNIE J 1 0.026

RENNKE HG 1 0.026

RENSEN PCN 1 0.026

RENTHLEI L 1 0.026

RENUSHIA R 1 0.026

REPOSSI GM 1 0.026

REPOVA K 1 0.026

RESENDE AC 1 0.026

RESTAGNO G 1 0.026

RETTIG R 1 0.026

REUSCH CE 1 0.026

REUTER S 1 0.026

REUVENI N 1 0.026

REVESZ L 1 0.026

REXHEPAJ R 1 0.026

REYES A 1 0.026

REYES OS 1 0.026

REYES-MARTINEZ C 1 0.026

REYES-UTRERA C 1 0.026

REYNOLDS JM 1 0.026

REYNOSO-CAMACHO R 1 0.026

REZA HM 1 0.026

REZZANI R 1 0.026

RHEE A 1 0.026

RHEE BD 1 0.026

RHEE H 1 0.026

RHO H 1 0.026

RHO YK 1 0.026

RIBEIRO RA 1 0.026

RICARDO SR 1 0.026

RICAURTE L 1 0.026

RICCOBENE R 1 0.026

RICH SS 1 0.026

RICHA S 1 0.026

RICHARDS M 1 0.026

RICHARDS RJ 1 0.026

RICHARDSON A 1 0.026

RICHARDSON I 1 0.026

RIDDER A 1 0.026

RIDER CC 1 0.026

RIDRUEJO E 1 0.026

RIEBER K 1 0.026

RIEDL E 1 0.026

RIEG T 1 0.026

RIESS R 1 0.026

RIESS RH 1 0.026

RIGALLEAU V 1 0.026

RILEY PR 1 0.026

RILEY SG 1 0.026

RIMLAND D 1 0.026

RIMONDI E 1 0.026

RINCON-CHOLES H 1 0.026

RING E 1 0.026

RINSCHEN MM 1 0.026

RIOS-PEREZ EB 1 0.026

RIOUX-LECLERC N 1 0.026

RIPLEY TL 1 0.026

RIPPE C 1 0.026

RIPPIN JD 1 0.026

RITTER O 1 0.026

RIVALAN J 1 0.026

RIVERA E 1 0.026

RIVERA F 1 0.026

RIVERA JA 1 0.026

RIVERO J 1 0.026

RIWANTO M 1 0.026

RIZZARI MD 1 0.026

ROBBINS JA 1 0.026

ROBENEK H 1 0.026

ROBERT T 1 0.026

ROBERTS AM 1 0.026

ROBERTS ISD 1 0.026

ROBERTS VS 1 0.026

ROBERTS WW 1 0.026

ROBERTSON E 1 0.026

ROBERTSON EJ 1 0.026

ROBERTSON S 1 0.026

ROBINSON-COHEN C 1 0.026

ROBSON MG 1 0.026

ROCA-HO H 1 0.026

ROCCHETTI MT 1 0.026

ROCHA JRC 1 0.026

ROCHA LB 1 0.026

ROCHA LR 1 0.026

ROCHA NP 1 0.026

ROCHE HM 1 0.026

ROCHETTE L 1 0.026

RODEN M 1 0.026

RODGERS BD 1 0.026

RODGERS KD 1 0.026

RODGERS KE 1 0.026

RODITI GH 1 0.026

RODRIGO R 1 0.026

RODRIGUES AL 1 0.026

RODRIGUES AM 1 0.026

RODRIGUES ARA 1 0.026

RODRIGUES CE 1 0.026

RODRIGUEZ D 1 0.026

RODRIGUEZ F 1 0.026

RODRIGUEZ MD 1 0.026

RODRIGUEZ RR 1 0.026

RODRIGUEZ W 1 0.026

RODRIGUEZ-CASTELLANO E 1 0.026

RODRIGUEZ-DIEZ R 1 0.026

RODRIGUEZ-HERNANDEZ A 1 0.026

RODRIGUEZ-ITURBE B 1 0.026

RODRIGUEZ-NIEVES JA 1 0.026

RODRIGUEZ-OSORIO L 1 0.026

RODRIGUEZ-PENA AB 1 0.026

RODRIGUEZ-REYNA TS 1 0.026

RODRIGUEZ-RODRIGUEZ AE 1 0.026

ROEDIG H 1 0.026

ROEST CROLLIUS H 1 0.026

ROFE AK 1 0.026

ROGAL SS 1 0.026

ROGERS JD 1 0.026

ROHDE RD 1 0.026

ROHRER TR 1 0.026

ROJAS-MORALES P 1 0.026

ROJAS-RIVERA J 1 0.026

ROKEYA B 1 0.026

ROKONAY R 1 0.026

ROKSNOER LCW 1 0.026

ROLDAN N 1 0.026

ROLF S 1 0.026

ROLIN B 1 0.026

ROLLIN BJ 1 0.026

ROLLINS BJ 1 0.026

ROLPH T 1 0.026

ROLSER M 1 0.026

ROMA F 1 0.026

ROMAN L 1 0.026

ROMAN LJ 1 0.026

ROMANIC AM 1 0.026

ROMANOV VV 1 0.026

ROMAO EA 1 0.026

ROMBAUT K 1 0.026

ROMEIRAS F 1 0.026

ROMEO G 1 0.026

ROMER I 1 0.026

ROMERO CA 1 0.026

ROMERO MJ 1 0.026

ROMERO R 1 0.026

ROMERO-GOMEZ M 1 0.026

ROMERO-TREJO D 1 0.026

ROMPE F 1 0.026

RONCAL-JIMENEZ C 1 0.026

RONDINONE C 1 0.026

RONG PF 1 0.026

RONKINA N 1 0.026

ROONEY MT 1 0.026

ROSA AC 1 0.026

ROSALES I 1 0.026

ROSALES JM 1 0.026

ROSAS SE 1 0.026

ROSE M 1 0.026

ROSEN S 1 0.026

ROSENBERGER D 1 0.026

ROSENFELDT F 1 0.026

ROSENGARD-BARLUND M 1 0.026

ROSENSTIEL P 1 0.026

ROSHANGAR L 1 0.026

ROSHANRAVAN B 1 0.026

ROSHANRAVAN H 1 0.026

ROSOFF JS 1 0.026

ROSOMAN NP 1 0.026

ROSS C 1 0.026

ROSSETTI A 1 0.026

ROSSI C 1 0.026

ROSSI M 1 0.026

ROSSI MC 1 0.026

ROSSO A 1 0.026

ROTH A 1 0.026

ROTH D 1 0.026

ROTHENBERG ME 1 0.026

ROTHSCHILD CB 1 0.026

ROTMANS JI 1 0.026

ROUBSANTHISUK W 1 0.026

ROUCH K 1 0.026

ROUMELIOTIS S 1 0.026

ROUSSEAU M 1 0.026

ROUSSY G 1 0.026

ROVARIS G 1 0.026

ROVEDA L 1 0.026

ROVERANO S 1 0.026

ROVIN BH 1 0.026

ROWLING MJ 1 0.026

ROY B 1 0.026

ROY D 1 0.026

ROYAL V 1 0.026

ROZEC B 1 0.026

ROZING J 1 0.026

RUAN SW 1 0.026

RUAN Y 1 0.026

RUBINSTEIN WS 1 0.026

RUBIO R 1 0.026

RUDOLPH EH 1 0.026

RUEF RA 1 0.026

RUGER BM 1 0.026

RUGGENENTI P 1 0.026

RUGGIERO C 1 0.026

RUGGIERO K 1 0.026

RUIFROK WP 1 0.026

RUIZ C 1 0.026

RUIZ DB 1 0.026

RUIZ P 1 0.026

RUIZ-ANDRES O 1 0.026

RULAND J 1 0.026

RUMBLE JR 1 0.026

RUMPEL E 1 0.026

RUMPEL H 1 0.026

RUNE N 1 0.026

RUOCCO G 1 0.026

RUPEREZ M 1 0.026

RUPESHKUMAR M 1 0.026

RUPIN A 1 0.026

RUPPERT J 1 0.026

RUSCHITZKA FT 1 0.026

RUSINEK H 1 0.026

RUSSEL FG 1 0.026

RUSSELL BH 1 0.026

RUSSELL TL 1 0.026

RUSSO FP 1 0.026

RUSSO G 1 0.026

RUSSO TE 1 0.026

RUSTER C 1 0.026

RUTKOWSKI JL 1 0.026

RUTLEDGE JC 1 0.026

RUZICKA M 1 0.026

RYBIN DV 1 0.026

RYOM L 1 0.026

RYOO IG 1 0.026

RYOU MG 1 0.026

RYSZ J 1 0.026

RYU JH 1 0.026

RYU M 1 0.026

RYUZAKI M 1 0.026

SA S 1 0.026

SAAB G 1 0.026

SAAD Y 1 0.026

SABA F 1 0.026

SABATINE MS 1 0.026

SABBAN B 1 0.026

SABBATINI M 1 0.026

SABBINENI H 1 0.026

SABBISETTI V 1 0.026

SABBISETTI VS 1 0.026

SABIRSH A 1 0.026

SABO AA 1 0.026

SABRA R 1 0.026

SABRIN F 1 0.026

SABRY A 1 0.026

SABRY D 1 0.026

SACCHI L 1 0.026

SACCON F 1 0.026

SACHAN R 1 0.026

SACHARCZUK W 1 0.026

SACKS SH 1 0.026

SADASIVAM SG 1 0.026

SADHAN D 1 0.026

SADLIER D 1 0.026

SAEED MI 1 0.026

SAEKI M 1 0.026

SAEMANN M 1 0.026

SAENGER AK 1 0.026

SAEZ APR 1 0.026

SAFCIUC F 1 0.026

SAGAI M 1 0.026

SAGESHIMA J 1 0.026

SAGHEB MM 1 0.026

SAGONE A 1 0.026

SAGOR MAT 1 0.026

SAHA C 1 0.026

SAHA M 1 0.026

SAHASRABUDHE V 1 0.026

SAHU PK 1 0.026

SAID M 1 0.026

SAID N 1 0.026

SAIDLIER D 1 0.026

SAIGAL S 1 0.026

SAINT RE 1 0.026

SAITO H 1 0.026

SAITO K 1 0.026

SAITO M 1 0.026

SAITO T 1 0.026

SAITO Y 1 0.026

SAKA C 1 0.026

SAKAGAMI H 1 0.026

SAKAI M 1 0.026

SAKAMOTO I 1 0.026

SAKAMOTO K 1 0.026

SAKATA Y 1 0.026

SAKHUJA V 1 0.026

SAKO M 1 0.026

SAKODA H 1 0.026

SAKUMA H 1 0.026

SAKURAI M 1 0.026

SAKURAI S 1 0.026

SAKURAI T 1 0.026

SAKURAI Y 1 0.026

SALAMANCA-BAUTISTA P 1 0.026

SALAMEH G 1 0.026

SALATTO CT 1 0.026

SALCEDO M 1 0.026

SALEKI H 1 0.026

SALEM F 1 0.026

SALEM HA 1 0.026

SALEM RM 1 0.026

SALIC K 1 0.026

SALIDO-RUIZ E 1 0.026

SALINARO F 1 0.026

SALINAS C 1 0.026

SALINAS-GONZALEZ A 1 0.026

SALINAS-PARRA N 1 0.026

SALIS O 1 0.026

SALLABERGER S 1 0.026

SALLEH N 1 0.026

SALLER A 1 0.026

SALLES CA 1 0.026

SALMON A 1 0.026

SALMON IJ 1 0.026

SALOM K 1 0.026

SALOMONE F 1 0.026

SALONER DA 1 0.026

SALSALI A 1 0.026

SALUDES MA 1 0.026

SALVIDIO G 1 0.026

SALYER SA 1 0.026

SALZANO A 1 0.026

SAMADI N 1 0.026

SAMANIEGO M 1 0.026

SAMARGHANDIAN S 1 0.026

SAMBATHKUMAR R 1 0.026

SAMMUT IA 1 0.026

SAMPAIO MS 1 0.026

SAMPIERI CL 1 0.026

SAMRA YA 1 0.026

SAMUEL T 1 0.026

SAN MARTIN JE 1 0.026

SANADA F 1 0.026

SANAI T 1 0.026

SANAJOU D 1 0.026

SANATI M 1 0.026

SANCAR-BAS S 1 0.026

SANCHES TR 1 0.026

SANCHEZ J 1 0.026

SANCHEZ SS 1 0.026

SANDBERG K 1 0.026

SANDBERG M 1 0.026

SANDFORD RS 1 0.026

SANDHOLM N 1 0.026

SANDOVAL PR 1 0.026

SANDOVAL-LOZANO VH 1 0.026

SANDS JM 1 0.026

SANDS RL 1 0.026

SANGALLI F 1 0.026

SANGHAVI M 1 0.026

SANN H 1 0.026

SANNA GM 1 0.026

SANNUTI A 1 0.026

SANSILVESTRI-MOREL P 1 0.026

SANSON E 1 0.026

SANTAMARIA B 1 0.026

SANTELLI A 1 0.026

SANTHAKUMAR A 1 0.026

SANTINI E 1 0.026

SANTORIELLO D 1 0.026

SANTOS I 1 0.026

SANTOS IB 1 0.026

SANTOS JV 1 0.026

SANTOS PF 1 0.026

SANTOS RAS 1 0.026

SANTOS SHS 1 0.026

SANTOS-BEZERRA DP 1 0.026

SANTUCCI L 1 0.026

SANZ J 1 0.026

SARAF M 1 0.026

SARAF MN 1 0.026

SARAF N 1 0.026

SARAHEIMO M 1 0.026

SARAN R 1 0.026

SARFRAZ M 1 0.026

SARI F 1 0.026

SARIKAYA AM 1 0.026

SARKAR P 1 0.026

SARKER MK 1 0.026

SARKER SD 1 0.026

SARNAK MJ 1 0.026

SARRET P 1 0.026

SARTEA MA 1 0.026

SARTI C 1 0.026

SARUTA T 1 0.026

SASAI Y 1 0.026

SASAKI M 1 0.026

SASAKI N 1 0.026

SASANO H 1 0.026

SASASE T 1 0.026

SASSO F 1 0.026

SATHAYE S 1 0.026

SATKO SG 1 0.026

SATMAN I 1 0.026

SATO A 1 0.026

SATO AYS 1 0.026

SATO EI 1 0.026

SATOH F 1 0.026

SATOH J 1 0.026

SATOH S 1 0.026

SATOMI S 1 0.026

SATOSKAR AA 1 0.026

SATOU R 1 0.026

SATRIANO J 1 0.026

SATTA A 1 0.026

SAUERHOEFER S 1 0.026

SAUNDERS E 1 0.026

SAUVE R 1 0.026

SAVARY L 1 0.026

SAWA Y 1 0.026

SAWABE M 1 0.026

SAWADA H 1 0.026

SAWADA N 1 0.026

SAWAI K 1 0.026

SAXENA R 1 0.026

SAYARLIOGLU H 1 0.026

SAYERS R 1 0.026

SCALBERT E 1 0.026

SCANLON TG 1 0.026

SCANTLEBERY AML 1 0.026

SCARFE L 1 0.026

SCHACHNER H 1 0.026

SCHACHTER O 1 0.026

SCHAEFER RM 1 0.026

SCHAFER S 1 0.026

SCHAFFER SW 1 0.026

SCHALIJ MJ 1 0.026

SCHALINSKE KL 1 0.026

SCHANZE A 1 0.026

SCHARPFENECKER M 1 0.026

SCHATTENBERG JM 1 0.026

SCHAUB JA 1 0.026

SCHAUERTE C 1 0.026

SCHAUT RG 1 0.026

SCHEEL PJ 1 0.026

SCHEEN AJ 1 0.026

SCHELLINGL JR 1 0.026

SCHEMBRI JM 1 0.026

SCHENA FP 1 0.026

SCHEPERS E 1 0.026

SCHERBAUM CR 1 0.026

SCHERF K 1 0.026

SCHERMER B 1 0.026

SCHERNTHANER G 1 0.026

SCHETTLER V 1 0.026

SCHIAVON LL 1 0.026

SCHIEPPATI A 1 0.026

SCHIFF ER 1 0.026

SCHIKOWSKI T 1 0.026

SCHILLING WP 1 0.026

SCHILTER H 1 0.026

SCHILTER HC 1 0.026

SCHINNER E 1 0.026

SCHIRMACHER P 1 0.026

SCHLAGWEIN N 1 0.026

SCHLAPBACH A 1 0.026

SCHLERMAN FJ 1 0.026

SCHLINGEMANN R 1 0.026

SCHLOSSMANN J 1 0.026

SCHMAUSS S 1 0.026

SCHMID P 1 0.026

SCHMID R 1 0.026

SCHMIDT A 1 0.026

SCHMIDT H 1 0.026

SCHMIDT KG 1 0.026

SCHMIDTS HL 1 0.026

SCHMIEDEKE B 1 0.026

SCHMIEDEKE D 1 0.026

SCHMITT R 1 0.026

SCHMITZ JM 1 0.026

SCHNEE J 1 0.026

SCHNEIDER M 1 0.026

SCHOENEMANN C 1 0.026

SCHOLLUM JBW 1 0.026

SCHOMIG M 1 0.026

SCHORDAN E 1 0.026

SCHORDAN S 1 0.026

SCHOUTEN EM 1 0.026

SCHREIBER R 1 0.026

SCHREUDER TCMA 1 0.026

SCHRIJVERS BF 1 0.026

SCHRODER K 1 0.026

SCHUBAUER-BERIGAN MK 1 0.026

SCHUBERT J 1 0.026

SCHUBERT M 1 0.026

SCHUBERT T 1 0.026

SCHUELER S 1 0.026

SCHULAK JA 1 0.026

SCHULTE C 1 0.026

SCHULTE T 1 0.026

SCHULTHEISS HP 1 0.026

SCHULTZ BE 1 0.026

SCHULZ BL 1 0.026

SCHULZE-LOHOFF E 1 0.026

SCHULZE-TANZIL G 1 0.026

SCHUPART R 1 0.026

SCHWAB TR 1 0.026

SCHWAKE M 1 0.026

SCHWARTZ S 1 0.026

SCHWARTZMAN M 1 0.026

SCHWARZ C 1 0.026

SCHWEDLER SB 1 0.026

SCHWENGER V 1 0.026

SCHWETZ V 1 0.026

SCIACQUA A 1 0.026

SCIAN MJ 1 0.026

SCOTT DR 1 0.026

SCOTT WR 1 0.026

SCURR LL 1 0.026

SEAH KK 1 0.026

SEBHAT IK 1 0.026

SEBIRE NJ 1 0.026

SEBOKOVA E 1 0.026

SECCHI A 1 0.026

SECCHIERO P 1 0.026

SECCIA T 1 0.026

SECHER T 1 0.026

SECINTI E 1 0.026

SEDA O 1 0.026

SEDEEK M 1 0.026

SEDMAK DD 1 0.026

SEDOR JR 1 0.026

SEEGER H 1 0.026

SEGERS VFM 1 0.026

SEGURO AC 1 0.026

SEI Y 1 0.026

SEICA RM 1 0.026

SEIFFERT D 1 0.026

SEKI T 1 0.026

SEKIGUCHI K 1 0.026

SEKINE A 1 0.026

SEKIZUKA A 1 0.026

SEKO Y 1 0.026

SELBI W 1 0.026

SELBY NM 1 0.026

SELCUK NY 1 0.026

SELEEM Y 1 0.026

SELEJAN SR 1 0.026

SELJEFLOT I 1 0.026

SELLARES J 1 0.026

SELLERS E 1 0.026

SELMI A 1 0.026

SELVAM GS 1 0.026

SELVANAYAGAM JB 1 0.026

SELVES J 1 0.026

SEMPLE T 1 0.026

SENBONMATSU T 1 0.026

SENE D 1 0.026

SENECHAL A 1 0.026

SENGSTOCK D 1 0.026

SENMARU T 1 0.026

SENO S 1 0.026

SEO DY 1 0.026

SEO E 1 0.026

SEO HY 1 0.026

SEO JW 1 0.026

SEO P 1 0.026

SEOK YM 1 0.026

SEONG S 1 0.026

SEPRAMANIAM S 1 0.026

SERINO G 1 0.026

SERIZAWA K 1 0.026

SERLIN Y 1 0.026

SERPAS L 1 0.026

SERRA R 1 0.026

SERRAINO GF 1 0.026

SERRANO I 1 0.026

SESTI G 1 0.026

SETH A 1 0.026

SETHNA C 1 0.026

SETOGUCHI A 1 0.026

SEVAG D 1 0.026

SEWELL KL 1 0.026

SEXTON P 1 0.026

SHA JB 1 0.026

SHA Q 1 0.026

SHA WG 1 0.026

SHACKLEFORD DM 1 0.026

SHADIA H 1 0.026

SHAFIEE MA 1 0.026

SHAGRAWY I 1 0.026

SHAH AM 1 0.026

SHAH AP 1 0.026

SHAH M 1 0.026

SHAH N 1 0.026

SHAH SA 1 0.026

SHAH VN 1 0.026

SHAKHMARDANOVA SA 1 0.026

SHAKIBAEI M 1 0.026

SHAMSHIRSAZ AA 1 0.026

SHAMSUYAROVA A 1 0.026

SHAN CY 1 0.026

SHAN LJ 1 0.026

SHAN XL 1 0.026

SHAN ZX 1 0.026

SHANG GK 1 0.026

SHANG HC 1 0.026

SHANG J 1 0.026

SHANG WJ 1 0.026

SHANKAR A 1 0.026

SHANMUGAM N 1 0.026

SHANMUGASUNDARAM K 1 0.026

SHAO A 1 0.026

SHAO DC 1 0.026

SHAO F 1 0.026

SHAO GF 1 0.026

SHAO JJ 1 0.026

SHAO K 1 0.026

SHAO RS 1 0.026

SHAO YH 1 0.026

SHAO YM 1 0.026

SHAPIRO J 1 0.026

SHAPIRO JI 1 0.026

SHARIFI N 1 0.026

SHARIFIAN M 1 0.026

SHARLAND AF 1 0.026

SHARMA AM 1 0.026

SHARMA D 1 0.026

SHARMA I 1 0.026

SHARMA M 1 0.026

SHARMA P 1 0.026

SHARMA R 1 0.026

SHARMA RK 1 0.026

SHARMA U 1 0.026

SHARPLES L 1 0.026

SHATI AA 1 0.026

SHAVNYA A 1 0.026

SHAW W 1 0.026

SHAWKY M 1 0.026

SHAYMAN JA 1 0.026

SHE L 1 0.026

SHE ZG 1 0.026

SHEA C 1 0.026

SHEASHAA HA 1 0.026

SHEBL FM 1 0.026

SHEEHAN S 1 0.026

SHEGOGUE D 1 0.026

SHEIKH O 1 0.026

SHELDRAKE TA 1 0.026

SHEMESH II 1 0.026

SHEN CJ 1 0.026

SHEN D 1 0.026

SHEN H 1 0.026

SHEN HR 1 0.026

SHEN JG 1 0.026

SHEN L 1 0.026

SHEN LJ 1 0.026

SHEN N 1 0.026

SHEN PL 1 0.026

SHEN PQ 1 0.026

SHEN Q 1 0.026

SHEN SM 1 0.026

SHEN T 1 0.026

SHEN W 1 0.026

SHEN WF 1 0.026

SHEN X 1 0.026

SHEN XH 1 0.026

SHEN ZD 1 0.026

SHEN ZL 1 0.026

SHENG JY 1 0.026

SHENG L 1 0.026

SHENG LL 1 0.026

SHENG XX 1 0.026

SHEPPARD D 1 0.026

SHEPPARD MC 1 0.026

SHEPPECK J 1 0.026

SHERAJEE SJ 1 0.026

SHERMAN AC 1 0.026

SHERMAN KE 1 0.026

SHERMAN R 1 0.026

SHETTY A 1 0.026

SHEVLIN L 1 0.026

SHI BM 1 0.026

SHI C 1 0.026

SHI DY 1 0.026

SHI GM 1 0.026

SHI GQ 1 0.026

SHI H 1 0.026

SHI HX 1 0.026

SHI JH 1 0.026

SHI JJ 1 0.026

SHI L 1 0.026

SHI LQ 1 0.026

SHI M 1 0.026

SHI MM 1 0.026

SHI MZ 1 0.026

SHI R 1 0.026

SHI SL 1 0.026

SHI W 1 0.026

SHI XJ 1 0.026

SHI YQ 1 0.026

SHIBA K 1 0.026

SHIBASAKI A 1 0.026

SHIBATA H 1 0.026

SHIBATA M 1 0.026

SHIBATA T 1 0.026

SHIBOLET O 1 0.026

SHIDE K 1 0.026

SHIEH YH 1 0.026

SHIELDS CA 1 0.026

SHIFFMAN ML 1 0.026

SHIH CC 1 0.026

SHIH YW 1 0.026

SHIIKI H 1 0.026

SHIMA Y 1 0.026

SHIMADA A 1 0.026

SHIMAMURA Y 1 0.026

SHIMAZU N 1 0.026

SHIMIZU A 1 0.026

SHIMIZU F 1 0.026

SHIMIZU K 1 0.026

SHIMIZU M 1 0.026

SHIMIZU N 1 0.026

SHIMODA HK 1 0.026

SHIMODA K 1 0.026

SHIMOMURA I 1 0.026

SHIMURA H 1 0.026

SHIN DH 1 0.026

SHIN H 1 0.026

SHIN HJ 1 0.026

SHIN JH 1 0.026

SHIN JM 1 0.026

SHIN K 1 0.026

SHIN YC 1 0.026

SHIN YJ 1 0.026

SHIN YT 1 0.026

SHINAGAWA-KOBAYASHI Y 1 0.026

SHINDO K 1 0.026

SHINKAI Y 1 0.026

SHINLAPAWITTAYATORN K 1 0.026

SHINOHARA H 1 0.026

SHINOZAKI Y 1 0.026

SHIOMI T 1 0.026

SHIOU YL 1 0.026

SHIRAKAWA K 1 0.026

SHIU SWM 1 0.026

SHIVAPURKAR N 1 0.026

SHLIPAK MG 1 0.026

SHOJI I 1 0.026

SHOKER A 1 0.026

SHOMORI K 1 0.026

SHORE AC 1 0.026

SHORT CD 1 0.026

SHORT M 1 0.026

SHORTLAND J 1 0.026

SHOTORBANI PY 1 0.026

SHOYAMA Y 1 0.026

SHREAY S 1 0.026

SHRIVASTAV S 1 0.026

SHU KH 1 0.026

SHU Q 1 0.026

SHU S 1 0.026

SHU SQ 1 0.026

SHU SST 1 0.026

SHU SW 1 0.026

SHU YW 1 0.026

SHUAI H 1 0.026

SHUAI P 1 0.026

SHWEKE N 1 0.026

SI H 1 0.026

SI ZK 1 0.026

SIAKOTOS AN 1 0.026

SIBILANO R 1 0.026

SICARD L 1 0.026

SIEBENLIST U 1 0.026

SIERKA D 1 0.026

SIGMUND EE 1 0.026

SIGOVAN M 1 0.026

SIJMONSMA TP 1 0.026

SIKKA R 1 0.026

SIKORA P 1 0.026

SILLJE HH 1 0.026

SILVA M 1 0.026

SILVA MO 1 0.026

SILVA P 1 0.026

SILVA RRP 1 0.026

SILVA SRB 1 0.026

SILVEIRA MAD 1 0.026

SILVEIRO SP 1 0.026

SILVERBORN M 1 0.026

SILVERMAN M 1 0.026

SILVERSIDES DW 1 0.026

SIMARD L 1 0.026

SIMEONI A 1 0.026

SIMFOROUSH N 1 0.026

SIMIC I 1 0.026

SIMIONESCU M 1 0.026

SIMKO F 1 0.026

SIMMONDS NJ 1 0.026

SIMMONS CA 1 0.026

SIMOES MJ 1 0.026

SIMON KG 1 0.026

SIMON KJ 1 0.026

SIMON M 1 0.026

SIMON TC 1 0.026

SIMON V 1 0.026

SIMON-BOUY B 1 0.026

SIMPSON DJ 1 0.026

SIMS OT 1 0.026

SIN HK 1 0.026

SINDHI R 1 0.026

SINDHU T 1 0.026

SINGARAYER R 1 0.026

SINGER J 1 0.026

SINGER JD 1 0.026

SINGH A 1 0.026

SINGH J 1 0.026

SINGH RM 1 0.026

SINGH RS 1 0.026

SINGH UK 1 0.026

SINGH V 1 0.026

SINHA IP 1 0.026

SIRI FM 1 0.026

SIRIOPOL D 1 0.026

SIROT Y 1 0.026

SISSON-ROSS S 1 0.026

SITHAMPARANATHAN S 1 0.026

SIVUNEN J 1 0.026

SJOSTROM CD 1 0.026

SKALIOTI C 1 0.026

SKARSFELDT T 1 0.026

SKENE A 1 0.026

SKINNER SL 1 0.026

SKOGLUND C 1 0.026

SKRYPNIKOV V 1 0.026

SKRYPNYK NI 1 0.026

SKRZYPCZAK-JANKUN E 1 0.026

SLAGMAN MCJ 1 0.026

SLATOPOLSKY E 1 0.026

SLAUGHTER TN 1 0.026

SLAVOTINEK A 1 0.026

SLAWINSKA-MORAWSKA M 1 0.026

SLEEMAN MW 1 0.026

SLOAN A 1 0.026

SLOAN BJ 1 0.026

SLOMOVIC S 1 0.026

SLYNE J 1 0.026

SMALL DM 1 0.026

SMALLWOOD RA 1 0.026

SMELCEROVIC A 1 0.026

SMILES A 1 0.026

SMILES AM 1 0.026

SMINA TP 1 0.026

SMIT JWA 1 0.026

SMITH BH 1 0.026

SMITH C 1 0.026

SMITH CM 1 0.026

SMITH DM 1 0.026

SMITH E 1 0.026

SMITH ER 1 0.026

SMITH J 1 0.026

SMITH RE 1 0.026

SMITH RN 1 0.026

SMITH SA 1 0.026

SMITH SW 1 0.026

SMOLAR J 1 0.026

SMOLLE M 1 0.026

SMOYER WE 1 0.026

SMYTH AR 1 0.026

SNIJCKERS C 1 0.026

SO WV 1 0.026

SOARES AA 1 0.026

SOARES TD 1 0.026

SOBH MA 1 0.026

SOBREVIA L 1 0.026

SOCHA MJ 1 0.026

SODERLUND J 1 0.026

SOFRONIADOU S 1 0.026

SOGABE A 1 0.026

SOGAWA N 1 0.026

SOGAWA Y 1 0.026

SOHN HS 1 0.026

SOHN M 1 0.026

SOIN AS 1 0.026

SOKHI J 1 0.026

SOL M 1 0.026

SOLA A 1 0.026

SOLA E 1 0.026

SOLAK Y 1 0.026

SOLARINO R 1 0.026

SOLEZ K 1 0.026

SOLIMENE U 1 0.026

SOLINI A 1 0.026

SOLIS EG 1 0.026

SOLIS G 1 0.026

SOLOGOVA SS 1 0.026

SOLOMON R 1 0.026

SOLOWAY MS 1 0.026

SOLTYS KA 1 0.026

SOMA M 1 0.026

SOMANATH PR 1 0.026

SOMANI GS 1 0.026

SOMASUNDARAM R 1 0.026

SOMMER M 1 0.026

SOMMER P 1 0.026

SOMMERFELD N 1 0.026

SON Y 1 0.026

SONE M 1 0.026

SONG AN 1 0.026

SONG HN 1 0.026

SONG HP 1 0.026

SONG IH 1 0.026

SONG J 1 0.026

SONG JS 1 0.026

SONG JY 1 0.026

SONG KH 1 0.026

SONG L 1 0.026

SONG LT 1 0.026

SONG M 1 0.026

SONG N 1 0.026

SONG P 1 0.026

SONG PA 1 0.026

SONG R 1 0.026

SONG SJ 1 0.026

SONG SX 1 0.026

SONG W 1 0.026

SONG XM 1 0.026

SONG XR 1 0.026

SONG XW 1 0.026

SONG YG 1 0.026

SONG ZJ 1 0.026

SONG ZP 1 0.026

SONG ZX 1 0.026

SONKODI S 1 0.026

SONNENBLICK EH 1 0.026

SONO M 1 0.026

SONTA T 1 0.026

SOONG Y 1 0.026

SORCINI M 1 0.026

SORENSEN CM 1 0.026

SORENSEN-ZENDER I 1 0.026

SORIANO V 1 0.026

SORO-PAAVONEN A 1 0.026

SOROKIN L 1 0.026

SOROP O 1 0.026

SOROUR SM 1 0.026

SOTI C 1 0.026

SOTO K 1 0.026

SOTO V 1 0.026

SOTOMAYOR CG 1 0.026

SOULIS T 1 0.026

SOURIJ H 1 0.026

SOUSA T 1 0.026

SOUTHERN KW 1 0.026

SOUZA CS 1 0.026

SOUZA RPS 1 0.026

SOUZA SC 1 0.026

SOUZA-MENEZES J 1 0.026

SOWA JP 1 0.026

SOWERS D 1 0.026

SOWERS J 1 0.026

SOYAMA A 1 0.026

SOYLEMEZOGLU O 1 0.026

SOYLU A 1 0.026

SPATZ ES 1 0.026

SPECKS U 1 0.026

SPECTOR KS 1 0.026

SPEER T 1 0.026

SPEIL C 1 0.026

SPEK AC 1 0.026

SPENCER MW 1 0.026

SPENCER S 1 0.026

SPENCER T 1 0.026

SPERLING KR 1 0.026

SPICER TS 1 0.026

SPIERA R 1 0.026

SPILLMANN F 1 0.026

SPINNER ML 1 0.026

SPIRES T 1 0.026

SPITALEWITZ S 1 0.026

SPONG R 1 0.026

SPOREA I 1 0.026

SPOREA L 1 0.026

SPRAGUE SM 1 0.026

SPRAY BJ 1 0.026

SPURNEY RF 1 0.026

SPUSTOVA V 1 0.026

SREDNI B 1 0.026

SREEDHAR R 1 0.026

SRIDHAR S 1 0.026

SRIDHAR VS 1 0.026

SRINIVASAN AK 1 0.026

SRINIVASAN K 1 0.026

SRINIVASAN P 1 0.026

SRIRAMARAO P 1 0.026

SRIVASTAVA SK 1 0.026

SRIYASTAVA SP 1 0.026

SROUGI M 1 0.026

ST CLAIR EW 1 0.026

STACCHIOTTI A 1 0.026

STACHURSKA A 1 0.026

STAEHR M 1 0.026

STAHL K 1 0.026

STAHL R 1 0.026

STAM K 1 0.026

STAM W 1 0.026

STAMBE C 1 0.026

STANLEY K 1 0.026

STANNERS SR 1 0.026

STAPLETON DI 1 0.026

STARCK M 1 0.026

STAROST MF 1 0.026

STARUSCHENKO A 1 0.026

STASCH JP 1 0.026

STAUDER G 1 0.026

STAUDINGER P 1 0.026

STAVARACHI M 1 0.026

STAVARU C 1 0.026

STAVROULOPOULOS A 1 0.026

STEEDS RP 1 0.026

STEEL L 1 0.026

STEEN DL 1 0.026

STEFAN M 1 0.026

STEFANIDIS I 1 0.026

STEFANUTTI A 1 0.026

STEFENELLI C 1 0.026

STEGEMAN CA 1 0.026

STEIGER S 1 0.026

STEIGERWALT SP 1 0.026

STEIN CS 1 0.026

STEIN G 1 0.026

STEIN R 1 0.026

STEINMAN TI 1 0.026

STELLA M 1 0.026

STENKULA KG 1 0.026

STENO B 1 0.026

STENOVA E 1 0.026

STENVINKEL P 1 0.026

STEPAN KM 1 0.026

STEPANOVSKA B 1 0.026

STEPP D 1 0.026

STEPPAN S 1 0.026

STERN M 1 0.026

STERN RC 1 0.026

STERNIK P 1 0.026

STEVENS KK 1 0.026

STEVENS SR 1 0.026

STEVIGNY C 1 0.026

STEWART DJ 1 0.026

STEWART J 1 0.026

STEWART K 1 0.026

STEWART PM 1 0.026

STEWART S 1 0.026

STICCHI D 1 0.026

STIEF A 1 0.026

STIEGER B 1 0.026

STIEHL DP 1 0.026

STIER CT 1 0.026

STITT AW 1 0.026

STOELCKER B 1 0.026

STOLZ DB 1 0.026

STONE JA 1 0.026

STONE JH 1 0.026

STONE JR 1 0.026

STOOP R 1 0.026

STORCH GA 1 0.026

STOURNARAS C 1 0.026

STRADA G 1 0.026

STRATTA P 1 0.026

STREBECK F 1 0.026

STRIBOS EGD 1 0.026

STRIFFLER JS 1 0.026

STROMSTEDT M 1 0.026

STRONG R 1 0.026

STUBBS A 1 0.026

STURMAN JA 1 0.026

SU B 1 0.026

SU BH 1 0.026

SU GB 1 0.026

SU HF 1 0.026

SU J 1 0.026

SU LL 1 0.026

SU MJ 1 0.026

SU Q 1 0.026

SU SH 1 0.026

SU SQ 1 0.026

SU WF 1 0.026

SU WL 1 0.026

SU X 1 0.026

SU XL 1 0.026

SU XQ 1 0.026

SU ZX 1 0.026

SUAREZ-ALVAREZ B 1 0.026

SUBATHRA M 1 0.026

SUBESINGHE M 1 0.026

SUBHAN N 1 0.026

SUBILEAU M 1 0.026

SUBRAMANIAN SC 1 0.026

SUCHAL K 1 0.026

SUDANO I 1 0.026

SUDIRMAN S 1 0.026

SUDUNABUQI S 1 0.026

SUE L 1 0.026

SUEMATSU M 1 0.026

SUETA D 1 0.026

SUGAHARA M 1 0.026

SUGAMATA M 1 0.026

SUGANUMA Y 1 0.026

SUGATANI T 1 0.026

SUGAYA M 1 0.026

SUGIMOTO R 1 0.026

SUGIRI D 1 0.026

SUGIURA Y 1 0.026

SUGIYAMA K 1 0.026

SUH JS 1 0.026

SUH SH 1 0.026

SUI B 1 0.026

SUI JY 1 0.026

SUI Y 1 0.026

SUICA VI 1 0.026

SUICMEZ M 1 0.026

SUKUMARAN V 1 0.026

SULAIMAN SA 1 0.026

SULERIA HAR 1 0.026

SULLIVAN JC 1 0.026

SULLIVAN T 1 0.026

SULTAN B 1 0.026

SUMIDA H 1 0.026

SUMMERS AM 1 0.026

SUMUAL S 1 0.026

SUN BS 1 0.026

SUN CH 1 0.026

SUN CY 1 0.026

SUN DF 1 0.026

SUN DJ 1 0.026

SUN DM 1 0.026

SUN FY 1 0.026

SUN GB 1 0.026

SUN GG 1 0.026

SUN GQ 1 0.026

SUN HZ 1 0.026

SUN JD 1 0.026

SUN JW 1 0.026

SUN JY 1 0.026

SUN K 1 0.026

SUN MN 1 0.026

SUN MRM 1 0.026

SUN MY 1 0.026

SUN P 1 0.026

SUN QM 1 0.026

SUN RX 1 0.026

SUN S 1 0.026

SUN SR 1 0.026

SUN TL 1 0.026

SUN WB 1 0.026

SUN WF 1 0.026

SUN WJ 1 0.026

SUN WN 1 0.026

SUN WS 1 0.026

SUN XB 1 0.026

SUN XD 1 0.026

SUN XF 1 0.026

SUN XJ 1 0.026

SUN XY 1 0.026

SUN YA 1 0.026

SUN YC 1 0.026

SUN YF 1 0.026

SUN ZQ 1 0.026

SUN ZX 1 0.026

SUN ZY 1 0.026

SUNDRAM K 1 0.026

SUNDY JS 1 0.026

SUNG JM 1 0.026

SUNG MJ 1 0.026

SUNSOA H 1 0.026

SUO CJ 1 0.026

SUPRAMANIYAM S 1 0.026

SUREN D 1 0.026

SURIGUGA 1 0.026

SUSZYNSKI TM 1 0.026

SUTARIYA BK 1 0.026

SUTHAHAR N 1 0.026

SUTHERLAND E 1 0.026

SUTTERWALA FS 1 0.026

SUTTON-TYRRELL K 1 0.026

SUVITAIVAL T 1 0.026

SUZUKI D 1 0.026

SUZUKI E 1 0.026

SUZUKI M 1 0.026

SVEC F 1 0.026

SVELTO M 1 0.026

SVENNINGSEN P 1 0.026

SVISTUNOV AA 1 0.026

SWAMINATHAN S 1 0.026

SWAMY GK 1 0.026

SWAN EJ 1 0.026

SWARD K 1 0.026

SWEENEY K 1 0.026

SWOBODA PP 1 0.026

SWYNGHEDAUW B 1 0.026

SYED A 1 0.026

SYPNIEWSKA G 1 0.026

SYREENI A 1 0.026

SYRJANEN J 1 0.026

SYTWU HK 1 0.026

SZABO V 1 0.026

SZADKOWSKA I 1 0.026

SZADKOWSKI K 1 0.026

SZALAI AJ 1 0.026

SZCZEPANSKA-SADOWSKA E 1 0.026

SZEBENI B 1 0.026

SZKIBINSZKIJ E 1 0.026

SZLAVIK N 1 0.026

SZTECHMAN D 1 0.026

SZYNDRALEWIEZ C 1 0.026

SZYSZKA A 1 0.026

TABATA Y 1 0.026

TABATABAEIFAR M 1 0.026

TABER DJ 1 0.026

TABIBIAN JH 1 0.026

TACCETTI G 1 0.026

TACHIBANA S 1 0.026

TACHIKAWA H 1 0.026

TACKE F 1 0.026

TADA Y 1 0.026

TADDEO D 1 0.026

TADMOR H 1 0.026

TAE IH 1 0.026

TAFT JL 1 0.026

TAGER AM 1 0.026

TAGUCHI K 1 0.026

TAGUMA Y 1 0.026

TAHARA T 1 0.026

TAHASEEN SV 1 0.026

TAHK H 1 0.026

TAI TA 1 0.026

TAILA M 1 0.026

TAJIMA N 1 0.026

TAKABATAKE Y 1 0.026

TAKACS IM 1 0.026

TAKACS T 1 0.026

TAKADA Y 1 0.026

TAKAHAMA H 1 0.026

TAKAHARA S 1 0.026

TAKAHASHI HE 1 0.026

TAKAKURA K 1 0.026

TAKAKURA S 1 0.026

TAKAMI Y 1 0.026

TAKAMIYA Y 1 0.026

TAKAMURA T 1 0.026

TAKANASHI-YANOBU R 1 0.026

TAKANO K 1 0.026

TAKANO M 1 0.026

TAKANO Y 1 0.026

TAKAO T 1 0.026

TAKAOKA M 1 0.026

TAKASAWA K 1 0.026

TAKASE HM 1 0.026

TAKASE M 1 0.026

TAKASHIMA S 1 0.026

TAKATA S 1 0.026

TAKAYANAGI K 1 0.026

TAKECHI H 1 0.026

TAKECHI K 1 0.026

TAKEDA K 1 0.026

TAKEDA S 1 0.026

TAKEDA-WATANABE A 1 0.026

TAKEMURA M 1 0.026

TAKENAKA M 1 0.026

TAKETANI H 1 0.026

TAKEUCHI M 1 0.026

TAKEUCHI Y 1 0.026

TAKEYA M 1 0.026

TAKIGUCHI M 1 0.026

TAKISHITA S 1 0.026

TAKIUE K 1 0.026

TAKIYAMA T 1 0.026

TAKIZAWA S 1 0.026

TAKUWA Y 1 0.026

TALER SJ 1 0.026

TALLMAN J 1 0.026

TALSMA DT 1 0.026

TAM FW 1 0.026

TAM VK 1 0.026

TAMAKI S 1 0.026

TAMAKI T 1 0.026

TAMAKI Z 1 0.026

TAMARGO M 1 0.026

TAMBELLINI R 1 0.026

TAMMEN H 1 0.026

TAMSMA JT 1 0.026

TAMURA Y 1 0.026

TAN CM 1 0.026

TAN HF 1 0.026

TAN HJ 1 0.026

TAN J 1 0.026

TAN JJ 1 0.026

TAN JQ 1 0.026

TAN M 1 0.026

TAN TK 1 0.026

TAN X 1 0.026

TAN XY 1 0.026

TAN YF 1 0.026

TANABE A 1 0.026

TANABE M 1 0.026

TANAKA A 1 0.026

TANAKA N 1 0.026

TANAKA R 1 0.026

TANDON C 1 0.026

TANDON S 1 0.026

TANG B 1 0.026

TANG CL 1 0.026

TANG CR 1 0.026

TANG D 1 0.026

TANG DM 1 0.026

TANG FJ 1 0.026

TANG G 1 0.026

TANG JM 1 0.026

TANG LH 1 0.026

TANG LX 1 0.026

TANG MJ 1 0.026

TANG NJ 1 0.026

TANG NN 1 0.026

TANG Q 1 0.026

TANG QZ 1 0.026

TANG SQ 1 0.026

TANG WH 1 0.026

TANG WHW 1 0.026

TANG WX 1 0.026

TANG WZ 1 0.026

TANG XF 1 0.026

TANG XQ 1 0.026

TANG YB 1 0.026

TANG YH 1 0.026

TANG YP 1 0.026

TANG YT 1 0.026

TANG YZ 1 0.026

TANG ZX 1 0.026

TANIGUCHI M 1 0.026

TANIGUCHI T 1 0.026

TANIGUCHI Y 1 0.026

TANIMOTO A 1 0.026

TANIZAWA T 1 0.026

TANRIOVER B 1 0.026

TANTAU J 1 0.026

TAO CH 1 0.026

TAO GL 1 0.026

TAO JL 1 0.026

TAO SB 1 0.026

TAO T 1 0.026

TAO WW 1 0.026

TAO Y 1 0.026

TAPIA J 1 0.026

TAPPARO M 1 0.026

TAPPUNI AR 1 0.026

TARAFDER A 1 0.026

TARANTINI L 1 0.026

TARASOV VV 1 0.026

TARDIF SD 1 0.026

TARECTECAN AA 1 0.026

TAREEVA IE 1 0.026

TARIF N 1 0.026

TARTAGLINO B 1 0.026

TARZIA V 1 0.026

TASANARONG A 1 0.026

TASCANOV MB 1 0.026

TASCI SK 1 0.026

TASHIRO Y 1 0.026

TATSINKAM A 1 0.026

TATU AM 1 0.026

TAURA D 1 0.026

TAVARES P 1 0.026

TAVASOLI M 1 0.026

TAVERNA D 1 0.026

TAYAMA Y 1 0.026

TAYLOR A 1 0.026

TAYLOR CG 1 0.026

TAYLOR CT 1 0.026

TAYLOR L 1 0.026

TAYLOR RS 1 0.026

TAYLOR SM 1 0.026

TAYLOR V 1 0.026

TCHERNYCHEV B 1 0.026

TECTOR AJ 1 0.026

TEEGEN EM 1 0.026

TEENAN O 1 0.026

TEERLINK JR 1 0.026

TEH R 1 0.026

TEH YS 1 0.026

TEHRANI HA 1 0.026

TEICHERT T 1 0.026

TEISSIER T 1 0.026

TEIXEIRA AL 1 0.026

TEIXEIRA F 1 0.026

TEIXEIRA LC 1 0.026

TEIXEIRA MM 1 0.026

TEIXEIRA VC 1 0.026

TEJWANI V 1 0.026

TEKESIN I 1 0.026

TEKIN-NEIJMANN S 1 0.026

TELLEZ A 1 0.026

TENG J 1 0.026

TENG YJ 1 0.026

TERADA Y 1 0.026

TERAI S 1 0.026

TERAMI T 1 0.026

TERAOKA S 1 0.026

TERASAKI F 1 0.026

TERAUCHI Y 1 0.026

TERI A 1 0.026

TERKER AS 1 0.026

TERRAULT NA 1 0.026

TERRAZ-DURASNEL C 1 0.026

TERVAERT JWC 1 0.026

TERZI F 1 0.026

TESCHNER M 1 0.026

TESS DA 1 0.026

TESSIER FJ 1 0.026

TESTANI JM 1 0.026

TESTOLINA M 1 0.026

THACKER JM 1 0.026

THADHANI RI 1 0.026

THAKKER RV 1 0.026

THAKRAR MV 1 0.026

THAKUR S 1 0.026

THAKURDESAI PA 1 0.026

THANH TN 1 0.026

THAWKO T 1 0.026

THAWORNCHINSOMBUT S 1 0.026

THEBERGE MC 1 0.026

THEILADE S 1 0.026

THEILIG F 1 0.026

THEILMEIER G 1 0.026

THELMA BK 1 0.026

THEOCHARIS S 1 0.026

THIBAULT N 1 0.026

THIEMANN S 1 0.026

THIEME K 1 0.026

THIERRY A 1 0.026

THIJS L 1 0.026

THIJSSEN J 1 0.026

THILAGANATHAN B 1 0.026

THIRUGNANASOTHY L 1 0.026

THISTED L 1 0.026

THNG CH 1 0.026

THOENY HC 1 0.026

THOMAS AA 1 0.026

THOMAS CR 1 0.026

THOMAS D 1 0.026

THOMAS DB 1 0.026

THOMAS L 1 0.026

THOMAS M 1 0.026

THOMASOVA D 1 0.026

THOMPSON AM 1 0.026

THOMPSON EJ 1 0.026

THOMSEN LH 1 0.026

THOMSEN MB 1 0.026

THOMSON N 1 0.026

THOMSON NM 1 0.026

THOMSON SE 1 0.026

THON A 1 0.026

THONGNAK L 1 0.026

THONGNAK LO 1 0.026

THORBURN DR 1 0.026

THORIKAY M 1 0.026

THORN LM 1 0.026

THORNHILL BA 1 0.026

THRANE ST 1 0.026

THURMOND RL 1 0.026

TIAN CY 1 0.026

TIAN D 1 0.026

TIAN FQ 1 0.026

TIAN H 1 0.026

TIAN HM 1 0.026

TIAN HY 1 0.026

TIAN HZ 1 0.026

TIAN J 1 0.026

TIAN LM 1 0.026

TIAN PP 1 0.026

TIAN SF 1 0.026

TIAN W 1 0.026

TIAN WD 1 0.026

TIAN WF 1 0.026

TIAN XH 1 0.026

TIAN XQ 1 0.026

TIAN YC 1 0.026

TIAN YM 1 0.026

TIAN ZQ 1 0.026

TIAUW V 1 0.026

TIBALDI E 1 0.026

TIKKANEN H 1 0.026

TILLER AM 1 0.026

TILMON R 1 0.026

TILTON RG 1 0.026

TIMAR B 1 0.026

TIMSIT J 1 0.026

TIMSIT MO 1 0.026

TING A 1 0.026

TINZMANN R 1 0.026

TIROSH B 1 0.026

TISATO V 1 0.026

TISLER A 1 0.026

TIWARI AK 1 0.026

TIWARI V 1 0.026

TOBAR A 1 0.026

TOBE S 1 0.026

TOBE SW 1 0.026

TOBLLI J 1 0.026

TOCEWICZ K 1 0.026

TOCHIGI Y 1 0.026

TOD P 1 0.026

TODA M 1 0.026

TODA N 1 0.026

TODIRAS M 1 0.026

TOEDEBUSCH R 1 0.026

TOFTENG SS 1 0.026

TOGAWA A 1 0.026

TOGAWA H 1 0.026

TOGAWA M 1 0.026

TOGLIATTO G 1 0.026

TOH BH 1 0.026

TOH I 1 0.026

TOHYAMA K 1 0.026

TOKUDOME T 1 0.026

TOKUMOTO M 1 0.026

TOKUNAGA K 1 0.026

TOKUSHIGE K 1 0.026

TOKUTOMI Y 1 0.026

TOLAN DR 1 0.026

TOLLER C 1 0.026

TOLONEN N 1 0.026

TOMAR T 1 0.026

TOMASONI S 1 0.026

TOMAT A 1 0.026

TOMIC TT 1 0.026

TOMIDOKORO K 1 0.026

TOMINAGA T 1 0.026

TOMIOKA S 1 0.026

TOMITA H 1 0.026

TOMITA K 1 0.026

TOMITA M 1 0.026

TOMLINSON LA 1 0.026

TOMOVIC K 1 0.026

TOMSON CR 1 0.026

TOMURA Y 1 0.026

TONELIUS P 1 0.026

TONG B 1 0.026

TONG D 1 0.026

TONG F 1 0.026

TONG JD 1 0.026

TONG N 1 0.026

TONG X 1 0.026

TONSHOFF B 1 0.026

TOOSI MN 1 0.026

TOPAL H 1 0.026

TOPLISS D 1 0.026

TORII K 1 0.026

TORIIZUKA K 1 0.026

TORIMURA T 1 0.026

TORMEN M 1 0.026

TORRE-VILLALVAZO I 1 0.026

TORREGGIANI M 1 0.026

TORRES F 1 0.026

TORRES IB 1 0.026

TORRES N 1 0.026

TORRES VE 1 0.026

TORRES-RAMOS YD 1 0.026

TORSELLO B 1 0.026

TORTEREAU A 1 0.026

TORTI SV 1 0.026

TORUN D 1 0.026

TOS T 1 0.026

TOSONI A 1 0.026

TOSSIDOU I 1 0.026

TOSTADO-GONZALEZ M 1 0.026

TOSTES RC 1 0.026

TOTO R 1 0.026

TOTO RD 1 0.026

TOTSUNE K 1 0.026

TOUCHARD G 1 0.026

TOUZET S 1 0.026

TOVAR AR 1 0.026

TOWNEND JN 1 0.026

TOYAMA K 1 0.026

TOYOKUNI S 1 0.026

TOZ H 1 0.026

TRABACE T 1 0.026

TRACY RP 1 0.026

TRAHAN S 1 0.026

TRAN MT 1 0.026

TRAN SLM 1 0.026

TRAN TT 1 0.026

TRANDAFIRESCU V 1 0.026

TRAUNER M 1 0.026

TRAUPE T 1 0.026

TRAUTWEIN C 1 0.026

TRAYKOVA-BRAUCH M 1 0.026

TREDUP C 1 0.026

TREGOUET DA 1 0.026

TREMBLAY G 1 0.026

TREPICCIONE F 1 0.026

TREVISANI A 1 0.026

TRIFA AP 1 0.026

TRIGKA K 1 0.026

TRIKUDANATHAN S 1 0.026

TRIMBLE G 1 0.026

TRIMBOLI D 1 0.026

TRINACTY CM 1 0.026

TRIPATHY S 1 0.026

TRISCHBERGER C 1 0.026

TRITTA S 1 0.026

TROGADIS J 1 0.026

TROJANOWSKA M 1 0.026

TROLLIET P 1 0.026

TROMP J 1 0.026

TRONC F 1 0.026

TROST K 1 0.026

TROTTA F 1 0.026

TROYANOV S 1 0.026

TRUDEAU L 1 0.026

TRUDEAU S 1 0.026

TRUJILLO J 1 0.026

TRULL A 1 0.026

TRUMMER C 1 0.026

TRUONG LD 1 0.026

TRYGGVASON K 1 0.026

TRYPHONOPOULOS P 1 0.026

TRZEBICKI J 1 0.026

TSAI CH 1 0.026

TSAI CS 1 0.026

TSAI FC 1 0.026

TSAI IT 1 0.026

TSAI JF 1 0.026

TSAI JP 1 0.026

TSAI PJ 1 0.026

TSAI PY 1 0.026

TSAI SH 1 0.026

TSAI SY 1 0.026

TSAI YC 1 0.026

TSAI YL 1 0.026

TSAI YS 1 0.026

TSAKAS S 1 0.026

TSALAMANDRIS C 1 0.026

TSALASTRA W 1 0.026

TSANG M 1 0.026

TSAPENKO M 1 0.026

TSAPENKO MV 1 0.026

TSCHOPE C 1 0.026

TSE HF 1 0.026

TSENG HF 1 0.026

TSENG WL 1 0.026

TSIANI E 1 0.026

TSIMARATOS M 1 0.026

TSOGBADRAKH B 1 0.026

TSUBOI H 1 0.026

TSUBOI N 1 0.026

TSUBOUCHI H 1 0.026

TSUCHIDA H 1 0.026

TSUCHIYAMA Y 1 0.026

TSUJI N 1 0.026

TSUJI T 1 0.026

TSUJIMOTO H 1 0.026

TSUJIMURA T 1 0.026

TSUKAMOTO M 1 0.026

TSUKAMOTO Y 1 0.026

TSUKIYAMA K 1 0.026

TSUMOTO H 1 0.026

TSUMURA N 1 0.026

TSUNEMI A 1 0.026

TSUNEYAMA K 1 0.026

TSURUGA E 1 0.026

TSURUOKA H 1 0.026

TSURUTA S 1 0.026

TSURUTA Y 1 0.026

TSURUYA K 1 0.026

TSUTSUI M 1 0.026

TSYTKIN-KIRSCHENZWEIG S 1 0.026

TU CS 1 0.026

TU H 1 0.026

TU JK 1 0.026

TU L 1 0.026

TU M 1 0.026

TU NM 1 0.026

TU YF 1 0.026

TU YG 1 0.026

TU YJ 1 0.026

TU Z 1 0.026

TUCCARI G 1 0.026

TUCKER JK 1 0.026

TUEGEL C 1 0.026

TUEROS L 1 0.026

TULPPO MP 1 0.026

TULUCE K 1 0.026

TULUCE SY 1 0.026

TUMLIN JA 1 0.026

TUNCAY E 1 0.026

TUNG HD 1 0.026

TUNG YT 1 0.026

TUOMILEHTO J 1 0.026

TURA A 1 0.026

TURAL C 1 0.026

TURAN B 1 0.026

TURK T 1 0.026

TURKAY C 1 0.026

TURKIN I 1 0.026

TURKMEN M 1 0.026

TURNA J 1 0.026

TURNER JM 1 0.026

TURNER M 1 0.026

TURUNEN U 1 0.026

TUSCHL T 1 0.026

TUTTLE K 1 0.026

TUYISHIME F 1 0.026

TWIGG S 1 0.026

TYAGI N 1 0.026

TZENG YM 1 0.026

UBUKATA N 1 0.026

UCAR R 1 0.026

UCERO AC 1 0.026

UCHIDA HA 1 0.026

UCHIDA S 1 0.026

UCHIDA T 1 0.026

UCHII M 1 0.026

UDELL JA 1 0.026

UEDA H 1 0.026

UEDA K 1 0.026

UEDA M 1 0.026

UEDA N 1 0.026

UEKAWA K 1 0.026

UELAND T 1 0.026

UEMURA Y 1 0.026

UESHIMA K 1 0.026

UGAWA T 1 0.026

UGI S 1 0.026

UHLES S 1 0.026

UHLIG P 1 0.026

UIJL E 1 0.026

UKKOLA O 1 0.026

ULASOVA E 1 0.026

ULLRICH S 1 0.026

ULUSU NN 1 0.026

UM JE 1 0.026

UMEMURA A 1 0.026

UMESHITA K 1 0.026

UMEZAWA K 1 0.026

UMEZAWA M 1 0.026

UMEZONO T 1 0.026

UNE S 1 0.026

UNIYAL S 1 0.026

UNLU SM 1 0.026

UNWIN RJ 1 0.026

UPADHYAY A 1 0.026

URADE Y 1 0.026

URBANEK A 1 0.026

URBANEK C 1 0.026

URENA P 1 0.026

URIBE N 1 0.026

URQUILLA PR 1 0.026

USA K 1 0.026

USINGER W 1 0.026

USINGER WR 1 0.026

USMAN AA 1 0.026

USTUN I 1 0.026

USUDA N 1 0.026

USUI J 1 0.026

USUI K 1 0.026

UTO H 1 0.026

UTO K 1 0.026

UTO T 1 0.026

UTSUMI H 1 0.026

UTSUNOMIYA Y 1 0.026

UYY E 1 0.026

UZELI S 1 0.026

VADIVEL N 1 0.026

VAGO L 1 0.026

VAHANIAN A 1 0.026

VAHED SZ 1 0.026

VAINGANKAR S 1 0.026

VAJIR M 1 0.026

VALA H 1 0.026

VALASTRO B 1 0.026

VALENTE CP 1 0.026

VALENTINE RJ 1 0.026

VALET P 1 0.026

VALIATTI MF 1 0.026

VALLA D 1 0.026

VALLE SOF 1 0.026

VALLEE M 1 0.026

VALVERDE AM 1 0.026

VAN BEZU J 1 0.026

VAN BIESEN W 1 0.026

VAN BILSEN M 1 0.026

VAN BOMMEL RJ 1 0.026

VAN BUITEN A 1 0.026

VAN DE WOUW J 1 0.026

VAN DEN BERG BM 1 0.026

VAN DEN BERG E 1 0.026

VAN DEN BERG JMW 1 0.026

VAN DEN DORPEL MA 1 0.026

VAN DEN HEUVEL LP 1 0.026

VAN DEN HEUVEL LPWJ 1 0.026

VAN DEN HOFF MJB 1 0.026

VAN DEN MEIRACKER AH 1 0.026

VAN DER HAUWAERT C 1 0.026

VAN DER HORST G 1 0.026

VAN DER MEULEN EF 1 0.026

VAN DER PIJL JW 1 0.026

VAN DER PLUIJM G 1 0.026

VAN DER POL P 1 0.026

VAN DER PUTTEN K 1 0.026

VAN DER VELDE ET 1 0.026

VAN DER VORST EPC 1 0.026

VAN DER WOUDE FJ 1 0.026

VAN DIJK CGM 1 0.026

VAN DINTHER M 1 0.026

VAN DOKKUM RPE 1 0.026

VAN DUIN RWB 1 0.026

VAN ERVEN L 1 0.026

VAN GEEST RJ 1 0.026

VAN GEUNS RJ 1 0.026

VAN HEEREBEEK L 1 0.026

VAN JAD 1 0.026

VAN KRANENBURG M 1 0.026

VAN KRIEKEN R 1 0.026

VAN LANDSCHOOT M 1 0.026

VAN LONDEN M 1 0.026

VAN MOURIK I 1 0.026

VAN NOORDEN CJF 1 0.026

VAN OOSTROM O 1 0.026

VAN PAASSEN P 1 0.026

VAN RAALTE DH 1 0.026

VAN REMMEN H 1 0.026

VAN ROEYEN C 1 0.026

VAN TASSELL BW 1 0.026

VAN TEEFFELEN JWGE 1 0.026

VAN VEELEN PA 1 0.026

VAN VEEN I 1 0.026

VANBECKEVOORT D 1 0.026

VANDEKERCKHOVE L 1 0.026

VANDERLEI LA 1 0.026

VANECKOVA I 1 0.026

VANHERWEGHEM JL 1 0.026

VANHILLE P 1 0.026

VANHOVE T 1 0.026

VANKRIEKEN R 1 0.026

VARA-MESSLER M 1 0.026

VARAGIC J 1 0.026

VARANI J 1 0.026

VASAN RS 1 0.026

VASILOPOULOU E 1 0.026

VASILUTA L 1 0.026

VASKO R 1 0.026

VASSILIADOU A 1 0.026

VAUGHAN DE 1 0.026

VAUGHAN ED 1 0.026

VAVRINEC P 1 0.026

VAYSSAIRAT M 1 0.026

VAZQUEZ A 1 0.026

VAZQUEZ-GOMEZ M 1 0.026

VAZQUEZ-LA MADRID J 1 0.026

VAZQUEZ-MARTIN A 1 0.026

VEELKEN R 1 0.026

VEERAVEEDU PT 1 0.026

VEGA G 1 0.026

VEGA-GOMEZ H 1 0.026

VEGLIA E 1 0.026

VEGLIO F 1 0.026

VEIRAS LC 1 0.026

VEKEMANS M 1 0.026

VELASQUEZ MT 1 0.026

VELEZ JCQ 1 0.026

VELKOSKA E 1 0.026

VELLA J 1 0.026

VELTHUIS BK 1 0.026

VELUSAMY P 1 0.026

VEMURI K 1 0.026

VEMURI VK 1 0.026

VENKAT V 1 0.026

VENKATACHALAM M 1 0.026

VENKATESAN S 1 0.026

VENKATESWARAMURTHY N 1 0.026

VENKITACHALAM L 1 0.026

VERA M 1 0.026

VERBEUREN TJ 1 0.026

VERDALLES U 1 0.026

VERDE E 1 0.026

VERDONSCHOT J 1 0.026

VERES-SZEKELY A 1 0.026

VERGELY C 1 0.026

VERHAMME P 1 0.026

VERHEYEN N 1 0.026

VERJANS R 1 0.026

VERKMAN AS 1 0.026

VERLANDER JW 1 0.026

VERMA S 1 0.026

VERMEULEN Z 1 0.026

VERNEROVA Z 1 0.026

VERNET A 1 0.026

VERNET D 1 0.026

VERNOCHET A 1 0.026

VERONESE C 1 0.026

VERRIJKEN A 1 0.026

VERROUST PJ 1 0.026

VERRY P 1 0.026

VERSTEEG EMM 1 0.026

VERTY ANA 1 0.026

VERVAET B 1 0.026

VERVAET BA 1 0.026

VERZIEUX V 1 0.026

VESCOVO G 1 0.026

VESEY DA 1 0.026

VESSAL G 1 0.026

VETHAKKAN SR 1 0.026

VETTOR R 1 0.026

VIAL C 1 0.026

VIANA GN 1 0.026

VIANELLO D 1 0.026

VIAU A 1 0.026

VIBERTI G 1 0.026

VIENNE AR 1 0.026

VIERKOTTER A 1 0.026

VIGANO S 1 0.026

VIGGIANO D 1 0.026

VIGOTTI FN 1 0.026

VIINIKKA L 1 0.026

VIJAYARAJ S 1 0.026

VIKLICKY O 1 0.026

VILAR MJP 1 0.026

VILAYUR E 1 0.026

VILLA M 1 0.026

VILLA S 1 0.026

VILLABLANCA C 1 0.026

VILLANUEVA-MILLAN MJ 1 0.026

VILLARREAL F 1 0.026

VILLAVICENCIO MS 1 0.026

VINCIGUERRA M 1 0.026

VINK H 1 0.026

VINOGRADOV IV 1 0.026

VIO CP 1 0.026

VIOLETTE SM 1 0.026

VIOLLET B 1 0.026

VIONNET N 1 0.026

VIRZI GM 1 0.026

VISALLI G 1 0.026

VISCONTI L 1 0.026

VISNAR-PEROVIC A 1 0.026

VISWANADHAPALLI S 1 0.026

VISWANATHAN V 1 0.026

VIVIER PH 1 0.026

VJECHA MJ 1 0.026

VLODAVSKY I 1 0.026

VOCINO G 1 0.026

VOELKL J 1 0.026

VOGELBACHER R 1 0.026

VOGT L 1 0.026

VOIGT S 1 0.026

VOLK HD 1 0.026

VOLLBORT A 1 0.026

VOLOSENCO I 1 0.026

VOLPINI RA 1 0.026

VOLTAN R 1 0.026

VOLZKE H 1 0.026

VON EYNATTEN M 1 0.026

VON LEWINSKI D 1 0.026

VON LUEDER TG 1 0.026

VON SCHOLTEN BJ 1 0.026

VON STILLFRIED S 1 0.026

VON WEYHERN CWH 1 0.026

VON WICHMANN MA 1 0.026

VONGISE H 1 0.026

VORA A 1 0.026

VOS FE 1 0.026

VOSKOBOEV NV 1 0.026

VOSSOUGHI M 1 0.026

VOZIYAN P 1 0.026

VRIESMAN PV 1 0.026

VURBIC D 1 0.026

WAASDORP M 1 0.026

WACKER F 1 0.026

WADA K 1 0.026

WADA Y 1 0.026

WADEI HM 1 0.026

WADEN J 1 0.026

WAFA EW 1 0.026

WAGGOTT D 1 0.026

WAGNER B 1 0.026

WAGNER J 1 0.026

WAGNER L 1 0.026

WAGNER LJ 1 0.026

WAGNER Z 1 0.026

WAGURI M 1 0.026

WAHAB NA 1 0.026

WAHL P 1 0.026

WAKEFIELD AJ 1 0.026

WAKEFIELD J 1 0.026

WAKEFIELD JD 1 0.026

WAKINO S 1 0.026

WAKISAKA M 1 0.026

WAKITA T 1 0.026

WALANA W 1 0.026

WALES JK 1 0.026

WALES JKH 1 0.026

WALKER HJ 1 0.026

WALKER JP 1 0.026

WALL BM 1 0.026

WALL MJ 1 0.026

WALLACE DP 1 0.026

WALLACE KJ 1 0.026

WALLACE S 1 0.026

WALLER JR 1 0.026

WALLIA A 1 0.026

WALLNER EI 1 0.026

WALLNER M 1 0.026

WALMSLEY RS 1 0.026

WALSH JL 1 0.026

WALTON C 1 0.026

WALZ K 1 0.026

WAN C 1 0.026

WAN JD 1 0.026

WAN JN 1 0.026

WAN LW 1 0.026

WAN QJ 1 0.026

WAN Y 1 0.026

WANCHAI K 1 0.026

WANG BD 1 0.026

WANG CF 1 0.026

WANG CP 1 0.026

WANG DC 1 0.026

WANG DH 1 0.026

WANG DJ 1 0.026

WANG DJR 1 0.026

WANG DN 1 0.026

WANG DP 1 0.026

WANG DT 1 0.026

WANG EY 1 0.026

WANG FF 1 0.026

WANG FZ 1 0.026

WANG GB 1 0.026

WANG GD 1 0.026

WANG GF 1 0.026

WANG GG 1 0.026

WANG GH 1 0.026

WANG GQ 1 0.026

WANG GS 1 0.026

WANG GX 1 0.026

WANG HL 1 0.026

WANG HM 1 0.026

WANG HT 1 0.026

WANG JK 1 0.026

WANG JM 1 0.026

WANG KA 1 0.026

WANG KF 1 0.026

WANG KJ 1 0.026

WANG KY 1 0.026

WANG LA 1 0.026

WANG LK 1 0.026

WANG LT 1 0.026

WANG LX 1 0.026

WANG MB 1 0.026

WANG MC 1 0.026

WANG MDXL 1 0.026

WANG MG 1 0.026

WANG MJ 1 0.026

WANG MR 1 0.026

WANG N 1 0.026

WANG NN 1 0.026

WANG PJ 1 0.026

WANG PR 1 0.026

WANG QW 1 0.026

WANG RJ 1 0.026

WANG RQ 1 0.026

WANG RY 1 0.026

WANG ST 1 0.026

WANG SZ 1 0.026

WANG TF 1 0.026

WANG TT 1 0.026

WANG WG 1 0.026

WANG WM 1 0.026

WANG WQ 1 0.026

WANG XA 1 0.026

WANG XC 1 0.026

WANG XD 1 0.026

WANG XF 1 0.026

WANG XK 1 0.026

WANG XN 1 0.026

WANG XNH 1 0.026

WANG XW 1 0.026

WANG YC 1 0.026

WANG YK 1 0.026

WANG YS 1 0.026

WANG ZB 1 0.026

WANG ZF 1 0.026

WANG ZK 1 0.026

WANG ZL 1 0.026

WANG ZS 1 0.026

WANG ZX 1 0.026

WANNER N 1 0.026

WAQAR T 1 0.026

WARD EV 1 0.026

WARD J 1 0.026

WARDLE EN 1 0.026

WARNER GM 1 0.026

WARNER JT 1 0.026

WARRAM JH 1 0.026

WARREN AM 1 0.026

WARREN W 1 0.026

WARWICK J 1 0.026

WASIAK D 1 0.026

WASSELL R 1 0.026

WASSERFALL C 1 0.026

WATANABE D 1 0.026

WATANABE E 1 0.026

WATANABE J 1 0.026

WATSON CJ 1 0.026

WATSON D 1 0.026

WATSON P 1 0.026

WATSON S 1 0.026

WAUQUIER F 1 0.026

WAZNA E 1 0.026

WEBB DJ 1 0.026

WEBB M 1 0.026

WEBBER J 1 0.026

WEBER FL 1 0.026

WEBER G 1 0.026

WEBER S 1 0.026

WEBSTER A 1 0.026

WEBSTER GJ 1 0.026

WEDEMEYER H 1 0.026

WEGNER W 1 0.026

WEI B 1 0.026

WEI CC 1 0.026

WEI G 1 0.026

WEI GH 1 0.026

WEI GW 1 0.026

WEI GZ 1 0.026

WEI HL 1 0.026

WEI KS 1 0.026

WEI L 1 0.026

WEI LH 1 0.026

WEI LL 1 0.026

WEI LT 1 0.026

WEI MG 1 0.026

WEI PZ 1 0.026

WEI RM 1 0.026

WEI W 1 0.026

WEI X 1 0.026

WEI XH 1 0.026

WEI XJ 1 0.026

WEI XW 1 0.026

WEI Y 1 0.026

WEI YQ 1 0.026

WEI ZC 1 0.026

WEI ZG 1 0.026

WEI ZH 1 0.026

WEI ZP 1 0.026

WEIDEKAMM C 1 0.026

WEIDEMANN F 1 0.026

WEIDENBUSCH M 1 0.026

WEIER Q 1 0.026

WEIL EJ 1 0.026

WEIL J 1 0.026

WEILER HA 1 0.026

WEILL J 1 0.026

WEIMBS T 1 0.026

WEINBERG JM 1 0.026

WEINERT LS 1 0.026

WEINMANN-MENKE J 1 0.026

WEINRAUCH LA 1 0.026

WEINREB PH 1 0.026

WEINSTEIN T 1 0.026

WEINTRAUB NL 1 0.026

WEISBERG A 1 0.026

WEISS MF 1 0.026

WEISSMANN N 1 0.026

WELBOURNE TC 1 0.026

WELLSTEIN A 1 0.026

WELSH N 1 0.026

WELZEL TM 1 0.026

WEN DH 1 0.026

WEN J 1 0.026

WEN JH 1 0.026

WEN JM 1 0.026

WEN L 1 0.026

WEN MC 1 0.026

WEN Q 1 0.026

WEN R 1 0.026

WEN Y 1 0.026

WEN ZH 1 0.026

WENDT R 1 0.026

WENG CH 1 0.026

WENG GB 1 0.026

WENG H 1 0.026

WENG HC 1 0.026

WENG L 1 0.026

WENG QY 1 0.026

WENG XL 1 0.026

WENG Y 1 0.026

WENGER SL 1 0.026

WERDIN E 1 0.026

WERZOWA J 1 0.026

WEST A 1 0.026

WEST M 1 0.026

WESTALL GP 1 0.026

WESTERMANN D 1 0.026

WESTON BS 1 0.026

WETSEL RA 1 0.026

WETZEL MD 1 0.026

WETZELS JFM 1 0.026

WETZL V 1 0.026

WHEELER-JONES C 1 0.026

WHELTON P 1 0.026

WHITE MF 1 0.026

WHITLEY EM 1 0.026

WHITNEY JL 1 0.026

WHITTIER F 1 0.026

WICKENHAUSER C 1 0.026

WICKS SE 1 0.026

WICKSTROM SA 1 0.026

WIECH T 1 0.026

WIEDMEYER C 1 0.026

WIELINGA PY 1 0.026

WIELOPOLSKI PA 1 0.026

WIERSINGA WM 1 0.026

WIERZBICKI Z 1 0.026

WIESINGER M 1 0.026

WIEST G 1 0.026

WIETECHA T 1 0.026

WIETEN L 1 0.026

WIGG B 1 0.026

WIGGINS KJ 1 0.026

WILCK N 1 0.026

WILD G 1 0.026

WILDER SP 1 0.026

WILKENING A 1 0.026

WILKES MC 1 0.026

WILKINSON IB 1 0.026

WILKINSON P 1 0.026

WILKINSON PF 1 0.026

WILLARS GB 1 0.026

WILLEMSEN B 1 0.026

WILLEMSEN S 1 0.026

WILLIAMS AM 1 0.026

WILLIAMS B 1 0.026

WILLIAMS BM 1 0.026

WILLIAMS I 1 0.026

WILLIAMS L 1 0.026

WILLIAMS VR 1 0.026

WILLIAMS WW 1 0.026

WILLIS AM 1 0.026

WILLMES C 1 0.026

WILLNER J 1 0.026

WILSON DI 1 0.026

WILSON DW 1 0.026

WILSON JM 1 0.026

WILSON JW 1 0.026

WILSON P 1 0.026

WILSON SG 1 0.026

WILSON T 1 0.026

WINAVER J 1 0.026

WINBANKS C 1 0.026

WINCHESTER R 1 0.026

WINER D 1 0.026

WING L 1 0.026

WING MR 1 0.026

WINKLER C 1 0.026

WINN SK 1 0.026

WINNICKI W 1 0.026

WINTER M 1 0.026

WINTOUR EM 1 0.026

WINYARD PJ 1 0.026

WISE AF 1 0.026

WISE S 1 0.026

WISECUP E 1 0.026

WITHER J 1 0.026

WITTMANN I 1 0.026

WITTMANN M 1 0.026

WITTMANN S 1 0.026

WLAZEL RN 1 0.026

WLODKOWSKI T 1 0.026

WOHLMUTH H 1 0.026

WOJCIK JL 1 0.026

WOJCIKOWSKI K 1 0.026

WOLF JS 1 0.026

WOLTERS AAB 1 0.026

WONG AKM 1 0.026

WONG CP 1 0.026

WONG GLH 1 0.026

WONG J 1 0.026

WONG JKW 1 0.026

WONG JS 1 0.026

WONG LL 1 0.026

WONG MH 1 0.026

WONG PN 1 0.026

WONG RB 1 0.026

WONG RJ 1 0.026

WONG SE 1 0.026

WONG TYH 1 0.026

WONG VWS 1 0.026

WONG Y 1 0.026

WONG YY 1 0.026

WONGJARUPONG A 1 0.026

WOO D 1 0.026

WOO HI 1 0.026

WOO V 1 0.026

WOOD G 1 0.026

WOODMAN RJ 1 0.026

WOODROFFE RC 1 0.026

WOODRUFF TM 1 0.026

WOODS A 1 0.026

WOODS TC 1 0.026

WOODSON M 1 0.026

WOON PY 1 0.026

WORMLEIGHTON JV 1 0.026

WORZFELD T 1 0.026

WOZNIAK TC 1 0.026

WRIGHT JT 1 0.026

WRIGHT JW 1 0.026

WRIGHT MB 1 0.026

WU AM 1 0.026

WU CD 1 0.026

WU CW 1 0.026

WU CZ 1 0.026

WU DC 1 0.026

WU DD 1 0.026

WU DF 1 0.026

WU DK 1 0.026

WU DM 1 0.026

WU DP 1 0.026

WU DQ 1 0.026

WU FJ 1 0.026

WU FR 1 0.026

WU HF 1 0.026

WU HH 1 0.026

WU HM 1 0.026

WU HY 1 0.026

WU IH 1 0.026

WU JL 1 0.026

WU JP 1 0.026

WU JZ 1 0.026

WU LY 1 0.026

WU MM 1 0.026

WU MT 1 0.026

WU N 1 0.026

WU Q 1 0.026

WU QH 1 0.026

WU QJ 1 0.026

WU QW 1 0.026

WU QY 1 0.026

WU R 1 0.026

WU SF 1 0.026

WU SH 1 0.026

WU SJ 1 0.026

WU SK 1 0.026

WU SL 1 0.026

WU SP 1 0.026

WU SS 1 0.026

WU SW 1 0.026

WU SY 1 0.026

WU SZ 1 0.026

WU T 1 0.026

WU WL 1 0.026

WU WM 1 0.026

WU WP 1 0.026

WU WW 1 0.026

WU XG 1 0.026

WU XH 1 0.026

WU XL 1 0.026

WU XX 1 0.026

WU YJ 1 0.026

WU YL 1 0.026

WU YQ 1 0.026

WU YR 1 0.026

WU YW 1 0.026

WU ZG 1 0.026

WU ZK 1 0.026

WUETHRICH RP 1 0.026

WULFF X 1 0.026

WURPS H 1 0.026

WUTHRICH R 1 0.026

WUTHRICH RP 1 0.026

WYATT AW 1 0.026

WYATT J 1 0.026

WYGODA S 1 0.026

WYSOCKI J 1 0.026

XI L 1 0.026

XI LQ 1 0.026

XI YY 1 0.026

XIA CH 1 0.026

XIA CK 1 0.026

XIA F 1 0.026

XIA HL 1 0.026

XIA HM 1 0.026

XIA JJ 1 0.026

XIA JL 1 0.026

XIA LP 1 0.026

XIA M 1 0.026

XIA VW 1 0.026

XIA WK 1 0.026

XIA WP 1 0.026

XIA X 1 0.026

XIA XF 1 0.026

XIA XX 1 0.026

XIA YG 1 0.026

XIA ZN 1 0.026

XIANG E 1 0.026

XIANG HD 1 0.026

XIANG HL 1 0.026

XIANG XY 1 0.026

XIANG YX 1 0.026

XIAO H 1 0.026

XIAO HS 1 0.026

XIAO LT 1 0.026

XIAO LX 1 0.026

XIAO QH 1 0.026

XIAO SG 1 0.026

XIAO SY 1 0.026

XIAO T 1 0.026

XIAO TC 1 0.026

XIAO WZ 1 0.026

XIAO X 1 0.026

XIAO XH 1 0.026

XIAO XQ 1 0.026

XIAO YL 1 0.026

XIAO YY 1 0.026

XIAO Z 1 0.026

XIAO ZG 1 0.026

XIAO ZZ 1 0.026

XIE C 1 0.026

XIE CH 1 0.026

XIE CS 1 0.026

XIE CY 1 0.026

XIE DH 1 0.026

XIE DW 1 0.026

XIE FF 1 0.026

XIE FJ 1 0.026

XIE FY 1 0.026

XIE H 1 0.026

XIE HL 1 0.026

XIE J 1 0.026

XIE JJ 1 0.026

XIE JM 1 0.026

XIE JQ 1 0.026

XIE LL 1 0.026

XIE LY 1 0.026

XIE MY 1 0.026

XIE QH 1 0.026

XIE QY 1 0.026

XIE SS 1 0.026

XIE WB 1 0.026

XIE XC 1 0.026

XIE XQ 1 0.026

XIE XS 1 0.026

XIE YJ 1 0.026

XIE ZC 1 0.026

XIELIFU RH 1 0.026

XIN CW 1 0.026

XIN CY 1 0.026

XIN GD 1 0.026

XIN JL 1 0.026

XIN R 1 0.026

XIN X 1 0.026

XIN Y 1 0.026

XING CJ 1 0.026

XING LL 1 0.026

XING M 1 0.026

XING QQ 1 0.026

XING T 1 0.026

XING W 1 0.026

XING X 1 0.026

XING XS 1 0.026

XING YF 1 0.026

XING YH 1 0.026

XIONG C 1 0.026

XIONG CX 1 0.026

XIONG F 1 0.026

XIONG G 1 0.026

XIONG JR 1 0.026

XIONG LY 1 0.026

XIONG PH 1 0.026

XIONG SX 1 0.026

XIONG W 1 0.026

XIONG XP 1 0.026

XIONG Y 1 0.026

XIONG YW 1 0.026

XIONG Z 1 0.026

XIOUG MQ 1 0.026

XU BH 1 0.026

XU BK 1 0.026

XU CF 1 0.026

XU CQ 1 0.026

XU CY 1 0.026

XU DY 1 0.026

XU FF 1 0.026

XU FT 1 0.026

XU FX 1 0.026

XU HL 1 0.026

XU HP 1 0.026

XU HR 1 0.026

XU HW 1 0.026

XU HY 1 0.026

XU JC 1 0.026

XU KC 1 0.026

XU KY 1 0.026

XU KZY 1 0.026

XU LH 1 0.026

XU LL 1 0.026

XU LZ 1 0.026

XU MJ 1 0.026

XU MR 1 0.026

XU N 1 0.026

XU PJ 1 0.026

XU R 1 0.026

XU RC 1 0.026

XU RF 1 0.026

XU RH 1 0.026

XU RS 1 0.026

XU SQ 1 0.026

XU SS 1 0.026

XU SZ 1 0.026

XU TH 1 0.026

XU W 1 0.026

XU WJ 1 0.026

XU WW 1 0.026

XU XB 1 0.026

XU XH 1 0.026

XU XP 1 0.026

XU XY 1 0.026

XU XZ 1 0.026

XU YH 1 0.026

XU YJ 1 0.026

XU YK 1 0.026

XU YL 1 0.026

XU YW 1 0.026

XU YY 1 0.026

XU ZC 1 0.026

XU ZF 1 0.026

XU ZM 1 0.026

XU ZR 1 0.026

XUAN C 1 0.026

XUAN W 1 0.026

XUAN XY 1 0.026

XUE FP 1 0.026

XUE JY 1 0.026

XUE L 1 0.026

XUE MJ 1 0.026

XUE NN 1 0.026

XUE R 1 0.026

XYDAKIS AM 1 0.026

YADAV H 1 0.026

YADAV S 1 0.026

YADAV SP 1 0.026

YAGHOBIAN D 1 0.026

YAGHOBIAN S 1 0.026

YAGMUR E 1 0.026

YAHEFU R 1 0.026

YAHIAOUI Y 1 0.026

YAKALA GK 1 0.026

YAKES M 1 0.026

YALAMANCHILI HB 1 0.026

YALCIN KS 1 0.026

YAM I 1 0.026

YAMABE N 1 0.026

YAMADA K 1 0.026

YAMADA S 1 0.026

YAMADA T 1 0.026

YAMADA-OBARA N 1 0.026

YAMAGISHI SI 1 0.026

YAMAGUCHI I 1 0.026

YAMAGUCHI J 1 0.026

YAMAGUCHI K 1 0.026

YAMAGUCHI T 1 0.026

YAMAJI Y 1 0.026

YAMAKAGE S 1 0.026

YAMAMOTO N 1 0.026

YAMAMOTO O 1 0.026

YAMAMOTOYA T 1 0.026

YAMAMURA Y 1 0.026

YAMANAKA S 1 0.026

YAMANE K 1 0.026

YAMANI A 1 0.026

YAMAOKA S 1 0.026

YAMASAKI M 1 0.026

YAMASAKI S 1 0.026

YAMASHITA H 1 0.026

YAMATO O 1 0.026

YAMAUCHI H 1 0.026

YAMAYA H 1 0.026

YAMINI D 1 0.026

YAMMINE L 1 0.026

YAN BP 1 0.026

YAN CC 1 0.026

YAN CD 1 0.026

YAN CF 1 0.026

YAN HH 1 0.026

YAN HM 1 0.026

YAN HY 1 0.026

YAN JQ 1 0.026

YAN L 1 0.026

YAN LL 1 0.026

YAN RH 1 0.026

YAN RJ 1 0.026

YAN ST 1 0.026

YAN TX 1 0.026

YAN WZ 1 0.026

YAN XF 1 0.026

YAN XH 1 0.026

YAN Y 1 0.026

YAN YS 1 0.026

YAN YX 1 0.026

YAN YY 1 0.026

YAN ZB 1 0.026

YAN ZC 1 0.026

YAN ZX 1 0.026

YANAGAWA N 1 0.026

YANAGIDA K 1 0.026

YANAGIHARA N 1 0.026

YANAGITA S 1 0.026

YANAI M 1 0.026

YANASE N 1 0.026

YANEZ AJ 1 0.026

YANG BF 1 0.026

YANG BX 1 0.026

YANG CH 1 0.026

YANG CL 1 0.026

YANG CQ 1 0.026

YANG CT 1 0.026

YANG CX 1 0.026

YANG CY 1 0.026

YANG FY 1 0.026

YANG GN 1 0.026

YANG GZ 1 0.026

YANG HJ 1 0.026

YANG HL 1 0.026

YANG HM 1 0.026

YANG JB 1 0.026

YANG JC 1 0.026

YANG JD 1 0.026

YANG JH 1 0.026

YANG JJ 1 0.026

YANG JL 1 0.026

YANG JR 1 0.026

YANG JZ 1 0.026

YANG LB 1 0.026

YANG LL 1 0.026

YANG LN 1 0.026

YANG LP 1 0.026

YANG LQ 1 0.026

YANG LT 1 0.026

YANG ML 1 0.026

YANG PY 1 0.026

YANG QC 1 0.026

YANG QL 1 0.026

YANG QM 1 0.026

YANG QQ 1 0.026

YANG QY 1 0.026

YANG RH 1 0.026

YANG SJ 1 0.026

YANG SM 1 0.026

YANG SS 1 0.026

YANG SX 1 0.026

YANG SZ 1 0.026

YANG TX 1 0.026

YANG WL 1 0.026

YANG WQ 1 0.026

YANG WW 1 0.026

YANG WZ 1 0.026

YANG XH 1 0.026

YANG XJ 1 0.026

YANG XK 1 0.026

YANG XS 1 0.026

YANG XW 1 0.026

YANG YJ 1 0.026

YANG YM 1 0.026

YANG YR 1 0.026

YANG YT 1 0.026

YANG Z 1 0.026

YANG ZC 1 0.026

YANG ZF 1 0.026

YANG ZL 1 0.026

YANG ZN 1 0.026

YANG ZX 1 0.026

YANIKKAYA G 1 0.026

YANO K 1 0.026

YANO S 1 0.026

YANO Y 1 0.026

YAO DY 1 0.026

YAO G 1 0.026

YAO GH 1 0.026

YAO H 1 0.026

YAO K 1 0.026

YAO PB 1 0.026

YAO QS 1 0.026

YAO T 1 0.026

YAO W 1 0.026

YAO YF 1 0.026

YAO YM 1 0.026

YAO YZ 1 0.026

YARBROUGH B 1 0.026

YARD BA 1 0.026

YARDE M 1 0.026

YASUDA H 1 0.026

YASUDA Y 1 0.026

YASUKAWA H 1 0.026

YASUMURA S 1 0.026

YASUNO S 1 0.026

YATABE J 1 0.026

YATABE MS 1 0.026

YAVARI V 1 0.026

YAVUZ A 1 0.026

YAZDIAN-ROBATI R 1 0.026

YE B 1 0.026

YE CY 1 0.026

YE DQ 1 0.026

YE F 1 0.026

YE HY 1 0.026

YE JM 1 0.026

YE K 1 0.026

YE L 1 0.026

YE LF 1 0.026

YE SD 1 0.026

YE SQ 1 0.026

YE XY 1 0.026

YE Y 1 0.026

YE YL 1 0.026

YE YZ 1 0.026

YE Z 1 0.026

YE ZC 1 0.026

YE ZJ 1 0.026

YE ZL 1 0.026

YEE AJ 1 0.026

YEE HY 1 0.026

YEE J 1 0.026

YEH CH 1 0.026

YEH KT 1 0.026

YEH SM 1 0.026

YEH YC 1 0.026

YEN CL 1 0.026

YEN MH 1 0.026

YEN TH 1 0.026

YENEN E 1 0.026

YENGE G 1 0.026

YEO KM 1 0.026

YERRAM P 1 0.026

YESHUA H 1 0.026

YEUNG MW 1 0.026

YEUNG SP 1 0.026

YI C 1 0.026

YI LN 1 0.026

YI R 1 0.026

YI ZC 1 0.026

YI ZZ 1 0.026

YILDIRIM Y 1 0.026

YILDIRIM ZY 1 0.026

YILDIZ A 1 0.026

YILMAZ A 1 0.026

YILMAZ F 1 0.026

YILMAZ H 1 0.026

YILMAZ MI 1 0.026

YILMAZ Y 1 0.026

YILMAZ-GULEC E 1 0.026

YIN D 1 0.026

YIN DD 1 0.026

YIN DK 1 0.026

YIN HQ 1 0.026

YIN JL 1 0.026

YIN JM 1 0.026

YIN JY 1 0.026

YIN LN 1 0.026

YIN LP 1 0.026

YIN LT 1 0.026

YIN MC 1 0.026

YIN P 1 0.026

YIN SH 1 0.026

YIN WD 1 0.026

YIN WJ 1 0.026

YIN WQ 1 0.026

YIN X 1 0.026

YIN XH 1 0.026

YIN XN 1 0.026

YIN ZK 1 0.026

YIN ZM 1 0.026

YIN ZQ 1 0.026

YIN ZW 1 0.026

YIN ZY 1 0.026

YING GQ 1 0.026

YING K 1 0.026

YIP K 1 0.026

YLIKORKALA O 1 0.026

YOGI A 1 0.026

YOGO K 1 0.026

YOKAWA T 1 0.026

YOKOMAKU Y 1 0.026

YOKOO T 1 0.026

YOKOTE S 1 0.026

YOKOYAMA T 1 0.026

YOKOYAMA Y 1 0.026

YONEKI Y 1 0.026

YONEKURA H 1 0.026

YONEKURA Y 1 0.026

YONG R 1 0.026

YOO J 1 0.026

YOO KD 1 0.026

YOO YH 1 0.026

YOON BH 1 0.026

YOON CS 1 0.026

YOON D 1 0.026

YOON HK 1 0.026

YOON J 1 0.026

YOON KC 1 0.026

YOON SK 1 0.026

YOON YM 1 0.026

YORIOKA N 1 0.026

YOSHI S 1 0.026

YOSHIDA K 1 0.026

YOSHIDA S 1 0.026

YOSHIHARA D 1 0.026

YOSHIHARA F 1 0.026

YOSHIKAWA N 1 0.026

YOSHIMINE Y 1 0.026

YOSHIMORI T 1 0.026

YOSHIMOTO T 1 0.026

YOSHIMURA A 1 0.026

YOSHINAGA K 1 0.026

YOSHIZAKI A 1 0.026

YOSHIZAWA H 1 0.026

YOSHIZURMI M 1 0.026

YOU CQ 1 0.026

YOU L 1 0.026

YOU YH 1 0.026

YOU YT 1 0.026

YOUM JB 1 0.026

YOUNIS NN 1 0.026

YOUNOSSI ZM 1 0.026

YOUNOSZAI Z 1 0.026

YTTERBERG SR 1 0.026

YU B 1 0.026

YU BC 1 0.026

YU BY 1 0.026

YU CC 1 0.026

YU CH 1 0.026

YU CJ 1 0.026

YU CL 1 0.026

YU CP 1 0.026

YU G 1 0.026

YU HJ 1 0.026

YU HL 1 0.026

YU HR 1 0.026

YU HT 1 0.026

YU HW 1 0.026

YU HY 1 0.026

YU JH 1 0.026

YU JS 1 0.026

YU KS 1 0.026

YU LH 1 0.026

YU LJ 1 0.026

YU M 1 0.026

YU MX 1 0.026

YU MY 1 0.026

YU N 1 0.026

YU P 1 0.026

YU PF 1 0.026

YU Q 1 0.026

YU SH 1 0.026

YU SJ 1 0.026

YU SN 1 0.026

YU SQ 1 0.026

YU SY 1 0.026

YU TH 1 0.026

YU TT 1 0.026

YU WM 1 0.026

YU XC 1 0.026

YU XL 1 0.026

YU YH 1 0.026

YU YT 1 0.026

YUAN FH 1 0.026

YUAN JJ 1 0.026

YUAN JY 1 0.026

YUAN P 1 0.026

YUAN RR 1 0.026

YUAN RY 1 0.026

YUAN SG 1 0.026

YUAN WD 1 0.026

YUAN WJ 1 0.026

YUAN WP 1 0.026

YUAN Y 1 0.026

YUAN YC 1 0.026

YUAN YJ 1 0.026

YUAN ZP 1 0.026

YUANA Y 1 0.026

YUASA D 1 0.026

YUE DK 1 0.026

YUE RZ 1 0.026

YUE SL 1 0.026

YUE XD 1 0.026

YUE Y 1 0.026

YULDIZ M 1 0.026

YUMURA W 1 0.026

YUN SH 1 0.026

YUN SP 1 0.026

YUNG S 1 0.026

YUNG SS 1 0.026

YUREKLI B 1 0.026

YURTTUTAN N 1 0.026

YUSOFF FM 1 0.026

YUSUFI ANK 1 0.026

ZABAD OM 1 0.026

ZABOLIAN A 1 0.026

ZACCHIA M 1 0.026

ZACH MS 1 0.026

ZAFIRIOU S 1 0.026

ZAGHLOUL MS 1 0.026

ZAHARIS CZ 1 0.026

ZAHER WA 1 0.026

ZAHRADKA P 1 0.026

ZAHRAN A 1 0.026

ZAI WJ 1 0.026

ZAKARIA EM 1 0.026

ZALAZAR A 1 0.026

ZAMAMI Y 1 0.026

ZAMANI P 1 0.026

ZAMILA M 1 0.026

ZAMORA L 1 0.026

ZAMYATKIN O 1 0.026

ZANCHI C 1 0.026

ZANDI-NEJAD K 1 0.026

ZANDVOORT A 1 0.026

ZANG GX 1 0.026

ZANG J 1 0.026

ZANG JT 1 0.026

ZANG WJ 1 0.026

ZANG YC 1 0.026

ZANG YH 1 0.026

ZANGEMEISTER-WITTKE U 1 0.026

ZANNINI L 1 0.026

ZANONI JN 1 0.026

ZAOUI P 1 0.026

ZARGAR H 1 0.026

ZARGHAMI N 1 0.026

ZARJOU A 1 0.026

ZARRABI A 1 0.026

ZAULI G 1 0.026

ZAVADIL J 1 0.026

ZAWAIDEH MA 1 0.026

ZAYERI ZD 1 0.026

ZECH P 1 0.026

ZEE J 1 0.026

ZEIER M 1 0.026

ZELBER-SAGI S 1 0.026

ZELLER C 1 0.026

ZELLER M 1 0.026

ZELTZ C 1 0.026

ZEN M 1 0.026

ZEN Z 1 0.026

ZENG G 1 0.026

ZENG HX 1 0.026

ZENG J 1 0.026

ZENG MH 1 0.026

ZENG R 1 0.026

ZENG S 1 0.026

ZENG T 1 0.026

ZENG W 1 0.026

ZENG WJ 1 0.026

ZENG XF 1 0.026

ZENG YB 1 0.026

ZENG-BROUWERS J 1 0.026

ZENT R 1 0.026

ZERA T 1 0.026

ZERBI P 1 0.026

ZERIN F 1 0.026

ZEUZEM S 1 0.026

ZEYTINOGLU A 1 0.026

ZEZINA L 1 0.026

ZHA H 1 0.026

ZHAI JX 1 0.026

ZHAI MT 1 0.026

ZHAI ZH 1 0.026

ZHAN GD 1 0.026

ZHAN XJ 1 0.026

ZHAN ZB 1 0.026

ZHANG A 1 0.026

ZHANG AF 1 0.026

ZHANG AQ 1 0.026

ZHANG BH 1 0.026

ZHANG BL 1 0.026

ZHANG BR 1 0.026

ZHANG BX 1 0.026

ZHANG BZ 1 0.026

ZHANG CG 1 0.026

ZHANG CL 1 0.026

ZHANG D 1 0.026

ZHANG DH 1 0.026

ZHANG DJ 1 0.026

ZHANG DN 1 0.026

ZHANG DW 1 0.026

ZHANG DY 1 0.026

ZHANG FP 1 0.026

ZHANG FR 1 0.026

ZHANG GJ 1 0.026

ZHANG GL 1 0.026

ZHANG GZ 1 0.026

ZHANG HC 1 0.026

ZHANG HN 1 0.026

ZHANG HR 1 0.026

ZHANG HT 1 0.026

ZHANG JQ 1 0.026

ZHANG JZ 1 0.026

ZHANG KB 1 0.026

ZHANG LH 1 0.026

ZHANG LP 1 0.026

ZHANG LZ 1 0.026

ZHANG MJ 1 0.026

ZHANG MS 1 0.026

ZHANG MX 1 0.026

ZHANG MY 1 0.026

ZHANG NX 1 0.026

ZHANG NY 1 0.026

ZHANG PL 1 0.026

ZHANG RH 1 0.026

ZHANG RM 1 0.026

ZHANG RR 1 0.026

ZHANG SA 1 0.026

ZHANG SB 1 0.026

ZHANG SF 1 0.026

ZHANG SX 1 0.026

ZHANG SZ 1 0.026

ZHANG WD 1 0.026

ZHANG WQ 1 0.026

ZHANG WY 1 0.026

ZHANG WZ 1 0.026

ZHANG XA 1 0.026

ZHANG XD 1 0.026

ZHANG XN 1 0.026

ZHANG XP 1 0.026

ZHANG XT 1 0.026

ZHANG YC 1 0.026

ZHANG YG 1 0.026

ZHANG YR 1 0.026

ZHANG YT 1 0.026

ZHANG YW 1 0.026

ZHANG YZ 1 0.026

ZHANG ZF 1 0.026

ZHANG ZH 1 0.026

ZHANG ZP 1 0.026

ZHANG ZW 1 0.026

ZHANG ZX 1 0.026

ZHAO C 1 0.026

ZHAO CC 1 0.026

ZHAO CF 1 0.026

ZHAO CH 1 0.026

ZHAO CS 1 0.026

ZHAO DM 1 0.026

ZHAO DX 1 0.026

ZHAO JG 1 0.026

ZHAO JM 1 0.026

ZHAO JR 1 0.026

ZHAO JS 1 0.026

ZHAO LC 1 0.026

ZHAO LP 1 0.026

ZHAO LW 1 0.026

ZHAO MG 1 0.026

ZHAO MH 1 0.026

ZHAO MM 1 0.026

ZHAO MT 1 0.026

ZHAO RZ 1 0.026

ZHAO S 1 0.026

ZHAO SR 1 0.026

ZHAO TB 1 0.026

ZHAO TV 1 0.026

ZHAO TY 1 0.026

ZHAO W 1 0.026

ZHAO WB 1 0.026

ZHAO XC 1 0.026

ZHAO XH 1 0.026

ZHAO XN 1 0.026

ZHAO XP 1 0.026

ZHAO XX 1 0.026

ZHAO XZ 1 0.026

ZHAO YC 1 0.026

ZHAO YF 1 0.026

ZHAO YG 1 0.026

ZHAO YR 1 0.026

ZHAO YX 1 0.026

ZHAO Z 1 0.026

ZHAO ZH 1 0.026

ZHAO ZJ 1 0.026

ZHAO ZM 1 0.026

ZHAO ZX 1 0.026

ZHAO ZY 1 0.026

ZHENG CL 1 0.026

ZHENG D 1 0.026

ZHENG DH 1 0.026

ZHENG GH 1 0.026

ZHENG GP 1 0.026

ZHENG HJ 1 0.026

ZHENG HL 1 0.026

ZHENG HT 1 0.026

ZHENG JF 1 0.026

ZHENG JM 1 0.026

ZHENG JY 1 0.026

ZHENG L 1 0.026

ZHENG LF 1 0.026

ZHENG LL 1 0.026

ZHENG LT 1 0.026

ZHENG M 1 0.026

ZHENG PL 1 0.026

ZHENG PX 1 0.026

ZHENG Q 1 0.026

ZHENG RP 1 0.026

ZHENG SB 1 0.026

ZHENG SL 1 0.026

ZHENG SQ 1 0.026

ZHENG SZ 1 0.026

ZHENG WC 1 0.026

ZHENG XP 1 0.026

ZHENG XX 1 0.026

ZHENG XY 1 0.026

ZHENG Y 1 0.026

ZHENG YL 1 0.026

ZHENG YW 1 0.026

ZHENG ZC 1 0.026

ZHENG ZH 1 0.026

ZHENYUKH O 1 0.026

ZHERNOVKOV V 1 0.026

ZHO ZG 1 0.026

ZHONG CD 1 0.026

ZHONG HB 1 0.026

ZHONG J 1 0.026

ZHONG JQ 1 0.026

ZHONG JY 1 0.026

ZHONG L 1 0.026

ZHONG LL 1 0.026

ZHONG MR 1 0.026

ZHONG SH 1 0.026

ZHONG T 1 0.026

ZHONG W 1 0.026

ZHONG WX 1 0.026

ZHONG XF 1 0.026

ZHOU AL 1 0.026

ZHOU BR 1 0.026

ZHOU CM 1 0.026

ZHOU DW 1 0.026

ZHOU FF 1 0.026

ZHOU GH 1 0.026

ZHOU HM 1 0.026

ZHOU HY 1 0.026

ZHOU JH 1 0.026

ZHOU JW 1 0.026

ZHOU KB 1 0.026

ZHOU LS 1 0.026

ZHOU LY 1 0.026

ZHOU ML 1 0.026

ZHOU MS 1 0.026

ZHOU MT 1 0.026

ZHOU MY 1 0.026

ZHOU Q 1 0.026

ZHOU QD 1 0.026

ZHOU R 1 0.026

ZHOU SJ 1 0.026

ZHOU SW 1 0.026

ZHOU TH 1 0.026

ZHOU WD 1 0.026

ZHOU WH 1 0.026

ZHOU WQ 1 0.026

ZHOU XL 1 0.026

ZHOU XQ 1 0.026

ZHOU XT 1 0.026

ZHOU XW 1 0.026

ZHOU XX 1 0.026

ZHOU YR 1 0.026

ZHOU YT 1 0.026

ZHOU YZ 1 0.026

ZHOU Z 1 0.026

ZHOU ZF 1 0.026

ZHOU ZQ 1 0.026

ZHOU ZY 1 0.026

ZHU BB 1 0.026

ZHU C 1 0.026

ZHU CG 1 0.026

ZHU CL 1 0.026

ZHU CX 1 0.026

ZHU F 1 0.026

ZHU GH 1 0.026

ZHU JF 1 0.026

ZHU JH 1 0.026

ZHU JQ 1 0.026

ZHU JY 1 0.026

ZHU JZ 1 0.026

ZHU LL 1 0.026

ZHU LP 1 0.026

ZHU MY 1 0.026

ZHU PP 1 0.026

ZHU Q 1 0.026

ZHU QH 1 0.026

ZHU QQ 1 0.026

ZHU RF 1 0.026

ZHU RY 1 0.026

ZHU S 1 0.026

ZHU SJ 1 0.026

ZHU WJ 1 0.026

ZHU WP 1 0.026

ZHU WW 1 0.026

ZHU XD 1 0.026

ZHU XG 1 0.026

ZHU XX 1 0.026

ZHU YF 1 0.026

ZHU YJ 1 0.026

ZHU YP 1 0.026

ZHU YQ 1 0.026

ZHU YZ 1 0.026

ZHU ZB 1 0.026

ZHU ZH 1 0.026

ZHU ZQ 1 0.026

ZHUANG J 1 0.026

ZHUANG JH 1 0.026

ZHUANG K 1 0.026

ZHUANG LH 1 0.026

ZHUANG MH 1 0.026

ZHUANG XH 1 0.026

ZHUANG Z 1 0.026

ZHUO ZX 1 0.026

ZIBARA K 1 0.026

ZICHA J 1 0.026

ZIELINSKA M 1 0.026

ZIEMIANSKI P 1 0.026

ZIETSE R 1 0.026

ZILLER N 1 0.026

ZIMBER-STROBL U 1 0.026

ZIMDAHL H 1 0.026

ZIMETBAUM P 1 0.026

ZIMMERMAN D 1 0.026

ZIMMERMANN T 1 0.026

ZIMPELMANN J 1 0.026

ZINI E 1 0.026

ZINMAN B 1 0.026

ZINN KR 1 0.026

ZIOU XH 1 0.026

ZIYADEH F 1 0.026

ZOIS NE 1 0.026

ZOLLER A 1 0.026

ZOPPI A 1 0.026

ZORNIG M 1 0.026

ZORZI A 1 0.026

ZOTTER-TUFARO C 1 0.026

ZOU C 1 0.026

ZOU HB 1 0.026

ZOU J 1 0.026

ZOU JH 1 0.026

ZOU JJ 1 0.026

ZOU LB 1 0.026

ZOU LW 1 0.026

ZOU LX 1 0.026

ZOU P 1 0.026

ZOU XZ 1 0.026

ZOU YG 1 0.026

ZOUEIN FA 1 0.026

ZOULIM F 1 0.026

ZSENGELLER ZK 1 0.026

ZU HERINGDORF DM 1 0.026

ZU YX 1 0.026

ZUBER J 1 0.026

ZUCKERMAN JE 1 0.026

ZUGRAVU A 1 0.026

ZUO BJ 1 0.026

ZUO C 1 0.026

ZUO F 1 0.026

ZUO H 1 0.026

ZUO K 1 0.026

ZUO N 1 0.026

ZUO WZ 1 0.026

ZUO XC 1 0.026

ZUO YD 1 0.026

ZURAKOWSKI D 1 0.026

ZWINGMANN C 1 0.026

ZYCBAND E 1 0.026

(0 records (0.000%) do not contain data in the field being analyzed.)
