# Supplementary figures and images for: Bibliometric Analysis of Renal Fibrosis in Diabetic Kidney Disease From 1985 to 2020
Source: Front Public Health. 2022 Feb 4;10:767591. doi: 10.3389/fpubh.2022.767591 (PMC8855938; doi:10.3389/fpubh.2022.767591)

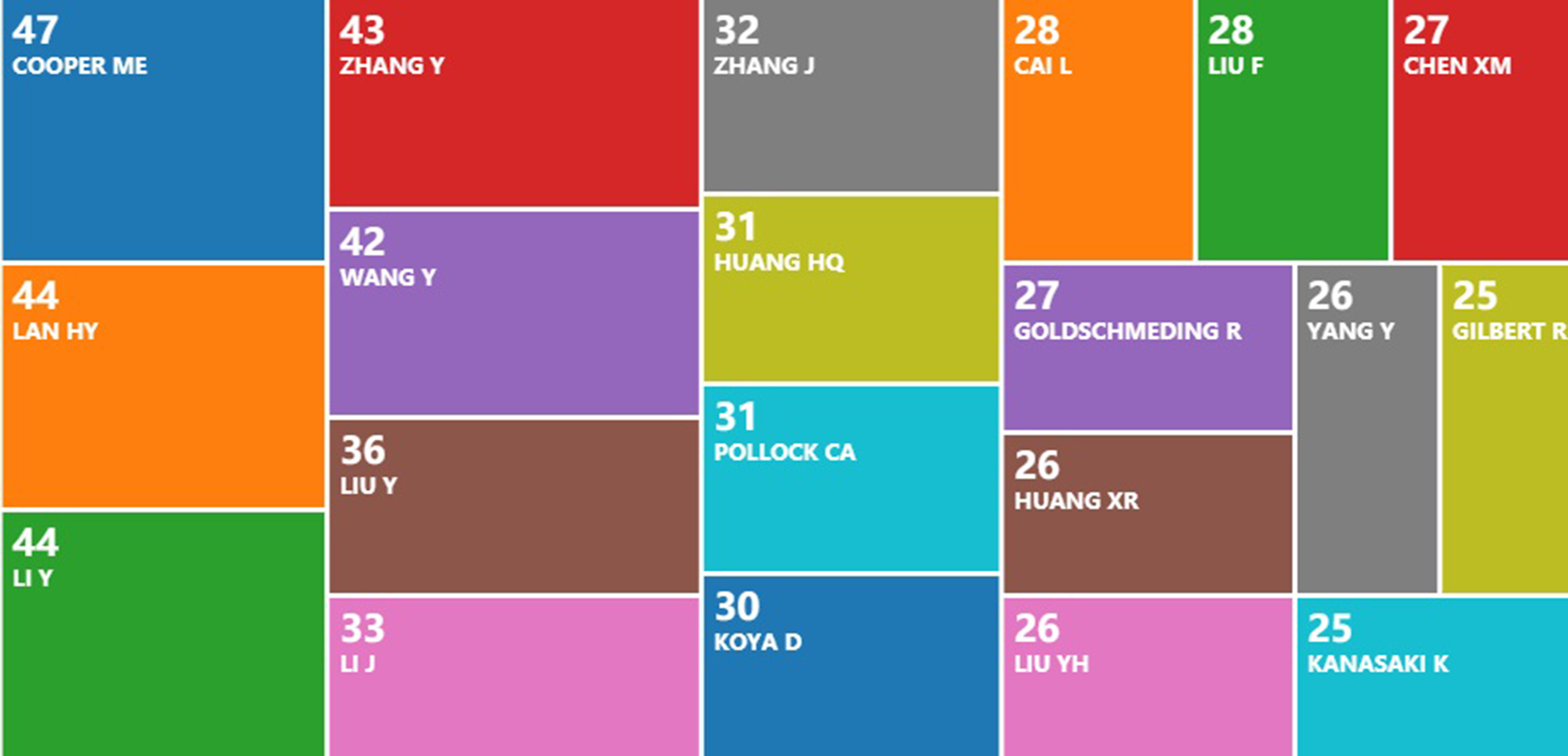

Supplement: Supplementary file 5 [file Image_1.JPEG]

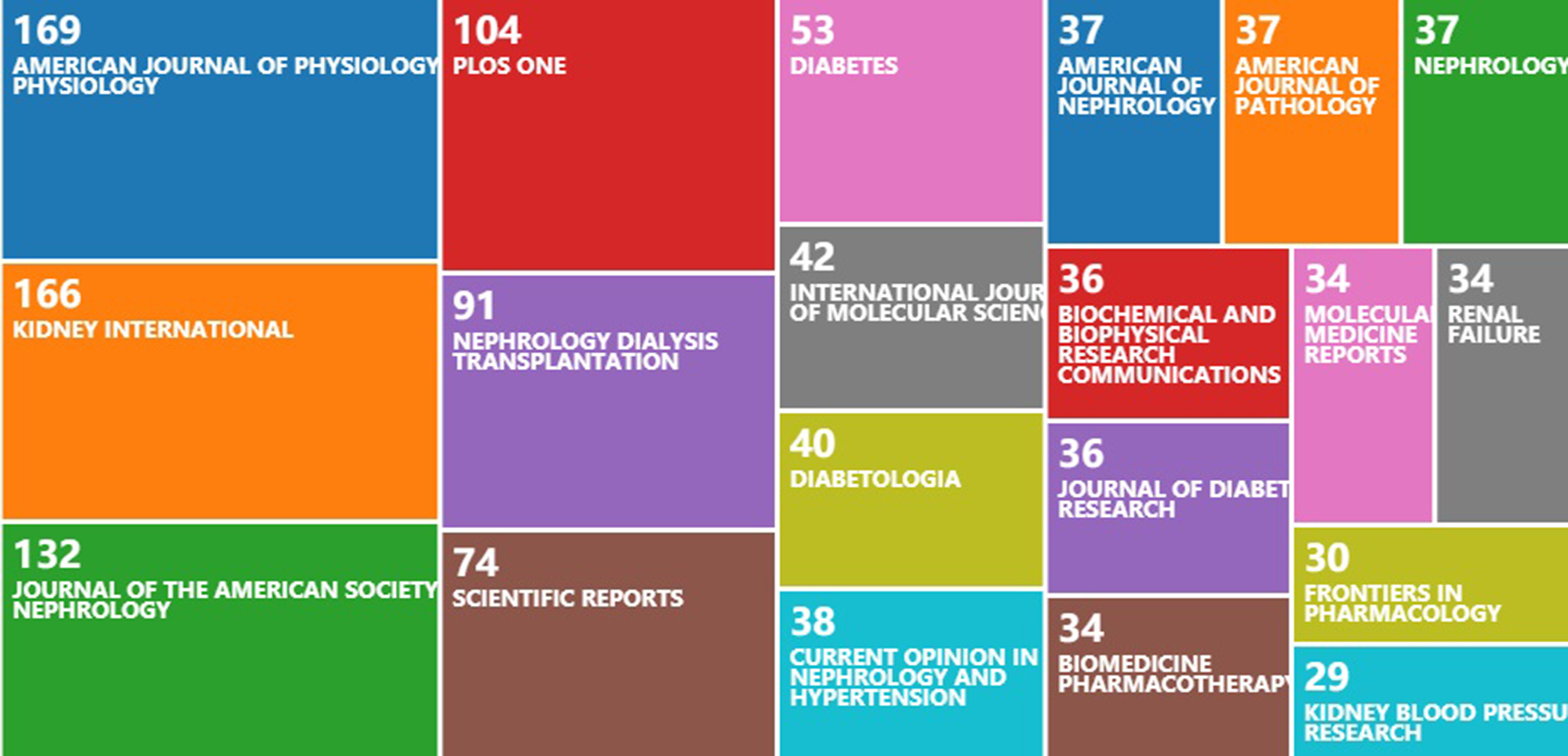

Supplement: Supplementary file 6 [file Image_2.JPEG]

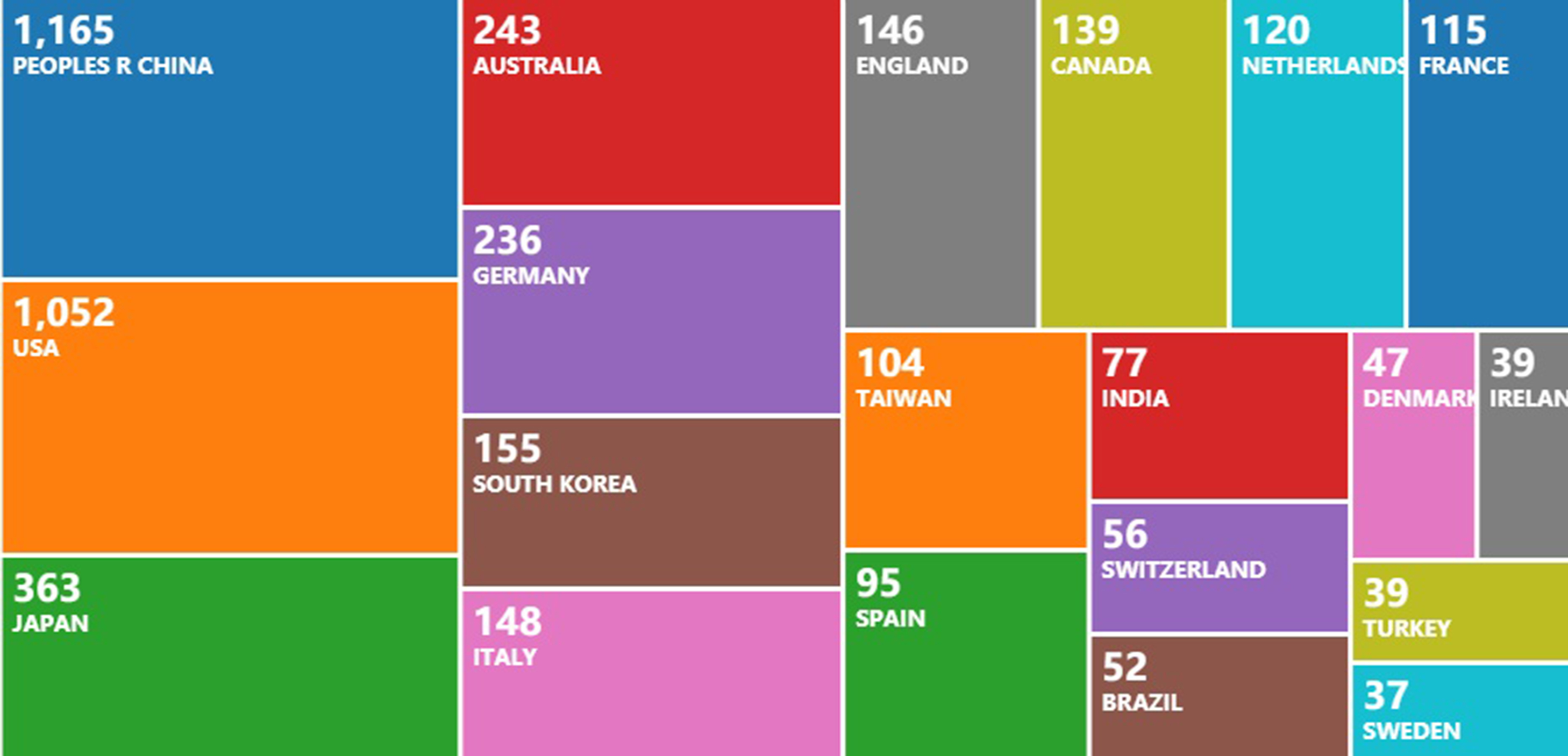

Supplement: Supplementary file 7 [file Image_3.JPEG]

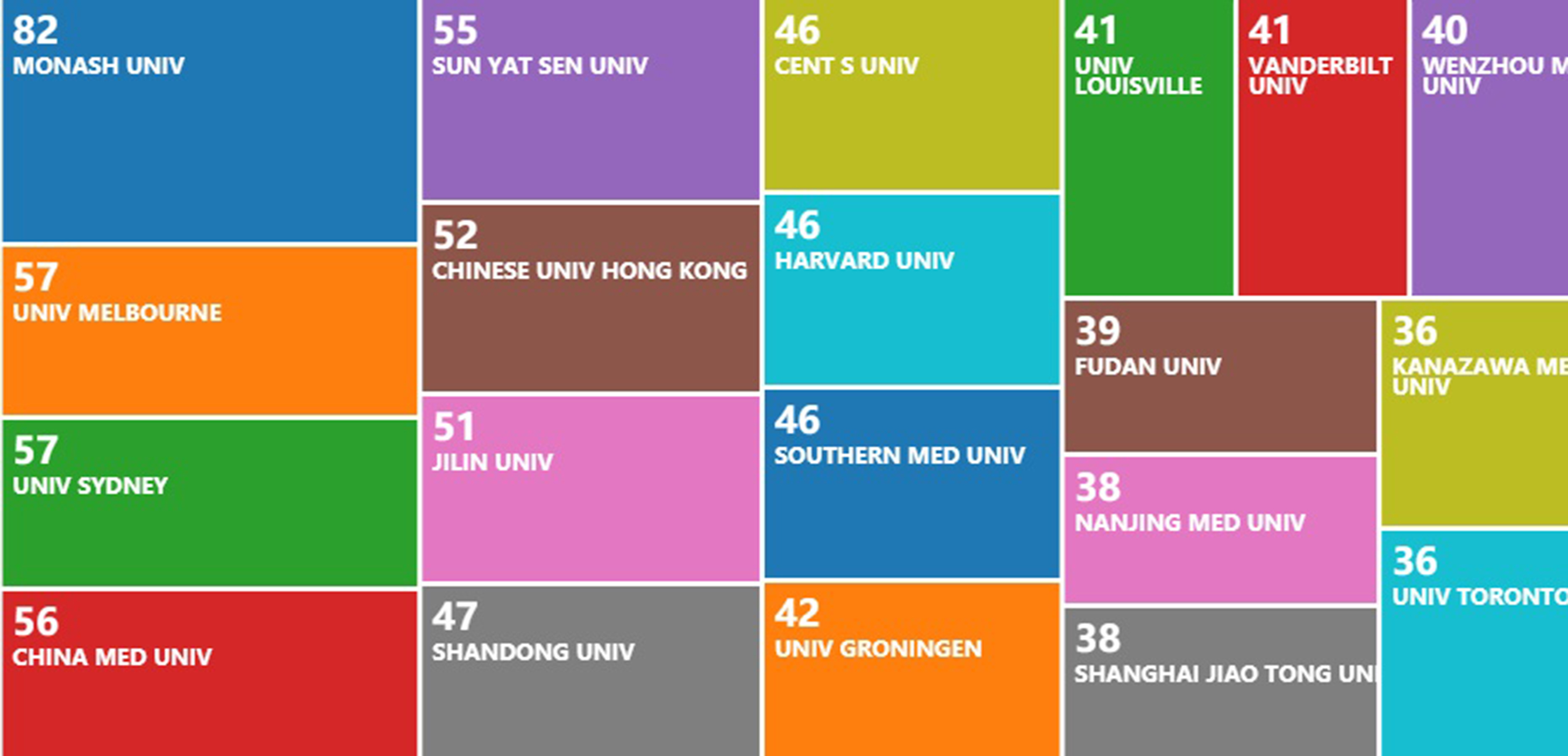

Supplement: Supplementary file 8 [file Image_4.JPEG]
